# Systematic review and meta-analysis of AI in lung cancer metastasis imaging for diagnosis and prognosis

**DOI:** 10.1038/s41746-026-02858-1

**Published:** 2026-06-05

**Authors:** Yiyao Sun, Lijun Jin, Boyi Wang, Chunna Yang, Ying Fan, Peng Zhao, Yan Zhang, Xueyan Ge, Xianzheng Sha, Juan Su, Xiran Jiang

**Affiliations:** 1 School of Intelligent Medicine, China Medical University, Shenyang, Liaoning PR China; 2 School of Control Science and Engineering, Shandong University, Jinan, Shandong PR China; 3 Department of Biomedical Engineering, School of Information Science and Technology, Fudan University, Shanghai, PR China; 4 Department of Medical Imaging, Cancer Hospital of Dalian University of Technology, Liaoning Cancer Hospital & Institute, Shenyang, Liaoning PR China; 5 Liaoning Provincial Center for Disease Prevention and Control, Shenyang, Liaoning PR China

**Keywords:** Biomarkers, Cancer, Oncology

## Abstract

Lung cancer (LC) remains the leading cause of cancer-related mortality, and distant metastases (DMs) are common. Imaging-based AI research has largely focused on primary tumors, with metastatic lesions insufficiently investigated. This study provides the first metastasis-focused quantitative synthesis of AI performance for imaging-based evaluation of DMs in LC across classification and outcome-prediction tasks, with 59 of 64 eligible reports in the meta-analysis. Task-specific meta-analysis showed pooled sensitivity, specificity, and AUC (best-performing model) of 0.88, 0.87, and 0.91 for molecular-level prediction; 0.87, 0.90, and 0.93 for metastasis differentiation; 0.89, 0.94, and 0.90 for tumor type classification; and 0.82, 0.86, and 0.89 for outcome prediction, respectively. Heterogeneity between studies was substantial (*I²* > 90% for key analyses). Subgroup analyses showed favorable performance across study settings. The findings support AI for imaging-based evaluation of DMs in LC, highlighting the need for robust and interpretable models for translation.

## Introduction

Lung cancer (LC) is one of the most common malignant cancers, with incidence ranking second and mortality ranking first globally^1^. At the time of initial diagnosis, ~40–50% of patients present with distant metastasis (DM)^2^. Disseminated tumor cells preferentially colonize organs with complex structures and high metabolic activity, such as brain, bone, and liver, where abundant vascularization and highly specialized microenvironments provide permissive niches for tumor cell survival and evolution^3^. Consequently, the reported incidences of metastases to the brain, bone, and liver are ~27%, 41.4%, and 23.9%, respectively^4^.

LC metastasis is a highly dynamic and biologically complex process, driven by multiple molecular mechanisms, including epithelial-mesenchymal transition (EMT), angiogenesis, and lymphangiogenesis^5,6^, which collectively enable tumor cells to acquire migratory, invasive, and colonizing capacities. Within organs that are both structurally and functionally complex, such as the brain^7^, bone^8^, and liver^9^, metastatic lesions engage in complex interactions with the host tissue, generating imaging features that often provide a more comprehensive and biologically informative perspective on LC evolution than that obtained from the primary pulmonary tumor alone^5^. The biological information embedded in these metastases provides essential insights into the molecular profiles and histological subtypes of LC, which are strongly associated with patient prognosis and therapeutic response^10^. Although histopathological examination remains the gold standard for characterizing tumor architecture and the surrounding microenvironment, obtaining tissue from distant metastatic sites, particularly from eloquent neurological structures such as the brain parenchyma or spinal cord, presents significant technical challenges and carries a notable risk of neurological complications^11,12^. These limitations highlight the need for developing noninvasive approaches capable of characterizing DMs.

**Fig. 1:**
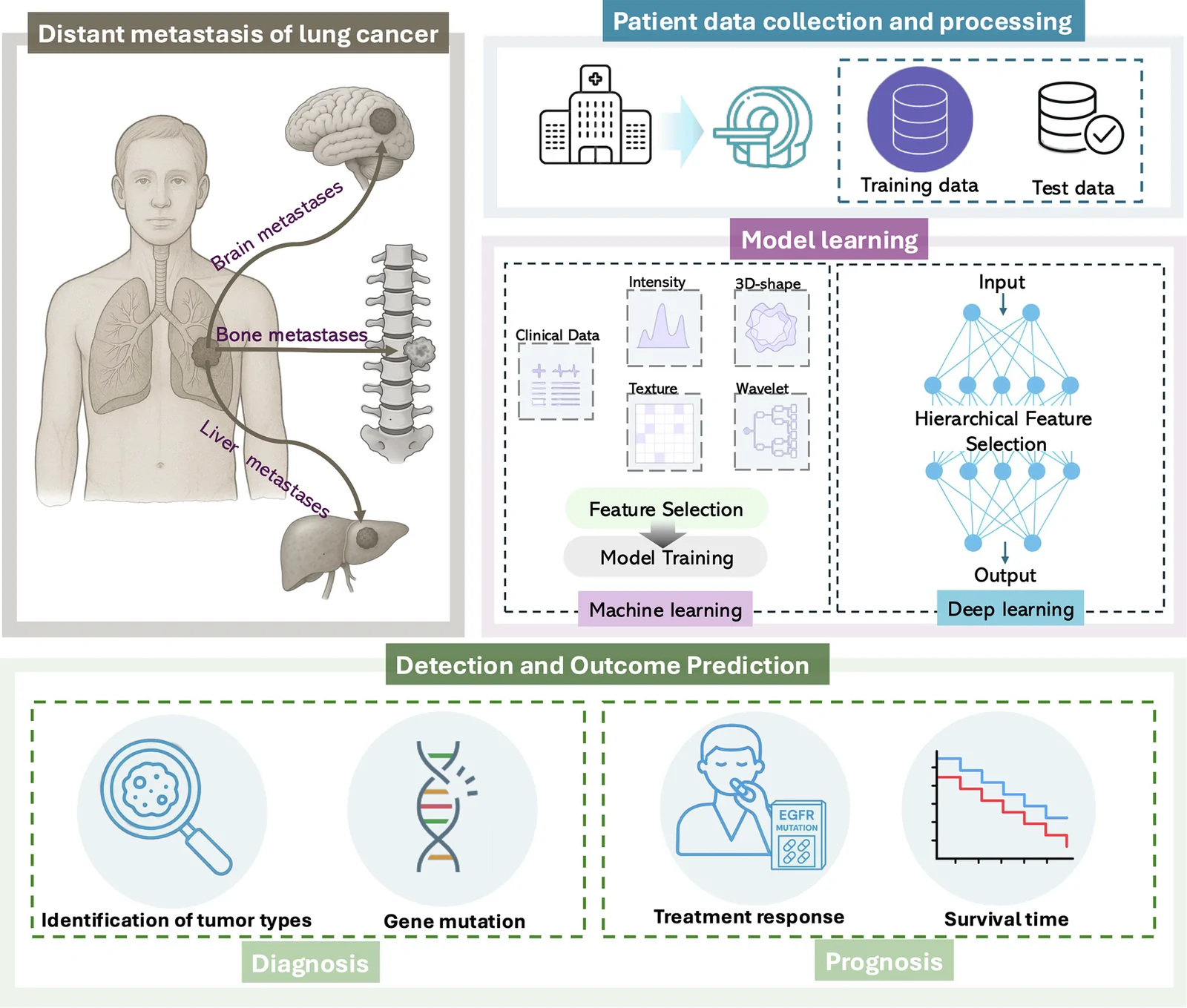
Conceptual workflow of AI-driven imaging analysis of distant metastases in LC. The diagram illustrates the overall framework of the included studies, covering patient data collection and preprocessing, AI model development, and major downstream tasks.

The rapid advancement of medical imaging technologies has driven the development of high-resolution and wide-coverage imaging techniques for the visualization of critical organs. Compared to conventional tissue biopsy, medical imaging provides a noninvasive method for the comprehensive assessment of tumor burden and dynamic monitoring of disease progression^12^. However, routine clinical image interpretation remains largely qualitative beyond simple size measurements^13^, leading to subjectivity, inter-reader variability, and inefficiency that can compromise timely and accurate decision-making^14^. Artificial intelligence (AI) has begun to address these limitations by objectively extracting information beyond human perception, thereby advancing the automated analysis of radiological data in oncology^15^. In particular, radiomics enables high-dimensional, quantitative characterization of tumors and their surrounding tissues based on conventional imaging, capturing multiparametric features such as texture, morphology, and intensity to reflect intratumoral heterogeneity^16^. Building on this foundation, deep-learning architectures, including convolutional neural networks and Transformers, have further enhanced automated feature learning, imaging-molecular association modeling and outcome prediction^17,18^. Due to the rich biological information embedded in the imaging of DMs in LC, AI-driven imaging studies have rapidly expanded from simple lesion detection to more biologically informative tasks in diagnosis and prognosis, including molecular mutation prediction, pathological subtype classification^19^, metastatic site discrimination^20^ and prognostic evaluation^21^. The overall analytical workflow of this study is summarized in Fig. 1.

To the best of our knowledge, there is still a lack of quantitative synthesis summarizing the available evidence on AI performance across imaging of DMs in these important and diverse aspects. Existing studies^22^ have primarily focused on primary lung lesions while overlooking the critical impact of these metastatic sites on disease progression and therapeutic outcomes. Therefore, this study conducts a comprehensive and detailed systematic review and meta-analysis to summarize and assess existing AI-based studies on the imaging of DMs in LC, focusing on diagnosis and outcome evaluation. The aim of the study is to summarize the current research landscape, evaluate methodological quality and variability of existing studies and analyze tumor heterogeneity so as to provide recommendations for future investigations.

## Results

### Study selection and characteristics of eligible studies

**Fig. 2:**
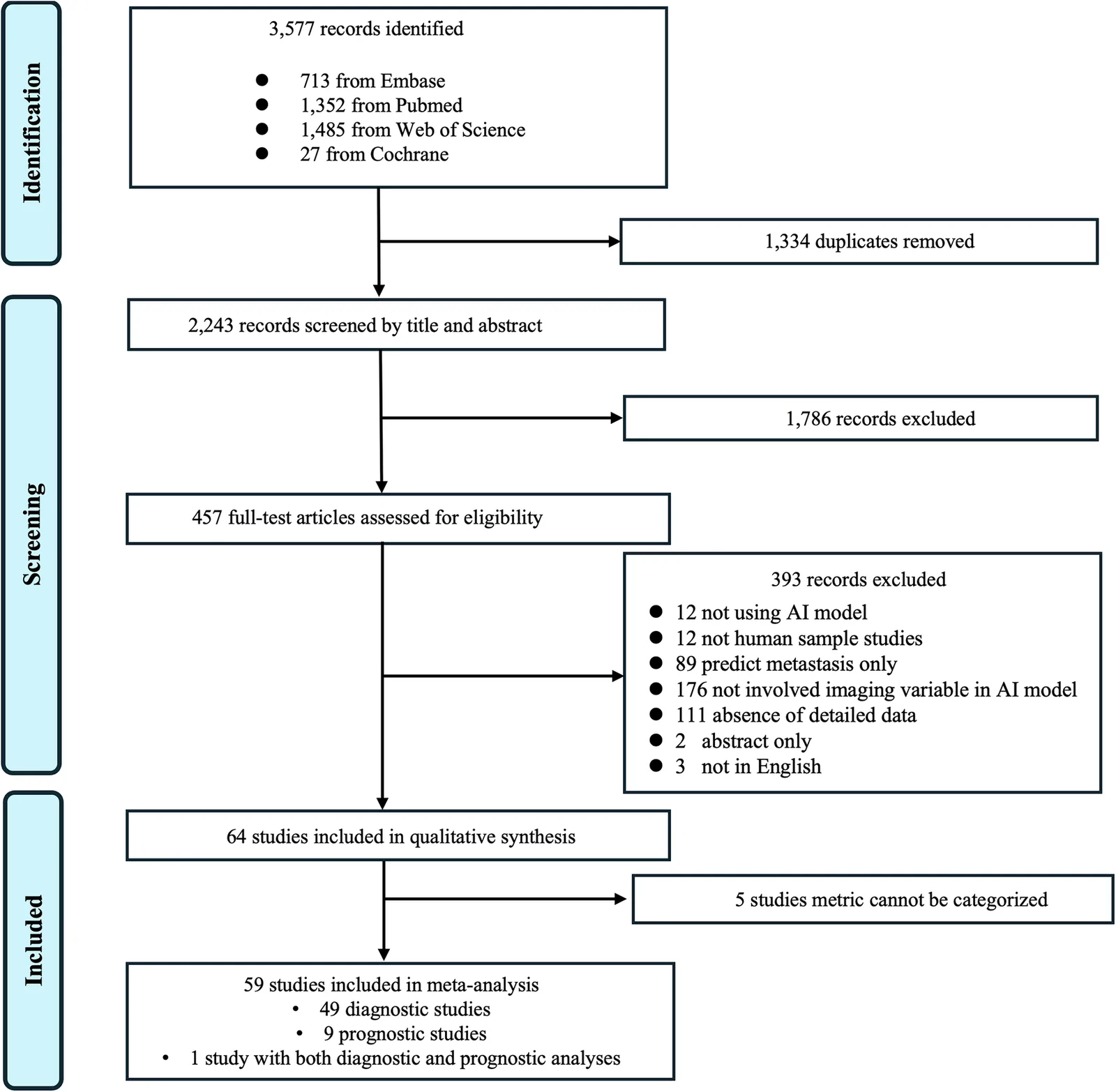
PRISMA flow diagram of the study selection process. The diagram illustrates the identification, screening, eligibility assessment, and final inclusion of studies investigating artificial intelligence applications in imaging analysis of distant metastases in LC.

A total of 3577 studies were initially identified through the database search. After removing 1334 duplicates, 2243 unique records were screened based on title and abstract. Subsequently, 460 full-text articles were assessed for eligibility, of which 393 were excluded for failing to meet the inclusion criteria. Ultimately, 64 studies were included in the systematic review. After excluding 5 studies that did not provide sufficient data and screening for potential cohort overlap to minimize duplicate patient inclusion, 59 studies were retained for the final meta-analysis, of which 49 reported only diagnostic tasks, 9 reported only prognostic (Outcome prediction) tasks, and 1 reported both diagnostic and prognostic results (Fig. 2).

### Summary of demographic characteristics and AI model details

This study systematically summarized and analyzed the demographic characteristics and AI model details of the included studies. Most of the studies were conducted in East Asian populations, primarily from China and South Korea. The mean patient age was 61.4 ± 14.3 years, with sample sizes varying from fewer than 50 to over 700 cases. Imaging data for brain and bone metastases were predominantly based on MRI, with the most common sequences being T1-weighted contrast-enhanced (T1CE), T2-weighted (T2WI), and FLAIR, whereas CT was primarily used for liver metastases, typically acquired prior to treatment. Almost all studies excluded cases with poor image quality or severe artifacts to ensure data reliability. Methodologically, most studies employed traditional ML approaches, such as LASSO combined with logistic regression, support vector machines (SVM), and random forests (RF). In recent years, there has been an increasing adoption of DL frameworks, including architectures such as ResNet and EfficientNet. Most studies used 5-fold or 10-fold cross-validation, while only about one-third incorporated external validation datasets, indicating that the generalizability of current models remains limited. Detailed characteristics of the included studies are summarized in Supplementary Tables 1–4. Detailed all-model AI performance results are provided as task-based 2×2 contingency tables in Supplementary Tables 5, 6, comprising a total of 409 tables (250 for Molecular-level prediction, 63 for Metastasis differentiation, 37 for Tumor type classification, and 59 for Outcome prediction).

**Fig. 3:**
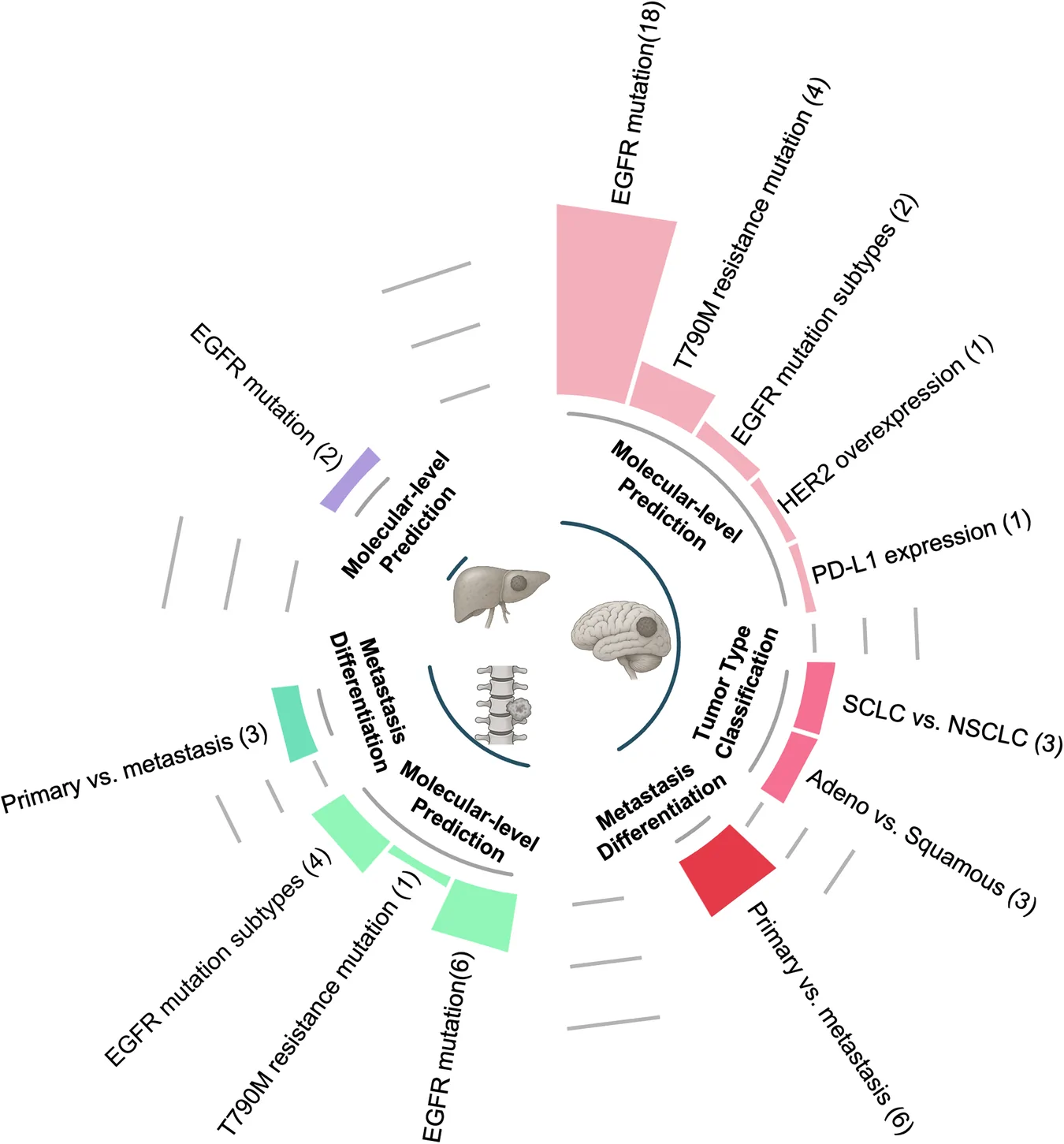
Distribution of diagnostic AI studies across metastatic sites and task categories in LC. The diagram illustrates the number of included diagnostic studies according to metastatic site (brain, bone, and liver).

For the diagnostic-task studies of Molecular-level prediction task, Metastasis differentiation task, and Tumor type classification task included in this meta-analysis, we conducted a statistical analysis stratified by metastatic site. This included 36 studies on brain metastases, 13 on bone metastases, and 2 on liver metastases (Fig. 3). Overall, the diagnostic studies primarily focused on Molecular-level prediction. Among these, the prediction of EGFR mutation status was the most common objective, reported in 26 studies (18 on brain, 6 on bone, and 2 on liver metastases), followed by the prediction of T790M resistance mutations (5 studies: 4 on brain and 1 on bone metastases). Some studies also investigated other molecular biomarkers, including EGFR exon subtypes, HER2, and PD-L1 expression. The second major research focus was Tumor type classification, primarily distinguishing small-cell lung cancer (SCLC) versus non-small-cell lung cancer (NSCLC) (3 studies) and adenocarcinoma versus squamous carcinoma (3 studies). In addition, Metastasis differentiation, aimed at distinguishing primary from metastatic lesions, was another significant area of research, involving 9 studies (6 on brain and 3 on bone metastases). For Outcome prediction task, all imaging data were derived from brain metastases, primarily focusing on evaluating treatment efficacy and survival outcomes under various therapeutic strategies, including EGFR-TKI targeted therapy, immune checkpoint inhibitors, and radiotherapy modalities such as SRS, GKRS, and WBRT. This trend indicates that current AI-based research has predominantly focused on metastatic sites with more distinct imaging phenotypes and higher clinical relevance, whereas investigations into DMs in other organs remain relatively limited.

### Technology readiness level (TRL)

**Fig. 4:**
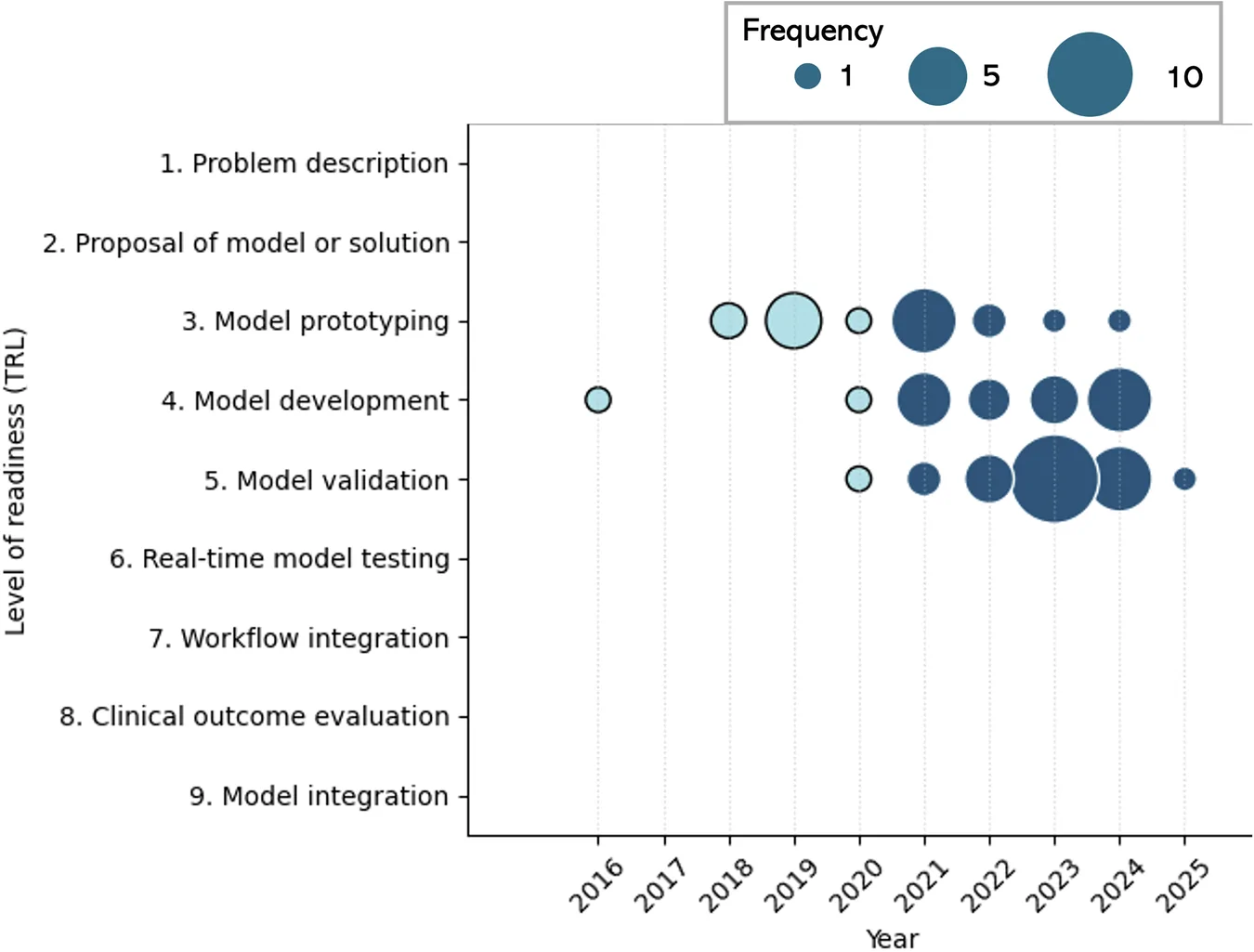
Distribution of studies across various technology readiness levels. The bubble plot summarizes the number of studies published each year across technology readiness level (TRL) categories, with bubble size representing study frequency.

The results showed high research activity but relatively low maturity across the literature, with most studies remaining at TRL ≤ 5, corresponding to algorithm development, internal validation, or retrospective testing. This suggests that although AI applications for imaging-based DMs in lung cancer are increasingly common and conceptually well developed, real-world validation and clinical implementation remain limited. Overall, the field is characterized by extensive methodological exploration but insufficient progress toward clinical deployment. The distribution of TRL levels across studies is summarized in Fig. 4.

### Quality assessment

The results of the QUADAS-AI quality assessment are summarized in Supplementary Fig. 1. Among the 64 studies evaluated, most demonstrated a low risk of bias in the domains of patient selection, reference standard, and flow and timing. However, due to the lack of external validation, the index test domain frequently showed a high risk of bias and concerns regarding applicability. Detailed evaluations for each quality dimension are provided in Supplementary Fig. 2.

### Overall performance of AI algorithms

**Fig. 5:**
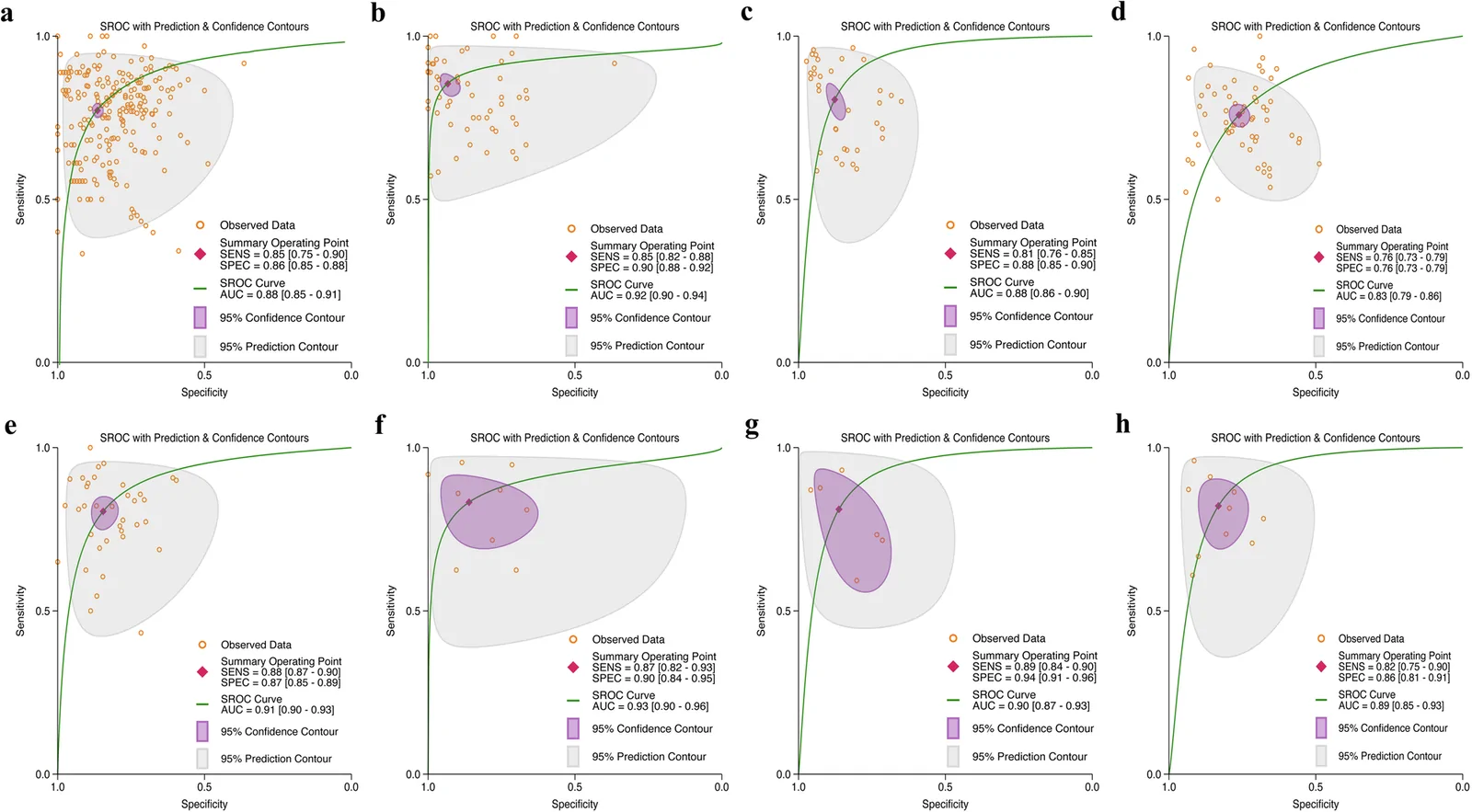
Summary receiver operating characteristic (SROC) curves of the AI models across different analytical tasks. **a** Molecular-level prediction for all models (36 studies with 250 tables), **b** Metastasis differentiation for all models (9 studies with 63 tables), **c** Tumor type classification for all models (6 studies with 37 tables), **d** Outcome prediction for all models (10 studies with 29 tables), **e** Molecular-level prediction for the best-performing model (36 studies with 36 tables), **f** Metastasis differentiation for the best-performing model (9 studies with 9 tables), **g** Tumor type classification for the best-performing model (6 studies with 6 tables), and **h** Outcome prediction for the best-performing model (10 studies with 10 tables). SROC summary receiver operating characteristic, SENS summary sensitivity, SPEC summary specificity.

The SROC curves in Fig. 5 illustrate performance of the AI models for four tasks (panels a, b, c, d for all models; panels e, f, g, h for the best-performing models). Molecular-level prediction task included 36 studies with pooled SEN/SPE values of 0.85/0.86, with an AUC of 0.88 (95% CI 0.85–0.91) in all models. For the best-performing model, and with pooled SEN/SPE values of 0.88/0.87, with an AUC of 0.91 (95% CI 0.90–0.93). Metastasis differentiation task included 9 studies with pooled SEN/SPE values of 0.85/0.90, with an AUC of 0.92 (95% CI 0.90–0.94) in all models. For the best-performing model with pooled SEN/SPE values of 0.87/0.90, with an AUC of 0.93 (95% CI 0.90–0.96). Tumor type classification task included 6 studies with pooled SEN/SPE values of 0.81/0.88, with an AUC of 0.88 (95% CI 0.86–0.90). For the best-performing model, with pooled SEN/SPE values of 0.89/0.94, with an AUC of 0.90 (95% CI 0.87–0.93). Similarly, Outcome prediction task included 10 studies performance with pooled SEN/SPE values of 0.76/0.76, with an AUC of 0.83 (95% CI 0.79–0.86) in all models. For the best-performing model, with pooled SEN/SPE values of 0.82/0.86, with an AUC of 0.89 (95% CI 0.85–0.93). We observed that across the four task categories, AI models achieved relatively better performance in the metastasis differentiation task. Although the overall performance of Outcome prediction task was slightly lower than that of the other tasks, they still demonstrated satisfactory predictive accuracy.

### Subgroup meta-analyses

Table 1 summarizes the pooled diagnostic performance for Molecular-level prediction. In the Metastatic lesion subgroup (the Brain subgroup showed better performance), pooled SEN/SPE were 0.89/0.89 for brain metastases, 0.86/0.84 for bone metastases, and 0.86/0.80 for liver metastases, with corresponding AUCs of 0.93 (95% CI 0.91–0.95), 0.90 (95% CI 0.89–0.90), and 0.88 (95% CI 0.81–0.95), respectively (Supplementary Figs. 3, 24, 45, 66). For Molecular subtype classification subgroup, pooled SEN/SPE/AUC were 0.87/0.86/0.91 (95% CI 0.88–0.93) for EGFR mutation; 0.89/0.84/0.93 (95% CI 0.91–0.96) for EGFR mutation subtypes; 0.86/0.93/0.94 (95% CI 0.92–0.95) for T790M resistance mutation; 0.92/0.93/0.97 (95% CI 0.96–0.98) for HER2 overexpression; and 0.95/0.70/0.83 (95% CI 0.67–0.99) for PD-L1 expression (Supplementary Figs. 4, 25, 46, 67). For the Lesion-level ROI subgroup, Primary-metastatic models demonstrated slightly higher performance than Metastatic-only models, with pooled SEN/SPE of 0.86/0.88 and 0.88/0.84, and AUCs of 0.92 (95% CI 0.91–0.93) and 0.90 (95% CI 0.88–0.92), respectively (Supplementary Figs. 5, 26, 47, 68). In the Local metastatic ROI subgroup, Intra-peritumoral models achieved slightly better performance than Intratumoral models, with pooled SEN/SPE of 0.90/0.88 and 0.87/0.85, and AUCs of 0.91 (95% CI 0.89–0.93) and 0.90 (95% CI 0.88–0.92), respectively (Supplementary Figs. 6, 27, 48, 69). When comparing algorithms, DL models exhibited higher performance than ML models with pooled SEN/SPE of 0.92/0.89 and 0.87/0.87, and AUCs of 0.93 (95% CI 0.89–0.96) and 0.91 (95% CI 0.89–0.93), respectively (Supplementary Figs. 7, 28, 49, 70). In the Cohort subgroup, internal and external validation demonstrated nearly identical performance, with SEN/SPE of 0.87/0.86 and 0.89/0.87, and AUCs of 0.90 (95% CI 0.88–0.94) and 0.91 (95% CI 0.89–0.93), respectively (Supplementary Figs. 8, 29, 50, 71). Finally, in the Feature-source subgroup, Imaging-clinical integrated models showed higher performance than Imaging-only models with pooled SEN/SPE of 0.90/0.88 and 0.88/0.85, and AUCs of 0.92 (95% CI 0.89–0.95) and 0.89 (95% CI 0.87–0.91), respectively (Supplementary Figs. 9, 30, 51, 72).

**Table 1:** Summary estimates of pooled diagnostic performance for molecular-level prediction task of AI in DMs imaging in LC

Subgroup	No. of studies	Sensitivity			Specificity			*P*^b^	AUC (95% CI)
		Sensitivity	*P*^a^	*I*^*2*^ (95% CI)	Specificity	*P*^a^	*I*^*2*^ (95% CI)		
Overall	36	0.88 (0.87, 0.90)	<0.05	99.1 (98.9–99.3)	0.87 (0.85, 0.89)	<0.05	97.9 (97.5–98.4)		0.91 (0.90, 0.93)
Metastatic lesion								<0.05	
Brain	24	0.89 (0.87, 0.91)	<0.05	97.3 (96.9–97.7)	0.89 (0.87, 0.92)	<0.05	98.6 (98.2–99.1)		0.93 (0.91, 0.95)
Bone	10	0.86 (0.83, 0.89)	<0.05	93.1 (92.4–93.8)	0.84 (0.80, 0.88)	<0.05	62.6 (62.0–63.4)		0.90 (0.89, 0.90)
Liver	2	0.86 (0.81, 0.92)	<0.05	63.9 (60.0–66.2)	0.80 (0.76, 0.84)	<0.05	60.0 (59.4–60.8)		0.88 (0.81, 0.95)
Molecular subtype classification								<0.05	
EGFR mutation	23	0.87 (0.83, 0.90)	<0.05	98.8 (98.4–99.2)	0.86 (0.82, 0.89)	<0.05	97.8 (97.4–98.3)		0.91 (0.88, 0.93)
EGFR mutation subtypes	6	0.89 (0.85, 0.93)	<0.05	91.0 (90.4–91.7)	0.84 (0.79, 0.86)	<0.05	96.0 (95.6–96.5)		0.93 (0.91, 0.96)
T790M resistance mutation	5	0.86 (0.77, 0.94)	<0.05	95.4 (94.9–96.0)	0.93 (0.90, 0.97)	<0.05	85.0 (84.5–85.7)		0.94 (0.92, 0.95)
HER2 overexpression	1	0.92 (0.91, 0.93)	–	–	0.93 (0.92, 0.94)	–	–		0.97 (0.96, 0.98)
PD- L1 expression	1	0.95 (0.91, 0.99)	–	–	0.70 (0.46, 0.94)	–	–		0.83 (0.67, 0.99)
Lesion-level ROI								<0.05	
Primary-metastatic	5	0.88 (0.84, 0.92)	<0.05	67.5 (65.2–69.8)	0.88 (0.81, 0.95)	<0.05	84.6 (84.1–85.3)		0.92 (0.91, 0.93)
Metastatic	31	0.86 (0.84, 0.88)	<0.05	98.1 (96.6–99.6)	0.84 (0.82, 0.86)	<0.05	98.2 (97.8–98.7)		0.90 (0.88, 0.92)
Local metastatic ROI								<0.05	
Intratumoral	18	0.87 (0.85, 0.89)	<0.05	99.0 (98.5–99.5)	0.85 (0.83, 0.87)	<0.05	97.5 (97.1–98.0)		0.90 (0.88, 0.92)
Intra-peritumoral	18	0.90 (0.86, 0.94)	<0.05	98.1 (96.9–99.3)	0.88 (0.84, 0.92)	<0.05	97.6 (97.2–98.1)		0.91 (0.89, 0.93)
Algorithm								<0.05	
ML	30	0.87 (0.85, 0.89)	<0.05	96.1 (94.2–98.0)	0.87 (0.85, 0.89)	<0.05	97.7 (97.3–98.2)		0.91 (0.89, 0.93)
DL	6	0.92 (0.88, 0.95)	<0.05	91.9 (89.9–93.9)	0.89 (0.82, 0.96)	<0.05	97.8 (97.4–98.3)		0.93 (0.89, 0.96)
Cohort								<0.05	
Internal validation cohort	15	0.87 (0.85, 0.89)	<0.05	99.2 (97.7–99.9)	0.86 (0.83, 0.90)	<0.05	97.9 (97.5–98.4)		0.90 (0.88, 0.94)
External validation cohort	21	0.89 (0.87, 0.91)	<0.05	98.0 (96.5–99.5)	0.87 (0.84, 0.91)	<0.05	97.8 (97.4–98.3)		0.91 (0.89, 0.93)
Feature-source								<0.05	
Imaging-only	22	0.88 (0.86, 0.90)	<0.05	97.4 (95.8–99.0)	0.85 (0.83, 0.87)	<0.05	98.7 (98.3–99.2)		0.89 (0.87, 0.91)
Imaging-clinical integrated	14	0.90 (0.87, 0.92)	<0.05	83.6 (81.4–85.8)	0.88 (0.84, 0.92)	<0.05	89.5 (89.0–90.1)		0.92 (0.89, 0.95)

*DMs* distant metastases, *LC* lung cancer, *ROI* region of interest, *ML* machine learning, *DL* deep learning, *CI* confidence interval.

^a^*P*-value for heterogeneity within each subgroup.

^b^*P*-value for heterogeneity between subgroups with meta-regression analysis.

Table 2 summarizes the pooled diagnostic performance for Metastasis differentiation. In the Metastatic lesion subgroup, the Brain metastasis tended to exhibit higher pooled estimates than the Bone metastasis, with pooled SEN/SPE of 0.93/0.95 and 0.87/0.89, and AUCs of 0.95 (95% CI 0.93–0.98) and 0.94 (95% CI 0.89–0.99), respectively (Supplementary Figs. 10, 31, 52, 73). For the Lesion-level ROI subgroup, Primary-metastatic models demonstrated higher pooled performance than Metastatic-only models, with pooled SEN/SPE of 0.88/0.95 and 0.84/0.88, and AUCs of 0.95 (95% CI 0.89–0.99) and 0.93 (95% CI 0.89–0.96), respectively (Supplementary Figs. 11, 32, 53, 74). In the Local metastatic ROI subgroup, intra- and peritumoral combined models performed better than intratumoral-only models, with pooled SEN/SPE of 0.91/0.89 and 0.86/0.88, and AUCs of 0.93 (95% CI 0.89–0.97) and 0.91 (95% CI 0.89–0.93), respectively (Supplementary Figs. 12, 33, 54, 75). When comparing algorithms, DL models demonstrated relatively higher performance compared with ML models, with pooled SEN/SPE of 0.86/0.92 and 0.84/0.87, and AUCs of 0.94 (95% CI 0.90–0.99) and 0.93 (95% CI 0.89–0.97), respectively (Supplementary Figs. 13, 34, 55, 76). In the Cohort subgroup, internal and external validation showed largely comparable performance, with pooled SEN/SPE of 0.86/0.90 and 0.87/0.90, and AUCs of 0.94 (95% CI 0.89–0.98) and 0.92 (95% CI 0.89–0.95), respectively (Supplementary Figs. 14, 35, 56, 77). Finally, in the Feature-source subgroup, Imaging-clinical integrated models were associated with higher pooled performance than Imaging-only models, with pooled SEN/SPE of 0.89/0.88 and 0.88/0.86, and AUCs of 0.91 (95% CI 0.85–0.97) and 0.90 (95% CI 0.88–0.92), respectively (Supplementary Figs. 15, 36, 57, 78).

**Table 2:** Summary estimates of pooled diagnostic performance for metastasis differentiation task of AI in DMs imaging in LC

Subgroup	No. of studies	Sensitivity			Specificity			*P*^b^	AUC (95% CI)
		Sensitivity	*P*^a^	*I*^*2*^ (95% CI)	Specificity	*P*^a^	*I*^*2*^ (95% CI)		
Overall	9	0.87 (0.82, 0.93)	<0.05	98.6 (98.3–98.9)	0.90 (0.84, 0.95)	<0.05	96.9 (96.6–97.1)		0.93 (0.90, 0.96)
Metastatic lesion								<0.05	
Brain	6	0.93 (0.88, 0.97)	<0.05	77.7 (77.4–78.0)	0.95 (0.93, 0.97)	<0.05	79.5 (79.2–79.7)		0.95 (0.93, 0.98)
Bone	3	0.87 (0.82, 0.91)	<0.05	94.0 (93.7–94.3)	0.89 (0.80, 0.98)	<0.05	97.9 (97.6–98.1)		0.94 (0.89, 0.99)
Lesion-level ROI								<0.05	
Primary-metastatic	2	0.88 (0.78, 0.99)	<0.05	63.0 (62.7–63.3)	0.95 (0.89, 0.99)	0.162	48.8 (48.5–49.0)		0.95 (0.89, 0.99)
Metastatic	7	0.84 (0.83, 0.85)	<0.05	94.9 (94.6–95.2)	0.88 (0.81, 0.95)	<0.05	94.9 (94.6–95.1)		0.93 (0.89, 0.96)
Local metastatic ROI								<0.05	
Intratumoral	8	0.86 (0.83, 0.90)	<0.05	91.7 (91.4–92.0)	0.88 (0.84, 0.92)	<0.05	96.9 (96.6–97.1)		0.91 (0.89, 0.93)
Intra-peritumoral	1	0.91 (0.82, 0.99)	–	–	0.89 (0.83, 0.97)	–	–		0.93 (0.89, 0.97)
Algorithm								<0.05	
ML	5	0.84 (0.82, 0.86)	<0.05	91.1 (90.8–91.4)	0.87 (0.80, 0.95)	<0.05	92.6 (92.3–92.8)		0.93 (0.89, 0.97)
DL	4	0.86 (0.76, 0.96)	<0.05	90.9 (90.6–91.2)	0.92 (0.84, 0.99)	<0.05	91.0 (90.7–91.2)		0.94 (0.90, 0.99)
Cohort								<0.05	
Internal validation cohort	4	0.86 (0.82, 0.90)	<0.05	93.1 (92.8–93.4)	0.90 (0.82, 0.97)	<0.05	97.2 (96.9–97.4)		0.93 (0.90, 0.97)
External validation cohort	5	0.87 (0.78, 0.96)	<0.05	90.3 (90.0–90.6)	0.90 (0.82, 0.98)	<0.05	94.9 (94.6–95.1)		0.94 (0.89, 0.98)
Feature-source								<0.05	
Imaging-only	6	0.88 (0.81, 0.95)	<0.05	89.0 (88.7–89.3)	0.86 (0.82, 0.90)	<0.05	95.9 (95.6–96.1)		0.90 (0.88, 0.92)
Imaging-clinical integrated	3	0.89 (0.80, 0.98)	<0.05	63.7 (63.4–64.0)	0.88 (0.81, 0.95)	0.077	61.1 (60.8–61.3)		0.91 (0.85, 0.97)

*DMs* distant metastases, *LC* lung cancer, *ROI* region of interest, *ML* machine learning, *DL* deep learning, *CI* confidence interval.

^a^*P*-value for heterogeneity within each subgroup.

^b^*P*-value for heterogeneity between subgroups with meta-regression analysis.

Table 3 summarizes the pooled performance of AI models in the imaging-based Tumor type classification task for DMs in LC. In Histologic subtype classification subgroup, the pooled SEN/SPE for Adeno/Squamous was 0.88/0.93, with an AUC of 0.90 (95% CI 0.87–0.93), while for SCLC/NSCLC, the pooled SEN/SPE was 0.87/0.88, with an AUC of 0.87 (95% CI 0.76–0.98) (Supplementary Figs. 16, 37, 58, 79). When comparing algorithms, DL models showed higher pooled performance than ML models, with pooled SEN/SPE of 0.90/0.94 and 0.76/0.92, and AUCs of 0.91 (95% CI 0.88–0.94) and 0.83 (95% CI 0.75–0.91), respectively (Supplementary Figs. 17, 38, 59, 80). In the Cohort subgroup, internal and external validation showed nearly comparable performance, with pooled SEN/SPE of 0.85/0.87 and 0.87/0.89, and AUCs of 0.88 (95% CI 0.84–0.92) and 0.90 (95% CI 0.86–0.94), respectively (Supplementary Figs. 18, 39, 60, 81). Finally, in the Feature-source subgroup, Imaging-clinical integrated models demonstrated better performance than Imaging-only models, with pooled SEN/SPE of 0.88/0.93 and 0.87/0.92, and AUCs of 0.90 (95% CI 0.80–0.99) and 0.89 (95% CI 0.83–0.95), respectively (Supplementary Figs. 19, 40, 61, 82).

**Table 3:** Summary estimates of pooled diagnostic performance for tumor type classification task of AI in DMs imaging in LC

Subgroup	No. of studies	Sensitivity			Specificity			*P*^b^	AUC (95% CI)
		Sensitivity	*P*^a^	*I*^*2*^ (95% CI)	Specificity	*P*^a^	*I*^*2*^ (95% CI)		
Overall (brain)	6	0.89 (0.84, 0.90)	<0.05	75.8 (75.5–76.1)	0.94 (0.91, 0.96)	<0.05	78.9 (78.6–79.2)		0.90 (0.87, 0.93)
Histologic subtype classification								<0.05	
Adeno/Squamous	3	0.88 (0.80, 0.97)	<0.05	72.1 (71.8–72.4)	0.93 (0.90, 0.96)	<0.05	60.0 (59.7–60.3)		0.90 (0.87, 0.93)
SCLC/NSCLC	3	0.87 (0.75, 0.99)	0.084	59.6 (59.3–59.9)	0.88 (0.75, 0.99)	0.064	62.1 (61.8–62.4)		0.87 (0.76, 0.98)
Algorithm								<0.05	
ML	1	0.76 (0.62, 0.90)	–	–	0.92 (0.86, 0.98)	–	–		0.83 (0.75, 0.91)
DL	5	0.90 (0.85, 0.95)	<0.05	75.4 (75.1–75.7)	0.94 (0.91, 0.96)	<0.05	77.1 (76.8–77.4)		0.91 (0.88, 0.94)
Cohort								<0.05	
Internal validation cohort	4	0.85 (0.82, 0.88)	<0.05	64.3 (64.0–64.6)	0.87 (0.84, 0.90)	<0.05	76.4 (76.1–76.7)		0.88 (0.84, 0.92)
External validation cohort	2	0.87 (0.83, 0.90)	<0.05	73.3 (73.0–73.6)	0.89 (0.74, 0.99)	<0.05	69.4 (69.1–69.7)		0.90 (0.86, 0.94)
Feature-source								<0.05	
Imaging-only	4	0.87 (0.85, 0.89)	<0.05	69.4 (69.1–69.7)	0.92 (0.87, 0.97)	<0.05	61.6 (61.3–61.9)		0.89 (0.83, 0.95)
Imaging-clinical integrated	2	0.88 (0.68, 0.99)	<0.05	76.3 (76.0–76.6)	0.93 (0.90, 0.97)	<0.05	50.0 (49.7–50.3)		0.90 (0.80, 0.99)

^a^*P*-value for heterogeneity within each subgroup.

^b^*P*-value for heterogeneity between subgroups with meta-regression analysis.

*DMs* distant metastases, *LC* lung cancer, *ROI* region of interest, *ML* machine learning, *DL* deep learning, *CI* confidence interval, *SCLC* small cell lung cancer, *NSCLC* non-small cell lung cancer.

Table 4 summarizes the pooled performance of AI models in the imaging-based Outcome prediction task for distant metastases in LC. In the Local metastatic ROI subgroup, Intra-peritumoral models showed relatively higher pooled performance compared to Intratumoral models, with pooled SEN/SPE of 0.85/0.91 and 0.80/0.81, and AUCs of 0.91 (95% CI 0.88–0.93) and 0.87 (95% CI 0.84–0.90), respectively (Supplementary Figs. 20, 41, 62, 83). In the Cohort subgroup, internal and external validation cohorts showed comparable performance, with SEN/SPE of 0.80/0.81 and 0.85/0.91, and AUCs of 0.87 (95% CI 0.84–0.90) and 0.91 (95% CI 0.88–0.93), respectively, suggesting stable model generalization across validation settings (Supplementary Figs. 21, 42, 63, 84). In the Feature-source subgroup, Imaging-clinical integrated models again showed higher pooled performance than Imaging-only models, with SEN/SPE of 0.84/0.90 and 0.80/0.78, and AUCs of 0.92 (95% CI 0.88–0.95) and 0.85 (95% CI 0.78–0.93), respectively (Supplementary Figs. 22, 43, 64, 84). In the Prognostic type classification subgroup, Survival classification models demonstrated relatively higher pooled performance compared to Response classification, with pooled SEN/SPE of 0.88/0.86 and 0.78/0.86, and AUCs of 0.93 (95% CI 0.89–0.96) and 0.86 (95% CI 0.81–0.92), respectively (Supplementary Figs. 23, 44, 65, 86).

**Table 4:** Summary estimates of pooled performance for outcome prediction task of AI in DMs imaging in LC

Subgroup	No. of studies	Sensitivity			Specificity			*P*^b^	AUC (95% CI)
		Sensitivity	*P*^a^	*I*^*2*^ (95% CI)	Specificity	*P*^a^	*I*^*2*^ (95% CI)		
Overall (brain)	10	0.82 (0.75, 0.90)	<0.05	95.4 (94.8–96.0)	0.86 (0.81, 0.91)	<0.05	89.7 (89.1–90.3)		0.89 (0.85, 0.93)
Local metastatic ROI								<0.05	
Intratumoral	7	0.80 (0.73, 0.92)	<0.05	94.5 (93.9–95.1)	0.81 (0.75, 0.84)	<0.05	53.5 (53.0–54.0)		0.87 (0.84, 0.90)
Intra-peritumoral	3	0.85 (0.79, 0.89)	<0.05	64.0 (63.6–64.4)	0.91 (0.87, 0.94)	<0.05	78.0 (77.4–78.6)		0.91 (0.88, 0.93)
Cohort								<0.05	
Internal validation cohort	7	0.80 (0.73, 0.92)	<0.05	94.5 (93.9–95.1)	0.81 (0.75, 0.84)	<0.05	53.5 (53.0–54.0)		0.87 (0.84, 0.90)
External validation cohort	3	0.85 (0.79, 0.89)	<0.05	64.0 (63.6–64.4)	0.91 (0.87, 0.94)	<0.05	78.0 (77.4–78.6)		0.91 (0.88, 0.93)
Feature-source								<0.05	
Imaging-only	3	0.80 (0.71, 0.88)	<0.05	87.6 (87.1–88.1)	0.78 (0.70, 0.87)	<0.05	76.4 (75.8–77.0)		0.85 (0.78, 0.93)
Imaging-clinical integrated	7	0.84 (0.75, 0.92)	<0.05	92.3 (91.7–92.9)	0.90 (0.87, 0.94)	<0.05	64.2 (63.7–64.7)		0.92 (0.88, 0.95)
Prognostic type classification								<0.05	
Response classification	5	0.78 (0.72, 0.84)	<0.05	75.3 (74.8, 75.8)	0.86 (0.81, 0.91)	0.06	55.1 (54.7, 55.5)		0.86 (0.81, 0.92)
Survival classification	5	0.88 (0.81, 0.95)	<0.05	84.9 (84.6, 85.2)	0.86 (0.78, 0.94)	<0.05	91.9 (91.6, 92.2)		0.93 (0.89, 0.96)

*DMs* distant metastases, *LC* lung cancer, *ROI* region of interest, *CI* confidence interval.

^a^*P*-value for heterogeneity within each subgroup.

^b^*P*-value for heterogeneity between subgroups with meta-regression analysis.

### Heterogeneity analysis

The results of four meta-analyses of 59 studies suggested the potential utility of AI algorithms in both diagnostic and prognostic prediction based on imaging of DMs in LC. However, substantial heterogeneity was observed among the included studies (*I²* > 75%). The detailed results of subgroup and meta-regression analyses exploring the potential sources of between-study heterogeneity are presented in Tables 1–4. The results highlighted statistically significant differences among subgroups. Funnel plot analysis indicated no significant publication bias (*P* > 0.10) among the included studies (Supplementary Fig. 87).

## Discussion

This comprehensive meta-analysis provides a metastasis-focused synthesis showing that both ML and DL approaches achieved promising performance in the diagnostic assessment and outcome prediction of DMs in LC. The findings highlight the potential of AI to improve precision imaging and clinical decision-making in metastatic LC. Potential sources of between-study heterogeneity were systematically explored through subgroup analyses and meta-regression in each task-specific meta-analysis. Study quality and risk of bias were evaluated using the QUADAS-AI tool, which is specifically designed for AI-based diagnostic research. Four task-specific meta-analyses were conducted in this study according to distinct clinical tasks. Additionally, considering that the unique microenvironments of metastatic sites not only determine the metastatic tendency and growth characteristics of tumors but also lead to significant heterogeneity in their imaging manifestations^23^, we established Metastatic lesion subgroup within each task. This approach assessed the effect of metastatic site (e.g., brain, bone, and liver) on AI model accuracy and examined AI performance across multiple tasks, which facilitates understanding of AI model behavior under specific clinical conditions and provides more precise guidance for future investigations. To date, few meta-analyses have examined imaging of brain metastases in LC, with existing studies focusing exclusively on EGFR mutations^24,25^, whose pooled estimates for EGFR mutation prediction were comparable to our results. Our meta-analyses broaden the evidence base by evaluating multiple clinically relevant tasks and by extending analyses across metastatic sites.

The meta-analysis showed that the Metastasis differentiation task achieved slightly higher predictive performance than the other tasks, which may be explained by relatively stronger, biologically grounded imaging contrasts between metastatic lesions and common mimics within each target organ. In the brain, primary gliomas typically exhibit diffuse infiltrative growth along white matter tracts, producing infiltrative edema with indistinct boundaries and gradual signal transitions on imaging^26^, whereas brain metastases more often present as focal lesions with well-defined vasogenic edema related to blood-brain barrier disruption and inflammatory changes^27^. Similarly, bone metastases arise from tumor-driven remodeling of the bone-marrow microenvironment, characterized by coupled osteolysis/osteogenesis, immune infiltration, and cytokine network alterations^28^, which differs from normal bone homeostasis and from imaging patterns of primary bone tumors that often reflect localized neoplastic osteogenesis^29,30^ and angiogenesis^31^. These microenvironmental differences translate into distinct texture, intensity distribution, and spatial structural patterns on imaging^32,33^, providing discriminative cues that AI models can leverage to differentiate lesion types and, potentially, detect early remodeling-related changes.

Across both the Molecular-level prediction and Metastasis differentiation tasks, AI performance was higher for brain metastases than for bone metastases. Although performance for liver metastases appeared slightly lower than for the other two sites, this subgroup included only two studies^34,35^ and should therefore be interpreted as exploratory. The observed site-specific variation may reflect differences in imaging conspicuity and organ-specific anatomical and physiological contexts. Brain metastases often show marked contrast enhancement, prominent perilesional edema, and relatively well-defined margins, which provide stable and information-rich patterns for model learning^36^. In contrast, bone metastases are influenced by heterogeneous bone density, variable remodeling phenotypes, and imaging artifacts, which may reduce feature stability and reproducibility despite characteristic osteolytic/osteoblastic changes^37,38^. Evidence for liver metastases remains limited, and the intrinsic challenges of hepatic imaging, including complex hemodynamics and relatively low lesion-to-parenchyma contrast in some settings, may further hinder robust model development and validation^39^.

It was observed that the intra-peritumoral models (which combine peritumoral and intratumoral regions) tended to perform better than models that used only the intratumoral regions. This finding indicates that the tumor habitat is an important characteristic of metastatic lesions that influences AI model accuracy. AI models in imaging-based diagnosis and prognostication of DM lesions should not be limited to the tumor mass alone, but should also integrate features from the surrounding microenvironment^40^. Tumor and host tissue frequently exhibit complex edema responses, changes in vascular permeability, and immune cell infiltration, all of which provide essential biological signals that significantly contribute to model performance^41^. Especially for brain metastases, three distinct spatial regions comprising the tumor active area, the peritumoral edema area, and the necrotic area not only represent different pathological states but also reflect the diversity of tumor growth dynamics, microenvironmental adaptation, and signaling regulation^29^. The tumor active area typically exhibits features of high metabolic activity and angiogenesis, indicating rapid tumor cell proliferation and increased energy demand. The peritumoral edema area, located at the interface between the tumor and brain parenchyma, serves as a critical area of interaction between tumor cells, immune cells, and reactive glial components, thereby revealing the dynamic processes of microenvironmental remodeling and immune response^42^. The necrotic area, in contrast, reflects metabolic exhaustion and tissue degradation, resulting from prolonged hypoxia and inflammatory processes^43^. These regional variations are “encoded” into imaging manifestations, producing differences in intensity, texture, and spatial distribution that can be recognized and learned by AI models to predict gene mutation status^44^, histological subtypes of LC (Adeno/Squamous^45^ and SCLC/NSCLC^46^), and patient outcome prediction^47,48^.

Some of the included studies employed a combined analysis strategy for primary and DM lesions, integrating CT imaging of the primary lung tumor with MRI of DMs to enhance model predictive performance^30^. The advantage of this multimodal and multi-site imaging integration lies not only in the increased volume of information but also in its ability to comprehensively characterize the systemic biological behavior of the tumor. The imaging features of the primary tumor reflect the initial differentiation state, metabolic activity, and genetic mutation background of tumor cells, whereas metastatic lesions represent phenotypic changes following re-colonization, immune evasion, and vascular remodeling within heterogeneous microenvironments^28,31^. The joint analysis of imaging data from both primary and DM lesions enables AI models to capture both gene-driven signals and environment-adaptive features, thereby improving the accuracy of predictions regarding disease progression and treatment response.

Furthermore, the integration of imaging data and clinical information has been shown to improve predictive performance in both diagnostic and prognostic models. The inclusion of patients’ clinical variables such as age, sex, smoking history, tumor stage, molecular subtype, serum tumor markers, and treatment regimen, along with imaging features, into AI models can reduce noise and bias that may be introduced by relying solely on imaging data^32^. However, a systematic framework for the joint multimodal analysis of both primary and DM lesions in combination with clinical characteristics remains absent. Significant challenges remain in several critical areas, including image registration, feature selection, modality integration, and model interpretability^33^.

The subgroup analysis comparing ML and DL demonstrated notable differences, with DL models generally outperforming ML-based AI models across different tasks. ML models are highly dependent on the processes of feature extraction and selection, making their performance susceptible to human expertise and parameter tuning^49^. Moreover, ML is often limited when handling high-dimensional, nonlinear, or multimodal data, making it less effective at capturing complex and latent feature patterns^50^. In contrast, DL models have the capability to automatically learn high-dimensional, nonlinear imaging features, which enhances their predictive accuracy and feature representation, particularly in complex tasks such as multimodal fusion analysis or cross-organ metastasis identification^51^.

Although AI models have demonstrated considerable promise in the imaging of DMs in LC, these systems are far from perfect. It is essential to critically consider several methodological challenges for further improvement.

First, data still serves as the foundational element of the entire system, regardless of whether the approach is ML or DL. In our meta-analysis, most included studies were retrospective, and their sample sizes were generally limited, with the vast majority of studies including fewer than 400 patients, and only one study enrolling as many as 730 patients^52^. Fewer than half of the studies employed a multicenter design. It is well established that model performance tends to be more stable with larger training data^53^, suggesting that future efforts should prioritize multicenter, large-scale data collaboration to enhance performance consistency and generalizability of the proposed models. Moreover, significant heterogeneity in scanning protocols and equipment across centers, along with reliance on manual annotation of images and labels, may introduce subjective bias and noise into AI models^54^. These issues suggest that future research should not only focus on the innovation of algorithms themselves but also pay more attention to the construction and standardization of high-quality image cohorts, cross-center data collaboration, as well as the standardization of annotation processes and evaluation metrics, so as to effectively improve the reproducibility and transferability of AI in clinical implementation. Additionally, current studies have predominantly focused on brain metastases, while research on other metastatic sites, such as the bone and liver, remain limited. Notably, all prognostic studies focused exclusively on brain metastases. This bias limits a comprehensive understanding of AI performance across different metastatic organs, and the prognostic analyses included in this study were primarily based on binary endpoints as defined in the original studies; consequently, the pooled estimates should be interpreted as classification-based outcome prediction rather than a comprehensive synthesis of time-to-event prognostic modeling. Taken together, these limitations underscore substantial unmet clinical needs in this field.

Second, the “black-box” decision-making mechanism of ML and DL remains a crucial challenge restricting the clinical translation of AI models^55^. Existing studies have started to explore approaches to improve the interpretability of AI models. A common strategy involves the use of visualization techniques such as saliency maps and heatmaps to map the key regions identified by AI models onto recognizable radiological features in medical images^56^. For instance, in studies on brain metastases from LC, saliency maps can highlight tumor margins or regions associated with the blood-brain barrier preferentially attended to by the model^57^. Similarly, in MRI analyses of bone metastases, heatmaps can reveal areas of osteolysis or newly formed bone that are identified as salient by the model^58^. Although these methods provide preliminary support for model interpretability, their application remains limited, particularly in linking high-dimensional features to specific biological mechanisms within the tumor microenvironment, as a systematic theoretical framework for such correspondence is still lacking^18^. Moreover, most of these interpretability approaches are post-hoc analyses, meaning they are applied after model development to assist in interpreting results through visualization tools, rather than being integrated into the model design process itself. Future research should focus on incorporating interpretability constraints during model architecture design, guided by multimodal biological knowledge, to steer and constrain AI models in learning imaging features that are biologically relevant to the tumor microenvironment.

Third, in our analysis of the included studies, we observed a trend toward integrating clinically available variables with imaging features to improve model performance. Building on this direction, although publicly available multimodal and multi-omics databases for DMs of LC remain scarce and feature registration and integration are technically demanding^59^, the integration of multimodal imaging with multi-omics data is still regarded as a promising route to further improve both prediction accuracy and biological interpretability. Current AI models lack specific designs for learning microenvironmental features of DM lesions, making it difficult to reveal the correspondence between deep imaging representations and underlying biological mechanisms. Future efforts could focus on optimizing model architectures, particularly deep-learning networks, by integrating modules such as attention mechanisms and multi-scale feature fusion and further attempting to introduce multi-omics data that can capture information about cellular states, signaling pathways, and microenvironment composition, such as transcriptomics, proteomics, and single-cell sequencing. By jointly modeling different imaging modalities together with molecular-level information, AI models can perform cross-level correlation analyses that link “imaging phenotypes” to “biological mechanisms”^60^, thereby providing a biological foundation and interpretative framework for AI feature learning^61^. For example, in brain metastasis research, imaging features can be jointly modeled with transcriptomic data reflecting neuroglial responses, angiogenesis, or matrix remodeling^62^, thereby uncovering tumor invasion patterns and mechanisms of brain microenvironment remodeling. In bone metastases, MRI can be integrated with bone metabolism-related molecular markers (such as RANKL, OPG, and M-CSF) to characterize the dynamic balance between bone destruction and repair^63^. In liver metastases, fusion analysis combining CT imaging with features of the tumor immune microenvironment can reveal the interactions between the tumor vascular supply and immune heterogeneity^55^.

Furthermore, the TRL evaluation results in this study reveal that most current studies remain at the stage of algorithm development and validation and have not yet achieved testing or integrated application in real clinical environments. There is still a clear gap between research and practical application of these AI methods. Moreover, only 2 of the 64 eligible studies compared AI outputs with physician assessments. Implementing an end-to-end design from model development to clinical deployment and feedback mechanisms with the participation of physicians would be beneficial to realize the true clinical translation of AI tools in the management of distant lung cancer metastases.

This study has several limitations. First, the majority of the included studies were retrospective, which restricts the strength of the evidence. The absence of prospective studies further limits the assessment of AI model generalizability in real-world clinical settings. Second, the restriction to English-language publications may have introduced selection bias, potentially excluding important findings reported in other languages. Third, the studies involved patients with LC at various stages, leading to heterogeneity in patient characteristics and prognostic factors. Fourth, due to the limited number of AI studies using time-to-event survival modeling, we were unable to adequately synthesize survival regression performance and thus cannot fully reflect the overall trend in this area. Fifth, many of the included studies were conducted in Asian populations, which may limit the global generalizability of the reported AI performance. Furthermore, this meta-analysis has inherent limitations. Meta-analyses are highly dependent on published studies, which may be biased toward positive results, and thus may introduce publication bias and limit a comprehensive assessment of AI model performance consistency.

Overall, this study represents a comprehensive evaluation of existing literature on image-based AI algorithms for DM in LC. Current evidence suggests that AI may add value beyond routine radiologist interpretation at specific clinical decision points, including pretreatment diagnostic assessment and personalized treatment decision-making, by offering a noninvasive and quantitative characterization of metastatic heterogeneity and the tumor microenvironment. Nevertheless, existing studies remain limited by inadequate quality and the early stages of AI technology.

## Methods

### Protocol registration and study design

This systematic review was prospectively registered in PROSPERO (Registration Number: CRD420251056539). The study adhered to the guidelines outlined in the PRISMA (Preferred Reporting Items for Systematic Reviews and Meta-Analyses) statement^64^ and the MOOSE (Meta-analyses of Observational Studies in Epidemiology) guidelines^65^.

### Search strategy and eligibility criteria

A comprehensive literature search was conducted across four electronic databases: PubMed, EMBASE, Cochrane Library, and Web of Science, from their inception to April 18, 2025. The search strategy combined terms related to artificial intelligence, lung cancer, and imaging of distant metastases (e.g., “artificial intelligence” OR “deep learning” OR “radiomics” AND “lung cancer” AND (“metastatic lesions” OR “brain metastases” OR “bone metastases” OR “liver metastases”)). Only studies published in English were included. Preliminary screening included studies that applied AI algorithms to imaging data of DM lesions in LC for diagnostic or prognostic prediction. The full search strategy is provided in Supplementary Note 1.

Eligible studies were required to report model performance metrics such as sensitivity (SEN), specificity (SPE), or area under the curve (AUC), or to provide sufficient data for constructing a 2×2 contingency table. Among the prognostic studies included, the majority focused on predicting the efficacy of targeted therapies. These studies, however, did not utilize regression-based prognostic models. Instead, they employed a binary classification approach, categorizing patients into two groups, such as responsive and non-responsive groups. To quantitatively assess and demonstrate the Outcome prediction performance of these models, we constructed contingency tables based on data reported by authors in the original studies. The following types of studies were excluded from the meta-analysis: 1) Studies lacking key information or with insufficient data to construct a 2×2 contingency table or prognostic studies that did not report available data, 2) Studies reporting performance metrics only on the training datasets, 3) Experimental studies involving animals or basic research, and 4) Editorials, letters, commentaries, reviews, or case reports. The inclusion and exclusion criteria were jointly developed by three independent investigators (B.W., C.Y. and Y.F.). Initial screening of titles and abstracts was performed by two trained reviewers (L.J. and B.W.), and subsequently cross-verified by another two reviewers (J.S. and Y.S.). Any disagreements were resolved through group discussion until a consensus was reached.

### Data extraction

Two reviewers (L.J. and B.W.) independently extracted data from each eligible study, followed by independent verification by another two reviewers (J.S. and Y.S.). Extracted data were cross-checked for consistency, and discrepancies were resolved through discussion or consultation with a fifth investigator (X.J.). Diagnostic accuracy data, including true positives, false positives, true negatives, and false negatives, were directly extracted to construct 2×2 contingency tables for the calculation of SEN and SPE. If multiple models were presented in a single study, we extracted both the best-performing model and the corresponding comparator models, and conducted separate analyses for each^66^. We assessed the Technology Readiness Level (TRL) of the included studies with an emphasis on data utilization and clinical translation^67^, and the detailed operational criteria and quantification table are provided in Supplementary Note 2.

### Quality assessment

The methodological quality and risk of bias of the included studies were assessed using the QUADAS-AI (Quality Assessment of Diagnostic Accuracy Studies-Artificial Intelligence) tool^68^. Two reviewers (L.J. and B.W.) independently performed the assessment, which was then cross-checked by another two reviewers (J.S. and Y.S). Any disagreements were resolved through discussion with a fifth investigator (X.J.). The QUADAS-AI tool evaluates four key domains: patient selection, index test, reference standard, and flow and timing. Risk of bias was classified as “low”, “high”, or “unclear” for each domain. The details of the QUADAS-AI assessments are presented in Supplementary Note 3. Potential cohort overlap was additionally assessed, with details provided in Supplementary Note 4.

### Meta-analysis

This study encompasses the following four tasks in the investigation of image-based AI models in DMs of LC: Molecular-level prediction, Metastasis differentiation, Tumor type classification, Outcome prediction. For each task, the hierarchical summary receiver operating characteristic (SROC) curve was constructed to systematically evaluate the performance of AI-based models. This combined curve was plotted with its corresponding 95% confidence region and 95% prediction region around the averaged SE, SP, and AUC estimates in the SROC figures. The overall performance based on the best-performing models from each included study was plotted separately, along with the performance of all models, including those tested on validation datasets and in comparative experiments. Between-study heterogeneity was assessed using Cochran’s *Q* test and *I*² statistic, where significant heterogeneity was defined as *P* ≤ 0.10 or *I²* > 50%. To identify potential sources of heterogeneity, subgroup analyses and meta-regression were performed based on best-performing models. A random-effects model was applied to account for assumed variability across studies. Forest plots were generated for subgroup analyses to facilitate performance comparisons. Additionally, SROC curves were used to illustrate the distribution trends of sensitivity and specificity for all models within each subgroup. Funnel plots and regression tests were used to evaluate potential publication bias.

In each task, subgroup were conducted analyses generally based on the following study characteristics: 1) Type of metastatic lesion (Brain, Bone, or Liver), 2) Type of AI algorithm (ML vs. DL), 3) Use of internal versus external validation datasets, 4) Feature-source (Imaging-only vs. Combined imaging and clinical data), 5) Lesion-level Region of Interest (ROI) (Primary-metastatic combined vs. Metastatic-only), and 6) Local metastatic ROI (Intratumoral vs. Intra-peritumoral). Additionally, in Molecular-level task, which focused on predicting key gene mutations or immune marker expression (e.g., EGFR, T790, PD-L1), we added the Molecular subtype classification subgroup. In Tumor type classification task, which targeted on histological subtype classification, we added the Histologic subtype classification subgroup. In Outcome prediction task, we have added a Prognostic type of classification subgroup, covering response classification and survival classification.

### Statistical analysis

The methodological quality of the included studies was assessed using QUADAS-AI with RevMan (version 5.4). Metric comparisons were performed using the RevMan online calculator (version 5.4). All other statistical analyses were performed using Stata software (version 18.0), with a two-tailed type I error probability of 0.05 (*α* = 0.05).

## Supplementary information

Supplementary Materials

## Data Availability

The datasets generated and analyzed during the current study consist of study level data extracted and curated from previously published articles and are available from the corresponding author on reasonable request. Details of the search strategy, contingency tables, and risk of bias assessments are provided in Supplementary Notes 1–4, Supplementary Tables 5, 6, and Supplementary Fig. 2, respectively. All source studies included in this systematic review and meta-analysis can be identified through the reference list and Supplementary Information.

## Code availability

No custom code was used in this study. All analyses were performed using standard statistical software as described in the Methods section.
