# Supplementary material for: Systematic review and meta-analysis of AI in lung cancer metastasis imaging for diagnosis and prognosis
Source: NPJ Digit Med. 2026 Jun 5;9:688. doi: 10.1038/s41746-026-02858-1 (PMC13558739; doi:10.1038/s41746-026-02858-1)
Supplement: Supplementary file 1 [file 41746_2026_2858_MOESM1_ESM.pdf]

Supplementary Materials

Supplementary Notes

Supplementary Note 1 - Search Strategy

Supplementary Note 2 - Definition and Classification Criteria of Technology Readiness Levels (TRLs) for AI in Medical Imaging

Supplementary Note 3 - Description of quality assessment based on QUADAS-AI domains used to evaluate methodological quality

Supplementary Note 4 - Assessment of Potential Cohort Overlap

Supplementary Tables

Supplementary Table 1. Participant demographics for diagnosis-related studies (n = 52)

Supplementary Table 2. Participant demographics for Outcome prediction studies (n = 13)

Supplementary Table 3. Validation methods and algorithms for diagnosis-related studies (Molecular-level prediction, Metastasis differentiation, and Tumor type classification; n = 52).

Supplementary Table 4. Validation methods and algorithms for Outcome prediction studies (n = 13)

Supplementary Table 5. Contingency tables for AI diagnostic algorithms extracted from included studies

Supplementary Table 6. Contingency tables for AI prognostic algorithms extracted from included studies

Supplementary Figures

Supplementary Figure 1. QUADAS-AI summary plot

Supplementary Figure 2. Risk of bias and concern of applicability for each item in included studies

Supplementary Figure 3. Forest plot of pooled Molecular-level prediction performance (sensitivity) stratified by metastatic lesion subgroup.

Supplementary Figure 4. Forest plot of pooled Molecular-level prediction performance (sensitivity) stratified by molecular subtype classification subgroup.

Supplementary Figure 5. Forest plot of pooled Molecular-level prediction performance (sensitivity) stratified by lesion-level ROI subgroup.

Supplementary Figure 6. Forest plot of pooled Molecular-level prediction performance (sensitivity) stratified by local metastatic ROI subgroup.

Supplementary Figure 7. Forest plot of pooled Molecular-level prediction performance (sensitivity) stratified by algorithm type (ML vs. DL).

Supplementary Figure 8. Forest plot of pooled Molecular-level prediction performance (sensitivity) stratified by cohort type (internal vs. external).

Supplementary Figure 9. Forest plot of pooled Molecular-level prediction performance (sensitivity) stratified by feature-source subgroup.

Supplementary Figure 10. Forest plot of pooled Metastasis differentiation performance (sensitivity) stratified by metastatic lesion subgroup.

Supplementary Figure 11. Forest plot of pooled Metastasis differentiation performance (sensitivity) stratified by lesion-level ROI subgroup.

Supplementary Figure 12. Forest plot of pooled Metastasis differentiation performance (sensitivity) stratified by local metastatic ROI subgroup.
Supplementary Figure 13. Forest plot of pooled Metastasis differentiation performance (sensitivity) stratified by algorithm type (ML vs. DL).
Supplementary Figure 14. Forest plot of pooled Metastasis differentiation performance (sensitivity) stratified by cohort type (internal vs. external).
Supplementary Figure 15. Forest plot of pooled Metastasis differentiation performance (sensitivity) stratified by feature-source subgroup.
Supplementary Figure 16. Forest plot of pooled Tumor type classification performance (sensitivity) stratified by histologic subtype classification.
Supplementary Figure 17. Forest plot of pooled Tumor type classification performance (sensitivity) stratified by algorithm type (ML vs. DL).
Supplementary Figure 18. Forest plot of pooled Tumor type classification performance (sensitivity) stratified by cohort type (internal vs. external).
Supplementary Figure 19. Forest plot of pooled Tumor type classification performance (sensitivity) stratified by feature-source subgroup.
Supplementary Figure 20. Forest plot of pooled Outcome prediction (sensitivity) stratified by local metastatic ROI subgroup.
Supplementary Figure 21. Forest plot of pooled Outcome prediction (sensitivity) stratified by cohort type (internal vs. external).
Supplementary Figure 22. Forest plot of pooled Outcome prediction (sensitivity) stratified by feature-source subgroup.
Supplementary Figure 23. Forest plot of pooled Outcome prediction (sensitivity) stratified by prognostic type classification.
Supplementary Figure 24. Forest plot of pooled Molecular-level prediction performance (specificity) stratified by metastatic lesion subgroup.
Supplementary Figure 25. Forest plot of pooled Molecular-level prediction performance (specificity) stratified by molecular subtype classification subgroup.
Supplementary Figure 26. Forest plot of pooled Molecular-level prediction performance (specificity) stratified by lesion-level ROI subgroup.
Supplementary Figure 27. Forest plot of pooled Molecular-level prediction performance (specificity) stratified by local metastatic ROI subgroup.
Supplementary Figure 28. Forest plot of pooled Molecular-level prediction performance (specificity) stratified by algorithm type (ML vs. DL).
Supplementary Figure 29. Forest plot of pooled Molecular-level prediction performance (specificity) stratified by cohort type (internal vs. external).
Supplementary Figure 30. Forest plot of pooled Molecular-level prediction performance (specificity) stratified by feature-source subgroup.
Supplementary Figure 31. Forest plot of pooled Metastasis differentiation performance (specificity) stratified by metastatic lesion subgroup.
Supplementary Figure 32. Forest plot of pooled Metastasis differentiation performance (specificity) stratified by lesion-level ROI subgroup.
Supplementary Figure 33. Forest plot of pooled Metastasis differentiation performance (specificity) stratified by local metastatic ROI subgroup.

Supplementary Figure 34. Forest plot of pooled Metastasis differentiation performance (specificity) stratified by algorithm type (ML vs. DL).
Supplementary Figure 35. Forest plot of pooled Metastasis differentiation performance (specificity) stratified by cohort type (internal vs. external).
Supplementary Figure 36. Forest plot of pooled Metastasis differentiation performance (specificity) stratified by feature-source subgroup.
Supplementary Figure 37. Forest plot of pooled Tumor type classification performance (specificity) stratified by histologic subtype classification.
Supplementary Figure 38. Forest plot of pooled Tumor type classification performance (specificity) stratified by algorithm type (ML vs. DL).
Supplementary Figure 39. Forest plot of pooled Tumor type classification performance (specificity) stratified by cohort type (internal vs. external).
Supplementary Figure 40. Forest plot of pooled Tumor type classification performance (specificity) stratified by feature-source subgroup.
Supplementary Figure 41. Forest plot of pooled Outcome prediction (specificity) stratified by local metastatic ROI subgroup.
Supplementary Figure 42. Forest plot of pooled Outcome prediction (specificity) stratified by cohort type (internal vs. external).
Supplementary Figure 43. Forest plot of pooled Outcome prediction (specificity) stratified by feature-source subgroup.
Supplementary Figure 44. Forest plot of pooled Outcome prediction (specificity) stratified by prognostic type classification.
Supplementary Figure 45. Forest plot of pooled Molecular-level prediction performance (AUC) stratified by metastatic lesion subgroup.
Supplementary Figure 46. Forest plot of pooled Molecular-level prediction performance (AUC) stratified by molecular subtype classification subgroup.
Supplementary Figure 47. Forest plot of pooled Molecular-level prediction performance (AUC) stratified by lesion-level ROI subgroup.
Supplementary Figure 48. Forest plot of pooled Molecular-level prediction performance (AUC) stratified by local metastatic ROI subgroup.
Supplementary Figure 49. Forest plot of pooled Molecular-level prediction performance (AUC) stratified by algorithm type (ML vs. DL).
Supplementary Figure 50. Forest plot of pooled Molecular-level prediction performance (AUC) stratified by cohort type (internal vs. external).
Supplementary Figure 51. Forest plot of pooled Molecular-level prediction performance (AUC) stratified by feature-source subgroup.
Supplementary Figure 52. Forest plot of pooled Metastasis differentiation performance (AUC) stratified by metastatic lesion subgroup.
Supplementary Figure 53. Forest plot of pooled Metastasis differentiation performance (AUC) stratified by lesion-level ROI subgroup.
Supplementary Figure 54. Forest plot of pooled Metastasis differentiation performance (AUC) stratified by local metastatic ROI subgroup.
Supplementary Figure 55. Forest plot of pooled Metastasis differentiation performance (AUC) stratified by algorithm type (ML vs. DL).

Supplementary Figure 56. Forest plot of pooled Metastasis differentiation performance (AUC) stratified by cohort type (internal vs. external).
Supplementary Figure 57. Forest plot of pooled Metastasis differentiation performance (AUC) stratified by feature-source subgroup.
Supplementary Figure 58. Forest plot of pooled Tumor type classification performance (AUC) stratified by histologic subtype classification.
Supplementary Figure 59. Forest plot of pooled Tumor type classification performance (AUC) stratified by algorithm type (ML vs. DL).
Supplementary Figure 60. Forest plot of pooled Tumor type classification performance (AUC) stratified by cohort type (internal vs external).
Supplementary Figure 61. Forest plot of pooled Tumor type classification performance (AUC) stratified by feature-source subgroup.
Supplementary Figure 62. Forest plot of pooled Outcome prediction (AUC) stratified by local metastatic ROI subgroup.
Supplementary Figure 63. Forest plot of pooled Outcome prediction (AUC) stratified by cohort type (internal vs. external).
Supplementary Figure 64. Forest plot of pooled Outcome prediction (AUC) stratified by feature-source subgroup.
Supplementary Figure 65. Forest plot of pooled Outcome prediction (AUC) stratified by prognostic type classification.
Supplementary Figure 66. SROC curves stratified by metastatic lesion subgroup in Molecular-level prediction studies.
Supplementary Figure 67. SROC curves stratified by molecular subtype classification subgroup in Molecular-level prediction studies.
Supplementary Figure 68. SROC curves stratified by lesion-level ROI subgroup in Molecular-level prediction studies.
Supplementary Figure 69. SROC curves stratified by local metastatic ROI subgroup in Molecular-level prediction studies.
Supplementary Figure 70. SROC curves stratified by algorithm type (ML vs. DL) in Molecular-level prediction studies.
Supplementary Figure 71. SROC curves stratified by cohort type (internal vs. external) in Molecular-level prediction studies.
Supplementary Figure 72. SROC curves stratified by feature-source subgroup in Molecular-level prediction studies.
Supplementary Figure 73. SROC curves stratified by metastatic lesion subgroup in Metastasis differentiation studies.
Supplementary Figure 74. SROC curves stratified by lesion-level ROI subgroup in Metastasis differentiation studies.
Supplementary Figure 75. SROC curves stratified by local metastatic ROI subgroup in Metastasis differentiation studies.
Supplementary Figure 76. SROC curves stratified by algorithm type (ML vs. DL) in Metastasis differentiation studies.
Supplementary Figure 77. SROC curves stratified by cohort type (internal vs. external) in Metastasis differentiation studies.

Supplementary Figure 78. SROC curves stratified by feature-source subgroup in Metastasis differentiation studies.
Supplementary Figure 79. SROC curves stratified by histologic subtype classification subgroup in Tumor type classification studies.
Supplementary Figure 80. SROC curves stratified by algorithm type (ML vs. DL) in Tumor type classification studies.
Supplementary Figure 81. SROC curves stratified by cohort type (internal vs. external) in Tumor type classification studies.
Supplementary Figure 82. SROC curves stratified by feature-source subgroup in Tumor type classification studies.
Supplementary Figure 83. SROC curves stratified by local metastatic ROI subgroup in Outcome prediction studies.
Supplementary Figure 84. SROC curves stratified by cohort type (internal vs. external) in Outcome prediction studies.
Supplementary Figure 85. SROC curves stratified by feature-source subgroup in Outcome prediction studies.
Supplementary Figure 86. SROC curves stratified by prognostic type classification subgroup in Outcome prediction studies.
Supplementary Figure 87. Funnel plots for publication bias

Supplementary Note 1 - Search Strategy

Table E1.a. Search Strategies (PubMed)

Number	Search Terms	Search Hits
#1	"lung cancer"[Mesh] OR "lung neoplasms"[Mesh] OR "lung cancer*"[tiab] OR "lung neoplasm*"[tiab] OR "lung tumor*"[tiab] OR "pulmonary carcinoma"[tiab] OR "pulmonary neoplasm*"[tiab] OR "pulmonary cancer*"[tiab] OR "non-small cell lung cancer"[tiab] OR "NSCLC"[tiab] OR "small cell lung cancer"[tiab] OR "SCLC"[tiab]	377,812
#2	"EGFR"[tiab] OR "epidermal growth factor receptor"[tiab] OR "T790M"[tiab] OR "EGFR mutation*"[tiab] OR "EGFR-TKI"[tiab] OR "exon 19"[tiab] OR "exon 21"[tiab] OR "EGFR exon 19 deletion"[tiab] OR "EGFR exon 21 L858R"[tiab] OR "EGFR-sensitive mutation*"[tiab] OR "EGFR resistance mutation*"[tiab] OR "osimertinib"[tiab] OR "EGFR-TKI resistance"[tiab]	118,836
#3	#1 OR #2	470,531
#4	metastas*[tiab] OR "distant metastasis"[tiab] OR "distant metastases"[tiab] OR "metastatic spread"[tiab] OR "tumor spread"[tiab] OR "brain metastasis"[tiab] OR "brain metastases"[tiab] OR "cerebral metastasis"[tiab] OR "intracranial metastasis"[tiab] OR "liver metastasis"[tiab] OR "hepatic metastasis"[tiab] OR "bone metastasis"[tiab] OR "osseous metastasis"[tiab] OR "skeletal metastasis"[tiab] OR “spinal metastases”[tiab]	498,961
#5	"Artificial Intelligence"[mh]	229,415
#6	computational intelligence[tiab] OR machine intelligence[tiab] OR computer reasoning[tiab] OR computer vision[tiab]	12,137
#7	random forest*[tiab] OR decision tree*[tiab] OR naive bayes*[tiab] OR support vector machine*[tiab] OR support vector network*[tiab] OR natural language process*[tiab] OR NLP[tiab]	86,973
#8	"artificial intelligence"[tiab] OR "machine learning"[tiab] OR "deep learning"[tiab] OR "supervised learning"[tiab] OR "unsupervised learning"[tiab] OR "reinforcement learning"[tiab] OR "neural network*"[tiab] OR "convolution*"[tiab] OR "residual network*"[tiab] OR "transformer*"[tiab] OR "attention mechanism*"[tiab] OR "self-attention"[tiab] OR	358,698

	"generative adversarial network*"[tiab] OR "GAN"[tiab] OR "graph neural network*"[tiab] OR "GNN"[tiab] OR "vision transformer*"[tiab]	
#9	"radiomics"[tiab] OR "radiogenomics"[tiab] OR "radiomics feature*"[tiab] OR "image biomarker*"[tiab] OR "imaging signature*"[tiab] OR "radiological signature*"[tiab] OR "texture analysis"[tiab] OR "deep feature*"[tiab] OR "handcrafted feature*"[tiab] OR "nomogram"[tiab] OR "predictive model*"[tiab] OR "risk signature*"[tiab] OR "image-based classifier*"[tiab] OR "decision support model*"[tiab] OR "quantitative imaging biomarker*"[tiab] OR "computed signature*"[tiab]	86,545
#10	#5 OR #6 OR #7 OR #8 OR #9	545,477
#11	predict*[tiab] OR prognos*[tiab] OR prospective[tiab]	3,601,455
#12	risk[tiab] AND (estimat*[tiab] OR score*[tiab] OR calculat*[tiab] OR index*[tiab] OR assess*[tiab] OR stratif*[tiab] OR validat*[tiab])	1,569,971
#13	"c statistic"[tiab] OR discriminat*[tiab] OR AUC[tiab] OR "area under the curve"[tiab] OR "area under the receiver operator curve"[tiab] OR "risk ratio"[tiab] OR "odds ratio"[tiab] OR "hazard ratio"[tiab]	999,768
#14	"magnetic resonance imaging"[tiab] OR "MRI"[tiab] OR "T1-weighted"[tiab] OR "T1WI"[tiab] OR "T2-weighted"[tiab] OR "T2WI"[tiab] OR "diffusion-weighted imaging"[tiab] OR "DWI"[tiab] OR "ADC map"[tiab] OR "contrast-enhanced MRI"[tiab] OR "functional MRI"[tiab] OR "fMRI"[tiab] OR "computed tomography"[tiab] OR "CT"[tiab] OR "contrast-enhanced CT"[tiab] OR "CE-CT"[tiab] OR "chest CT"[tiab] OR "positron emission tomography"[tiab] OR "PET"[tiab] OR "PET/CT"[tiab] OR "PET-CT"[tiab] OR "18F-FDG"[tiab] OR "FDG-PET"[tiab] OR "ultrasound"[tiab] OR "ultrasonography"[tiab] OR "US"[tiab] OR "Doppler ultrasound"[tiab] OR "contrast-enhanced ultrasound"[tiab] OR "CEUS"[tiab] OR "medical imaging"[tiab] OR "radiological imaging"[tiab] OR "diagnostic imaging"[tiab]	2,175,651
#15	#11 OR #12 OR #13 OR #14	6,769,126
#16	#3 AND #4 AND #10 AND #15	1,352

Table E1.a. Search Strategies (Web of Science)

**Number**	**Search Terms**	**Search Hits**
#1	TS=("lung cancer*" OR "lung neoplasm*" OR "lung tumor*" OR "pulmonary carcinoma" OR "pulmonary neoplasm*" OR "pulmonary cancer*" OR "non-small cell lung cancer" OR "NSCLC" OR "small cell lung cancer" OR "SCLC")	382,761
#2	TS=("EGFR" OR "epidermal growth factor receptor" OR "T790M" OR "EGFR mutation*" OR "EGFR-TKI" OR "exon 19" OR "exon 21" OR "EGFR exon 19 deletion" OR "EGFR exon 21 L858R" OR "EGFR-sensitive mutation*" OR "EGFR resistance mutation*" OR "osimertinib" OR "EGFR-TKI resistance")	133,651
#3	#1 OR #2	480,683
#4	TS=(metastas* OR "distant metastasis" OR "distant metastases" OR "metastatic spread" OR "tumor spread" OR "brain metastasis" OR "brain metastases" OR "cerebral metastasis" OR "intracranial metastasis" OR "liver metastasis" OR "hepatic metastasis" OR "bone metastasis" OR "osseous metastasis" OR "skeletal metastasis" OR “spinal metastases”[tiab])	532,968
#5	TS=("computational intelligence" OR "machine intelligence" OR "computer reasoning" OR "computer vision")	93,775
#6	TS=("random forest*" OR "decision tree*" OR "naive bayes*" OR "support vector machine*" OR "support vector network*" OR "natural language process*" OR "NLP")	307,520
#7	TS=("artificial intelligence" OR "machine learning" OR "deep learning" OR "supervised learning" OR "unsupervised learning" OR "reinforcement learning" OR "neural network*" OR "convolution*" OR "residual network*" OR "transformer*" OR "attention mechanism*" OR "self-attention" OR "generative adversarial network*" OR "GAN" OR "graph neural network*" OR "GNN" OR "vision transformer*")	1,685,317
#8	TS=("radiomics" OR "radiogenomics" OR "radiomics feature*" OR "image biomarker*" OR "imaging signature*" OR	164,919

	"radiological signature*" OR "texture analysis" OR "deep feature*" OR "handcrafted feature*" OR "nomogram" OR "predictive model*" OR "risk signature*" OR "image-based classifier*" OR "decision support model*" OR "quantitative imaging biomarker*" OR "computed signature*")	
#9	#5 OR #6 OR #7 OR #8	1,997,479
#10	TS=(predict* OR prognos* OR prospective)	6,218,037
#11	TS=(risk AND (estimat* OR score* OR calculat* OR index* OR assess* OR stratifi* OR validat*))	2,139,294
#12	TS=("c statistic" OR discriminat* OR AUC OR "area under the curve" OR "area under the receiver operator curve" OR "risk ratio" OR "odds ratio" OR "hazard ratio")	1,236,193
#13	TS=("magnetic resonance imaging" OR MRI OR "T1-weighted" OR T1WI OR "T2-weighted" OR T2WI OR "diffusion-weighted imaging" OR DWI OR "ADC map" OR "contrast-enhanced MRI" OR "functional MRI" OR fMRI OR "computed tomography" OR CT OR "contrast-enhanced CT" OR CE-CT OR "chest CT" OR "positron emission tomography" OR PET OR "PET/CT" OR "PET-CT" OR "18F-FDG" OR "FDG-PET" OR "ultrasound" OR "ultrasonography" OR US OR "Doppler ultrasound" OR "contrast-enhanced ultrasound" OR CEUS OR "medical imaging" OR "radiological imaging" OR "diagnostic imaging")	3,208,946
#14	#10 OR #11 OR #12 OR #13	10,839,595
#15	#3 AND #4 AND #9 AND #14	1,485

Table E1.a. Search Strategies (Embase)

Number	Search Terms	Search Hits
#1	('lung cancer'/exp OR 'lung carcinoma'/exp OR 'pulmonary carcinoma'/exp OR 'pulmonary neoplasm*':ti,ab,kw OR 'non-small cell lung cancer':ti,ab,kw OR 'NSCLC':ti,ab,kw OR 'small cell lung cancer':ti,ab,kw OR 'SCLC':ti,ab,kw)	588,776
#2	('epidermal growth factor receptor'/exp OR EGFR:ti,ab,kw OR 'EGFR mutation*':ti,ab,kw OR 'EGFR-TKI':ti,ab,kw OR 'T790M':ti,ab,kw OR 'L858R':ti,ab,kw OR 'EGFR exon 19':ti,ab,kw OR 'EGFR exon 21':ti,ab,kw OR 'EGFR resistance mutation*':ti,ab,kw)	232,214
#3	#1 OR #2	760,379
#4	('metastasis'/exp OR 'distant metastasis':ti,ab,kw OR 'metastatic spread':ti,ab,kw OR 'brain metastasis'/exp OR 'cerebral metastasis':ti,ab,kw OR 'intracranial metastasis':ti,ab,kw OR 'hepatic metastasis':ti,ab,kw OR 'liver metastasis':ti,ab,kw OR 'bone metastasis':ti,ab,kw OR 'skeletal metastasis':ti,ab,kw OR 'spinal metastasis':ti,ab,kw)	984,181
#5	('computational intelligence'/exp OR 'machine intelligence':ti,ab,kw OR 'computer vision'/exp OR 'computer reasoning':ti,ab,kw)	7,458
#6	('random forest':ti,ab,kw OR 'decision tree':ti,ab,kw OR 'naive bayes':ti,ab,kw OR 'support vector machine':ti,ab,kw OR 'SVM':ti,ab,kw OR 'neural network*':ti,ab,kw OR 'NLP':ti,ab,kw OR 'natural language processing':ti,ab,kw)	265,377
#7	'artificial intelligence'/exp OR 'machine learning'/exp OR 'deep learning'/exp OR 'supervised learning':ti,ab,kw OR 'unsupervised learning':ti,ab,kw OR 'neural network*':ti,ab,kw OR 'convolutional neural network':ti,ab,kw OR 'residual network':ti,ab,kw OR resnet:ti,ab,kw OR 'transformer':ti,ab,kw OR 'attention mechanism':ti,ab,kw OR 'self-attention':ti,ab,kw OR 'gan':ti,ab,kw OR 'graph neural network':ti,ab,kw OR gnn:ti,ab,kw OR 'vision transformer':ti,ab,kw	784,361
#8	'radiomics'/exp OR 'radiogenomics'/exp OR 'image biomarker*':ti,ab,kw OR 'imaging biomarker*':ti,ab,kw OR 'radiological signature':ti,ab,kw OR 'texture analysis':ti,ab,kw OR 'deep feature':ti,ab,kw OR 'handcrafted feature':ti,ab,kw OR 'nomogram':ti,ab,kw OR 'predictive model':ti,ab,kw OR 'risk signature':ti,ab,kw OR 'image-based classifier':ti,ab,kw OR 'decision support model':ti,ab,kw OR 'quantitative imaging biomarker':ti,ab,kw OR 'computed signature':ti,ab,kw	104,522
#9	#5 OR #6 OR #7 OR #8	889,560
#10	'predict' OR 'prediction':ti,ab,kw OR 'prognosis'/exp OR 'prognostic':ti,ab,kw OR 'forecast':ti,ab,kw OR 'outcome	2,461,577

	prediction':ti,ab,kw	
#11	'risk factor'/exp OR 'risk score':ti,ab,kw OR 'score system':ti,ab,kw OR 'auc':ti,ab,kw OR 'area under curve':ti,ab,kw OR 'roc curve':ti,ab,kw OR 'sensitivity':ti,ab,kw OR 'specificity':ti,ab,kw OR 'risk ratio':ti,ab,kw OR 'odds ratio':ti,ab,kw OR 'hazard ratio':ti,ab,kw OR 'validate':ti,ab,kw OR 'validation':ti,ab,kw	4,751,884
#12	'magnetic resonance imaging'/exp OR 'mri':ti,ab,kw OR 'computed tomography'/exp OR 'ct scan':ti,ab,kw OR 'positron emission tomography'/exp OR 'pet scan':ti,ab,kw OR 'ultrasound imaging'/exp OR 'diffusion weighted imaging':ti,ab,kw OR 'functional mri':ti,ab,kw OR 'contrast-enhanced mri':ti,ab,kw OR 'dwi':ti,ab,kw OR 'pet/ct':ti,ab,kw OR 'radiological imaging':ti,ab,kw	2,790,320
#13	#10 OR #11 OR #12	7,004,610
#14	#3 AND #4 AND #9 AND #14	713

Table E1.a. Search Strategies (Cochrane library)

Number	Search Terms	Search Hits
#1	"lung cancer" OR "lung carcinoma" OR "pulmonary carcinoma" OR "non small cell lung cancer" OR NSCLC OR "small cell lung cancer" OR SCLC	29,175
#2	EGFR OR "EGFR mutation" OR "EGFR-TKI" OR T790M OR L858R OR "EGFR exon 19" OR "EGFR exon 21" OR "EGFR resistance mutation"	15,475
#3	"metastasis" OR "brain metastasis" OR "bone metastasis" OR "skeletal metastasis" OR "spinal metastasis" OR "liver metastasis"	28,951
#4	"artificial intelligence" OR "machine learning" OR "deep learning" OR "neural network" OR "convolutional neural network" OR CNN OR "graph neural network" OR GNN OR "transformer" OR "attention mechanism"	8,431
#5	"radiomics" OR "radiogenomics" OR "image biomarker" OR "texture analysis" OR "nomogram" OR "predictive model" OR "risk signature" OR "quantitative imaging"	3,765
#6	"predict" OR "prediction" OR "prognostic" OR "outcome" OR "forecast"	838,342
#7	"risk score" OR "AUC" OR "ROC curve" OR "hazard ratio" OR "validation" OR "performance evaluation"	80,614
#8	"MRI" OR "CT" OR "PET" OR "ultrasound" OR "DWI" OR "functional MRI" OR "PET/CT"	444,069
#9	(#1 OR #2) AND #3 AND (#4 OR #5) AND (#6 OR #7) AND #8	27

Supplementary Note 2 - Definition and Classification Criteria of Technology Readiness Levels (TRLs) for AI in Medical Imaging

TRL Level	Description	Example in Medical Imaging AI
**TRL 1**	Basic principles observed and reported	Basic research in AI for image segmentation or classification; development of theoretical algorithms.
**TRL 2**	Technology concept formulated	Conceptualization of AI models for medical image analysis; early theoretical frameworks or proof of concept for tumor detection.
**TRL 3**	Proof of concept through analytical	AI models tested in controlled laboratory environments on medical image datasets.

	and experimental work	
**TRL 4**	Component validation in a laboratory environment	AI model for specific task tested and validated on small-scale datasets in the lab.
**TRL 5**	Component validation in a relevant environment	Prototype AI system tested on real clinical images from hospitals or clinics.
**TRL 6**	System prototype demonstrated in a relevant operational environment	Full-scale prototype AI system demonstrated in real-world clinical settings.
**TRL 7**	System prototype demonstrated in operational environment	Operational AI system implemented and used by radiologists for image analysis in multiple hospitals/clinics, e.g., automated analysis for brain metastasis detection.
**TRL 8**	Actual system completed and mission qualified	Fully deployed AI system for medical image analysis in clinical practice; AI tools integrated into PACS (Picture Archiving and Communication System) for routine use.
**TRL 9**	System proven through successful mission operations	AI system continuously in use for diagnosis in clinical settings, with proven real-world success and regulatory approval.

The TRL classification was adapted to the context of imaging-based AI systems, covering stages from early theoretical development (TRL 1–2), laboratory validation (TRL 3–4), prototype testing in relevant clinical environments (TRL 5–6), to real-world clinical implementation and routine use (TRL 7–9).

Supplementary Note 3 - Details of Quality Assessment Based on QUADAS-AI Domains Used to Evaluate the Methodological Quality of the Studies Included

The quality assessment was based on multiple domains from QUADAS-AI, specifically evaluating the methodological quality of the studies. The signaling questions and risk of bias assessment for each domain are as follows:

1. Subject Selection

Signaling questions:

- Was the source, size, and quality of input data accurately characterized along with clear patient eligibility criteria?
- Was the data derived from open-source datasets?

- Was the rationale and breakdown of training, validation, and test sets presented?
- Was image pre-processing performed?
- Was the scanner model information used to acquire imaging data provided?

2. Index Test (AI)

Signaling question:

- Was external verification performed?

3. Reference Standard

Signaling question:

- Was the reference standard likely to correctly classify the target condition?

4. Work-flow

Signaling question:

- Was the time between the index test and the reference standard reasonable?

Additionally, the risk of bias for each domain was assessed as "low," "high," or "unclear." Specifically:

⊕ If all signaling questions for a domain were answered "Yes" the risk of bias for that domain was judged as "low."

⊖ If any signaling question was answered "No," it indicated a potential for bias. Review authors would need to engage in in-depth discussions to assess the risk of bias.

☃The "unclear" category was used only when insufficient data were reported to make a judgment.

Supplementary Note 4 - Assessment of Potential Cohort Overlap

Cohort-overlap screening strategy

To minimize potential double-counting of patient cohorts, we performed a structured screening for overlapping studies from two complementary perspectives: cohort-level characteristics and methodological features.

At the cohort-information level (Table S_X), we cross-checked recruitment periods, sample size, institutional sources, cohort descriptions (including cohort splits), imaging modality, and label distribution.

At the methodological level (Table S_Y), we compared inclusion and exclusion criteria, use of external validation, modeling algorithms, model inputs, region of interest (ROI) definitions, and diagnostic task types.

Table S_X. Cohort-level overlap screening across included studies (clustered by author group and metastatic site).

Cluster (First Author, Metastatic Site)	First Author (Ref), Year	Study Period	Number of Patients	Cohort description	Mean Age of Patients	Imaging Modality	Label Ratio
Cao et al., Brain	Cao et al. [10], 2022	Jan 2017 – Dec 2021	188	Jan 2017 – Dec 2021; Multicenter; Center 1 (primary cohort): 150 patients, stratified into training set (100 patients) and internal validation set (50 patients); Center 2 (external cohort): 38 patients	59.34 ± 8.96	MRI (T1CE, T2W)	**EGFR mutation / EGFR wild-type (n=92/96)**
	Cao et al. [21], 2024	Jan 2018 – Dec 2021	232	Jan 2018 – Dec 2021; Center 1 (training set): 160 patients; Center 2 (external validation set): 72 patients	60.22 ± 11.6	MRI (T1CE, T2W)	**EGFR wild-type / EGFR exon 19 mutation / EGFR exon 21 mutation (n=106/63/63)**
Fan et al., Brain	Fan et al. [12], 2022	Jul 2014 – Oct 2021	310	Center 1 (primary cohort): 230 patients (Jan 2017 – Mar 2021); Center 2 (external cohort): 80 patients (Jul 2014 – Oct 2021)	59.49 ± 8.74	MRI (T1CE, T2W)	**EGFR mutation / EGFR wild-type (n=175/135)**
	Fan et al. [13], 2023	Jan 2017 – Sep 2021	110	Center 1 (primary set): 80 patients (Jan 2017 – Sep 2021); Center 2 (external set): 30 patients (Jan 2018 – Jun 2021)	56.14 ± 9.00	MRI (T1CE, T2W)	**T790M positive / T790M negative (n=45/6)**
	Fan et al. [22], 2023	July 2014 – Dec 2021	310	Center 1 (primary cohort): 230 patients (Jan 2017 – Dec 2021); Center 2	58 ± 12.3	MRI (T1CE,	**EGFR mutation / EGFR wild-type**

Cluster (First Author, Metastatic Site)	First Author (Ref), Year	Study Period	Number of Patients	Cohort description	Mean Age of Patients	Imaging Modality	Label Ratio
				(external cohort): 80 patients (Jul 2014 – Oct 2021)		T2W)	**(n=135/95)**
	Fan et al. [31], 2023	July 2014 – Oct 2021	310	Center 1 (primary cohort): 230 patients (Jan 2017 – Dec 2021); Center 2 (external cohort): 80 patients (Jul 2014 – Oct 2021)	55 – 59	MRI (T1CE, T2W)	**EGFR mutation / EGFR wild-type (n=175/135)**
Li et al., Brain	Li et al. [16], 2023	Jan 2015 – Sep 2022	233	Center 1(Beijing Chest Hospital) :176(Jan 2015 – Oct 2020); Center 2(Capital Medical University):57 (Jan 2019 – Sep 2022)	59.61 ± 8.00	MRI (T2W, T2-FLAIR, DWI, T1CE)	**T790M mutation / no T790M mutation (n=95/138)**
	Li et al. [19], 2024	June 2019 – Jan 2023	399	Training cohort:306 (Jun 2019 – Jan 2023), Testing cohort:93 (Jan 2012 – Jan 2013); Single-center; Lesion count: Training cohort (1146 lesions), Testing cohort (354 lesions)	56.7 ± 11.2	MRI (T1-CE, T2W, T2-FLAIR, DWI)	**EGFR 19Del / EGFR 21L858R / EGFR wild-type (n=150/138/111)**
Cao et al., Bone	Cao et al. [42], 2022	Feb 2016 – Mar 2019	76	38 were with EGFR mutation in exon 19 (50%) and 38 were in exon 21(50%)	59.54	MRI (T1W, T2FS)	**EGFR exon 19 mutation / EGFR exon 21 mutation (n=1/1)**

**Cluster (First Author, Metastatic Site)**	**First Author (Ref), Year**	**Study Period**	**Number of Patients**	**Cohort description**	**Mean Age of Patients**	**Imaging Modality**	**Label Ratio**
	Cao et al. [43], 2023	Feb 2016 – Oct 2020	299	Liaoning Cancer Hospital: 257 patients (Feb 2016 – Oct 2020); Shengjing Hospital: 42 patients (Apr 2017 – Jun 2021)	59.95–60.12	MRI (T1W, T2FS)	EGFR wild-type / EGFR exon 19 mutation / EGFR exon 21 mutation / EGFR exon 18/20 mutation (n=138/71/76/14)
	Fan et al. [44], 2021	Jan 2017 – Dec 2020	94	Cancer Hospital of China Medical University: 94 (Jan 2017- Jan 2020)	59.32 ± 10.28	MRI (T1W, T2FS)	**EGFR mutation / EGFR wild-type (n=38/56)**
Fan et al., Bone	Fan et al. [45], 2022	Jan 2017 – Feb 2021	192	Center 1 (primary cohort): 160 patients (January 2017 – February 2021); Center 2 (external cohort): 32 patients (January 2018 – September 2021)	58 – 63	MRI (T1W, T2FS)	**EGFR mutation / EGFR wild-type (n=100/92)**
	Fan et al. [46], 2022	Jan 2017 – Oct 2021	183	Center 1 (primary set): 159 patients (January 2017 – September 2021); Center 2 (external set): 24 patients (January 2017 – September 2021)	58.71 ± 9.34	MRI (T1W, T2FS)	EGFR wild-type / exon 19 / exon 21 / other (n=121/48/32/21)
Hou et al., Liver	Hou et al. [51], 2024	Feb 2018 – Aug 2022	167	Center 1: 123 patients (Feb 2018 – Dec 2021); Center 2: 44 patients (Nov 2015 – Aug	60.86 ± 9.03	CT, MRI (T1CE)	EGFR mutation / EGFR wild-type (n=54/76)

Cluster (First Author, Metastatic Site)	First Author (Ref), Year	Study Period	Number of Patients	Cohort description	Mean Age of Patients	Imaging Modality	Label Ratio
				2022)			
	Hou et al. [52],2023	Aug 2017 – Dec 2021	130	130 patients (Aug. 2017-Dec. 2021)	56.7 – 61.9	MRI (T1CE, T2W)	EGFR mutation / EGFR wild-type (n=74/93)

Table S_Y. Methodological-level characteristics and key differences (clustered by author group and metastatic site).

Cluster (First Author, Metastatic Site)	First Author (Ref), Year	Inclusion and Exclusion Criteria	External Validation	Modeling Algorithm	Modeling input	ROI Region	Diagnostic task	Clearly Difference
Cao et al., Brain	Cao et al. [10], 2022	Inclusion criteria: (1) older than 18 years' old; (2) underwent brain T1CE and T2W MRI imaging before treatment; (3) with complete genetic testing results of the EGFR mutation status. Exclusion criteria: (1) carry other tumor diseases.	Yes	Lasso + LR	Combined tumor active area (TAA) and peritumoral oedema area (POA)	Whole tumor (W); Tumor active area (TAA); Peritumoral oedema area (POA)	Predict presence of EGFR mutation	Modeling algorithm: ML (Lasso + LR); Modeling input: Combined tumor active area (TAA) and peritumoral oedema area (POA); ROI Region: TAA

Cluster (First Author, Metastatic Site)	First Author (Ref), Year	Inclusion and Exclusion Criteria	External Validation	Modeling Algorithm	Modeling input	ROI Region	Diagnostic task	Clearly Difference
								and POA vs intracranial
	Cao et al. [21], 2024	Inclusion criteria: (1) pathologically confirmed BM from primary NSCLC; (2) EGFR mutation status and mutation sites confirmed by gene-based sequencing of pathological biopsy specimens; (3) complete preoperative BM MRI. Exclusion criteria: (1) presence of other tumors; (2) received radiochemotherapy.	Yes	ResNet + multiscale fusion	224 × 224-pixel ROI patches from T1CE and T2W MR images	Intracranial	EGFR mutation status	Modeling algorithm: DL (ResNet + multiscale fusion); Modeling input: 224 × 224-pixel ROI patches from T1CE and T2W MR images; ROI Region: intracranial
Fan et al., Brain	Fan et al. [12], 2022	Inclusion criteria: (1) underwent contrast-enhanced T1-weighted (T1C) and T2-weighted (T2W) MRI scans before treatment; (2) underwent EGFR-TKI therapy; (3) with no history of systemic anti-cancer treatment. Exclusion criteria: (1) with invisible lesions	Yes	Lasso + LR	Combined M/BP-interface (S2) and tumor active area (TAA)	Tumor margin, necrotic area, peritumoral edema	Predict T790M mutation	Modeling input: M/BP-interface + TAA; ROI Region: Tumor margin, necrotic area, peritumoral edema

Cluster (First Author, Metastatic Site)	First Author (Ref), Year	Inclusion and Exclusion Criteria	External Validation	Modeling Algorithm	Modeling input	ROI Region	Diagnostic task	Clearly Difference
		in brain MRI images; (2) with poor image quality; (3) with history of surgery resection.						
	Fan et al. [13], 2023	Inclusion criteria: (1) high-quality thoracic MRI data before any treatment (eg, surgery, radiochemotherapy, or targeted therapy); (2) complete clinical characteristics; (3) older than 18 years. Exclusion criteria: (1) patient with other tumor disease; (2) incomplete MRI data (eg, missing T1CE or T2W); (3) lack of genetic testing results of the T790M mutation status.	Yes	Lasso + LR	TAA alone, POA alone, and combined TAA+POA	TAA (Tumor Active Area) and POA	Predict acquired T790M mutation	ROI region: TAA (Tumor Active Area) and POA
	Fan et al. [22], 2023	Inclusion criteria: (1) complete gene test results; (2) T1-weighted contrast enhanced (T1CE) and T2-weighted (T2W) brain MRI scans before treatment; (3) complete clinical data. Exclusion	Yes	Lasso + LR	BTI alone and BTI combined with peritumoral edema volume (VPE)	brain–tumor interface (BTI)	EGFR mutation, T790M resistance mutation	ROI region: brain–tumor interface (BTI)

Cluster (First Author, Metastatic Site)	First Author (Ref), Year	Inclusion and Exclusion Criteria	External Validation	Modeling Algorithm	Modeling input	ROI Region	Diagnostic task	Clearly Difference
		criteria: (1) poor MR image quality; (2) other tumor diseases.						
	Fan et al. [31], 2023	Inclusion criteria: (1) pathologically confirmed NSCLC; (2) complete EGFR gene testing results; (3) underwent contrast-enhanced T1-weighted (T1C) and T2-weighted (T2W) brain MRI before any treatment; (4) no other tumor-causing diseases. Exclusion criteria: (1) incomplete clinical or imaging data; (2) poor image quality affecting feature extraction.	Yes	Lasso + LR	TAA alone, POA alone, and combined TAA+POA	Solid tumor area and peritumoral edema (excluding necrosis)	EGFR mutation status	ROI region: Solid tumor area and peritumoral edema (excluding necrosis)
Li et al., Brain	Li et al. [16], 2023	Inclusion criteria: (a) clinical and pathological BM from NSCLC; (b) confirmation of EGFR mutation in primary tumors, blood, or BM (surgery/biopsy); (c) T790M mutation genetic	Yes	Random Forest	Modeling part: Radiomics features from T2WI, T2-FLAIR, DWI, and T1-CE	Brain metastases (>5 mm), excluding peritumoral edema	Predict EGFR mutation status	Modeling Algorithm: Random Forest vs Radiomics + Graph Convolutional Network (GCN)

Cluster (First Author, Metastatic Site)	First Author (Ref), Year	Inclusion and Exclusion Criteria	External Validation	Modeling Algorithm	Modeling input	ROI Region	Diagnostic task	Clearly Difference
		testing results; (d) first high-quality MRI before BM treatment. Exclusion criteria: (a) incomplete clinical data; (b) history of other tumors; (c) other CNS diseases (e.g., cerebral infarction, trauma, Alzheimer's); (d) image artefacts or incomplete MRI.			sequences.			
	Li et al. [19], 2024	Inclusion criteria: (a) initial diagnosis of BM with pathological confirmation; (b) EGFR mutation genetic testing results (BM, primary tumor, or blood); (c) high-quality brain MRI before any treatment (surgery, radiochemotherapy, targeted therapy). Exclusion criteria: (a) history of other tumors or CNS diseases (infarction, trauma,	No	Radiomics + GCN	Fused radiomics features from T1-CE, T2WI, DWI, and T2-FLAIR sequences	Intracranial	EGFR mutation (19del, L858R, WT)	Modeling Algorithm: Random Forest vs Radiomics + Graph Convolutional Network (GCN)

Cluster (First Author, Metastatic Site)	First Author (Ref), Year	Inclusion and Exclusion Criteria	External Validation	Modeling Algorithm	Modeling input	ROI Region	Diagnostic task	Clearly Difference
		inflammation); (b) incomplete or low-quality MRI data; (c) lack of clinical data.						
Cao et al., Bone	Cao et al. [42], 2022	Inclusion criteria were: (1) pathologically confirmed as spinal metastases from primary lung adenocarcinoma; (2) without EGFR-TKI treatment; and (3) age over 18 years. Exclusion criteria were: (1) suffering from other tumor diseases; (2) received phosphate drug for bone metastases or radiochemotherapy; (3) with vertebral compressed fractures; and (4) with diffuse spinal metastasis.	No	Lasso + LR	Combined radiomics features from T1W and T2FS MRI whole tumor regions	Solid tumor enhancement region	Prediction of EGFR mutation subtypes (exon 19 vs exon 21)	ROI Region: Solid tumor enhancement region
	Cao et al. [43], 2023	Inclusion criteria were: (1) age exceed 18 years and (2) no treatment was given before MRI scans. Exclusion criteria were:	Yes	Lasso + LR	Combined radiomics features from T1W and	Intracranial	Prediction of EGFR mutation subtypes	ROI Region: Intracranial

Cluster (First Author, Metastatic Site)	First Author (Ref), Year	Inclusion and Exclusion Criteria	External Validation	Modeling Algorithm	Modeling input	ROI Region	Diagnostic task	Clearly Difference
		(1) suffering from other tumor diseases; (2) received phosphate drug for bone metastases or radiochemotherapy; (3) with vertebral compressed fractures; and (4) with diffuse spinal metastasis.			T2FS MRI whole tumor regions		(exon 19 vs exon 21)	
Fan et al., Bone	Fan et al. [44], 2021	Inclusion criteria were as follows: (i) patients who pathologically diagnosed with thoracic spinal metastases from primary lung adenocarcinoma, (ii) patients who underwent T1W and T2W MRI scans before surgery, and (iii) patients who underwent surgical resection with pathological confirmation. Exclusion criteria were: (i) patients with other tumor diseases, (ii) patients who had phosphate drug for bone	No	Lasso + LR	Inner subregion (S3) of T1W and T2FS MRI	Intracranial	Predict EGFR mutation status	Modeling input: Inner subregion (S3)

Cluster (First Author, Metastatic Site)	First Author (Ref), Year	Inclusion and Exclusion Criteria	External Validation	Modeling Algorithm	Modeling input	ROI Region	Diagnostic task	Clearly Difference
		metastases or radiotherapy on vertebra, and (iii) patients with vertebral compressed fractures.						
	Fan et al. [45], 2022	Inclusion criteria were: (1) patient was diagnosed as bone metastasis from primary lung adenocarcinoma, (2) availability of T1W and T2FS MRI sequences; (3) availability of complete clinical data; and (4) with complete gene test results of the T790M mutation status before and after the EGFR-TKI treatment. Exclusion criteria were: (1) patients with other tumor diseases or vertebral compressed fractures (VCFs); (2) with incomplete MRI data; and (3) with diffuse spinal metastasis.	Yes	Lasso + LR	Whole tumor region of T1W and T2FS MRI	Intracranial	EGFR mutation, T790M resistance mutation	Diagnostic target only
	Fan et al. [46],	Inclusion criteria were as follows: (i) age > 18; (ii) CET1	Yes	Lasso + LR	Whole tumor region of T1W	Intracranial	Predict EGFR	Diagnostic target only

Cluster (First Author, Metastatic Site)	First Author (Ref), Year	Inclusion and Exclusion Criteria	External Validation	Modeling Algorithm	Modeling input	ROI Region	Diagnostic task	Clearly Difference
	2022	MRI scans before surgery; and (iii) complete clinical data. Exclusion criteria were as follows: (i) other malignant tumor diseases; (ii) radiochemotherapy or treatment with phosphate-containing drugs; and (iii) vertebral compressed fractures.			and T2FS MRI		mutation and subtypes (exon 19, exon 21)	
Hou et al., Liver	Hou et al. [51], 2024	Inclusion criteria: (1) pathologically confirmed lung cancer with liver metastasis; (2) genetic testing performed; (3) underwent preoperative MRI scans (including T2W and CE-T1 sequences). Exclusion criteria: (1) lack of complete preoperative MRI images and clinicopathological data; (2) concurrent other malignant tumors; (3) poor MRI image	Yes	Lasso + LR	Combined intratumoral and peritumoral regions of primary tumor and hepatic metastasis	Tumor-liver interface	Predict EGFR mutation status	Modeling input: Combined primary tumor & hepatic metastasis; ROI Region: Tumor-liver interface

Cluster (First Author, Metastatic Site)	First Author (Ref), Year	Inclusion and Exclusion Criteria	External Validation	Modeling Algorithm	Modeling input	ROI Region	Diagnostic task	Clearly Difference
		quality or lesions < 8 mm.						
	Hou et al. [52],2023	Inclusion criteria: (i) older than 18 years; (ii) subsequent surgical resection with pathologically confirmed hepatic metastatic lung adenocarcinoma; (iii) complete CT and MRI data before treatment and complete clinical records. Exclusion criteria: (i) harboring other tumor diseases; (ii) received TKI therapy or radiochemotherapy before image acquisition; (iii) the metastatic tumor is too small to be manually segmented; (iv) MRI images with severe artifact	No	Lasso + LR	Tumor-liver interface (ROI-TLI) and whole tumor region (ROI-W)	Tumor-liver interface and origin site	Predict EGFR mutation status	Modeling input: Tumor-liver interface (ROI-TLI) and whole tumor region (ROI-W); ROI Region: Tumor-liver interface and origin site

Because AI model performance is influenced not only by cohort composition but also by modeling strategies and task definitions, this dual-level assessment was used to improve the robustness of overlap identification. No unequivocally duplicated patient cohorts or duplicate model reports were identified. Studies with potential but unconfirmed overlap were flagged for further sensitivity analysis.

Sensitivity analysis for potential overlap

To evaluate the potential impact of cohort overlap on the pooled estimates, we conducted leave-one-out sensitivity analyses for studies suspected to originate from the same research groups[1]. As shown in Table S_Z, the pooled estimates changed only minimally across iterations, and no single study materially altered the pooled results, which is consistent with the expected behavior of influence diagnostics and sensitivity analyses in meta-analysis[2].

Table S_Z. Leave-one-out sensitivity analysis for pooled diagnostic performance (clustered by author group and metastatic site).

Cluster (First Author, Metastatic Site)	Exclude study	No. of Studies	Overall Sensitivity		Overall Specificity		AUC (95% CI)
			Sensitivity	I^2 (95%CI)	Specificity	I^2 (95%CI)	
	None	36	0.88 (0.87,0.90)	99.1 (98.9-99.3)	0.87 (0.85,0.89)	97.9 (97.5-98.4)	0.91 (0.90-0.93)
Cao et al., Brain	Cao et al. [10], 2022	35	0.87 (0.85,0.89)	98.5 (97.8-99.2)	0.89 (0.86,0.92)	97.7 (96.9-98.5)	0.90 (0.88,0.92)
	Cao et al. [21], 2024	35	0.89 (0.86,0.92)	98.1 (97.4-98.8)	0.87 (0.84,0.90)	97.1 (96.3-97.9)	0.89 (0.87,0.91)
Fan et al., Brain	Fan et al. [12], 2022	35	0.90 (0.87,0.93)	97.7 (97.0-98.4)	0.86 (0.84,0.88)	96.9 (96.1-97.7)	0.91 (0.87,0.95)
	Fan et al. [13], 2023	35	0.87 (0.85,0.89)	98.1 (97.4-98.8)	0.88 (0.84,0.92)	97.2 (96.4-98.0)	0.92 (0.90,0.94)
	Fan et al. [22], 2023	35	0.88 (0.84,0.92)	98.3 (97.6-99.0)	0.87 (0.84,0.90)	97.6 (96.8-98.4)	0.90 (0.87,0.93)
	Fan et al. [31], 2023	35	0.89 (0.87,0.91)	99.1 (98.7-99.5)	0.86 (0.84,0.88)	97.8 (97.0-98.6)	0.89 (0.85,0.93)
Li et al., Brain	Li et al. [16], 2023	35	0.88 (0.87,0.89)	98.0 (97.3-98.7)	0.88 (0.86,0.90)	97.7 (96.9-98.5)	0.93 (0.89,0.97)
	Li et al. [19], 2024	35	0.89 (0.86,0.92)	97.6 (96.9-98.3)	0.87 (0.85,0.90)	96.9 (96.4-97.2)	0.92 (0.88,0.96)
Cao et al., Bone	Cao et al. [42], 2022	35	0.88 (0.85,0.91)	97.8 (97.1-98.5)	0.86 (0.83,0.89)	96.9 (96.1-97.7)	0.89 (0.86,0.92)
	Cao et al. [43], 2023	35	0.86 (0.84,0.88)	98.6 (97.9-99.3)	0.88 (0.85,0.91)	97.7 (96.9-98.5)	0.89 (0.85,0.93)
Fan et al., Bone	Fan et al. [44], 2021	35	0.86 (0.83,0.89)	98.2 (97.5-98.9)	0.87 (0.83,0.91)	96.9 (96.2-97.6)	0.89 (0.87,0.91)

Cluster (First Author, Metastatic Site)	Exclude study	No. of Studies	Overall Sensitivity		Overall Specificity		AUC (95% CI)
Hou et al., Liver	Fan et al. [45], 2022	35	0.88 (0.86,0.90)	98.4 (97.7-99.1)	0.88 (0.84,0.92)	96.6 (96.0-97.2)	0.92 (0.89,0.95)
	Fan et al. [46], 2022	35	0.90 (0.88,0.92)	97.9 (97.2-98.6)	0.86 (0.85,0.87)	97.0 (96.2-97.8)	0.89 (0.85,0.93)
	Hou et al. [51], 2024	35	0.87 (0.84,0.90)	98.3 (97.6-99.0)	0.89 (0.85,0.93)	96.8 (96.0-97.6)	0.92 (0.89,0.95)
	Hou et al. [52],2023	35	0.89 (0.85,0.93)	99.1 (98.8-99.4)	0.87 (0.83,0.91)	97.4 (96.6-98.2)	0.92 (0.89,0.95)

Supplementary References

1. Viechtbauer W, Cheung MW-L. Outlier and influence diagnostics for meta-analysis. Res Synth Methods. 2010;1(2):112–125.

2. Higgins JPT, Thomas J, Chandler J, et al., eds. Cochrane Handbook for Systematic Reviews of Interventions Version 6.5 (updated August 2024). Cochrane; 2024. Available from: https://www.cochrane.org/handbook.

Supplementary Table 1. Participant demographics for diagnosis-related studies (Molecular-level prediction, Metastasis differentiation, and Tumor type classification; n = 52)

First Author (Ref), Year	Metastatic Site	Region	Number of Patients	Mean Age of Patients	Imaging Modality	Image Source	Primary Tumor Inclusion	Low-quality Image Exclusion	Study Period	Reference Standard for Metastatic Lesions	Characteristics of Metastatic Lesions	TRL
Grossman et al. [1], 2021	Brain	Israel	69	63.7 ± 10.5	MRI (T1CE, T2W, FLAIR)	Pre-treatment	No	NR	NR	Histopathological diagnosis	Mean tumor volume: 18.3 ± 22.9 cm³ (no between-group difference, p = 0.48); up to three metastases per patient.	3
Park et al. [2], 2021	Brain	Korea	51	59.9 ± 12.5	MRI (T1CE, DTI)	Pre-treatment	No	Yes, Patients with prior Gamma Knife surgery or whole brain radiotherapy were excluded.	Aug 2011 – Jun 2018	Medical records	Mean maximal tumor diameter: 17.7 ± 8.2 mm (range 10–45 mm); lesions <10 mm were excluded; multiple lesions per patient were included.	3

First Author (Ref), Year	Metastatic Site	Region	Number of Patients	Mean Age of Patients	Imaging Modality	Image Source	Primary Tumor Inclusion	Low-quality Image Exclusion	Study Period	Reference Standard for Metastatic Lesions	Characteristics of Metastatic Lesions	TRL
Sun et al. [3], 2024	Brain	China	161	62.3 ± 7.1	MRI (T1CE, FLAIR, DWI, CE-SWI)	Pre-treatment	No	Yes, excluded patients with incomplete MRI or severe imaging artifacts	Jan 2021 – Dec 2023	Medical records	Only lesions ≥ 3 mm were included	4
Wang et al. [4], 2021	Brain	China	52	55.82 ± 9.08	MRI (T1CE, T2-FLAIR, T2W, DWI)	Pre-treatment	No	Yes, Excluded patients with incomplete or non-contrast MRI scans	Jun 2015 – Jul 2018	MR examinations	NR	5
Wu et al. [5], 2024	Brain	China	125	60.7 ± 10.8	MRI (T1W, T2W, T2-FLAIR, DWI, T1CE)	Pre-treatment	No	Yes, Excluded brain MRIs with artifacts or missing sequences	Jan 2010 – Dec 2019	Medical records	Number of lesions ≤10 mm: 8.11 ± 14.78 vs. 8.34 ± 29.02; >10 mm: 0.66 ± 1.32 vs. 0.98 ± 1.67.	4

First Author (Ref), Year	Metastatic Site	Region	Number of Patients	Mean Age of Patients	Imaging Modality	Image Source	Primary Tumor Inclusion	Low-quality Image Exclusion	Study Period	Reference Standard for Metastatic Lesions	Characteristics of Metastatic Lesions	TRL
Xu et al. [6], 2024	Brain	China	86	61.47 ± 11.79	MRI (T1W, T2W, T1CE)	Pre-treatment	No	NR	Jun 2019 – Oct 2023	Medical records	maximum diameter was ≥3 mm Regression categorization: 4–10 vs. >10 metastases	5
Yang et al. [7], 2024	Brain	China	293	60.34 ± 8.88	MRI (T1CE, T2W)	Pre-treatment	No	Yes, Excluded T1CE or T2W missing images	Jul 2014 – Dec 2022	MRI diagnosis and genetic testing	NR	5
Zhang et al. [8], 2021	Brain	China	144	62 ± 20	MRI (T1CE)	Pre-treatment	No	Yes, excluded: missing post-contrast T1 images, extracranial metastases, non-AD/SCC histology	Jan 2014 – Dec 2016	CT diagnosis	Patients with ≤10 metastases were included; for those with ≥2 metastases, all lesions were contoured.	4

First Author (Ref), Year	Metastatic Site	Region	Number of Patients	Mean Age of Patients	Imaging Modality	Image Source	Primary Tumor Inclusion	Low-quality Image Exclusion	Study Period	Reference Standard for Metastatic Lesions	Characteristics of Metastatic Lesions	TRL
Zheng et al. [9],2021	Brain	China	162	54±10	MRI (T1CE, T2W, T2-FLAIR)	Pre-treatment	No	Yes, excluded: missing pathology data, no EGFR testing, no pre-treatment MRI, prior brain RT	Jul 2014 – Jul 2021	Histopathological diagnosis	Mean lesion diameter: 39 ± 14 mm (range 13–76 mm); 58.6% single BM; 6.8% >10 lesions; mainly cerebrum/cerebellum.	4
Cao et al. [10], 2022	Brain	China	188	59.34 ± 8.96	MRI (T1CE, T2W)	Pre-treatment	No	Yes (excluded cases with severe artifacts or missing data)	Jan 2017 – Dec 2021	Medical records	NR	5

First Author (Ref), Year	Metastatic Site	Region	Number of Patients	Mean Age of Patients	Imaging Modality	Image Source	Primary Tumor Inclusion	Low-quality Image Exclusion	Study Period	Reference Standard for Metastatic Lesions	Characteristics of Metastatic Lesions	TRL
Chen et al. [11], 2020	Brain	China	110	57.51 ± 12.32	MRI (T1CE, FLAIR)	Pre-treatment	No	Yes (excluded: images < 5 mm slice thickness, missing FLAIR, motion/artefact severe images)	Jan 2009 – Dec 2017	MRI diagnosis	Lesions >5 mm were included; if >10 lesions were present, only the 10 largest were analyzed.	4
Fan et al. [12], 2022	Brain	China	310	59.49 ± 8.74	MRI (T1CE, T2W)	Pre-treatment	No	Yes, excluded poor quality or artifact-affected images	Jul 2014 – Oct 2021	Medical records	All lesions were located in the brain parenchyma as brain metastases (BM)	5
Fan et al. [13], 2023	Brain	China	110	56.14 ± 9.00	MRI (T1CE, T2W)	Pre-treatment	No	Yes, excluded poor quality or artifact-affected images	Jan 2017 – Sep 2021	Medical records	All lesions were brain parenchymal metastases originating from primary NSCLC	4

First Author (Ref), Year	Metastatic Site	Region	Number of Patients	Mean Age of Patients	Imaging Modality	Image Source	Primary Tumor Inclusion	Low-quality Image Exclusion	Study Period	Reference Standard for Metastatic Lesions	Characteristics of Metastatic Lesions	TRL
Haim et al. [14], 2022	Brain	Israel	59	62.0 ± 11.7	MRI (T1CE)	Pre-treatment	No	Yes, excluded post-therapy or obviously artifact-laden images	Jan 2009 –Jan 2019	Histopathological diagnosis	EGFR wild-type: mean tumor volume 1.21 ± 0.58 cm³; EGFR mutant: mean tumor volume 0.96 ± 0.52 cm³; one lesion per patient was analyzed.	3
Huang et al. [15], 2024	Brain	China	58	56.7 ± 10.3	MRI (T1W, T2W, FLAIR, DWI, T1CE)	Pre-treatment	No	Yes, excluded low-quality MRI images	Jun 2014 – Dec 2022	MRI diagnosis	52.8% of lesions had a tumor volume >2 cm³, and 65.9% were located in the anterior circulation.	3

First Author (Ref), Year	Metastatic Site	Region	Number of Patients	Mean Age of Patients	Imaging Modality	Image Source	Primary Tumor Inclusion	Low-quality Image Exclusion	Study Period	Reference Standard for Metastatic Lesions	Characteristics of Metastatic Lesions	TRL
Li et al. [16], 2023	Brain	China	233	59.61 ± 8.00	MRI (T2W, T2-FLAIR, DWI, T1CE)	Pre-treatment	No	Yes (excluded cases with artifacts or incomplete imaging data)	Jan 2015 – Sep 2022	Medical records	Lesions ≥5 mm were included; multiple metastatic lesions were allowed.	5
Meißner et al. [17], 2023	Brain	Germany	53	61.1 ± 9.1	MRI (T1CE)	Pre-treatment	NR	NR	Jan 2014 –Jan 2020	Histopathological diagnosis	Mean contrast-enhancing tumor volume: 24.6 ± 29.1 mL; 50% had solitary metastasis and 38.9% had 2–3 metastases.	4

First Author (Ref), Year	Metastatic Site	Region	Number of Patients	Mean Age of Patients	Imaging Modality	Image Source	Primary Tumor Inclusion	Low-quality Image Exclusion	Study Period	Reference Standard for Metastatic Lesions	Characteristics of Metastatic Lesions	TRL
Zhu et al. [18], 2024	Brain	China	250	63.22 ± 10.61	MRI (T1CE, T2W, FLAIR)	Pre-treatment	No	Yes, excluded patients with image artifacts	Dec 2010 – Apr 2023	Medical records	Mean maximum tumor diameter: 23.40 ± 11.07 mm; mean maximum edema diameter: 42.54 ± 29.54 mm; mean tumors per patient: 4.20 ± 5.55.	5
Li et al. [19], 2024	Brain	China	399	56.7 ± 11.2	MRI (T1-CE, T2W, T2-FLAIR, DWI)	Pre-treatment	No	Yes, low-quality images and lesions <5mm were excluded	June 2019 – Jan 2023	Histopathological diagnosis	Lesions ≥5 mm were included; multiple lesions per patient were allowed.	4

First Author (Ref), Year	Metastatic Site	Region	Number of Patients	Mean Age of Patients	Imaging Modality	Image Source	Primary Tumor Inclusion	Low-quality Image Exclusion	Study Period	Reference Standard for Metastatic Lesions	Characteristics of Metastatic Lesions	TRL
Zhou et al. [20], 2023	Brain	China	226	57 ± 10.2	MRI (TIC)	Pre-treatment	No	NR	Aug 2018 – Oct 2021	Medical records	Lesions with a maximum diameter ≥10 mm were included; multiple lesions per patient were allowed.	5
Cao et al. [21], 2024	Brain	China	232	60.22 ± 11.6	MRI (T1W-CE, T2W)	Pre-treatment	No	NR	Jan 2018 – Dec 2021	Medical records	Lesions with a maximum diameter ≥5 mm were included; multiple lesions per patient were allowed.	5
Fan et al. [22], 2023	Brain	China	310	58 ± 12.3	MRI (T1CE, T2W)	Pre-treatment	No	Yes, excluded patients with imaging artifacts	July 2014 – Dec 2021	Medical records	NR	5

First Author (Ref), Year	Metastatic Site	Region	Number of Patients	Mean Age of Patients	Imaging Modality	Image Source	Primary Tumor Inclusion	Low-quality Image Exclusion	Study Period	Reference Standard for Metastatic Lesions	Characteristics of Metastatic Lesions	TRL
Ahn et al. [23], 2020	Brain	Korea	61	62.3 ± 11.6	MRI (T1CE)	Pre-treatment	No	Yes, excluded patients with imaging artifacts	June 2012 – July 2018	Medical records	Measurable BMs were defined as Measurable BMs (>3 mm): mean 3.5 ± 3.3 (EGFR wild type) vs. 3.4 ± 3.0 (EGFR mutation) per patient.	4
Ouyang et al. [24], 2025	Brain	China	309	52 – 56	CT and MRI (T2W, T1CE)	Pre-treatment	Yes	Yes, excluded patients with image artifacts	June 2017 – June 2021	Medical records	Brain metastases ≥1 cm were included; multiple metastases were allowed.	5

First Author (Ref), Year	Metastatic Site	Region	Number of Patients	Mean Age of Patients	Imaging Modality	Image Source	Primary Tumor Inclusion	Low-quality Image Exclusion	Study Period	Reference Standard for Metastatic Lesions	Characteristics of Metastatic Lesions	TRL
Sui et al. [25], 2023	Brain	China	192	61 ± 10	MRI (T1CE)	Pre-treatment	No	Yes, and lesion size required to be larger than 30 pixels	Jan 2016 – June 2019	Histopathological and imaging diagnosis	Only lesions ≥30 pixels were included for model training.	4
Cao et al. [26], 2025	Brain	China	288	61.4 ± 12.3	T1-CE, T2W	Pre-treatment	No	Yes, excluded patients with image artifacts	Jan2019 – Dec 2023	Histopathological and imaging diagnosis	Only lesions with a diameter > 5 mm were included.	5
Li et al. [27], 2016	Brain	China	126	NR	T1-CE	Pre-treatment	No	NR	Aug 2014 – Mar2015	MRI diagnosis	NR	4
Li et al. [28], 2022	Brain	China	186	57.38 ± 13.22	T1-CE (contrast-enhanced T1), T2-FLAIR, T2W	Pre-treatment	No	Yes, excluded incomplete or artifact-affected images	Dec 2009 – Jan 2022	Histopathological diagnosis and genetic testing	Only lesions ≥ 5 mm were included	4

First Author (Ref), Year	Metastatic Site	Region	Number of Patients	Mean Age of Patients	Imaging Modality	Image Source	Primary Tumor Inclusion	Low-quality Image Exclusion	Study Period	Reference Standard for Metastatic Lesions	Characteristics of Metastatic Lesions	TRL
Li et al. [29], 2024	Brain	China	246	59.21 – 60.80	T1W, T2W, T2-FLAIR, DWI, CE-T1W	Pre-treatment	No	Yes, excluded motion/artifact, metal artifact, and poor-quality images	July 2016 – June 2022	Medical records	NR	5
Li et al. [30], 2024	Brain	China	268	NR	T1-CE (contrast-enhanced T1), T2-FLAIR	Pre-treatment	No	Yes, excluded patients with no genetic testing or missing imaging data; preprocessed by normalization and imputation	Jan 2015 – Jan 2022	Histopathological diagnosis	NR	4

First Author (Ref), Year	Metastatic Site	Region	Number of Patients	Mean Age of Patients	Imaging Modality	Image Source	Primary Tumor Inclusion	Low-quality Image Exclusion	Study Period	Reference Standard for Metastatic Lesions	Characteristics of Metastatic Lesions	TRL
Fan et al. [31], 2023	Brain	China	310	55 – 59	T1CE (contrast-enhanced T1), T2W (T2-weighted), T2-weighted	Pre-treatment	Yes	Yes, only included cases with good image quality, confirmed EGFR status, and no other tumor diseases	July 2014 – Oct 2021	Histopathological and imaging diagnosis	NR	5
Tulum et al. [32], 2023	Brain	Turkey	74	NR	T1CE (3D contrast-enhanced T1), T2W, FLAIR	Pre-treatment	No	Yes, included only lesions with diameter >1 cm, showing metastatic characteristics; excluded atypical cases	Mar 2020 – Mar 2022	Histopathological and imaging diagnosis	Lesions larger than 1 cm in diameter were included	3

First Author (Ref), Year	Metastatic Site	Region	Number of Patients	Mean Age of Patients	Imaging Modality	Image Source	Primary Tumor Inclusion	Low-quality Image Exclusion	Study Period	Reference Standard for Metastatic Lesions	Characteristics of Metastatic Lesions	TRL
Zhu et al. [33], 2023	Brain	China	277	52-56	CE-T1W	Pre-treatment	No	Yes, excluded 20 glioma patients with poor image quality	Apr 2016 – Sep 2022	Medical records	Lesions with a maximum diameter <5 mm were excluded; mean maximum tumor diameter 23.40 ± 11.07 mm; mean maximum edema diameter 42.54 ± 29.54 mm; mean number of lesions 4.20 ± 5.55.	4
Hu et al. [34],2024	Brain/Bone	China	BM = 150, SM = 140	58.21 ± 9.28	T1CE	Pre-treatment	Yes	Yes, clearly excluded patients with incomplete images or missing data	Nov 2017 – Dec 2021	Histopathological diagnosis	NR	5

First Author (Ref), Year	Metastatic Site	Region	Number of Patients	Mean Age of Patients	Imaging Modality	Image Source	Primary Tumor Inclusion	Low-quality Image Exclusion	Study Period	Reference Standard for Metastatic Lesions	Characteristics of Metastatic Lesions	TRL
Artzi et al. [35], 2019	Brain	Israel	227	60 ± 13	MRI (T1W)	Pre-treatment	No	Yes, excluded low-quality or incomplete T1CE datasets	NR	Histopathological diagnosis	Patients included those with solitary or multiple brain lesions, yet only a single lesion was extracted and analyzed for each patient	4

First Author (Ref), Year	Metastatic Site	Region	Number of Patients	Mean Age of Patients	Imaging Modality	Image Source	Primary Tumor Inclusion	Low-quality Image Exclusion	Study Period	Reference Standard for Metastatic Lesions	Characteristics of Metastatic Lesions	TRL
Han et al. [36],2021	Brain	China	168	54.6 ± 11.6	MRI (T1W)	Pre-treatment	No	Yes – excluded poor-quality or incomplete images per criteria	Jan 2010 – Dec 2017	Histopathological diagnosis	Only solitary supratentorial brain metastases were included All metastatic lesions were located in the supratentorial region Training cohort: 36.10 ± 14.82 mm Internal validation cohort: 37.28 ± 16.31 mm External validation cohort: 42.03 ± 17.25 mm	5

First Author (Ref), Year	Metastatic Site	Region	Number of Patients	Mean Age of Patients	Imaging Modality	Image Source	Primary Tumor Inclusion	Low-quality Image Exclusion	Study Period	Reference Standard for Metastatic Lesions	Characteristics of Metastatic Lesions	TRL
Chen et al. [37], 2019	Brain	China	134	51.6 ± 13.1	MRI (T1CE)	Pre-treatment	No	Yes, patients with other intracranial diseases or poor-quality MRI were excluded	Apr 2014 – Dec 2018	MRI diagnosis	Lesions smaller than 5 mm were excluded	4
Huang et al. [38],2022	Brain	China	187	NR	MRI (T1W, T2W, T1CE)	Pre-treatment	No	NR	Jan 2010-Dec 2018	Medical records	Lesion location: anterior circulation 65.9%, posterior circulation 30.9%, deep grey nuclei 3.25%; tumor volume: ≤2 cm^3 47.2% and >2 cm^3 52.8%.	4

First Author (Ref), Year	Metastatic Site	Region	Number of Patients	Mean Age of Patients	Imaging Modality	Image Source	Primary Tumor Inclusion	Low-quality Image Exclusion	Study Period	Reference Standard for Metastatic Lesions	Characteristics of Metastatic Lesions	TRL
Duan et al. [39],2023	Bone	China	730	NR	T1W, T2W, STIR	Pre-treatment	No	Yes, excluded patients with motion artifacts, incomplete images, or uncertain diagnosis	Jan 2016 – June 2021	MRI diagnosis	NR	5
Lang et al. [40],2019	Bone	China	61	56 ± 11	T2W, DCE, T1W	Pre-treatment	Yes	NR	2011 – 2015	Medical records	NR	4
Cheng et al. [41], 2023	Bone	China	203	58.12 ± 9.62	CT, MRI (T1W, T2FS)	Pre-treatment	Yes	Yes (excluded severe artifacts and large transfer lesions)	Dec 2017 – Sep 2021	Medical records	Lesions ≥10 mm were included; one lesion per patient was selected.	4

First Author (Ref), Year	Metastatic Site	Region	Number of Patients	Mean Age of Patients	Imaging Modality	Image Source	Primary Tumor Inclusion	Low-quality Image Exclusion	Study Period	Reference Standard for Metastatic Lesions	Characteristics of Metastatic Lesions	TRL
Cao et al. [42], 2022	Bone	China	76	59.54	MRI (T1W, T2FS)	NR	No	NR	Feb 2016 – Mar 2019	Histopathological diagnosis	All lesions were located in the thoracic spine	4
Cao et al. [43], 2023	Bone	China	299	59.95–60.12	MRI (T1W, T2FS)	Pre-treatment	No	Yes: Exclusion criteria included primary malignant tumors, other spinal diseases, prior history of radiotherapy or chemotherapy	Feb 2016 – Oct 2020	Medical records	All metastatic lesions were located in the spine	5
Fan et al. [44], 2021	Bone	China	94	59.32 ± 10.28	MRI (T1W, T2FS)	Pre-treatment	No	Yes: Excluded other tumor diseases, vertebral compression fractures or degenerative diseases	Jan 2017 – Dec 2020	Histopathological diagnosis	NR	3

First Author (Ref), Year	Metastatic Site	Region	Number of Patients	Mean Age of Patients	Imaging Modality	Image Source	Primary Tumor Inclusion	Low-quality Image Exclusion	Study Period	Reference Standard for Metastatic Lesions	Characteristics of Metastatic Lesions	TRL
Fan et al. [45], 2022	Bone	China	192	58 – 63	MRI (T1W, T2FS)	Pre-treatment	No	Yes, excluded: other tumor history, vertebral compression fractures, benign tumors, spinal degenerative diseases, incomplete MRI	Jan 2017 – Feb 2021	Histopathological diagnosis	NR	5
Fan et al. [46], 2022	Bone	China	183	58.71 ± 9.34	MRI (T1W, T2FS)	Pre-treatment	No	Yes, excluded: other malignancies, prior radiotherapy or bisphosphonate therapy, vertebral compression fractures	Jan 2017 – Oct 2021	Histopathological diagnosis	NR	5

First Author (Ref), Year	Metastatic Site	Region	Number of Patients	Mean Age of Patients	Imaging Modality	Image Source	Primary Tumor Inclusion	Low-quality Image Exclusion	Study Period	Reference Standard for Metastatic Lesions	Characteristics of Metastatic Lesions	TRL
Jiang et al. [47], 2021	Bone	China	97	61.74 ± 10.94	MRI (T1W, T2W, T2FS)	Pre-treatment	No	Yes, excluded: benign spinal tumors, MRI artifacts, vertebral compression fractures, radiotherapy/bisphosphonate history	Jan 2016 – Dec 2018	Histopathological diagnosis	All lesions were lumbar spinal bone metastases	3
Jiang et al. [48], 2023	Bone	China	265	59.7 ± 9.0	MRI (T1W, T2W)	Pre-treatment	No	Yes, excluded: other tumors, radiotherapy history, imaging artifacts/poor quality	NR	Medical records	All lesions were spinal bone metastases.	5

First Author (Ref), Year	Metastatic Site	Region	Number of Patients	Mean Age of Patients	Imaging Modality	Image Source	Primary Tumor Inclusion	Low-quality Image Exclusion	Study Period	Reference Standard for Metastatic Lesions	Characteristics of Metastatic Lesions	TRL
Ren et al. [49], 2021	Bone	China	162	59.9 – 60.1	MRI (T1W, T2W, T2FS)	Pre-treatment	No	Yes, excluded: imaging artifacts, incomplete data, radiotherapy history	Jan 2016 – Apr 2021	Histopathological diagnosis	All lesions were thoracic spinal metastases	5
Wang et al. [50], 2024	Bone	China	141	66.69 ± 8.34	WBS + CT + SPECT	Pre-treatment	No	Yes, excluded motion artifacts, incomplete data, primary bone tumors, etc.	July 2020 – June 2022	Medical records	NR	4
Hou et al. [51], 2024	Liver	China	165	56.7 – 61.9	MRI (T1CE, T2W)	Pre-treatment	No	Yes, excluded patients with poor MRI image quality or lesions < 8 mm	Feb 2018 – Aug 2022	Histopathological diagnosis	Liver metastases from lung cancer were included; lesions <8 mm were excluded.	5

First Author (Ref), Year	Metastatic Site	Region	Number of Patients	Mean Age of Patients	Imaging Modality	Image Source	Primary Tumor Inclusion	Low-quality Image Exclusion	Study Period	Reference Standard for Metastatic Lesions	Characteristics of Metastatic Lesions	TRL
Hou et al. [52],2023	Liver	China	130	60.86 ± 9.03	CT, MRI (T1CE)	Pre-treatment	Yes	Yes, excluded patients with severe imaging artifacts or lesions too small to assess	Aug 2017 – Dec 2021	Histopathological diagnosis	Metastases were present at initial diagnosis	4

Supplementary Table 2. Participant demographics for Outcome prediction studies (n = 13)

First Author (Ref), Year	Metastatic Site	Region	Number of Patients	Mean Age of Patients	Imaging Modality	Image Source	Primary Tumor Inclusion	Low-quality Image Exclusion	Study Period	Reference Standard for Metastatic Lesions	Characteristics of Metastatic Lesions	TRL
Qi et al. [53], 2024	Brain	China	194	60.0 ± 11.3	MRI (T1C, T2-FLAIR DWI, T1W, and T2W)	Pre-treatment	No	Yes, patients with image artifacts affecting quality were excluded	Jan 2017 – Mar 2023	Medical records	NR	4
Xu et al. [54], 2024	Brain	China	174	59.0 ± 18.2	MRI (T1C)	Pre-treatment	No	Yes, MRI images with poor quality due to motion artifacts or inadequate contrast injection were excluded	Jun 2019 – Jun 2022	Medical records	NR	5
Zhao et al. [55], 2021	Brain	China	87	50.5 ± 18.5	MRI (T1C, T2W)	Pre-treatment	No	Yes, images with poor quality due to motion artifacts or contrast-related signal inconsistency were excluded	Sep 2017 – Jul 2019	Imaging diagnosis	Inclusion criterion: lesions ≥0.5 cm.	3

First Author (Ref), Year	Metastatic Site	Region	Number of Patients	Mean Age of Patients	Imaging Modality	Image Source	Primary Tumor Inclusion	Low-quality Image Exclusion	Study Period	Reference Standard for Metastatic Lesions	Characteristics of Metastatic Lesions	TRL
Qu et al. [56], 2024	Brain	China	70	56.9 ± 10.5	MRI (CE-T1WI, T1WI, T2WI, T2-FLAIR, DWI)	Pre-treatment	No	Yes, excluded 66 cases in total due to poor image quality (n = 10) and missing clinical data (n = 28)	Feb 2018 – Aug 2021	imaging diagnosis	Lesions must have a minimum diameter of 10 mm.	3
Qi et al. [57], 2024	Brain	China	117	59.0 ± 8.7	MRI (T1+C, T1WI, T2WI, T2-FLAIR, DWI)	Pre-treatment	No	Yes, clearly excluded images with poor quality, local therapy cases.	Jan 2014 – Dec 2021	Medical records	NR	4
Park et al. [58], 2021	Brain	Korea	83	61.0 ± 11.0	MRI (T1C, T2W)	Pre-treatment	No	Yes, excluded patients with WBRT history, lesions < 1 cm, purely cystic lesions, or poor-quality images	May 2010 – Oct 2015	Medical records	Tumor size was assessed by maximal tumor diameter, with a mean of 17.7 mm	3

First Author (Ref), Year	Metastatic Site	Region	Number of Patients	Mean Age of Patients	Imaging Modality	Image Source	Primary Tumor Inclusion	Low-quality Image Exclusion	Study Period	Reference Standard for Metastatic Lesions	Characteristics of Metastatic Lesions	TRL
Fan et al. [31], 2023	Brain	China	310	57.0 ± 2.0	MRI (T1C, T2W)	Pre-treatment	Yes	Yes, included only patients with good image quality and no other malignancies	Jul 2014 – Oct 2021	Histopathological diagnosis	NR	5
Lv et al. [59], 2023	Brain	China	405	56.5 ± 12.0	MRI (T1-CE, T2WI, T2-FLAIR, DWI)	Pre-treatment + Post-treatment	No	Yes, excluded cases with poor image quality or other tumors	Jan 2014 – Jan 2023	Imaging diagnosis	NR	5
Liao et al. [60], 2021	Brain	China	307	60.8 ± 28.2	MRI (T1WI, T1CE, T2W)	Pre-treatment	No	Yes, Patients with incomplete clinical information were excluded	Jan 2011 – Dec 2018	MRI diagnosis	NR	4
Sun et al. [61], 2024	Brain	China	53	60.4 ± 9.3	MRI (T1CE)	Pre-treatment	No	Yes, Excluded patients with incomplete imaging data, intracranial lesions	Jan 2018 – Jun 2023	Imaging diagnosis	Patients had multiple brain metastases at diagnosis	3

First Author (Ref), Year	Metastatic Site	Region	Number of Patients	Mean Age of Patients	Imaging Modality	Image Source	Primary Tumor Inclusion	Low-quality Image Exclusion	Study Period	Reference Standard for Metastatic Lesions	Characteristics of Metastatic Lesions	TRL
								prior to treatment, or prior radiotherapy				
Wang et al. [62], 2024	Brain	China	72	65.5 ± 8.5	MRI (T1W, T2W, T1CE), CT	Pre-treatment	Yes	Yes, patients with image artifacts affecting quality were excluded	Jan 2018 – Dec 2020	MRI diagnosis	Included patients: 1 to ≥4 brain metastases	3
Chen et al. [63], 2021	Brain	China	110	57.5 ± 12.3	MRI (T1CE, T2-FLAIR)	Pre-treatment	No	Yes, images were retained based on preprocessing quality evaluation	Jan 2009 – Dec 2017	MRI diagnosis	NR	3
Gutsche et al. [64], 2022	Brain	Germany	150	64.0 ± 11.0	MRI (T1CE, T2, FLAIR)	Pre-treatment	No	NR	Jan 2012 – Dec 2019	Medical records	Mean metastasis volume: 1.7 mL (responding) vs 2.8 mL (non-responding) ($p<0.001$).	4

Supplementary Table 3. Validation methods and algorithms for diagnosis-related studies (Molecular-level prediction, Metastasis differentiation, and Tumor type classification; n = 52).

First Author (Ref), Year	Internal Validation Type	External Validation	Modeling Algorithm	ML/DL	Manual	ROI Region	Model Variables	Diagnostic Target	Gold Standard Type	AI versus clinicians
Grossman et al. [1], 2021	5-fold cross-validation	No	EfficientNet	DL	Yes	Intracranial	Radiomic features	Classification of BM from SCLC and NSCLC	Histopathology	No
Park et al. [2], 2021	10-fold cross-validation	No	SVM, AdaBoost, LDA	ML	Yes	Intracranial	Radiomic features	EGFR mutation status	Genetic testing	No
Sun et al. [3], 2024	Random split validation	No	LR, RF, SVM, XGBoost	ML	Yes	Intracranial	Radiomic features, clinical variables	Identify molecular mutation status	Histopathology	No
Wang et al. [4], 2021	Random split validation	Yes	LR	ML	Yes	Intracranial	Radiomic features	EGFR mutation status	Genetic testing	No
Wu et al. [5], 2024	3-fold and 5-fold cross-validation	No	LR, SVM, Random Forest, XGBoost	ML	Yes	Intracranial	Radiomic features	T790M mutation status	Genetic testing	No
Xu et al. [6], 2024	5-fold cross-validation	Yes	AdaBoost, LASSO +	ML	Yes	Intracranial	Radiomic features, clinical	EGFR mutation status	Genetic testing	No

First Author (Ref), Year	Internal Validation Type	External Validation	Modeling Algorithm	ML/DL	Manual	ROI Region	Model Variables	Diagnostic Target	Gold Standard Type	AI versus clinicians
			LR				variables			
Yang et al. [7], 2024	Random sampling validation	Yes	LASSO + LR	ML	Yes	Tumor Active Area and Peritumoral Edema Area	Radiomic features	EGFR negative, EGFR19 mutation, EGFR21 mutation	Genetic testing	No
Zhang et al. [8], 2021	Random split validation	No	SVM, LR	ML	Yes	Intracranial	Radiomic features, clinical variables	classification of squamous cell carcinoma versus adenocarcinoma	Histopathology	No
Zheng et al. [9],2021	Random split validation	No	LASSO + LR	ML	Yes	Intracranial	Radiomic features	EGFR mutation status	Genetic testing	No
Cao et al. [10], 2022	Stratified random sampling	Yes	Lasso+LR	ML	Yes	Whole tumor (W); Tumor active area (TAA); Peritumoral oedema area (POA)	Radiomic features, clinical features	Predict presence of EGFR mutation	Genetic testing	No

First Author (Ref), Year	Internal Validation Type	External Validation	Modeling Algorithm	ML/DL	Manual	ROI Region	Model Variables	Diagnostic Target	Gold Standard Type	AI versus clinicians
Chen et al. [11], 2020	LOOCV	No	RF	ML	Yes	Enhancing tumor; Peritumoral edema	Radiomic features, clinical features	Identify molecular mutation status	Molecular tests (FISH, IHC, NGS, etc.) from primary lung tissue	No
Fan et al. [12], 2022	Stratified random sampling	Yes	Lasso+LR	ML	Yes	Tumor margin, necrotic area, peritumoral edema	Radiomic feature	Predict T790M mutation	NGS test of primary lung tumor tissue	No
Fan et al. [13], 2023	Stratified random sampling	Yes	Lasso+LR	ML	Yes	TAA (Tumor Active Area) and POA	Radiomic feature	Predict acquired T790M mutation	Plasma ctDNA testing	No
Haim et al. [14], 2022	5-fold cross-validation	No	ResNet-50 + transfer learning (pretrained on ImageNet) + mixup augmentation	DL	Yes	Single lesion resection area and its peritumoral region	DL Imaging features	Predict EGFR mutation status	Pathological diagnosis + genetic test	No

First Author (Ref), Year	Internal Validation Type	External Validation	Modeling Algorithm	ML/DL	Manual	ROI Region	Model Variables	Diagnostic Target	Gold Standard Type	AI versus clinicians
Huang et al. [15], 2024	5-fold cross-validation	No	LASSO+LR	ML	Yes	whole tumor, enhancing tumor, and peritumoral region (1mm/3mm/5mm)	Radiomic features, clinical features	Predict EGFR mutation status	Pathological diagnosis + genetic test	No
Li et al. [16], 2023	10-fold cross-validation	Yes	Random Forest	ML	Yes	Brain metastases (>5 mm), excluding peritumoral edema	Radiomic features	Predict EGFR mutation status	Pathological diagnosis + genetic test	No
Meißner et al. [17], 2023	5-fold stratified cross-validation	Yes	Random Forest (RF)	ML	Yes	Intracranial	Radiomic features, clinical features	Predict PD-L1 expression (≥1%)	Immunohistochemistry (IHC), using E1L3N antibody	No
Zhu et al. [18], 2024	5-fold Cross-validation	Yes	ResNet101 and SVM	DL	Yes	Intracranial	DL features + clinical features	Pathological subtypes	Histopathology	No
Li et al. [19], 2024	5-fold cross-validation	No	Radiomics + GCN	ML+DL	Yes	Intracranial	Radiomic features + DL	EGFR mutation (19del, L858R,	Genetic testing	No

First Author (Ref), Year	Internal Validation Type	External Validation	Modeling Algorithm	ML/DL	Manual	ROI Region	Model Variables	Diagnostic Target	Gold Standard Type	AI versus clinicians
							features	WT)		
Zhou et al. [20], 2023	Random sample split validation	Yes	CNN–Transformer	DL	Yes	Intracranial	DL features	EGFR mutation status	Genetic testing	No
Cao et al. [21], 2024	5-fold cross-validation	Yes	ResNet + multiscale fusion	DL	Yes	Intracranial	DL features	EGFR mutation status	Genetic testing	No
Fan et al. [22], 2023	Random sample split validation	Yes	LASSO+LR	ML	Yes	brain–tumor interface (BTI)	Radiomics features + VPE volume	EGFR mutation, T790M resistance mutation	Genetic testing	No
Ahn et al. [23], 2020	10-fold cross-validation	No	Random forest, support vector machine, AdaBoost, LASSO +LR	ML	Yes	Intracranial	Radiomics features	EGFR mutation status	Genetic testing	No
Ouyang et al.	Random split	Yes	SVM, SGD,	ML	Yes	Primary	Radiomic	EGFR mutation	Genetic testing	No

First Author (Ref), Year	Internal Validation Type	External Validation	Modeling Algorithm	ML/DL	Manual	ROI Region	Model Variables	Diagnostic Target	Gold Standard Type	AI versus clinicians
[24], 2025	validation		KNN, Random Forest, XGBoost, LightGBM			pulmonary lesion and Intracranial of BM	features, clinical features	status		
Sui et al. [25], 2023	5-fold cross-validation	No	ResNet18	DL	Yes	Intracranial	DL features	Differentiate SCLC and NSCLC	Histopathology	No
Cao et al. [26], 2025	Random split validation	Yes	LR + ResNet34	DL + ML	Yes	Intracranial	Radiomic features, DL features	EGFR mutation status	Genetic testing	No
Li et al. [27], 2016	K-fold cross-validation	No	KNN, BP-ANN	ML, DL	Yes	Intracranial	Radiomic features, DL features	Histological subtype classification	Histopathology	No
Li et al. [28], 2022	10-fold cross-validation	No	Random forest	ML	Yes	Intracranial	Radiomic features	Distinguish EGFR vs. ALK mutation status	Genetic testing	No
Li et al. [29], 2024	Random split validation	Yes	ARFN (ResNet-	ML, DL	Yes	Intracranial	Radiomic features, DL	Differentiate SCLC and	Histopathology	No

First Author (Ref), Year	Internal Validation Type	External Validation	Modeling Algorithm	ML/DL	Manual	ROI Region	Model Variables	Diagnostic Target	Gold Standard Type	AI versus clinicians
			18), LR				features	NSCLC		
Li et al. [30], 2024	Random split validation	No	SVC + LASSO	ML	Yes	Only includes solid tumor areas	Radiomic features	Predict EGFR mutation and HER2 overexpression status	EGFR: based on NGS testing; HER2: determined by IHC and FISH	No
Fan et al. [31], 2023	Stratified random sampling + 10-fold cross-validation	Yes	LASSO+LR	ML	Yes	Solid tumor area and peritumoral edema (excluding necrosis)	Radiomic features	EGFR mutation status	Genetic testing	No
Tulum et al. [32], 2023	10-fold cross-validation	No	Radiomics, MLP, SWMDeep Learning; ResNet-50, EfficientNet-b0	ML + DL	Semi-automatic segmentation; (automatic boundary optimization)	Intracranial	Radiomic features, DL features	Differentiate SCLC and NSCLC	Histopathology	No
Zhu et al. [33],	8:2 random	No	MLP, SVM,	DL +	Yes	Solid tumor	Radiomic	Differentiate	Histopathology	No

First Author (Ref), Year	Internal Validation Type	External Validation	Modeling Algorithm	ML/DL	Manual	ROI Region	Model Variables	Diagnostic Target	Gold Standard Type	AI versus clinicians
2023	split + 5-fold cross-validation		RF, LR	ML		enhancement region	features, DL features	spinal granuloma vs. metastasis		
Hu et al. [34],2024	K-fold cross-validation	Yes	LASSO, SVM, Random Forest	ML	Yes	BM: TAA (enhancing area) + POA (peritumoral edema); SM: whole lesion	Radiomic features	EGFR mutation status	Genetic testing	No
Artzi et al. [35], 2019	8:2 random split + 5-fold cross-validation	No	SVM	ML	Semi-automatic segmentation (threshold + region growing) with manual expert	Contrast-enhancing tumor region (mid-three axial slices within bounding box)	Radiomic features,clinical features	Differentiate glioblastoma vs. brain metastasis (and metastatic subtypes)	Histopathology	No

First Author (Ref), Year	Internal Validation Type	External Validation	Modeling Algorithm	ML/DL	Manual	ROI Region	Model Variables	Diagnostic Target	Gold Standard Type	AI versus clinicians
					correction					
Han et al. [36],2021	5-fold cross-validation	Yes	LR, SVM, Decision Tree, Random Forest	ML	Yes	Intracranial	Radiomic features, clinical features	differentiation of MET-lung and MET-other	Histopathological	No
Chen et al. [37], 2019	Random split (training/testing = 4:1) with 100 repeated validations	No	Distance correlation + LR	ML	Yes	Entire enhancing tumor	Radiomic features	Differentiate glioblastoma (GBM) from metastatic brain tumors	Histopathological	No
Huang et al. [38],2022	5-fold cross-validation	No	LASSO + LR	ML	Yes	Intracranial	Radiomic features	Differentiate glioblastoma (GBM) from solitary brain metastases (SBM)	Histopathological	No
Duan et al. [39],2023	5-fold cross-validation	Yes	ResNet-18, ResNet-50, EfficientNet-B3	DL	semi-automatic lesion bounding	Intracranial	DL features	Differentiate spinal granuloma vs. metastasis	Histopathology	Yes

First Author (Ref), Year	Internal Validation Type	External Validation	Modeling Algorithm	ML/DL	Manual	ROI Region	Model Variables	Diagnostic Target	Gold Standard Type	AI versus clinicians
					box					
Lang et al. [40],2019	10-fold cross-validation	Yes	CNN and CLSTM/DAR	DL + ML	Yes	Whole tumor 3D ROI	Radiomic features, DL features	Determine whether spinal metastasis originated from lung cancer	Histopathology	No
Cheng et al. [41], 2023	10-fold Cross-validation	No	LASSO + LR	ML	Yes	Origin site (tumor + 5mm margin) + metastasis	Radiomic features	Predict EGFR mutation status	Genetic Testing	No
Cao et al. [42], 2022	10-fold Cross-validation	No	LASSO + LR	ML	Yes	Solid tumor enhancement region	Radiomic features	Prediction of EGFR mutation subtypes (exon 19 vs. exon 21)	Genetic Testing	No
Cao et al. [43], 2023	5-fold Cross-validation	Yes	LASSO + LR	ML	Yes	Intracranial	Radiomic features, clinical features	Prediction of EGFR mutation subtypes (exon 19 vs. exon 21)	Genetic Testing	No
Fan et al. [44],	10-fold Cross-	No	LASSO +	ML	Yes	Intracranial	Radiomic	Predict EGFR	Genetic	No

First Author (Ref), Year	Internal Validation Type	External Validation	Modeling Algorithm	ML/DL	Manual	ROI Region	Model Variables	Diagnostic Target	Gold Standard Type	AI versus clinicians
2021	validation		LR				features, clinical features	mutation status	sequencing results of primary lung tumor (NGS)	
Fan et al. [45], 2022	10-fold Cross-validation	Yes	LASSO + LR	ML	Yes	Intracranial	Radiomic features, clinical features	EGFR mutation, T790M resistance mutation	Primary tumor tissue NGS + post-treatment ctDNA	No
Fan et al. [46], 2022	5-fold Cross-validation	Yes	LASSO + LR	ML	Yes	Intracranial	Radiomic features, clinical features	Predict EGFR mutation and subtypes (exon 19, exon 21)	Pathological confirmation of primary tumor + NGS genetic sequencing results	No
Jiang et al. [47], 2021	10-fold Cross-validation	No	VGG16 (CNN) + LR	ML + DL	Yes	Intracranial and peritumoral region	Radiomic features, clinical features	Prediction of EGFR mutation status	Pathological confirmation of primary lung tumor + EGFR gene sequencing	No
Jiang et al. [48], 2023	Stratified random sampling	Yes	CM-EfNet	ML	Yes	Intracranial	DL features	Predict EGFR mutation status and subtypes (19	Pathological confirmation of primary tumor +	Yes

First Author (Ref), Year	Internal Validation Type	External Validation	Modeling Algorithm	ML/DL	Manual	ROI Region	Model Variables	Diagnostic Target	Gold Standard Type	AI versus clinicians
								vs. 21)	EGFR gene testing (NGS)	
Ren et al. [49], 2021	10-fold Cross-validation	Yes	LASSO + LR	ML	Yes	Intracranial and peritumoral region	Radiomic features	Prediction of EGFR mutation status	EGFR gene testing (NGS)	No
Wang et al. [50], 2024	10-fold Cross-validation	No	LASSO + LR	ML	Yes	Intracranial	Radiomic features, clinical features	Differentiation between bone metastasis and benign lesions	Histopathology	No
Hou et al. [51], 2024	Random Sample Validation	Yes	LASSO + LR	ML	Yes	Tumor-liver interface	Radiomic features	Predict EGFR mutation status	Genetic Testing	No
Hou et al. [52],2023	Random Sample Validation	No	LASSO + LR	ML	Yes	Tumor-liver interface and origin site	Radiomic features, clinical features	Predict EGFR mutation status	Genetic Testing	No

* ROI Region were categorized as intratumoral (within the tumor) and peritumoral (surrounding the tumor) according to the original study descriptions. Peritumoral regions were operationalized as either (1) a fixed-margin expansion beyond the tumor boundary (in millimeters) or (2) edema-based regions adjacent to the tumor (peritumoral edema). Both were treated as peritumoral regions in this meta-analysis.

Supplementary Table 4. Validation methods and algorithms for Outcome prediction studies (n = 13)

First Author (Ref), Year	Internal Validation Type	External Validation	Modeling Algorithm	ML/DL	Manual	ROI Region	Model Variables	Treatment Strategy	Follow-up Duration	Clinical Outcome	AI versus clinicians
Qi et al. [53], 2024	Random sample split validation	No	LASSO+LR	ML	Yes	Intracranial	Radiomic features, clinical variables	EGFR-TKI targeted therapy	15.67 months	iPFS	No
Xu et al. [54], 2024	5-fold cross-validation	Yes	LASSO+LR	ML	Yes	Intracranial and peritumoral region	Radiomic features, clinical variables	Immune checkpoint inhibitors	NR	iPFS	No
Zhao et al. [55], 2021	Random sample split validation	No	mRMR+LASSO	ML	Yes	Intracranial	Radiomic features, clinical variables	Ensartinib treatment	51 weeks	iPFS	No
Qu et al. [56], 2024	5-fold cross-validation	No	LASSO+LR	ML	Yes	Intracranial	Radiomic features, clinical variables	EGFR-TKI targeted therapy	12 months	1-year IP incidence	No
Qi et al. [57], 2024	10-fold cross-validation	No	LASSO+LR	ML	Yes	Intracranial	Radiomic features, clinical	EGFR-TKI targeted therapy	12.67 months	iPFS and PR + CR	No

First Author (Ref), Year	Internal Validation Type	External Validation	Modeling Algorithm	ML/DL	Manual	ROI Region	Model Variables	Treatment Strategy	Follow-up Duration	Clinical Outcome	AI versus clinicians
							variables				
Park et al. [58], 2021	Bootstrap internal validation	No	LASSO+LR	ML	Yes	Solid tumor regions (excluding necrotic, hemorrhagic, and cystic components)	Radiomic features, clinical variables	SRS	Median PFS 18 months; Median OS 26.8 months	PFS, OS	No
Fan et al. [31], 2023	Stratified random sampling + 10-fold cross-validation	Yes	LASSO+LR	ML	Yes	Tumor Active Area and Peritumoral Edema Area	Radiomic features	EGFR-TKI targeted therapy	NR	EGFR-TKI response status	No
Lv et al. [59], 2023	10-fold cross-validation	Yes	Random Forest	ML	Yes	BM boundary region	Radiomic features	EGFR-TKI targeted therapy	14 months	T790M resistance mutation status	No

First Author (Ref), Year	Internal Validation Type	External Validation	Modeling Algorithm	ML/DL	Manual	ROI Region	Model Variables	Treatment Strategy	Follow-up Duration	Clinical Outcome	AI versus clinicians
Liao et al. [60], 2021	Stratified random sampling	No	SVM	ML	Yes	Intracranial	Radiomic features, clinical variables	GKRS	Median OS 12.2 months	Local Tumor Control and OS	No
Sun et al. [61], 2024	Stratified random sampling	No	LASSO+LR	ML	Yes	Intracranial	Radiomic features	WBRT	1.5–2 months	CR/PR = responsive group; SD/PD = non-responsive group	No
Wang et al. [62], 2024	5-fold cross-validation	No	CoxPH, SVM, Tree-based models, and Deep learning (CoxCC)	ML, DL	Yes	BM(Intracranial) + primary site	Radiomic features, clinical variables	EGFR-TKI targeted therapy	10.4 months	PFS	No
Chen et al.	LOOCV	No	Gradient	ML	Yes	Intracranial	Radiomic	Standard	12.7 (as	PFS, OS	No

First Author (Ref), Year	Internal Validation Type	External Validation	Modeling Algorithm	ML/DL	Manual	ROI Region	Model Variables	Treatment Strategy	Follow-up Duration	Clinical Outcome	AI versus clinicians
[63], 2021			Boosting Classifier				features, clinical variables	treatment	reported)		
Gutsche et al. [64], 2022	Stratified random sampling + 5-fold cross-validation	No	Random Forest	ML, DL	No, 3D-CNN	Intracranial	Radiomic features, semantic features	SRS	≥180 days	Response vs. non-response	No

* ROI Region were categorized as intratumoral (within the tumor) and peritumoral (surrounding the tumor) according to the original study descriptions. Peritumoral regions were operationalized as either (1) a fixed-margin expansion beyond the tumor boundary (in millimeters) or (2) edema-based regions adjacent to the tumor (peritumoral edema). Both were treated as peritumoral regions in this meta-analysis.

Supplementary Table 5. Contingency tables for AI diagnostic algorithms extracted from included studies (341 tables from 52 studies)

First Author (Ref), Year	Total	TP	FP	TN	FN	Sensitivity	Specificity	AUROC
Grossman et al. [1], 2021	69	NR	NR	NR	NR	NR	NR	NR
Park et al. [2], 2021	42	11	9	17	5	0.688	0.667	0.649
	42	9	5	21	7	0.563	0.808	0.692
	42	13	6	20	3	0.813	0.769	0.726
	42	13	10	16	3	0.813	0.615	0.765
Sun et al. [3], 2024	32	17	3	7	5	0.719	0.573	0.818
	32	20	2	6	4	0.844	0.709	0.818
	32	17	5	7	3	0.719	0.406	0.479
	32	21	2	6	3	0.875	0.711	0.906
	32	20	3	5	4	0.813	0.604	0.760
	32	20	4	4	4	0.813	0.438	0.784
	32	20	2	6	4	0.813	0.688	0.859
	32	20	4	4	4	0.844	0.531	0.875
Wang et al. [4], 2021	37	12	2	13	10	0.564	0.885	0.734
	42	22	0	19	0	1.000	0.988	0.988
	42	12	4	16	10	0.541	0.822	0.780

First Author (Ref), Year	Total	TP	FP	TN	FN	Sensitivity	Specificity	AUROC
Wu et al. [5], 2024	42	11	3	17	11	0.490	0.854	0.679
	9	2	0	4	3	0.457	0.897	0.730
	10	6	0	4	0	1.000	0.980	0.987
	10	4	1	3	2	0.667	0.743	0.675
	10	3	0	4	3	0.478	0.877	0.633
	125	9	12	95	9	0.500	0.888	0.540
	125	12	6	101	6	0.660	0.944	0.720
	125	13	5	102	5	0.710	0.953	0.710
	125	13	5	102	5	0.700	0.953	0.700
	125	9	8	99	9	0.520	0.925	0.580
	125	10	7	100	8	0.580	0.935	0.650
	125	11	6	101	7	0.630	0.944	0.700
	125	10	6	101	8	0.580	0.944	0.630
	125	9	12	95	9	0.430	0.888	0.350
	125	10	8	99	8	0.540	0.925	0.460
	125	10	9	98	8	0.530	0.916	0.530
	125	15	2	105	3	0.860	0.981	0.870

First Author (Ref), Year	Total	TP	FP	TN	FN	Sensitivity	Specificity	AUROC
	125	12	3	104	6	0.690	0.972	0.750
	125	9	8	99	9	0.520	0.925	0.630
	125	10	10	97	8	0.540	0.907	0.380
	125	9	12	95	9	0.500	0.888	0.460
	125	12	5	102	6	0.670	0.953	0.730
	125	13	5	102	5	0.720	0.953	0.730
	125	11	7	100	7	0.610	0.935	0.670
	125	9	8	99	9	0.530	0.925	0.610
	125	11	7	100	7	0.590	0.935	0.660
	125	11	6	101	7	0.650	0.944	0.710
	125	10	6	101	8	0.540	0.944	0.640
	125	6	9	98	12	0.370	0.916	0.430
	125	10	8	99	8	0.570	0.925	0.490
	125	11	8	99	7	0.500	0.925	0.470
	125	16	2	105	2	0.880	0.981	0.850
	125	11	4	103	7	0.600	0.963	0.710
	125	10	7	100	8	0.560	0.935	0.660

First Author (Ref), Year	Total	TP	FP	TN	FN	Sensitivity	Specificity	AUROC
	125	10	9	98	8	0.530	0.916	0.420
	125	9	11	96	9	0.500	0.897	0.600
	125	17	7	100	1	0.950	0.935	0.990
	125	16	7	100	2	0.900	0.935	0.910
	125	16	2	105	2	0.900	0.981	0.910
	125	16	4	103	2	0.860	0.963	0.930
	125	16	8	99	2	0.870	0.925	0.930
	125	15	4	103	3	0.880	0.963	0.930
	125	16	4	103	2	0.880	0.963	0.930
	125	9	11	96	9	0.520	0.897	0.510
	125	11	7	100	7	0.590	0.935	0.490
	125	10	6	101	8	0.570	0.944	0.720
	125	16	2	105	2	0.880	0.981	0.850
	125	16	2	105	2	0.900	0.981	0.980
	125	16	4	103	2	0.880	0.963	0.950
	125	10	8	99	8	0.550	0.925	0.540
	125	10	5	102	8	0.570	0.953	0.620

First Author (Ref), Year	Total	TP	FP	TN	FN	Sensitivity	Specificity	AUROC
	125	17	1	106	1	0.930	0.991	0.960
	125	16	2	105	2	0.890	0.981	0.910
	125	16	1	106	2	0.910	0.991	0.920
	125	15	3	104	3	0.850	0.972	0.920
	125	16	3	104	2	0.840	0.972	0.880
	125	15	3	104	3	0.840	0.972	0.900
	125	16	3	104	2	0.850	0.972	0.890
	125	10	5	102	8	0.580	0.953	0.470
	125	15	3	104	3	0.660	0.972	0.660
	125	16	2	105	2	0.580	0.981	0.610
	125	17	1	106	1	0.870	0.991	0.860
	125	16	2	105	2	0.910	0.981	0.940
	125	16	2	105	2	0.870	0.981	0.940
	125	11	7	100	7	0.620	0.935	0.580
Xu et al. [6], 2024	182	69	10	78	25	0.739	0.883	0.895
	46	14	4	18	10	0.591	0.833	0.759
Yang et al. [7], 2024	113	49	6	52	6	0.891	0.897	0.955

First Author (Ref), Year	Total	TP	FP	TN	FN	Sensitivity	Specificity	AUROC
	55	22	3	26	4	0.846	0.893	0.946
	55	18	1	32	4	0.818	0.970	0.928
	57	24	5	24	4	0.857	0.828	0.879
	28	13	4	10	1	0.929	0.733	0.819
	28	8	2	15	3	0.727	0.882	0.882
	62	28	4	21	9	0.760	0.840	0.830
	37	15	4	15	3	0.818	0.769	0.825
	37	12	6	18	1	0.889	0.733	0.822
	61	30	4	20	7	0.810	0.833	0.812
	37	15	4	17	1	0.909	0.818	0.818
	37	14	6	15	2	0.889	0.733	0.800
	95	48	8	20	19	0.713	0.716	0.765
Zhang et al. [8], 2021	95	48	8	20	19	0.714	0.731	0.769
	95	40	4	27	24	0.594	0.857	0.795
	95	37	2	30	26	0.558	0.923	0.828
Zheng et al. [9],2021	108	36	17	48	7	0.837	0.738	0.850
	54	20	6	21	7	0.731	0.786	0.810

First Author (Ref), Year	Total	TP	FP	TN	FN	Sensitivity	Specificity	AUROC
Cao et al. [10], 2022	100	47	2	46	5	0.959	0.902	0.968
	50	20	4	24	2	0.833	0.923	0.901
	38	17	2	17	2	0.895	0.895	0.900
Chen et al. [11], 2020	110	54	21	31	6	0.731	0.906	0.912
	75	15	6	47	7	0.761	0.887	0.915
	75	13	2	58	2	0.893	0.977	0.985
Fan et al. [12], 2022	153	79	11	59	4	0.878	0.937	0.945
	77	32	13	31	1	0.733	0.969	0.880
	80	35	5	32	8	0.875	0.800	0.895
	89	32	14	38	5	0.717	0.884	0.869
	46	16	8	17	5	0.708	0.818	0.786
	40	15	5	16	4	0.750	0.800	0.802
Fan et al. [13], 2023	53	16	5	31	1	0.762	0.969	0.938
	27	8	3	14	2	0.818	0.875	0.864
	30	10	3	14	3	0.846	0.824	0.860
Haim et al. [14], 2022	59	10	6	42	1	0.687	0.977	0.910
Huang et al. [15], 2024	86	40	5	25	16	0.889	0.634	0.762

First Author (Ref), Year	Total	TP	FP	TN	FN	Sensitivity	Specificity	AUROC
Li et al. [16], 2023	37	17	2	10	8	0.895	0.556	0.725
	176	54	14	84	24	0.800	0.785	0.886
	57	17	10	25	5	0.650	0.843	0.850
Meißner et al. [17], 2023	36	14	4	14	4	0.800	0.810	0.830
	17	7	0	7	3	1.000	0.700	0.840
Zhu et al. [18], 2024	175	81	13	75	6	0.870	0.940	0.950
	175	70	24	63	18	0.750	0.780	0.810
	75	17	9	44	5	0.680	0.910	0.870
	75	20	6	41	8	0.770	0.850	0.840
Li et al. [19], 2024	306	78	0	186	42	1.000	0.820	1.000
	306	85	37	184	0	0.700	1.000	0.990
	306	88	19	187	12	0.830	0.940	0.970
	306	78	0	188	30	1.000	0.870	1.000
	306	89	33	184	0	0.730	1.000	1.000
	306	98	9	187	12	0.920	0.940	0.990
Zhou et al. [20], 2023	188	59	12	109	8	0.840	0.940	0.900
Cao et al. [21], 2024	160	57	18	67	18	0.760	0.790	0.720

First Author (Ref), Year	Total	TP	FP	TN	FN	Sensitivity	Specificity	AUROC
Fan et al. [22], 2023	160	63	12	68	17	0.840	0.810	0.780
	160	64	11	69	16	0.860	0.820	0.820
	160	67	8	73	12	0.900	0.870	0.910
	154	83	7	46	18	0.930	0.730	0.880
	154	73	17	44	20	0.820	0.700	0.790
	154	70	20	42	22	0.780	0.670	0.760
	154	88	2	43	21	0.980	0.330	0.660
	154	72	18	48	16	0.810	0.750	0.820
	154	72	18	37	27	0.810	0.590	0.740
Ahn et al. [23], 2020	61	25	4	32	0	0.870	1.000	0.870
	61	25	4	32	0	0.890	1.000	0.890
	61	25	4	32	0	0.880	1.000	0.870
	61	18	11	22	10	0.650	0.710	0.640
	61	18	11	28	4	0.630	0.890	0.760
	61	18	11	29	3	0.630	0.930	0.780
	61	20	9	26	6	0.700	0.830	0.760
	61	16	13	29	3	0.580	0.930	0.580

First Author (Ref), Year	Total	TP	FP	TN	FN	Sensitivity	Specificity	AUROC
Ouyang et al. [24], 2025	185	45	23	58	59	0.670	0.500	0.770
	185	51	17	79	38	0.750	0.680	0.890
	185	40	28	40	77	0.590	0.350	0.660
	185	44	24	56	61	0.660	0.480	0.630
	185	45	23	62	55	0.670	0.530	0.770
	185	43	25	52	65	0.640	0.450	0.640
	185	46	22	65	52	0.690	0.560	0.790
	185	51	17	78	39	0.750	0.670	0.800
	185	53	15	90	27	0.780	0.770	0.870
	185	46	22	63	54	0.680	0.540	0.750
Sui et al. [25], 2023	192	51	21	85	35	0.710	0.710	0.720
	192	54	18	91	29	0.760	0.760	0.760
	192	53	17	92	28	0.760	0.760	0.760
	192	58	14	97	23	0.810	0.810	0.810
	192	51	21	85	35	0.710	0.710	0.710
Cao et al. [26], 2025	216	73	9	117	17	0.900	0.880	0.930
	216	77	5	123	11	0.940	0.920	0.950

First Author (Ref), Year	Total	TP	FP	TN	FN	Sensitivity	Specificity	AUROC
	72	24	3	40	5	0.890	0.890	0.890
	72	24	3	40	5	0.910	0.900	0.940
	126	47	3	69	7	0.940	0.920	0.880
	126	45	5	67	9	0.900	0.890	0.820
	126	47	3	70	6	0.950	0.930	0.880
Li et al. [27], 2016	126	46	4	71	5	0.950	0.930	0.870
	126	46	4	74	2	0.930	0.980	0.940
	126	48	2	72	4	0.960	0.960	0.820
	126	46	4	70	6	0.920	0.930	0.880
	126	45	5	68	8	0.910	0.900	0.820
Li et al. [28], 2022	NR	NR	NR	NR	NR	NR	NR	NR
	30	11	4	11	4	0.770	0.780	0.790
	30	11	4	10	5	0.770	0.670	0.760
Li et al. [29], 2024	30	9	6	13	2	0.650	0.900	0.800
	30	8	7	13	2	0.580	0.930	0.770
	30	13	2	8	7	0.870	0.560	0.760
	30	10	5	13	2	0.670	0.890	0.800

First Author (Ref), Year	Total	TP	FP	TN	FN	Sensitivity	Specificity	AUROC
	90	17	4	23	11	0.390	0.790	0.570
	90	26	6	21	2	0.810	0.320	0.520
	90	18	2	25	10	0.800	0.340	0.550
	90	25	3	24	3	0.500	0.720	0.590
	90	27	5	22	1	0.470	0.660	0.540
	90	17	5	22	11	0.760	0.680	0.750
	90	39	4	43	4	0.920	0.930	0.980
Li et al. [30], 2024	90	35	8	31	16	0.830	0.670	0.860
	39	13	5	14	7	0.730	0.690	0.820
	39	10	8	14	7	0.570	0.670	0.700
	153	63	23	55	12	0.740	0.830	0.820
	153	58	28	60	7	0.680	0.910	0.850
	153	70	16	56	11	0.820	0.840	0.900
Fan et al. [31], 2023	77	32	11	26	8	0.760	0.780	0.820
	77	27	16	30	4	0.640	0.910	0.800
	77	28	15	32	2	0.670	0.970	0.860
	80	32	8	28	12	0.800	0.700	0.820

First Author (Ref), Year	Total	TP	FP	TN	FN	Sensitivity	Specificity	AUROC
	80	29	11	32	8	0.730	0.800	0.790
	80	32	8	34	6	0.800	0.850	0.900
	89	28	22	21	18	0.560	0.560	0.740
	89	31	19	24	15	0.630	0.630	0.760
	89	32	18	25	14	0.650	0.650	0.820
	46	17	8	16	5	0.710	0.770	0.720
	46	16	9	17	4	0.650	0.820	0.720
	46	20	5	15	6	0.830	0.730	0.790
	40	16	4	12	8	0.800	0.600	0.730
	40	9	11	19	1	0.450	0.950	0.720
	40	13	7	17	3	0.650	0.850	0.810
Tulum et al. [32], 2023	222	64	11	138	9	0.880	0.930	0.940
	222	68	7	143	4	0.940	0.950	0.990
	222	66	9	143	4	0.940	0.940	0.970
	222	65	10	142	5	0.930	0.930	0.970
Zhu et al. [33], 2023	194	56	0	133	5	1.000	0.970	1.000
	194	54	2	133	5	0.980	0.970	0.990

First Author (Ref), Year	Total	TP	FP	TN	FN	Sensitivity	Specificity	AUROC
	194	56	0	133	5	1.000	0.970	1.000
	194	56	0	133	5	1.000	0.970	1.000
	49	13	1	34	1	0.980	0.980	1.000
	49	12	2	34	1	0.960	0.980	0.990
	49	14	0	31	4	1.000	0.900	1.000
	49	13	1	31	4	0.980	0.900	0.990
	165	33	0	128	4	1.000	0.970	0.990
	165	32	1	128	4	0.970	0.970	1.000
	165	32	1	132	0	0.970	1.000	0.990
	165	32	1	108	24	0.970	0.820	0.950
	41	8	0	31	2	1.000	0.960	1.000
	41	8	0	32	1	1.000	0.980	1.000
	41	7	1	32	1	0.970	0.970	1.000
	41	7	1	28	5	0.980	0.860	0.980
	84	56	0	26	2	1.000	0.930	1.000
	84	56	0	26	2	1.000	0.930	1.000
	84	52	4	26	2	0.930	0.930	1.000

First Author (Ref), Year	Total	TP	FP	TN	FN	Sensitivity	Specificity	AUROC
	84	56	0	26	2	1.000	0.930	1.000
	21	12	2	7	0	0.920	1.000	0.990
	21	12	2	7	0	0.900	1.000	1.000
	21	11	3	7	0	0.820	1.000	1.000
	21	12	2	6	1	0.880	0.940	0.970
	167	53	3	111	0	0.960	1.000	1.000
	167	54	2	106	5	0.980	0.960	1.000
	167	54	2	106	5	0.980	0.960	0.980
	167	53	3	101	10	0.960	0.910	0.990
	42	13	1	26	2	0.980	0.960	0.990
	42	13	1	25	3	0.980	0.940	0.990
	42	13	1	25	3	0.980	0.940	0.990
	42	12	2	25	3	0.940	0.940	0.980
Hu et al. [34],2024	95	30	5	47	18	0.630	0.900	NR
	95	41	7	45	7	0.850	0.870	NR
	95	36	8	44	12	0.750	0.850	NR
	95	34	12	40	14	0.710	0.770	NR

First Author (Ref), Year	Total	TP	FP	TN	FN	Sensitivity	Specificity	AUROC
	95	31	15	37	17	0.650	0.710	NR
	41	38	13	39	10	0.790	0.750	NR
	41	39	13	39	9	0.810	0.750	NR
	41	44	33	19	4	0.920	0.360	NR
	41	37	11	41	11	0.770	0.790	NR
	41	35	4	48	13	0.730	0.920	NR
	30	36	9	43	12	0.750	0.830	NR
	30	36	15	37	12	0.750	0.710	NR
Artzi et al. [35], 2019	182	51	40	79	12	0.570	0.870	0.830
	45	13	9	20	3	0.620	0.880	0.860
	92	43	7	25	17	0.875	0.614	0.770
Han et al. [36],2021	46	19	6	14	7	0.793	0.706	0.759
	30	10	5	10	5	0.667	0.667	0.741
Chen et al. [37], 2019	107	47	13	40	7	0.790	0.870	0.830
	27	10	5	10	2	0.710	0.850	0.800
Huang et al. [38],2022	48	21	3	23	1	0.950	0.960	0.992
Duan et al. [39],2023	18	5	3	7	3	0.810	0.810	0.980

First Author (Ref), Year	Total	TP	FP	TN	FN	Sensitivity	Specificity	AUROC
	18	6	2	8	2	0.850	0.850	0.990
	18	7	1	9	1	0.960	0.960	0.900
	18	8	0	9	1	0.990	0.980	0.850
	29	9	3	12	5	0.820	0.740	0.950
	29	10	2	12	5	0.840	0.760	0.910
	29	10	2	13	4	0.910	0.800	0.870
	29	11	1	14	3	0.960	0.880	0.860
Lang et al. [40],2019	61	18	12	30	1	0.600	0.970	NR
	135	55	17	46	17	0.770	0.740	0.800
	135	61	11	54	9	0.850	0.870	0.900
	135	62	10	48	15	0.870	0.770	0.930
	68	26	10	24	8	0.730	0.770	0.700
Cheng et al. [41], 2023	68	23	11	28	6	0.680	0.840	0.780
	68	21	13	30	4	0.620	0.900	0.810
	73	27	4	26	16	0.880	0.630	0.800
	73	24	7	31	11	0.780	0.760	0.810
	73	28	3	31	11	0.910	0.760	0.880

First Author (Ref), Year	Total	TP	FP	TN	FN	Sensitivity	Specificity	AUROC
Cao et al. [42], 2022	37	11	4	17	5	0.750	0.810	0.760
	37	13	2	14	8	0.880	0.670	0.710
	37	9	6	20	2	0.630	0.910	0.800
	50	23	2	20	5	0.920	0.800	0.901
	26	11	2	11	2	0.846	0.846	0.882
	171	76	15	54	26	0.846	0.675	0.829
Cao et al. [43], 2023	91	29	11	45	6	0.725	0.900	0.885
	91	36	7	44	4	0.854	0.837	0.919
	86	36	9	26	15	0.804	0.650	0.760
	46	16	4	16	10	0.808	0.650	0.777
	46	15	7	22	2	0.708	0.955	0.811
	42	16	8	13	5	0.673	0.754	0.780
	24	8	3	9	4	0.795	0.744	0.846
	24	9	2	10	3	0.846	0.800	0.818
Fan et al. [44], 2021	62	31	6	21	4	0.838	0.840	0.879
	32	17	2	7	6	0.947	0.615	0.777
Fan et al. [45], 2022	106	36	18	46	6	0.685	0.885	0.849

First Author (Ref), Year	Total	TP	FP	TN	FN	Sensitivity	Specificity	AUROC
	54	18	6	26	4	0.750	0.867	0.842
	54	26	2	17	9	0.946	0.692	0.828
	28	8	4	15	1	0.667	0.938	0.823
	32	10	8	13	1	0.611	0.929	0.778
	18	7	1	8	2	0.875	0.800	0.800
	105	49	20	31	5	0.722	0.870	0.851
	69	26	2	23	18	0.964	0.561	0.816
	69	18	11	35	5	0.655	0.875	0.814
	54	26	9	14	5	0.771	0.737	0.780
Fan et al. [46], 2022	35	11	3	14	7	0.786	0.714	0.789
	35	12	3	14	6	0.800	0.700	0.770
	24	13	4	5	2	0.765	0.857	0.807
	17	4	2	6	5	0.833	0.636	0.742
	17	6	2	8	1	0.750	0.889	0.792
Jiang et al. [47], 2021	77	41	5	22	9	0.913	0.710	0.891
	20	9	3	7	1	0.750	0.875	0.771
Jiang et al. [48], 2023	149	70	16	51	12	0.824	0.825	0.866

First Author (Ref), Year	Total	TP	FP	TN	FN	Sensitivity	Specificity	AUROC
Ren et al. [49], 2021	84	39	13	28	4	0.767	0.906	0.851
	42	21	6	11	4	0.812	0.769	0.764
	110	51	1	38	11	0.839	0.792	0.888
	52	19	10	20	3	0.667	0.909	0.821
Wang et al. [50], 2024	99	43	5	44	7	0.896	0.863	0.939
	42	18	3	17	4	0.900	0.818	0.925
Hou et al. [51], 2024	82	26	6	33	17	0.830	0.670	0.840
	82	28	4	28	22	0.890	0.570	0.790
	41	14	3	14	10	0.830	0.610	0.770
	41	15	2	10	4	0.940	0.440	0.680
	44	9	8	19	5	0.580	0.800	0.730
	44	13	4	13	11	0.800	0.580	0.700
Hou et al. [52],2023	87	24	12	42	9	0.670	0.840	0.830
	87	32	4	36	15	0.890	0.710	0.820
	87	32	4	40	11	0.890	0.800	0.910
	43	13	4	19	7	0.780	0.760	0.760
	43	10	7	23	3	0.610	0.920	0.790

First Author (Ref), Year	Total	TP	FP	TN	FN	Sensitivity	Specificity	AUROC
	43	13	4	24	2	0.780	0.960	0.850

Supplementary Table 6. Contingency tables for AI prognostic algorithms extracted from included studies (62 tables from 13 studies)

First Author (Ref), Year	Total	TP	FP	TN	FN	Sensitivity	Specificity	AUROC
Qi et al. [53], 2024	120	54	6	43	17	0.920	0.730	0.870
	120	50	10	39	21	0.840	0.660	0.840
	120	51	9	55	5	0.850	0.930	0.900
	120	51	9	49	11	0.860	0.820	0.820
	120	46	14	48	12	0.780	0.800	0.750
	120	54	6	43	17	0.910	0.720	0.860
Xu et al. [54], 2024	122	38	20	51	13	0.670	0.810	0.810
	122	47	11	45	19	0.820	0.710	0.800
	122	38	20	47	17	0.670	0.740	0.740
	122	44	14	54	10	0.770	0.850	0.890
	52	10	13	29	0	0.460	1.000	0.760
	52	15	8	21	8	0.680	0.750	0.750
	52	21	2	20	9	0.930	0.700	0.770

First Author (Ref), Year	Total	TP	FP	TN	FN	Sensitivity	Specificity	AUROC
Zhao et al. [55], 2021	52	17	6	22	7	0.780	0.790	0.830
	16	4	1	9	2	0.870	0.870	0.840
	8	1	1	5	1	0.670	0.870	0.850
Qu et al. [56], 2024	70	20	12	25	13	0.640	0.680	0.640
	70	20	12	23	15	0.640	0.620	0.640
	70	22	10	19	19	0.700	0.510	0.740
	70	25	7	29	9	0.810	0.780	0.860
	70	19	13	25	13	0.610	0.670	0.640
	70	21	11	23	15	0.670	0.620	0.640
	70	22	10	23	15	0.710	0.610	0.750
	70	24	8	29	9	0.750	0.780	0.840
Qi et al. [57], 2024	117	NR	NR	NR	NR	NR	NR	NR
Park et al. [58], 2021	83	NR	NR	NR	NR	NR	NR	NR
Fan et al. [31], 2023	153	63	23	55	12	0.740	0.830	0.820
	153	58	28	60	7	0.680	0.910	0.850
	153	70	16	56	11	0.820	0.840	0.900
	77	32	11	26	8	0.760	0.780	0.820
	77	27	16	30	4	0.640	0.910	0.800

First Author (Ref), Year	Total	TP	FP	TN	FN	Sensitivity	Specificity	AUROC
	77	28	15	32	2	0.670	0.970	0.860
	80	32	8	28	12	0.800	0.700	0.820
	80	29	11	32	8	0.730	0.800	0.790
	80	32	8	34	6	0.800	0.850	0.900
	89	28	22	21	18	0.560	0.560	0.740
	89	31	19	24	15	0.630	0.630	0.760
	89	32	18	25	14	0.650	0.650	0.820
	46	17	8	16	5	0.710	0.770	0.720
	46	16	9	17	4	0.650	0.820	0.720
	46	20	5	15	6	0.830	0.730	0.790
	40	16	4	12	8	0.800	0.600	0.730
	40	9	11	19	1	0.450	0.950	0.720
	40	13	7	17	3	0.650	0.850	0.810
Lv et al. [59], 2023	294	81	13	148	52	0.870	0.740	0.880
	294	73	21	180	20	0.780	0.900	0.850
	111	40	14	42	15	0.750	0.740	0.830
	111	30	24	54	3	0.570	0.960	0.730
Liao et al. [60], 2021	93	66	2	13	12	0.850	0.850	0.860

First Author (Ref), Year	Total	TP	FP	TN	FN	Sensitivity	Specificity	AUROC
	93	54	3	24	12	0.690	0.830	0.800
	93	68	1	14	10	0.870	0.910	0.950
	71	24	16	20	11	0.680	0.550	0.640
	71	26	12	24	9	0.730	0.680	0.780
	71	27	7	29	8	0.770	0.810	0.820
Sun et al. [61], 2024	42	27	17	43	12	0.706	0.719	0.776
	11	27	18	45	9	0.750	0.714	0.767
Wang et al. [62], 2024	72	NR	NR	NR	NR	NR	NR	NR
	110	72	3	32	3	0.960	0.940	0.977
Chen et al. [63], 2021	75	18	3	43	11	0.880	0.810	0.905
	75	12	3	49	11	0.830	0.830	0.947
Gutsche et al. [64], 2022	105	36	19	40	10	0.660	0.800	0.750
	45	19	5	12	9	0.810	0.610	0.740

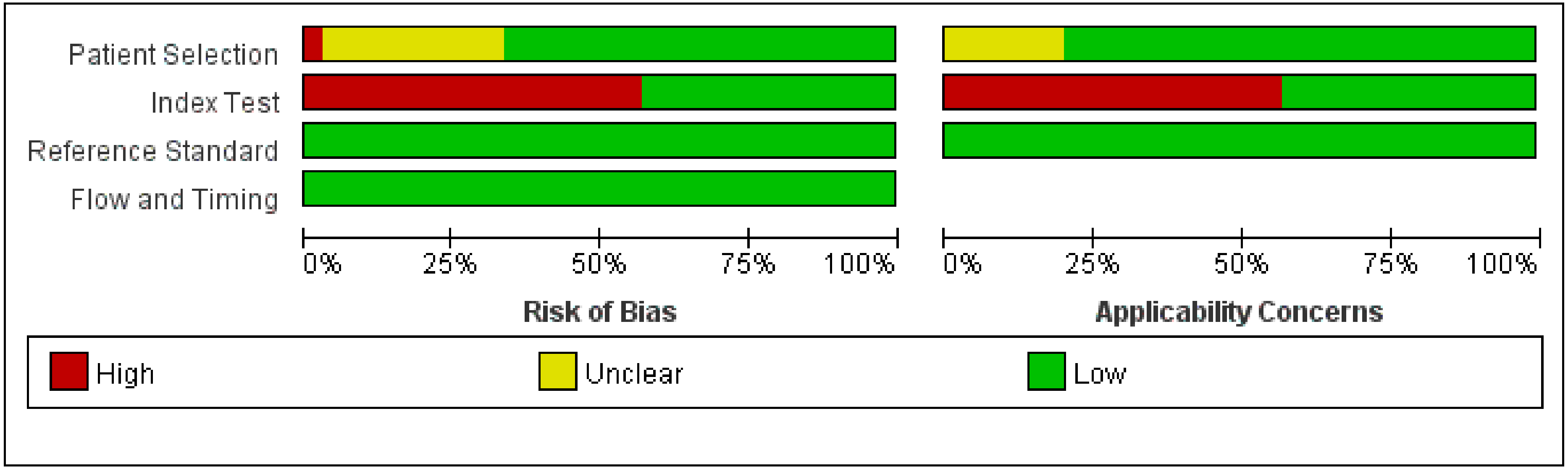

Supplementary Figure 1. QUADAS-AI summary plot

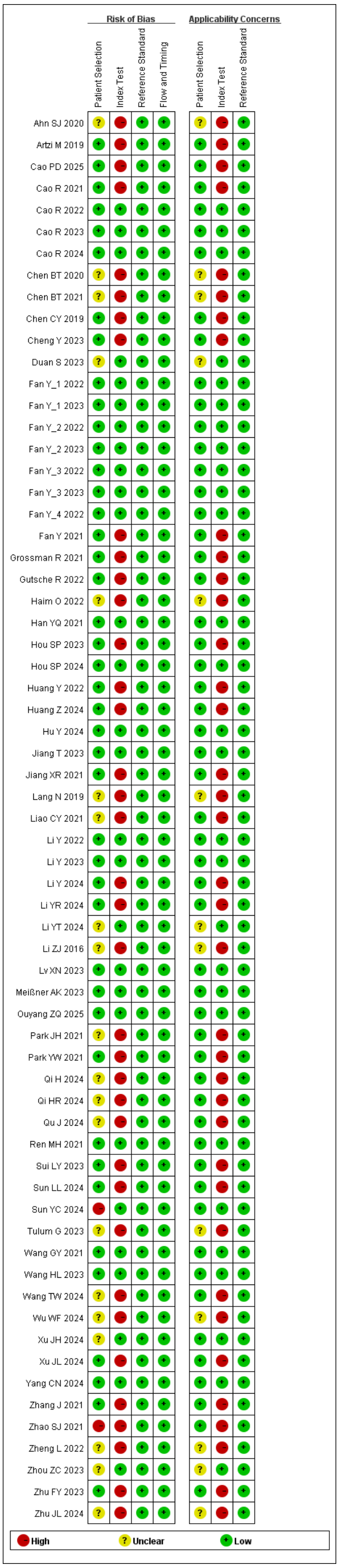

Supplementary Figure 2. Risk of bias and concern of applicability for each item in included studies

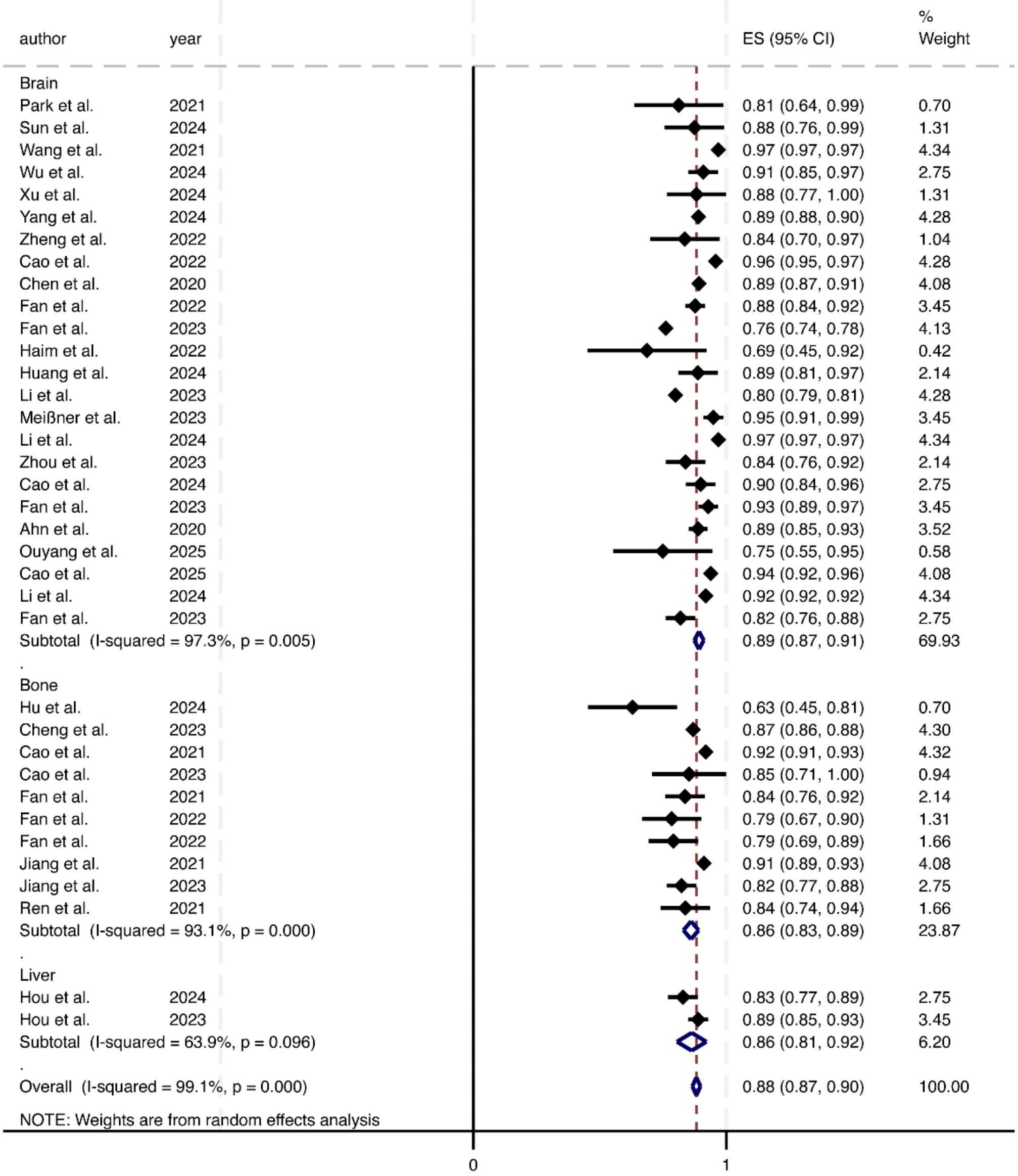

Supplementary Figure 3. Forest plot of pooled Molecular-level prediction performance (sensitivity) stratified by metastatic lesion subgroup.

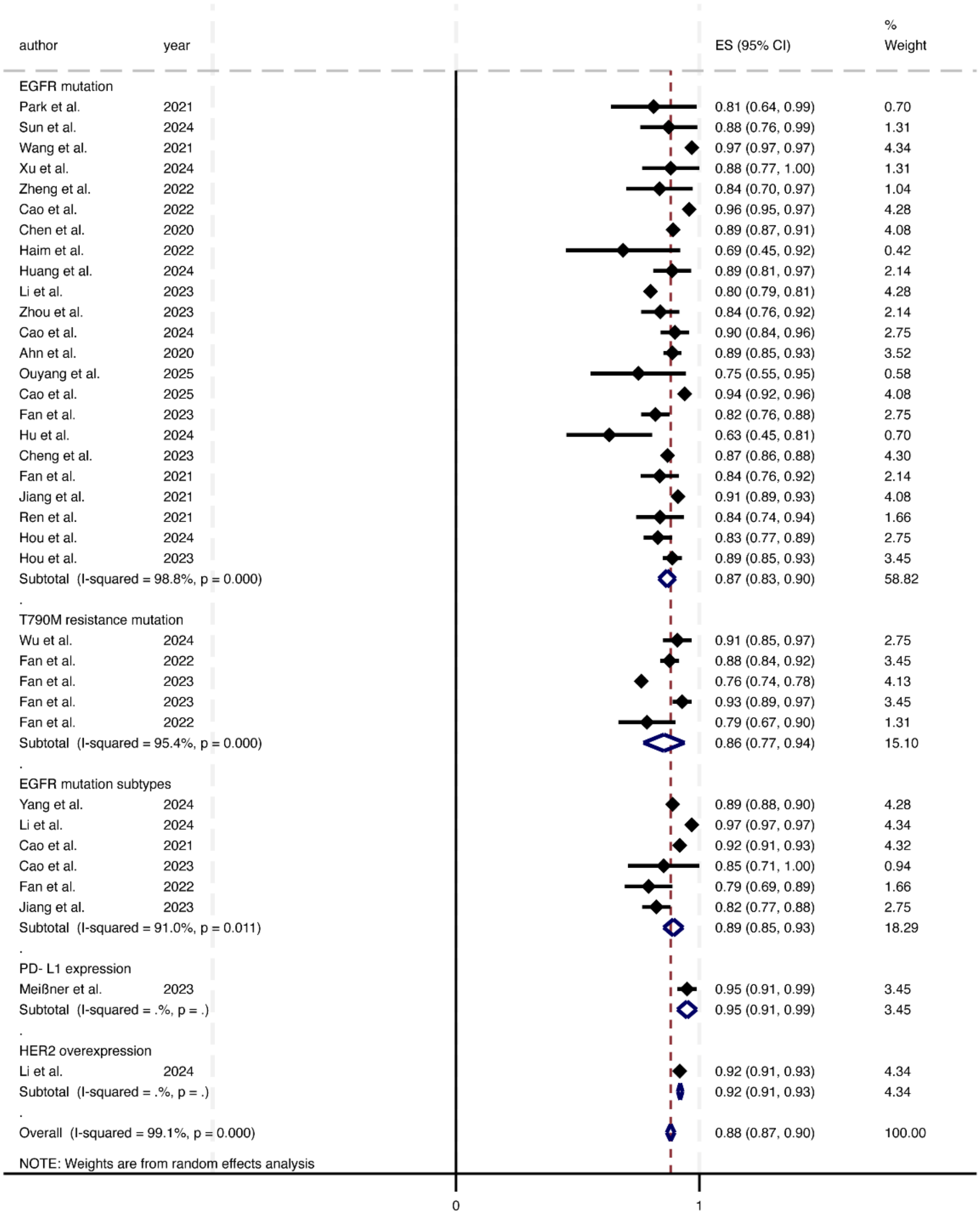

Supplementary Figure 4. Forest plot of pooled Molecular-level prediction performance (sensitivity) stratified by molecular subtype classification subgroup.

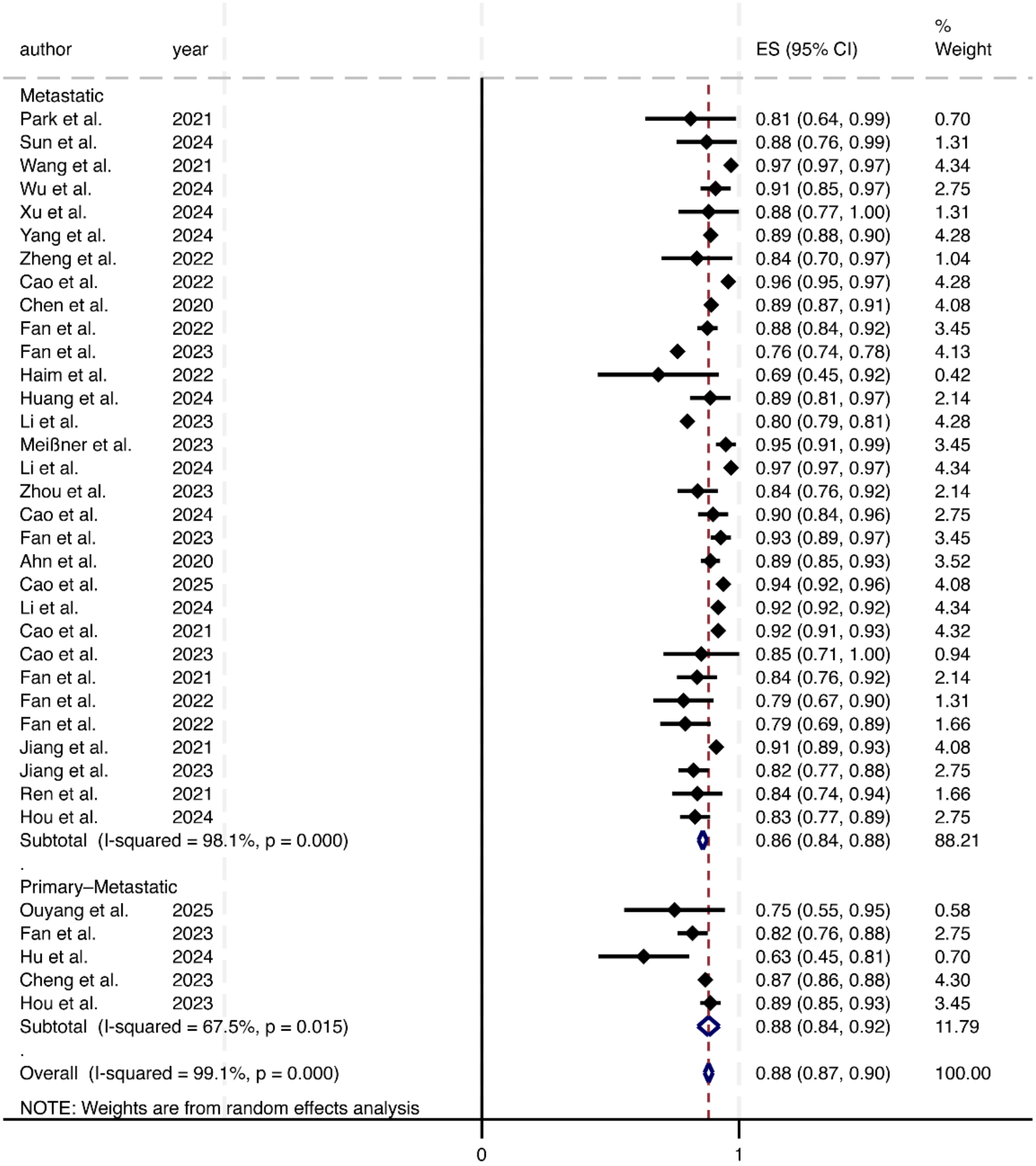

Supplementary Figure 5. Forest plot of pooled Molecular-level prediction performance (sensitivity) stratified by lesion-level ROI subgroup.

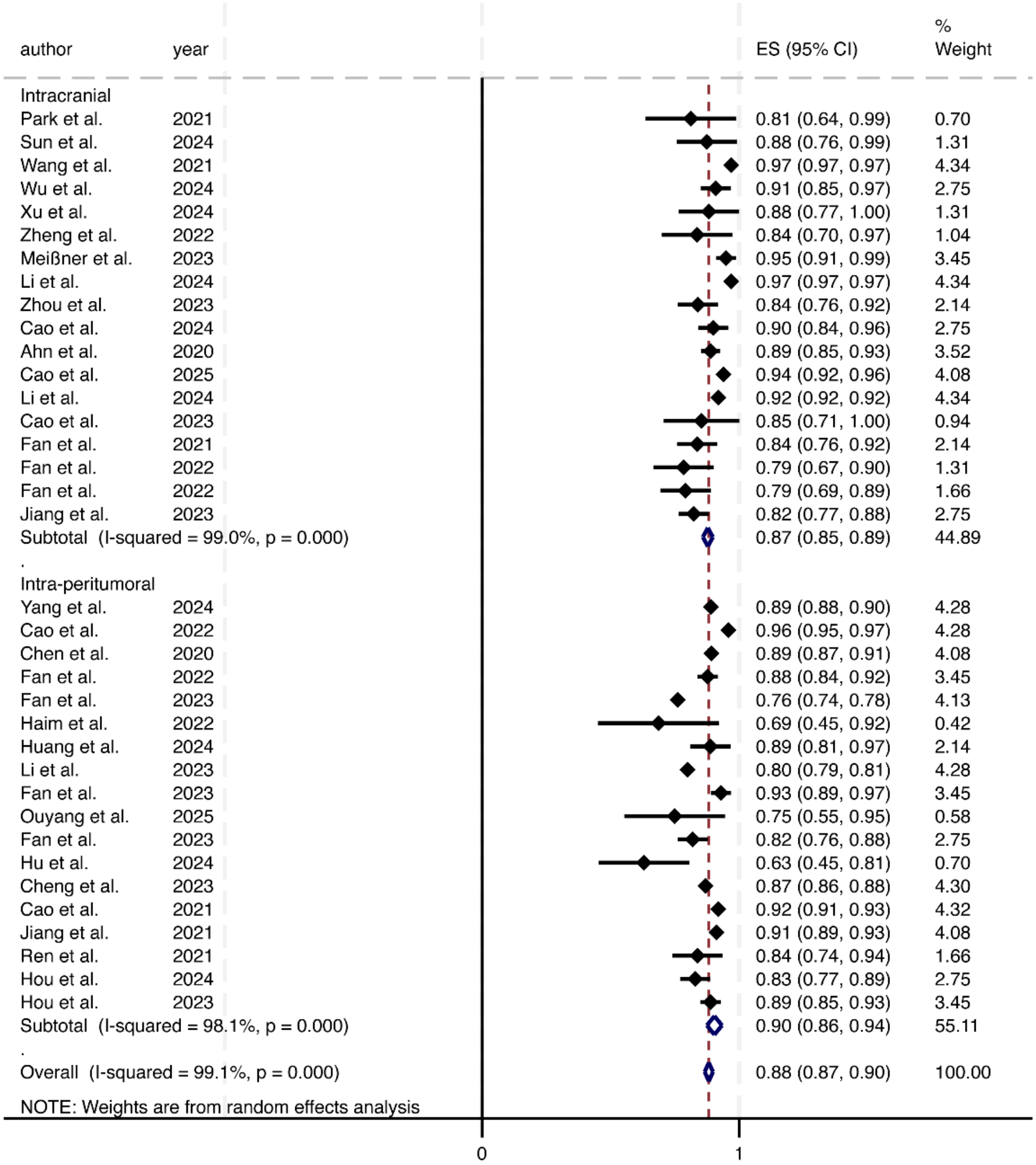

Supplementary Figure 6. Forest plot of pooled Molecular-level prediction performance (sensitivity) stratified by local metastatic ROI subgroup.

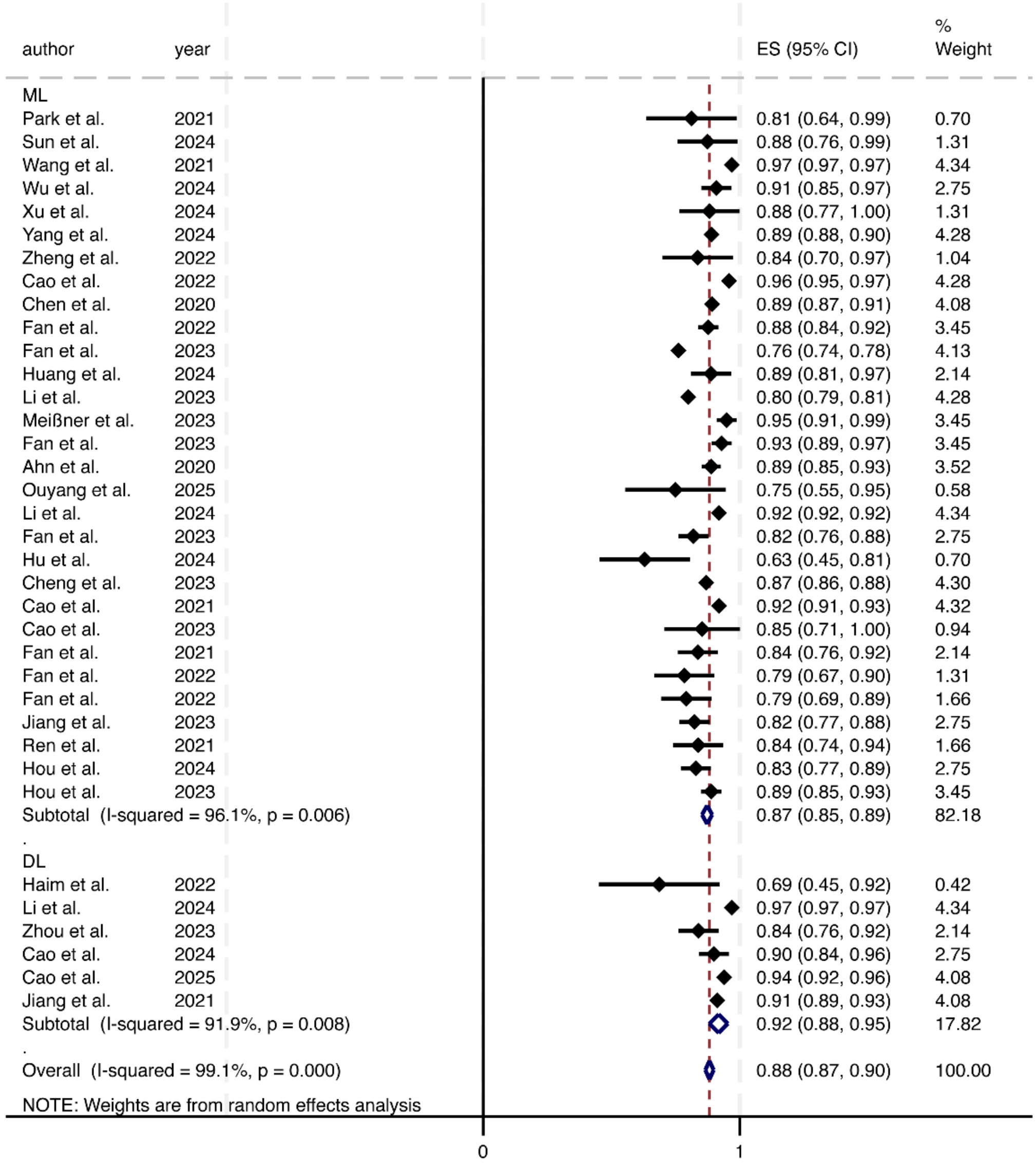

Supplementary Figure 7. Forest plot of pooled Molecular-level prediction performance (sensitivity) stratified by algorithm type (ML vs. DL).

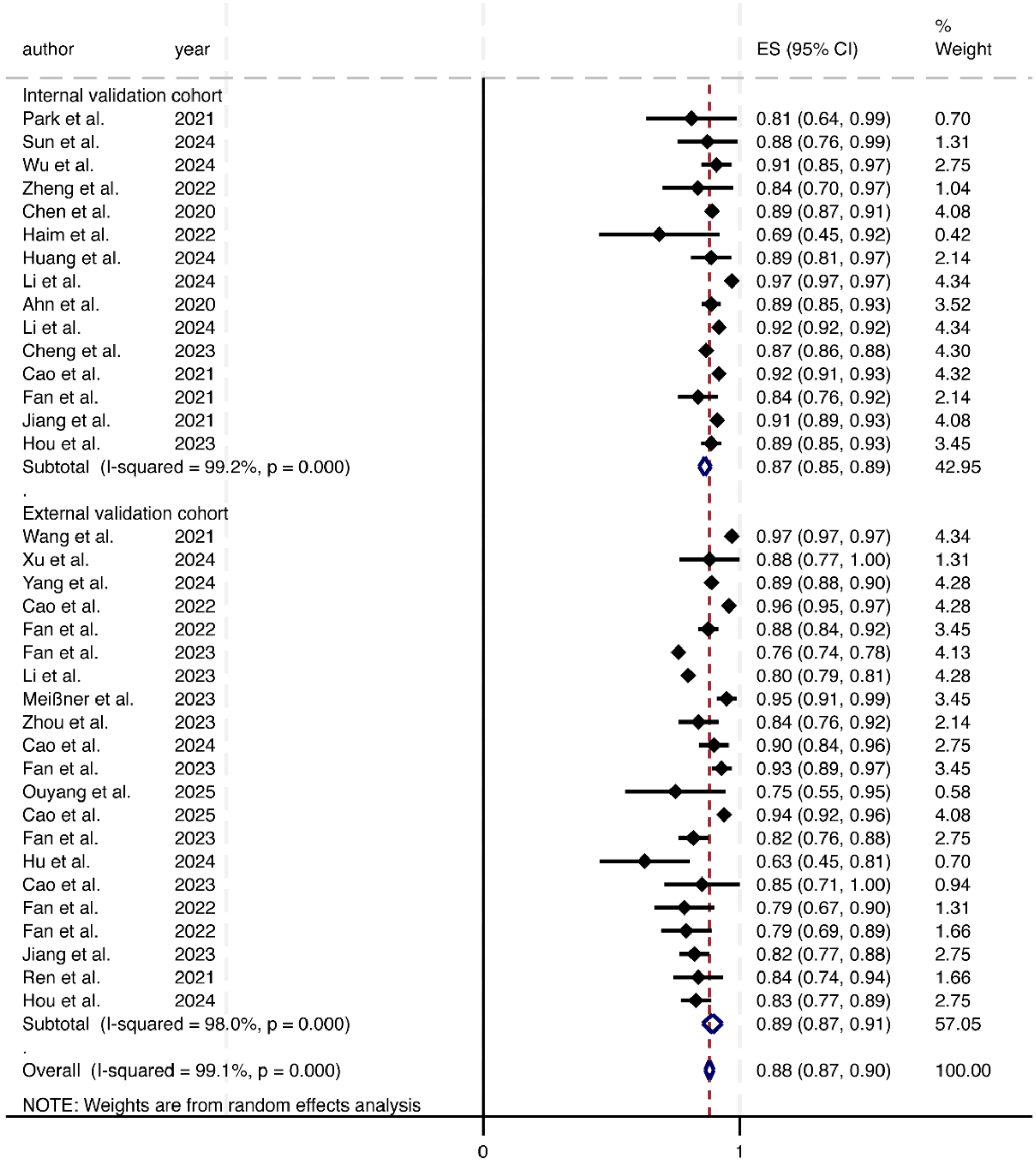

Supplementary Figure 8. Forest plot of pooled Molecular-level prediction performance (sensitivity) stratified by cohort type (internal vs. external).

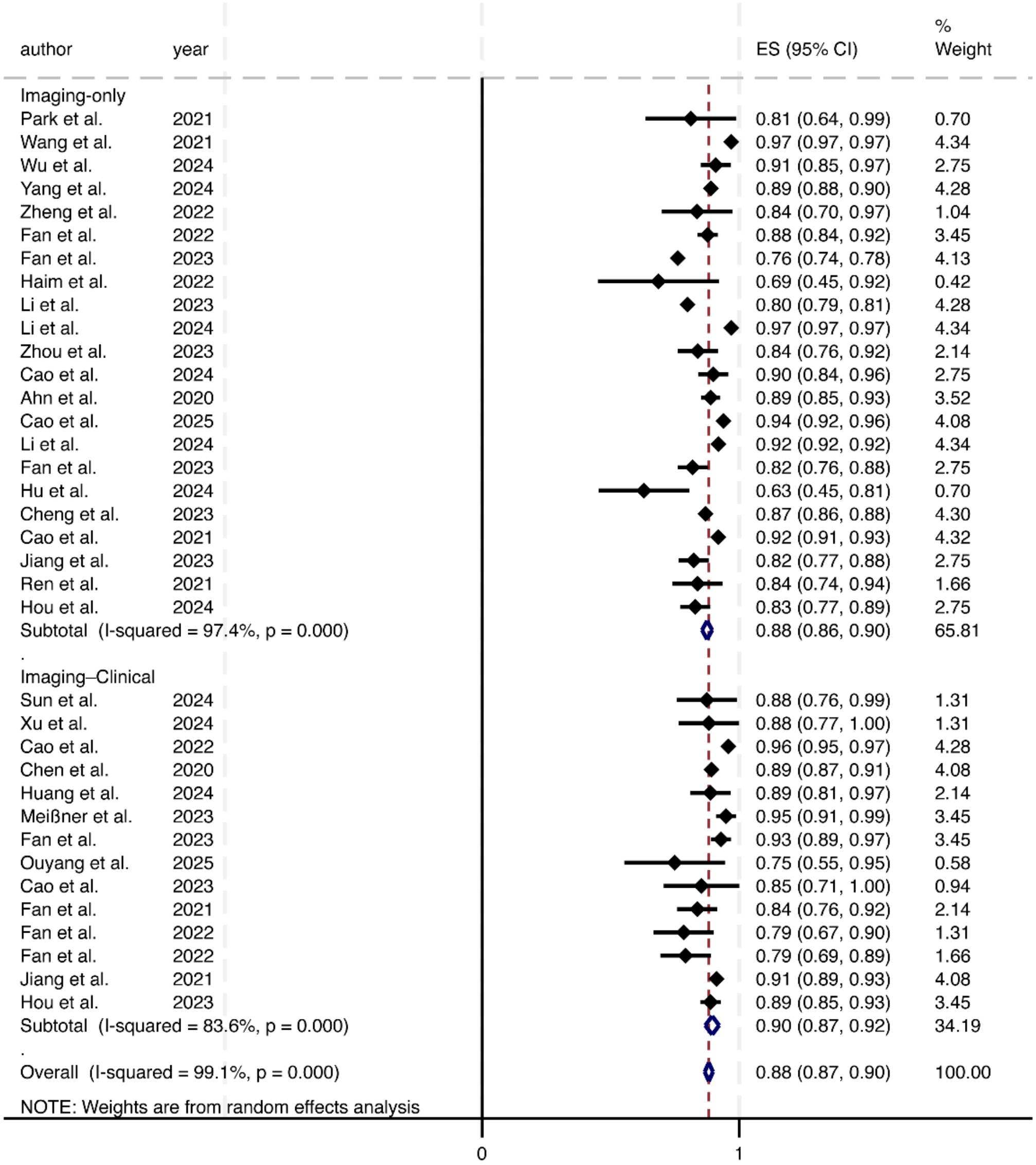

Supplementary Figure 9. Forest plot of pooled Molecular-level prediction performance (sensitivity) stratified by feature-source subgroup.

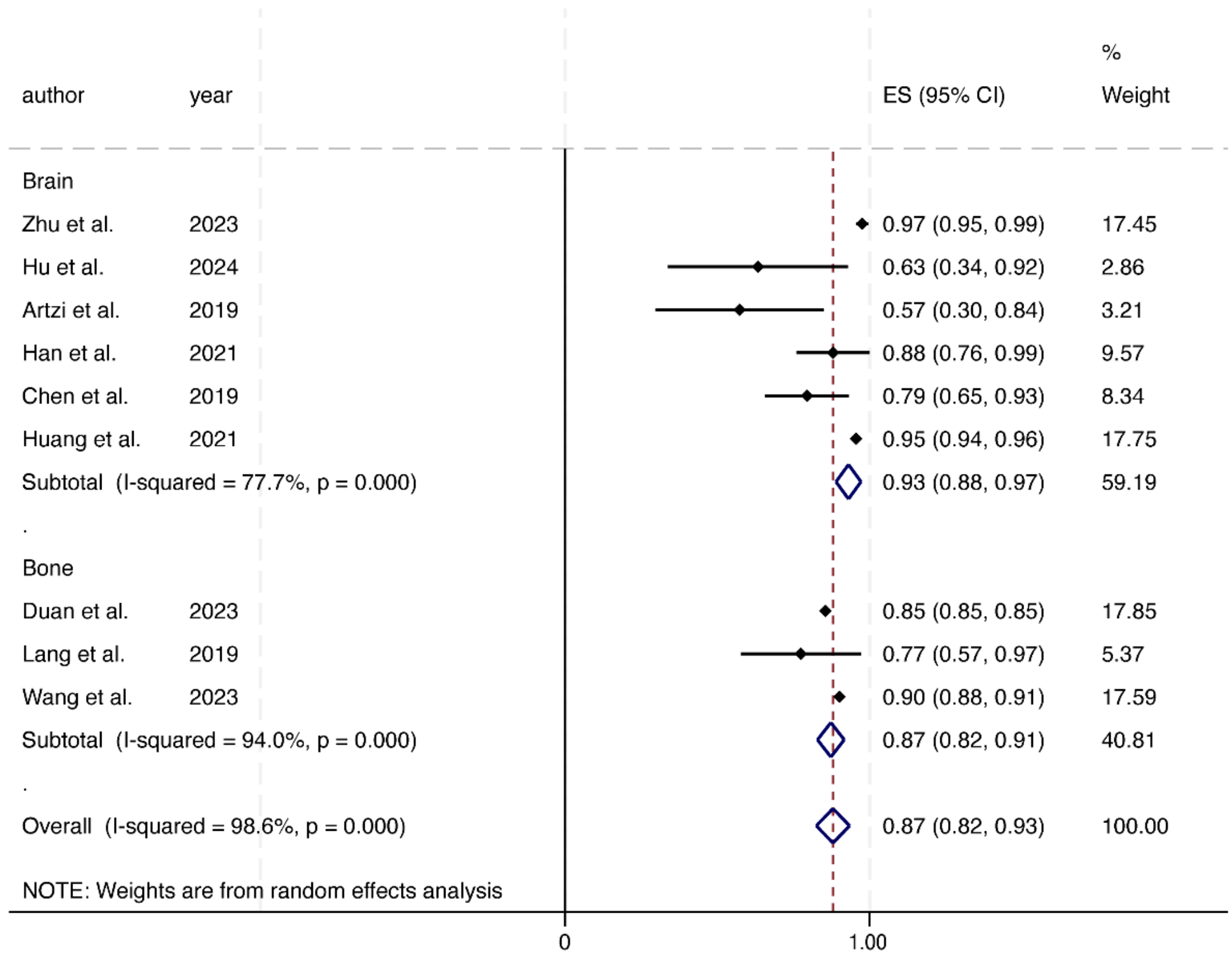

Supplementary Figure 10. Forest plot of pooled Metastasis differentiation performance (sensitivity) stratified by metastatic lesion subgroup.

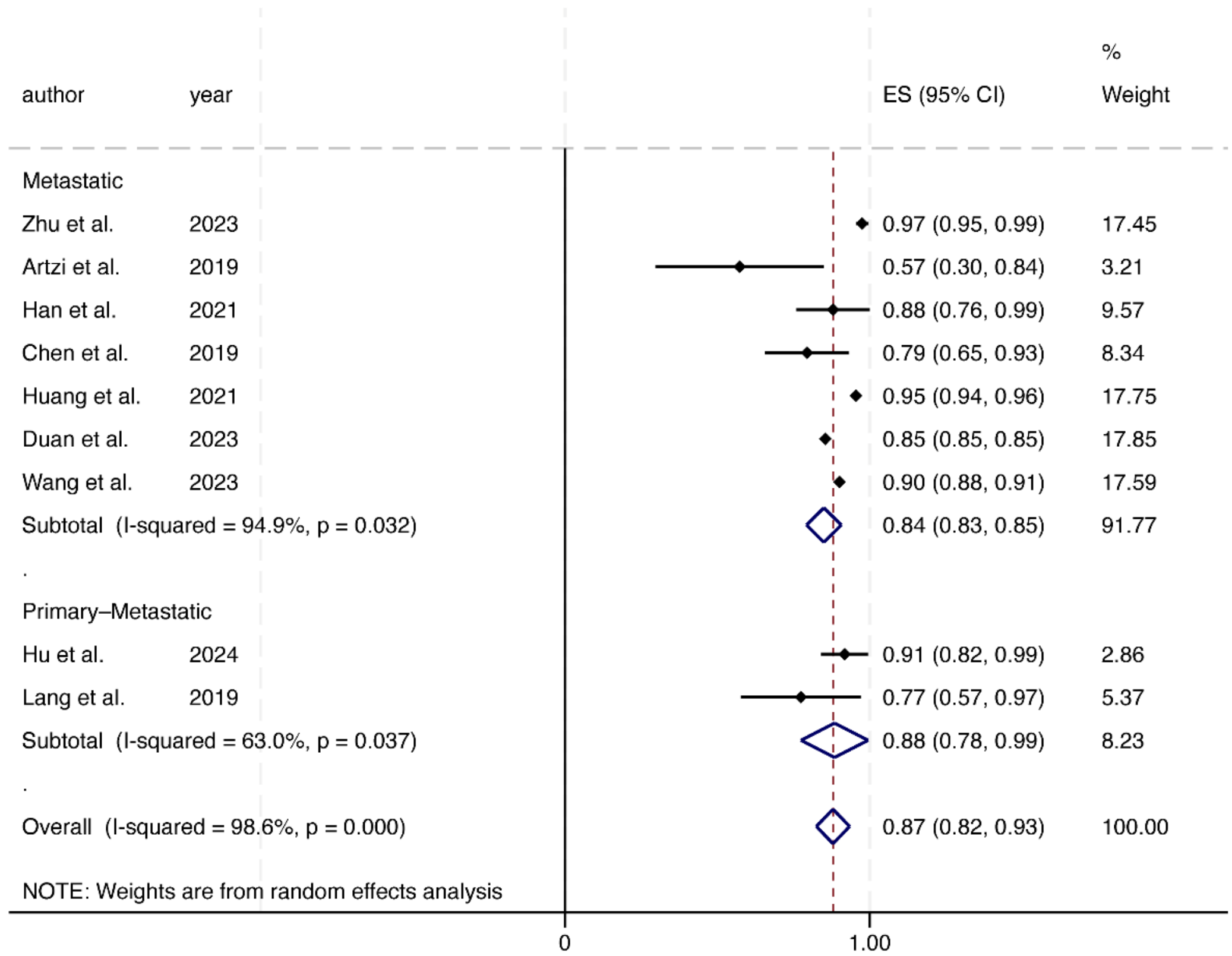

Supplementary Figure 11. Forest plot of pooled Metastasis differentiation performance (sensitivity) stratified by lesion-level ROI subgroup.

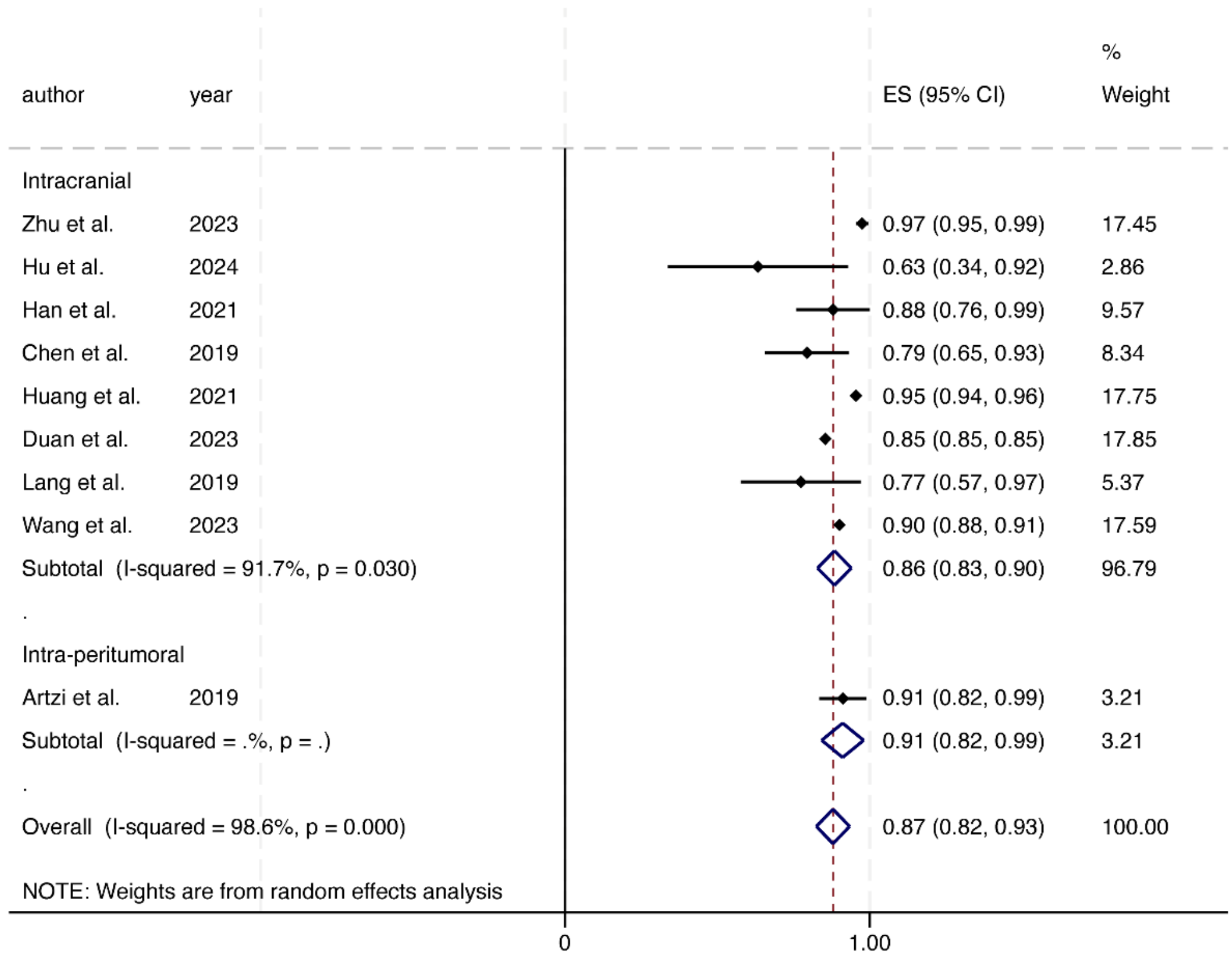

Supplementary Figure 12. Forest plot of pooled Metastasis differentiation performance (sensitivity) stratified by local metastatic ROI subgroup.

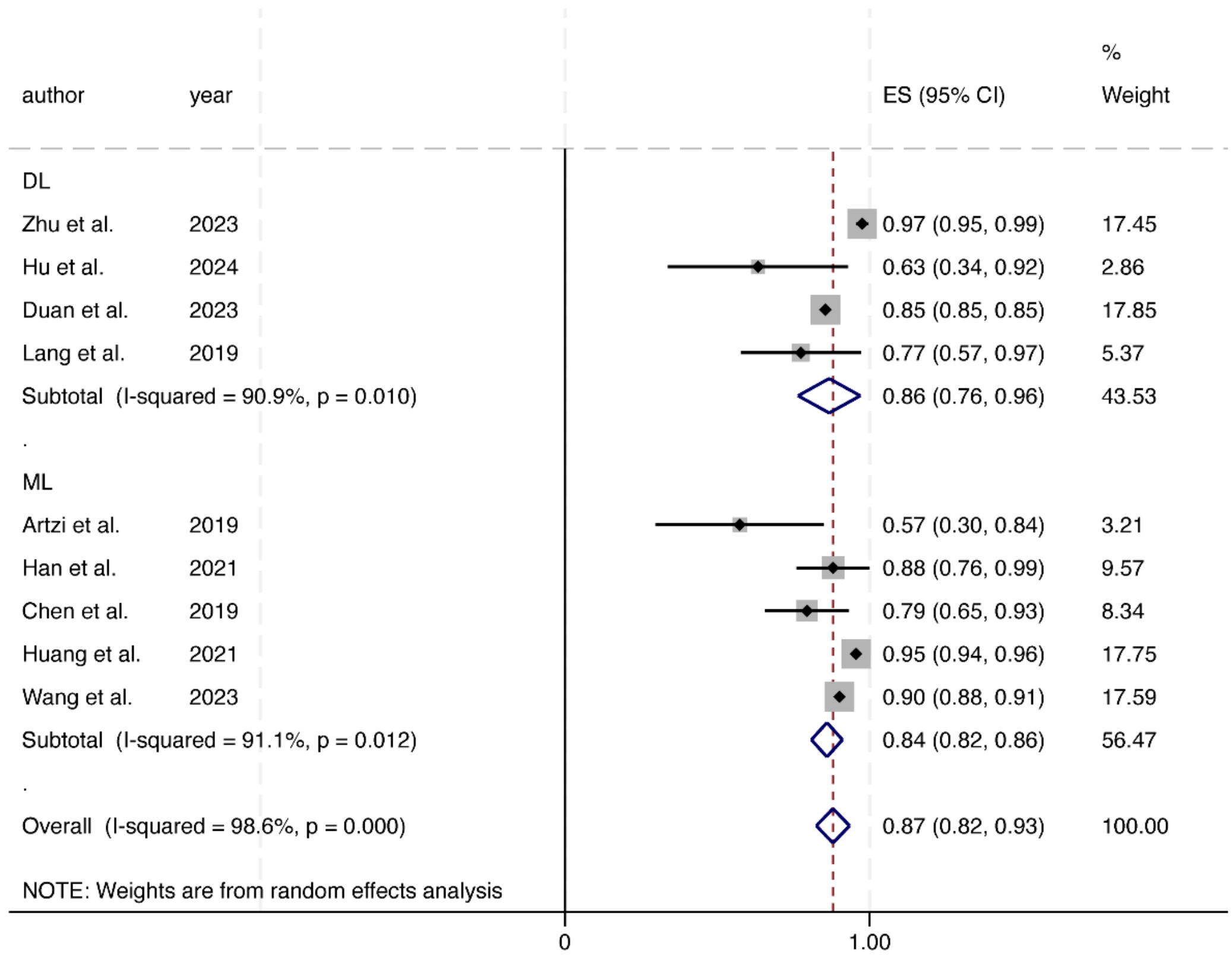

Supplementary Figure 13. Forest plot of pooled Metastasis differentiation performance (sensitivity) stratified by algorithm type (ML vs. DL).

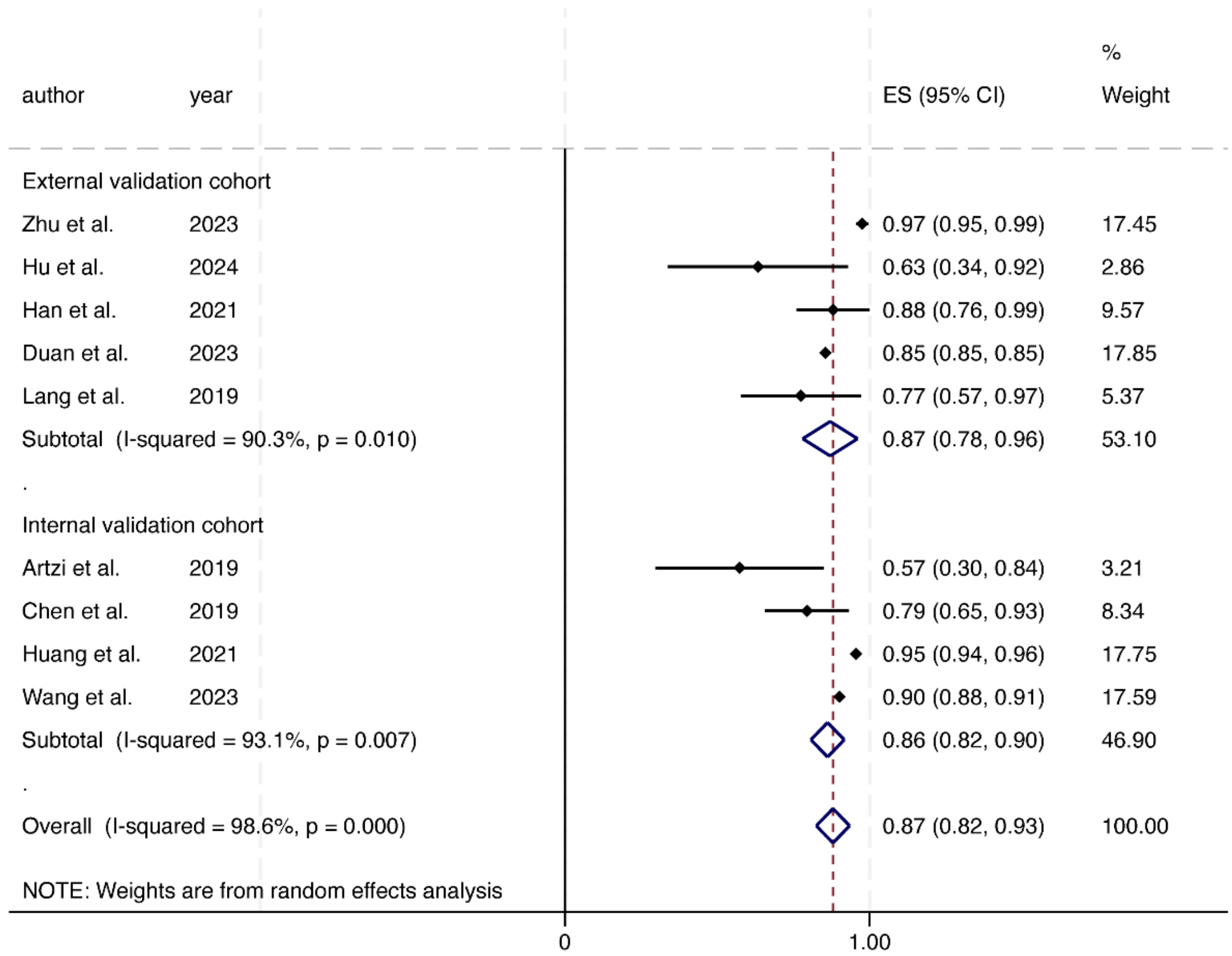

Supplementary Figure 14. Forest plot of pooled Metastasis differentiation performance (sensitivity) stratified by cohort type (internal vs. external).

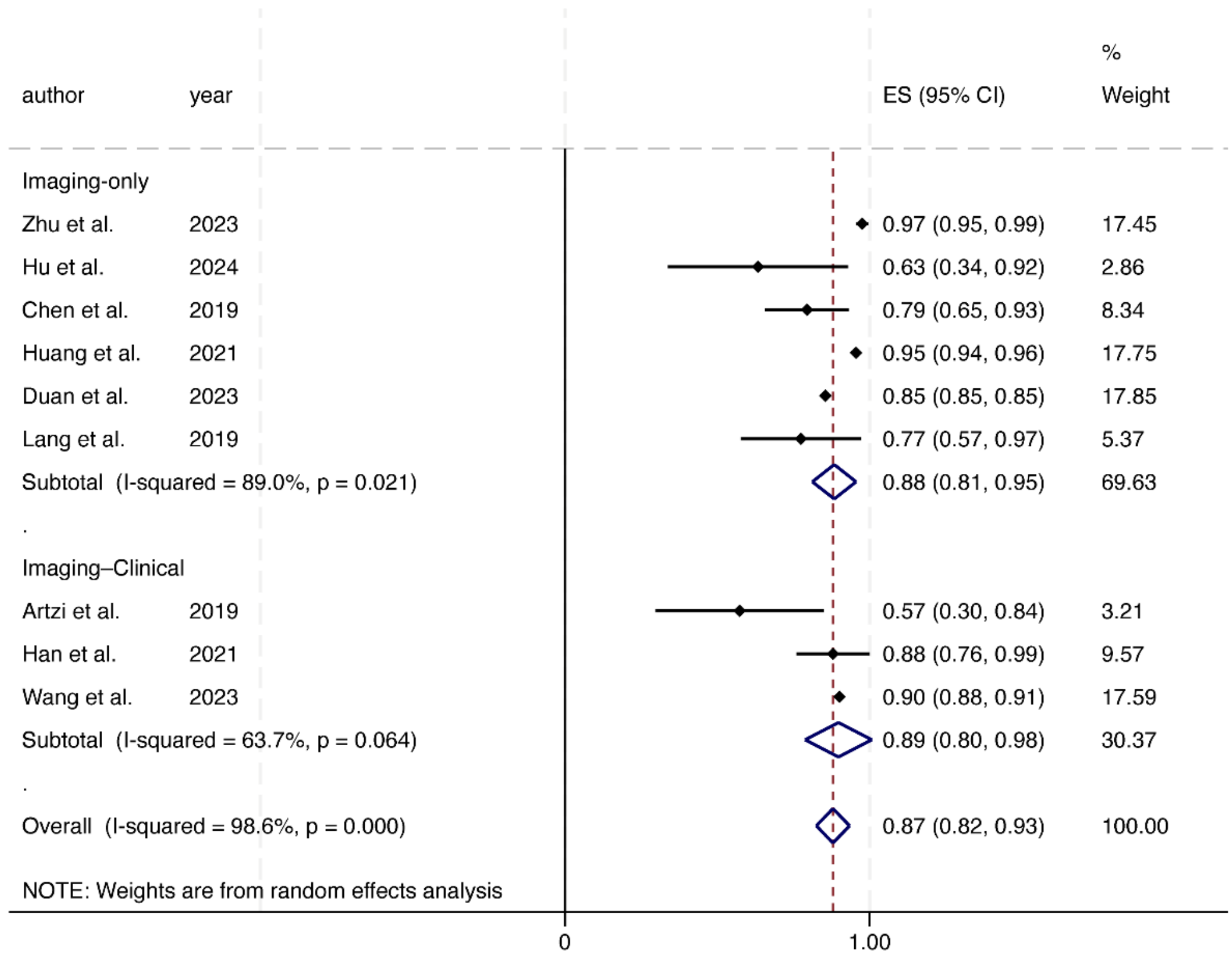

Supplementary Figure 15. Forest plot of pooled Metastasis differentiation performance (sensitivity) stratified by feature-source subgroup.

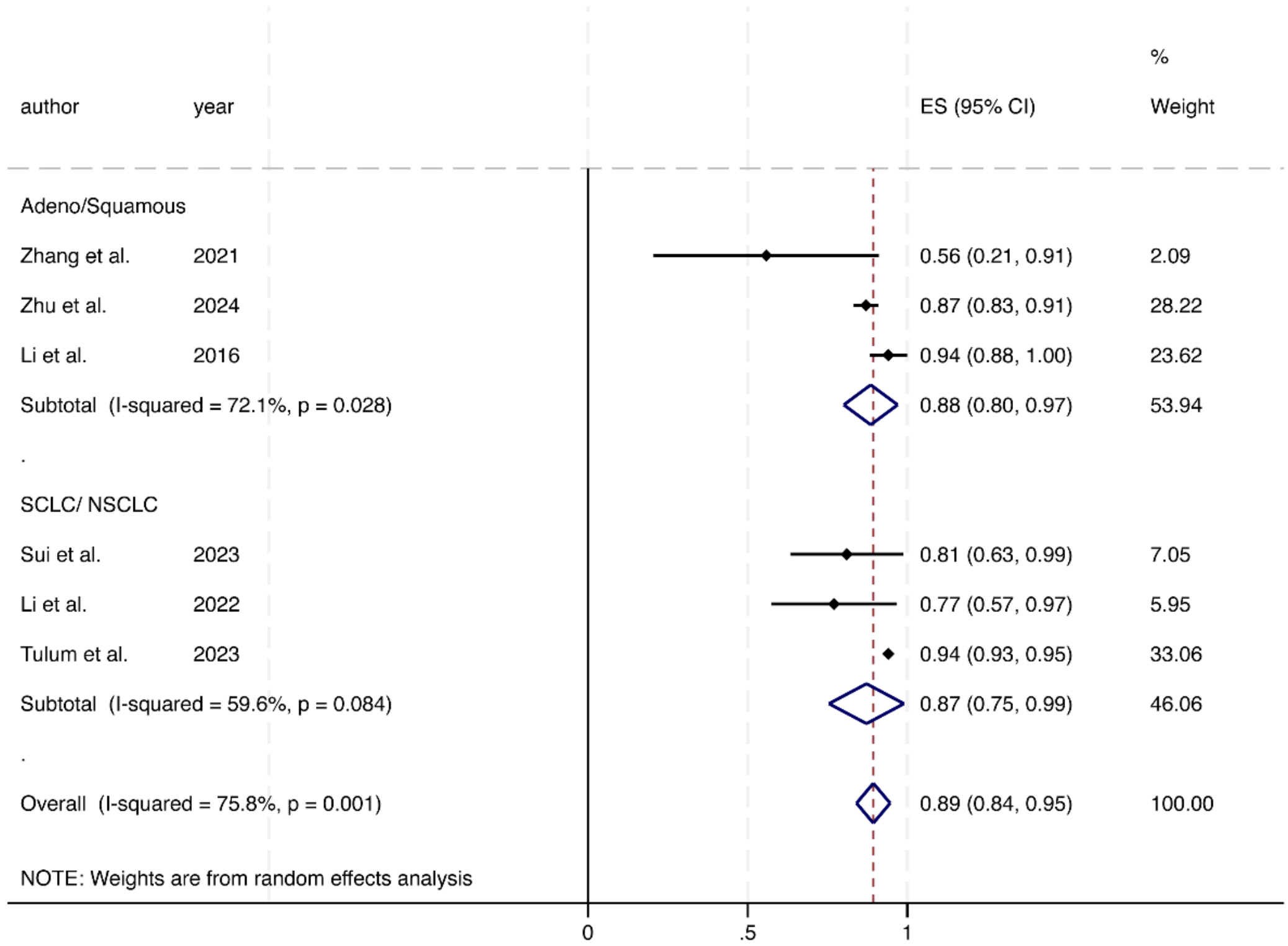

Supplementary Figure 16. Forest plot of pooled Tumor type classification performance (sensitivity) stratified by histologic subtype classification.

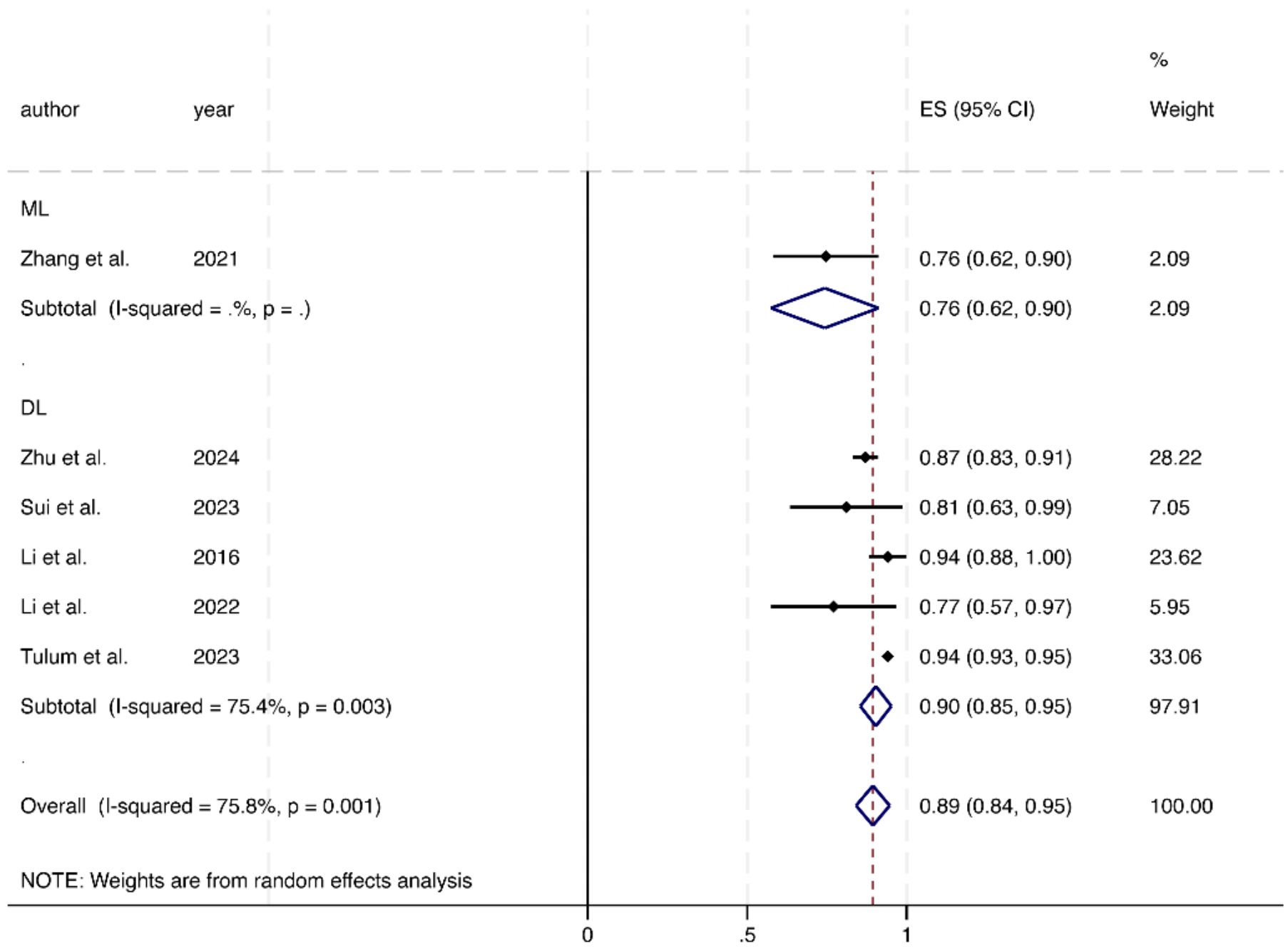

Supplementary Figure 17. Forest plot of pooled Tumor type classification performance (sensitivity) stratified by algorithm type (ML vs. DL).

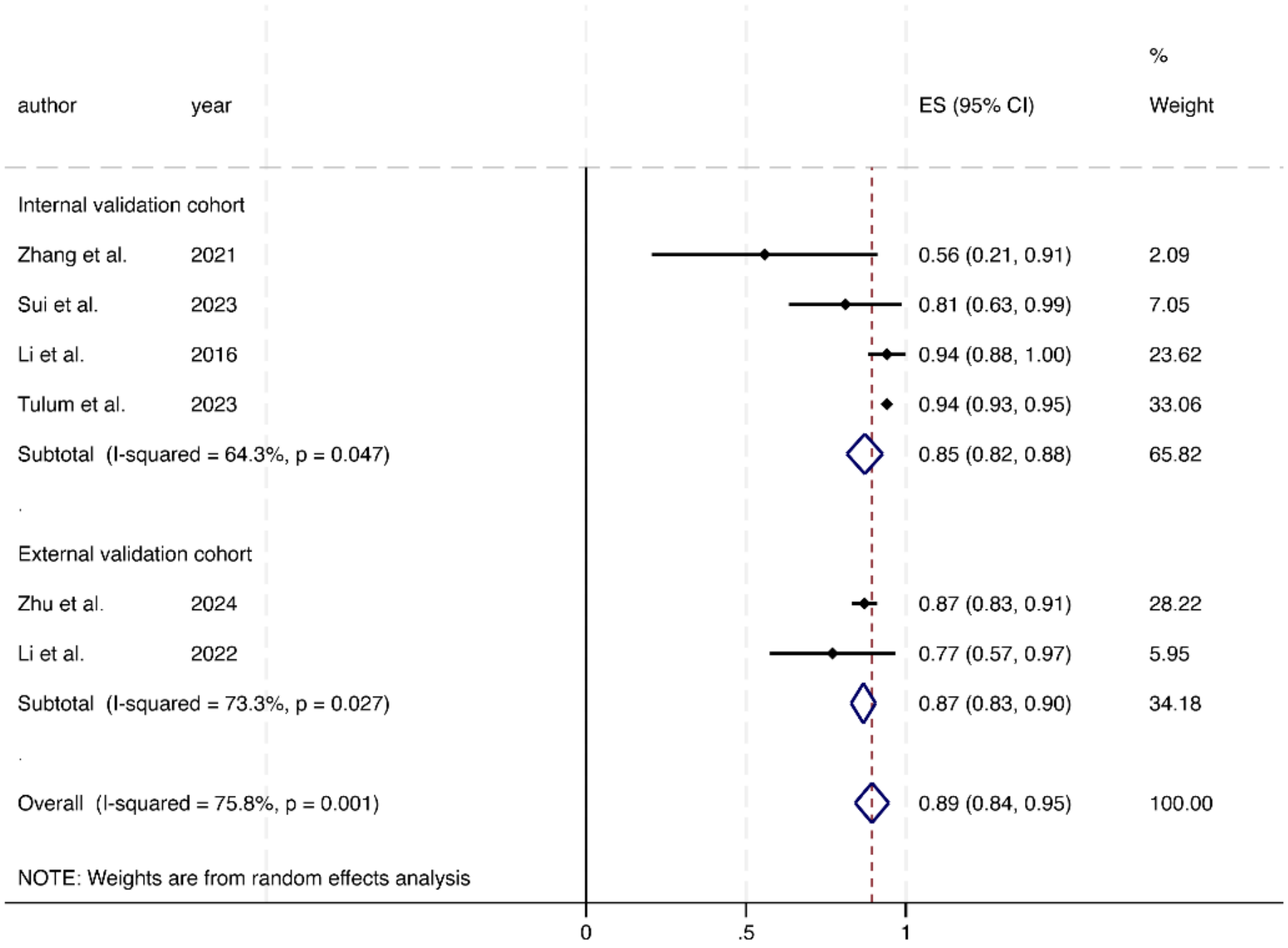

Supplementary Figure 18. Forest plot of pooled Tumor type classification performance (sensitivity) stratified by cohort type (internal vs. external).

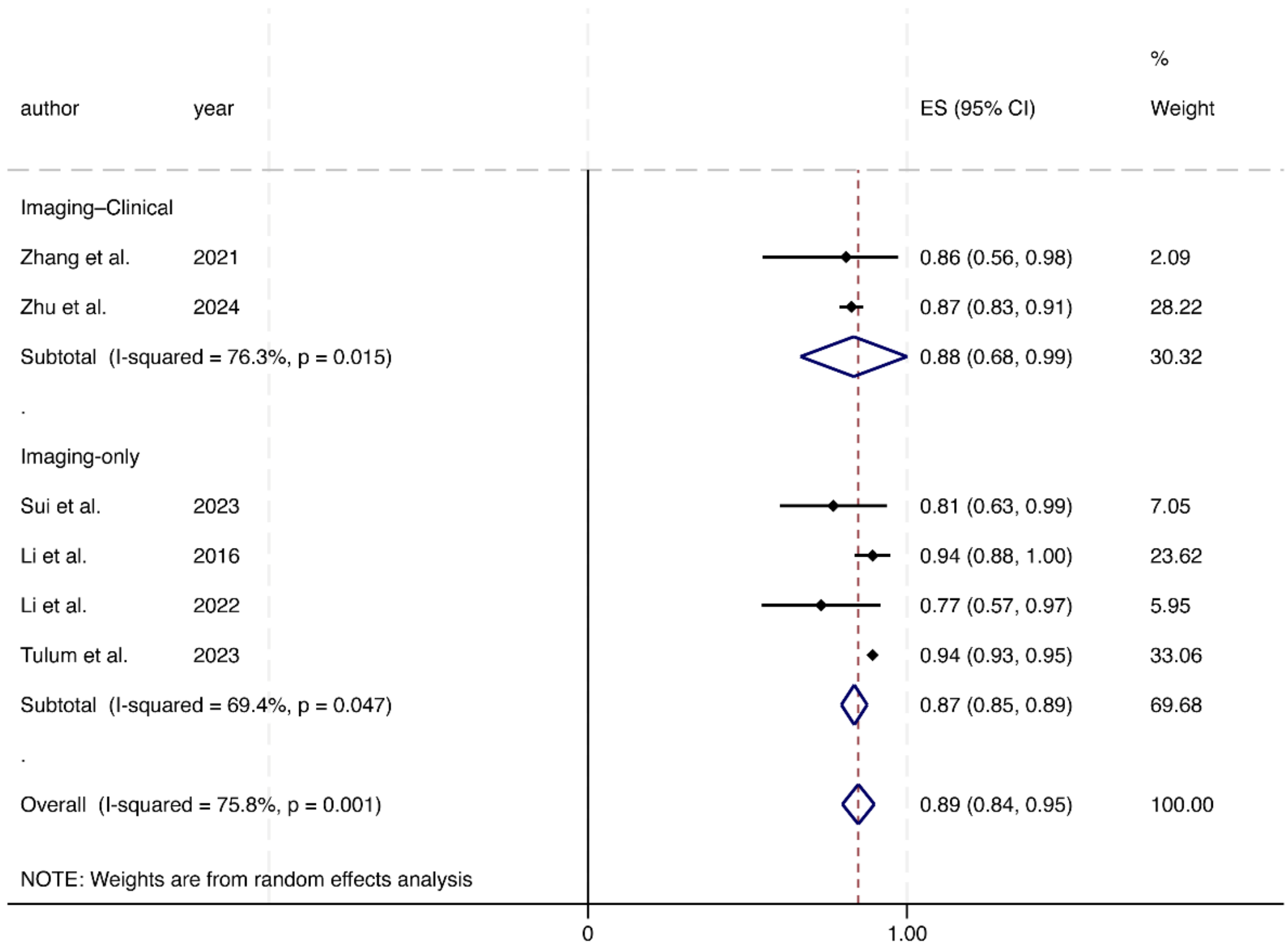

Supplementary Figure 19. Forest plot of pooled Tumor type classification performance (sensitivity) stratified by feature-source subgroup.

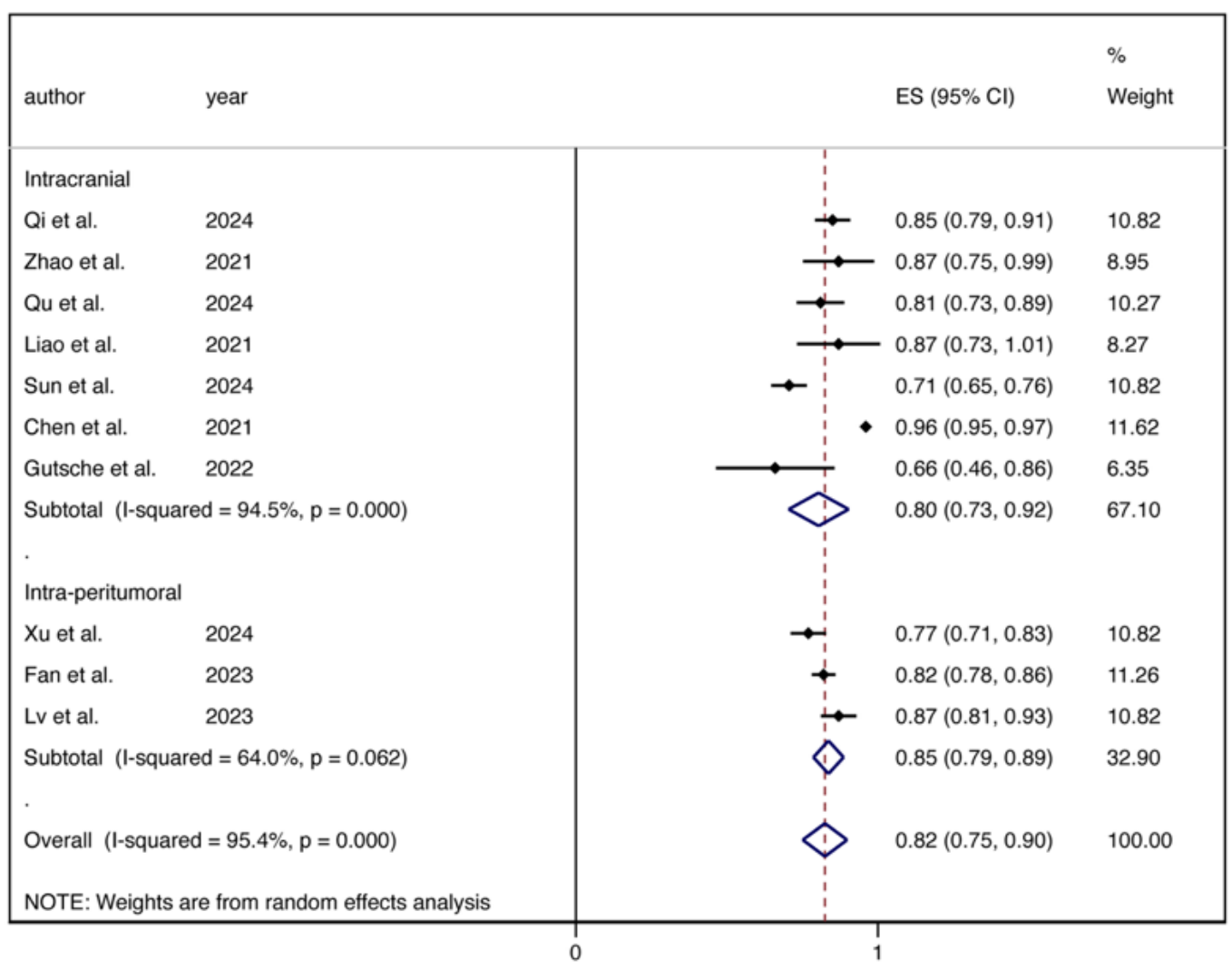

Supplementary Figure 20. Forest plot of pooled Outcome prediction (sensitivity) stratified by local metastatic ROI subgroup.

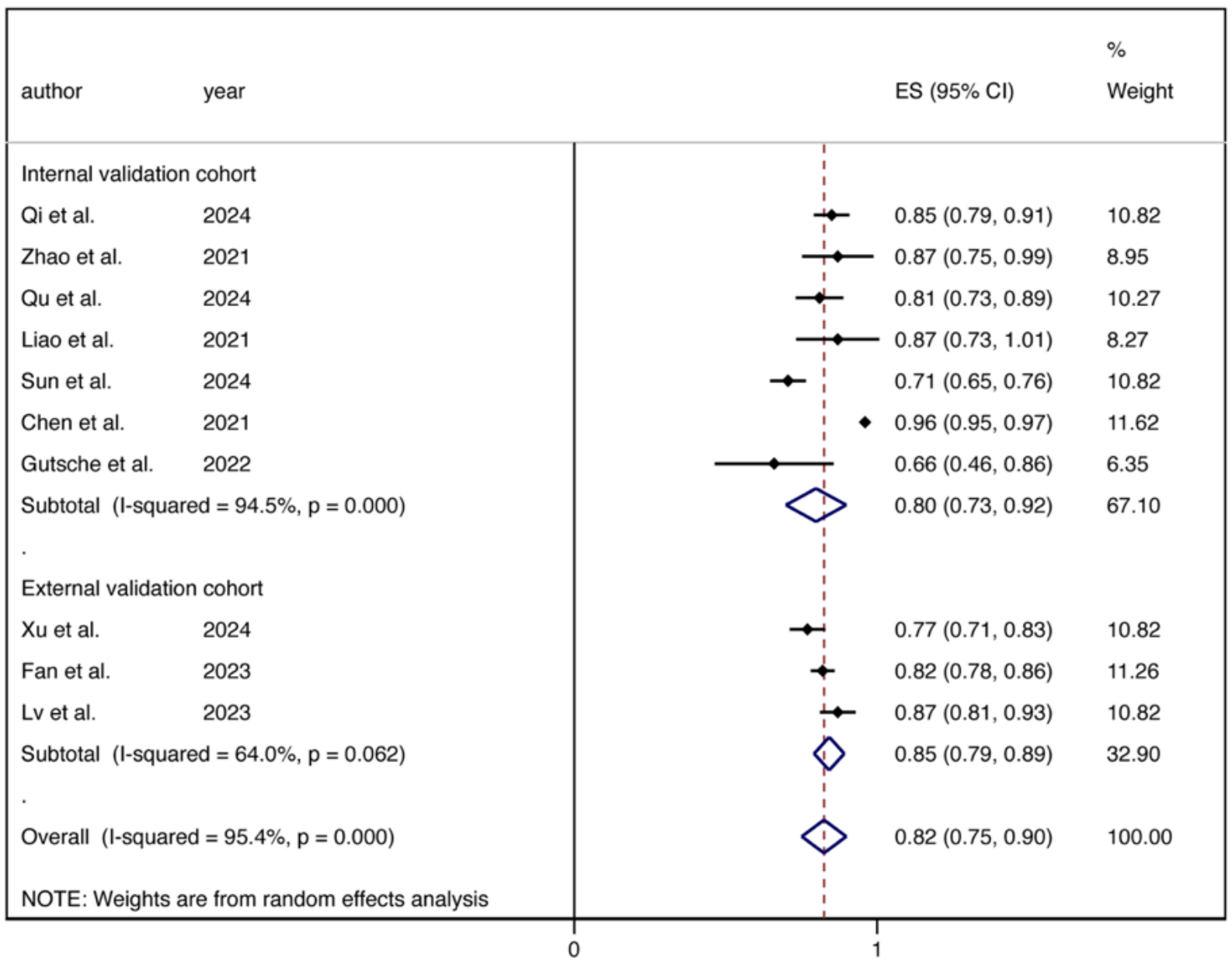

Supplementary Figure 21. Forest plot of pooled Outcome prediction (sensitivity) stratified by cohort type (internal vs. external).

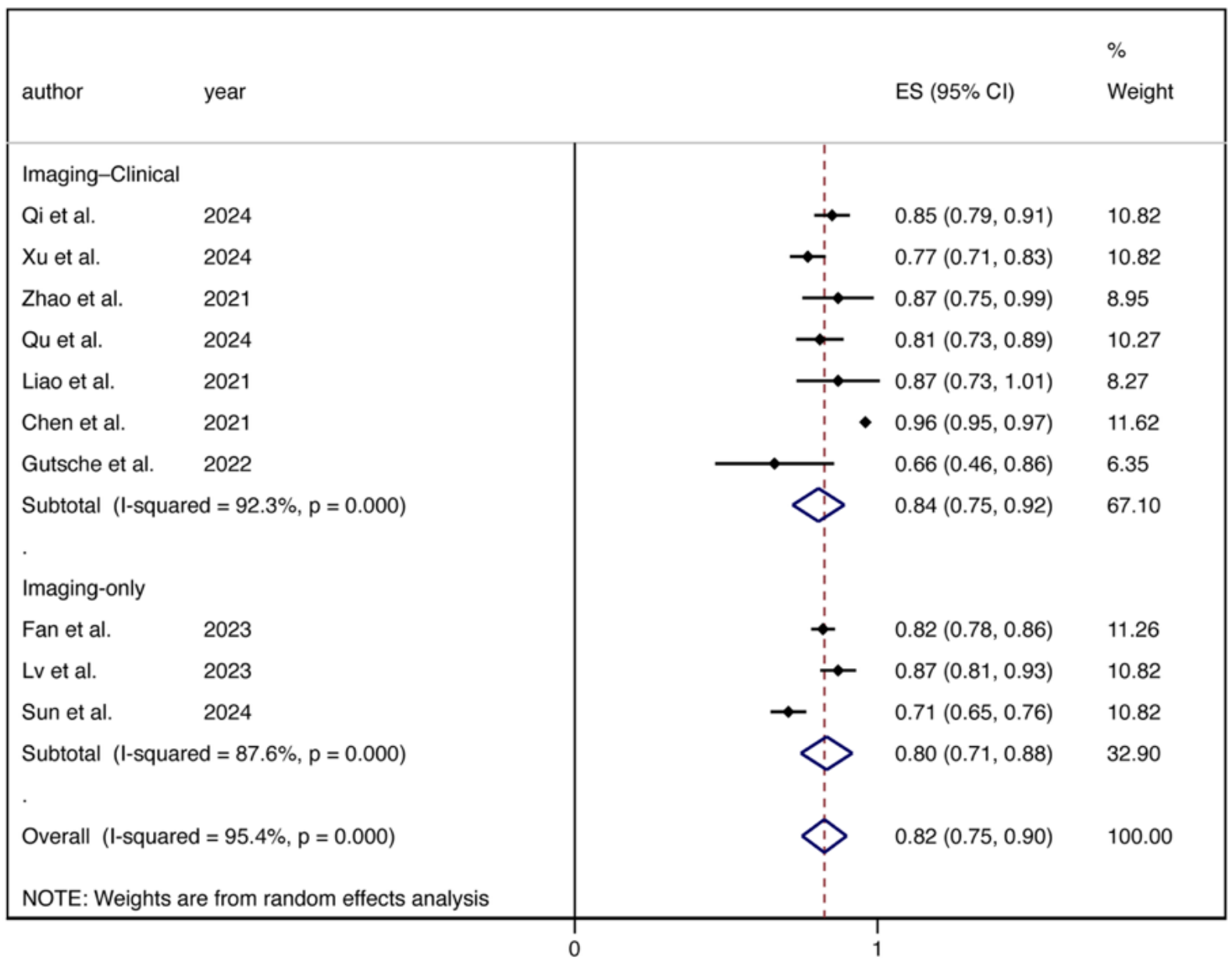

Supplementary Figure 22. Forest plot of pooled Outcome prediction (sensitivity) stratified by feature-source subgroup.

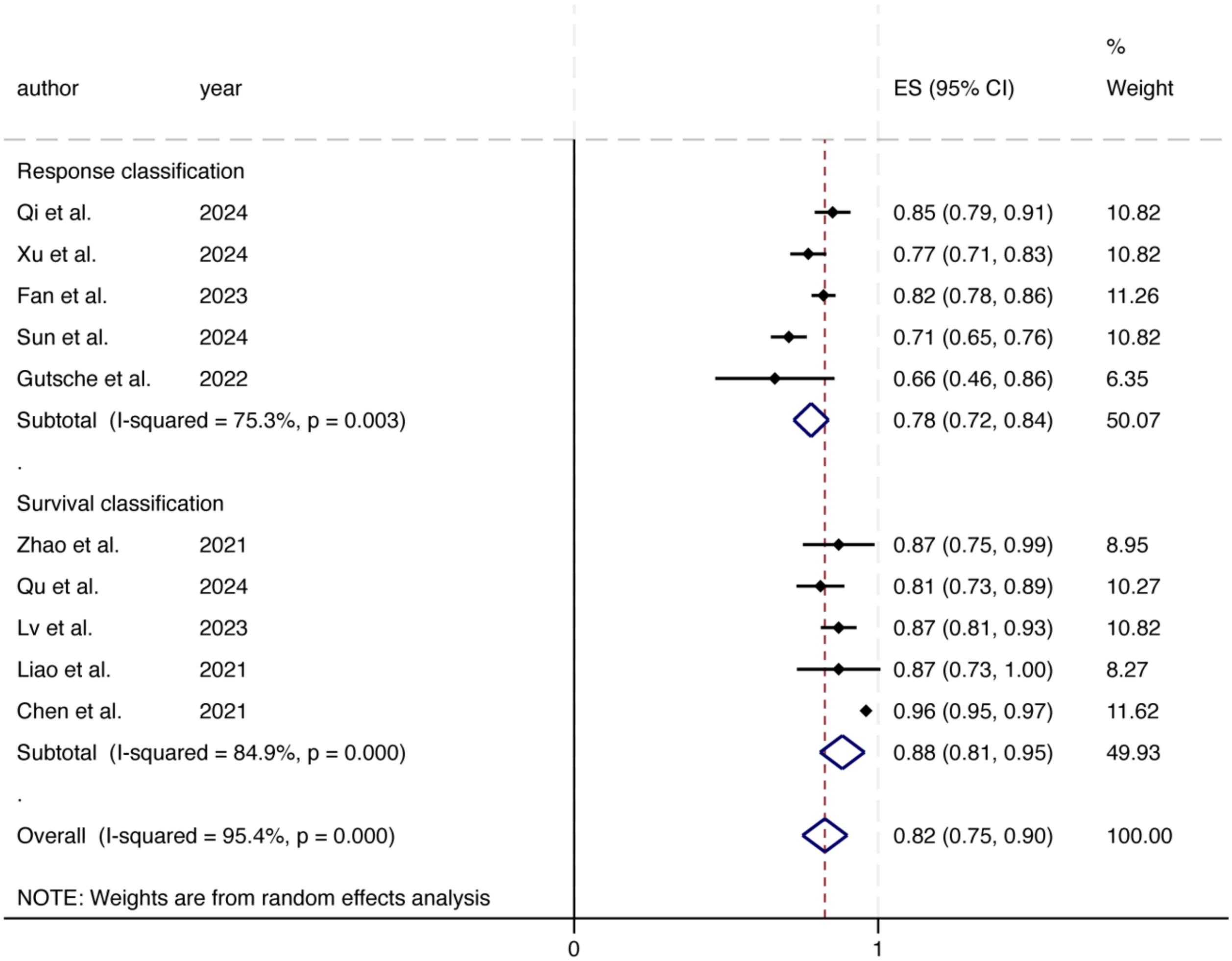

Supplementary Figure 23. Forest plot of pooled Outcome prediction (sensitivity) stratified by prognostic type classification.

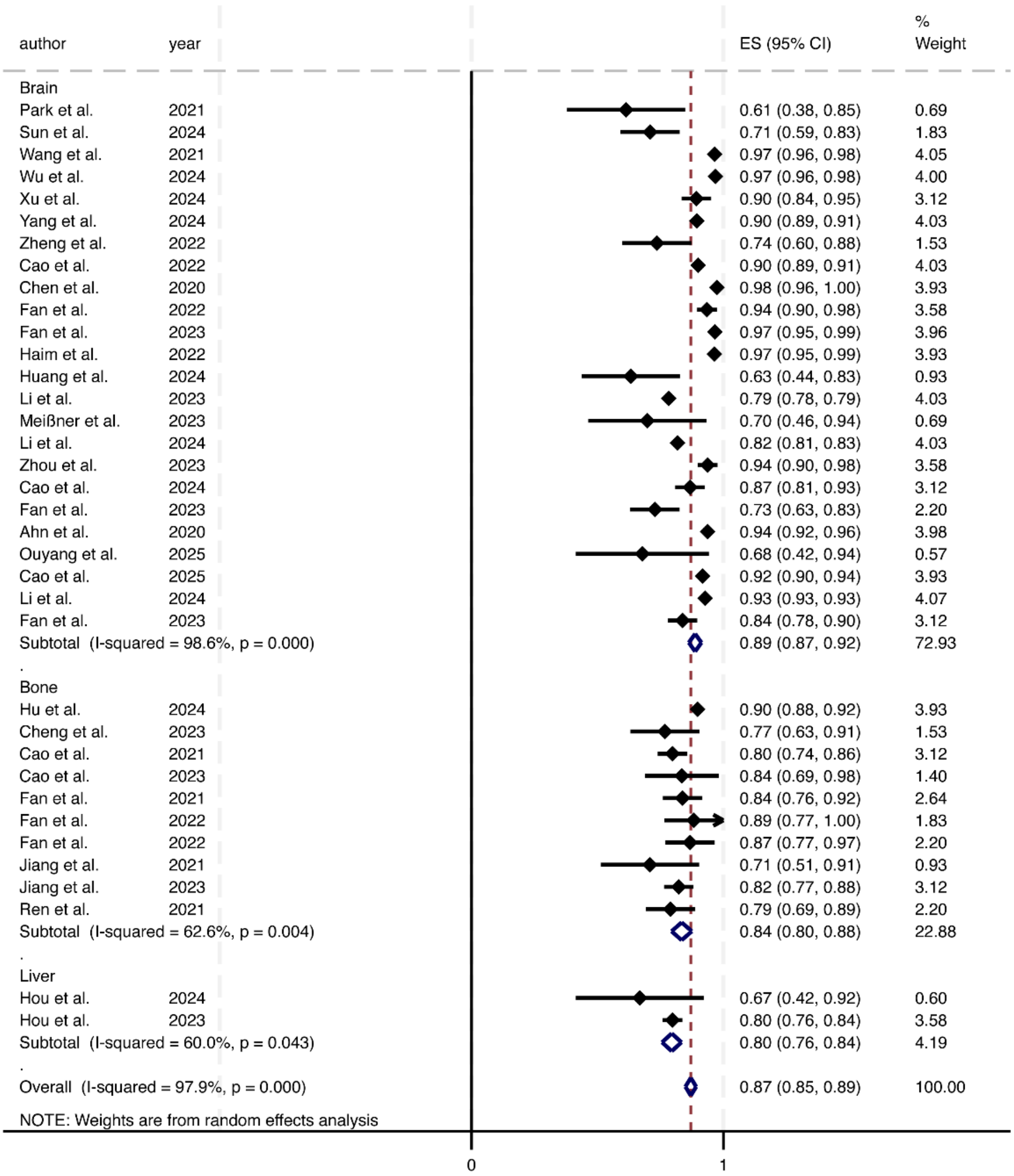

Supplementary Figure 24. Forest plot of pooled Molecular-level prediction performance (specificity) stratified by metastatic lesion subgroup.

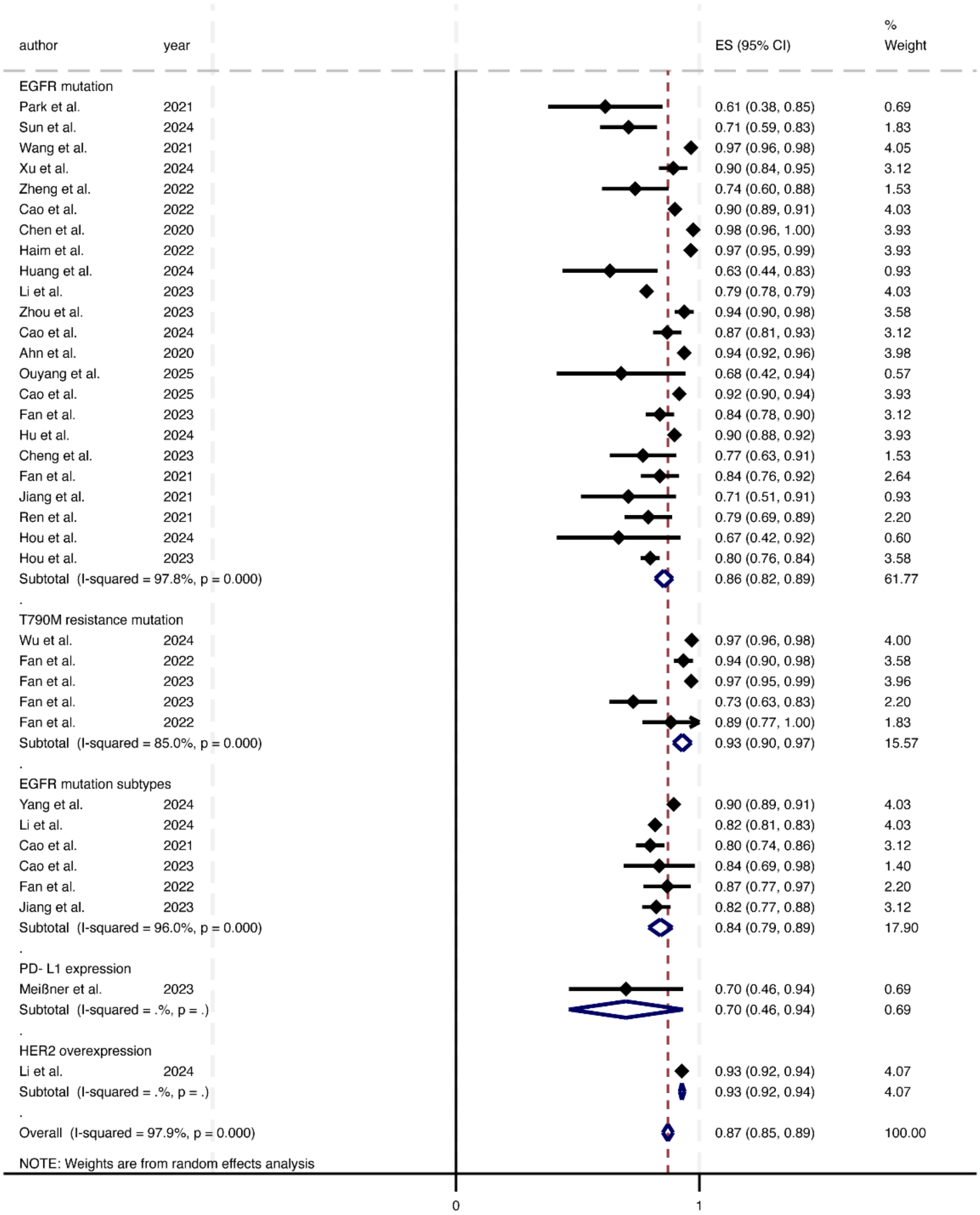

Supplementary Figure 25. Forest plot of pooled Molecular-level prediction performance (specificity) stratified by molecular subtype classification subgroup.

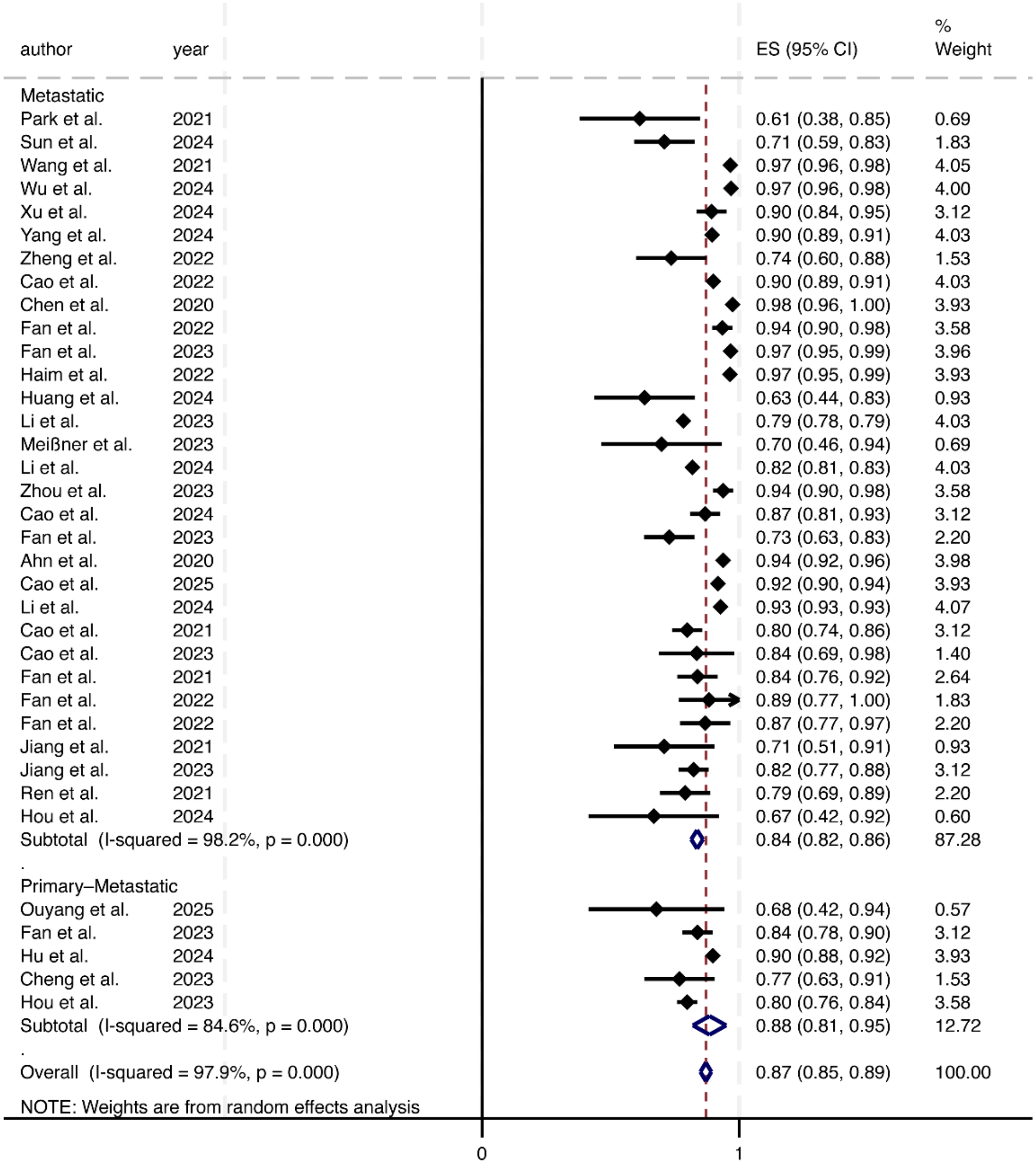

Supplementary Figure 26. Forest plot of pooled Molecular-level prediction performance (specificity) stratified by lesion-level ROI subgroup.

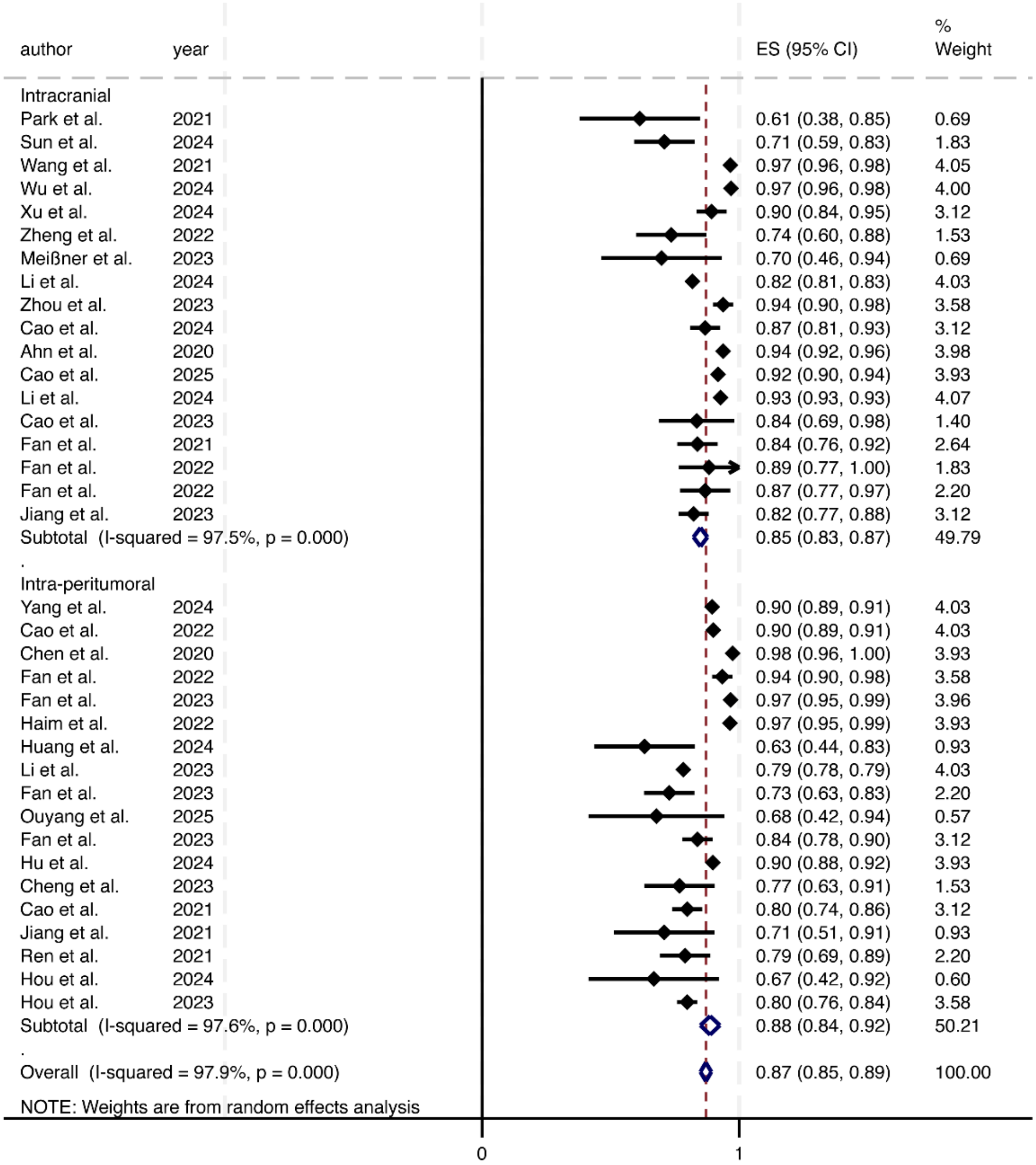

Supplementary Figure 27. Forest plot of pooled Molecular-level prediction performance (specificity) stratified by local metastatic ROI subgroup.

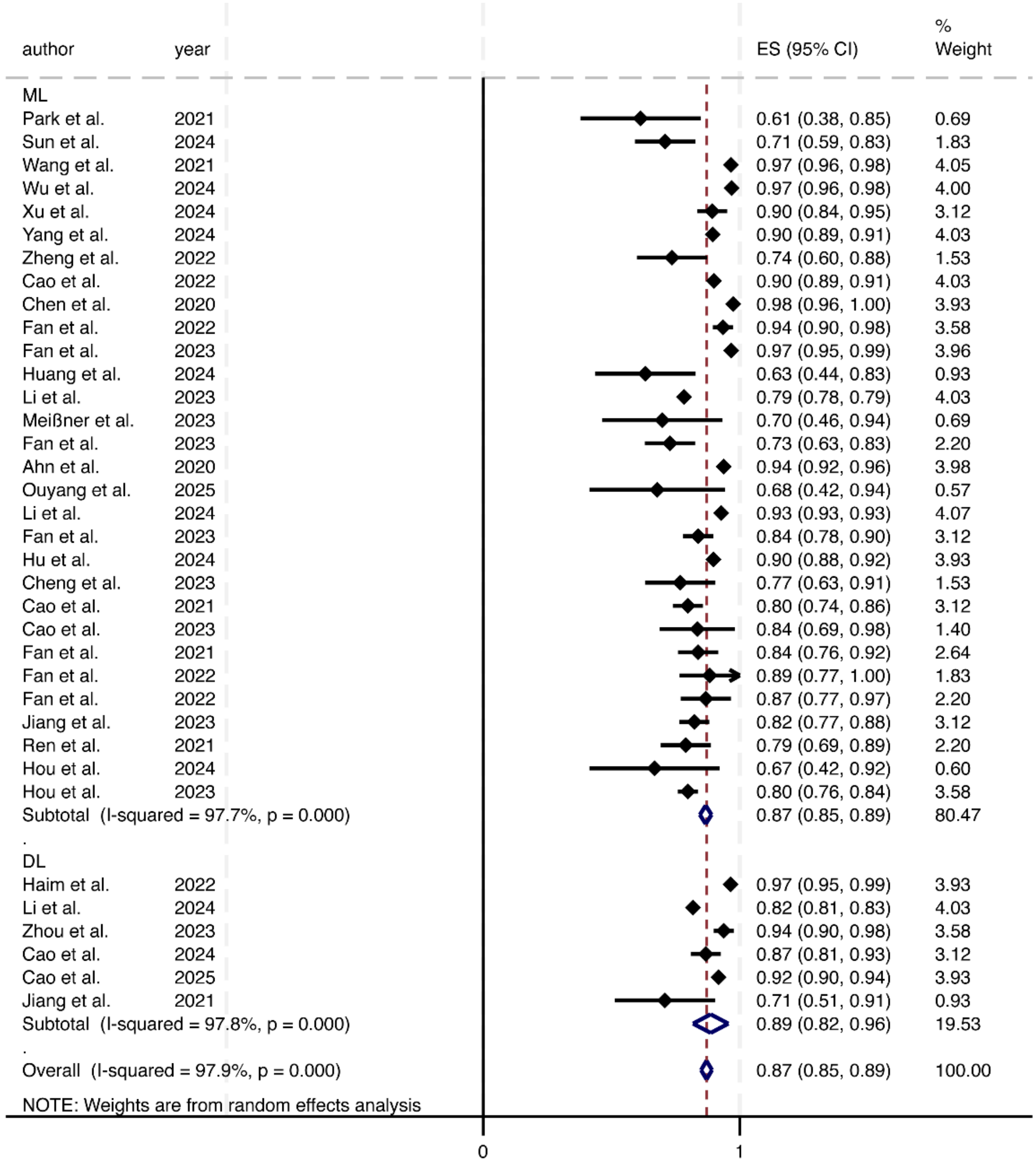

Supplementary Figure 28. Forest plot of pooled Molecular-level prediction performance (specificity) stratified by algorithm type (ML vs. DL).

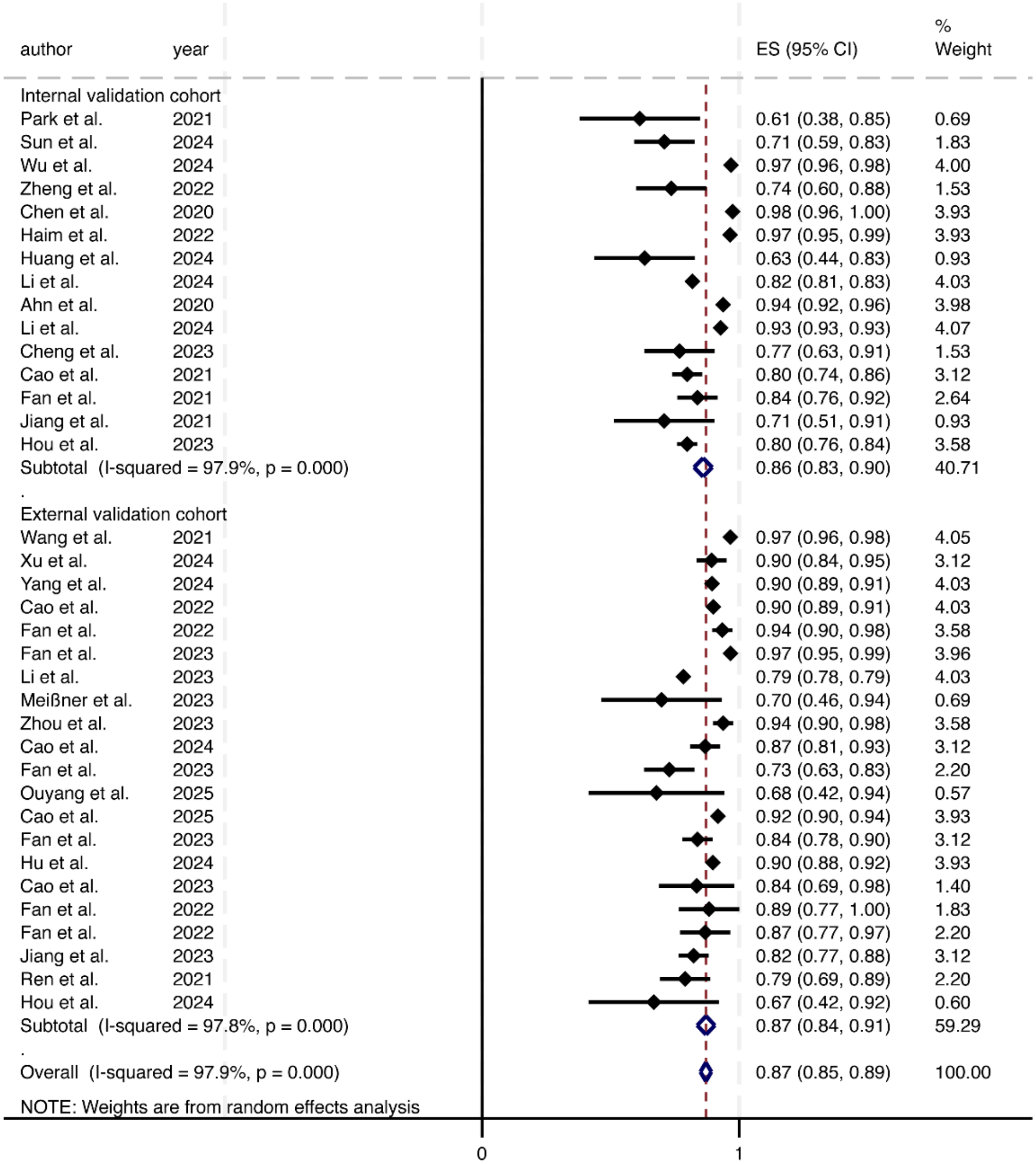

Supplementary Figure 29. Forest plot of pooled Molecular-level prediction performance (specificity) stratified by cohort type (internal vs. external).

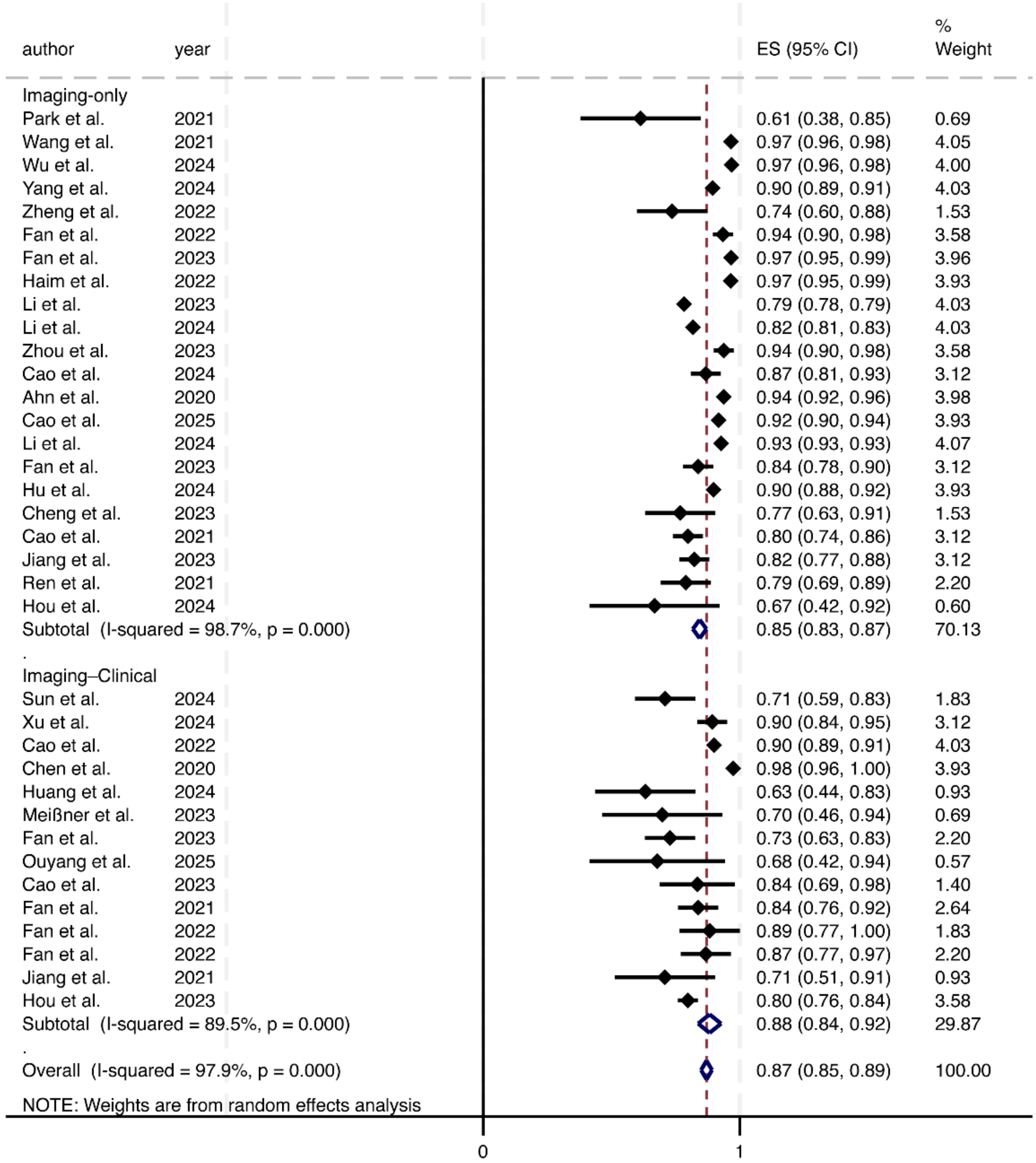

Supplementary Figure 30. Forest plot of pooled Molecular-level prediction performance (specificity) stratified by feature-source subgroup.

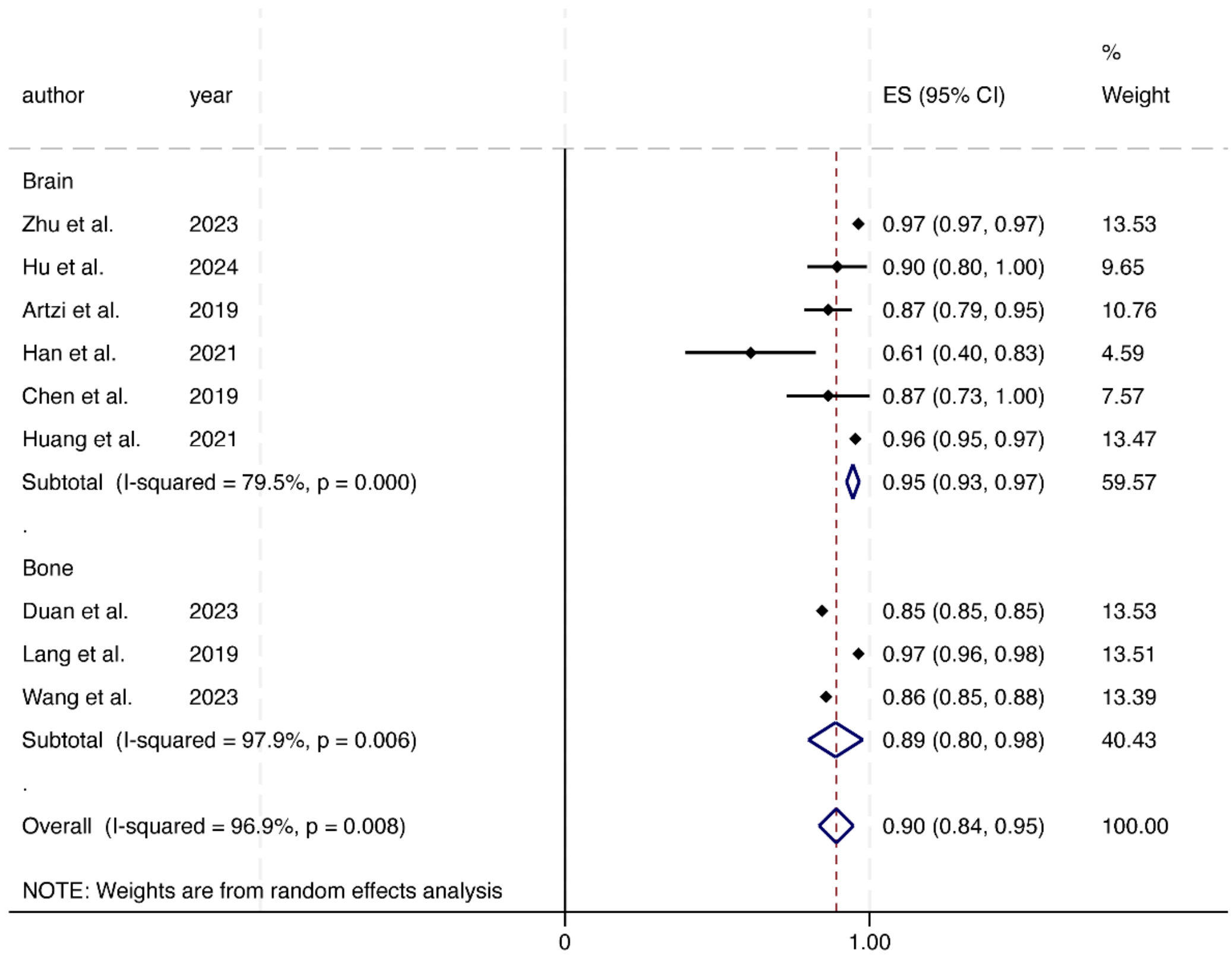

Supplementary Figure 31. Forest plot of pooled Metastasis differentiation performance (specificity) stratified by metastatic lesion subgroup.

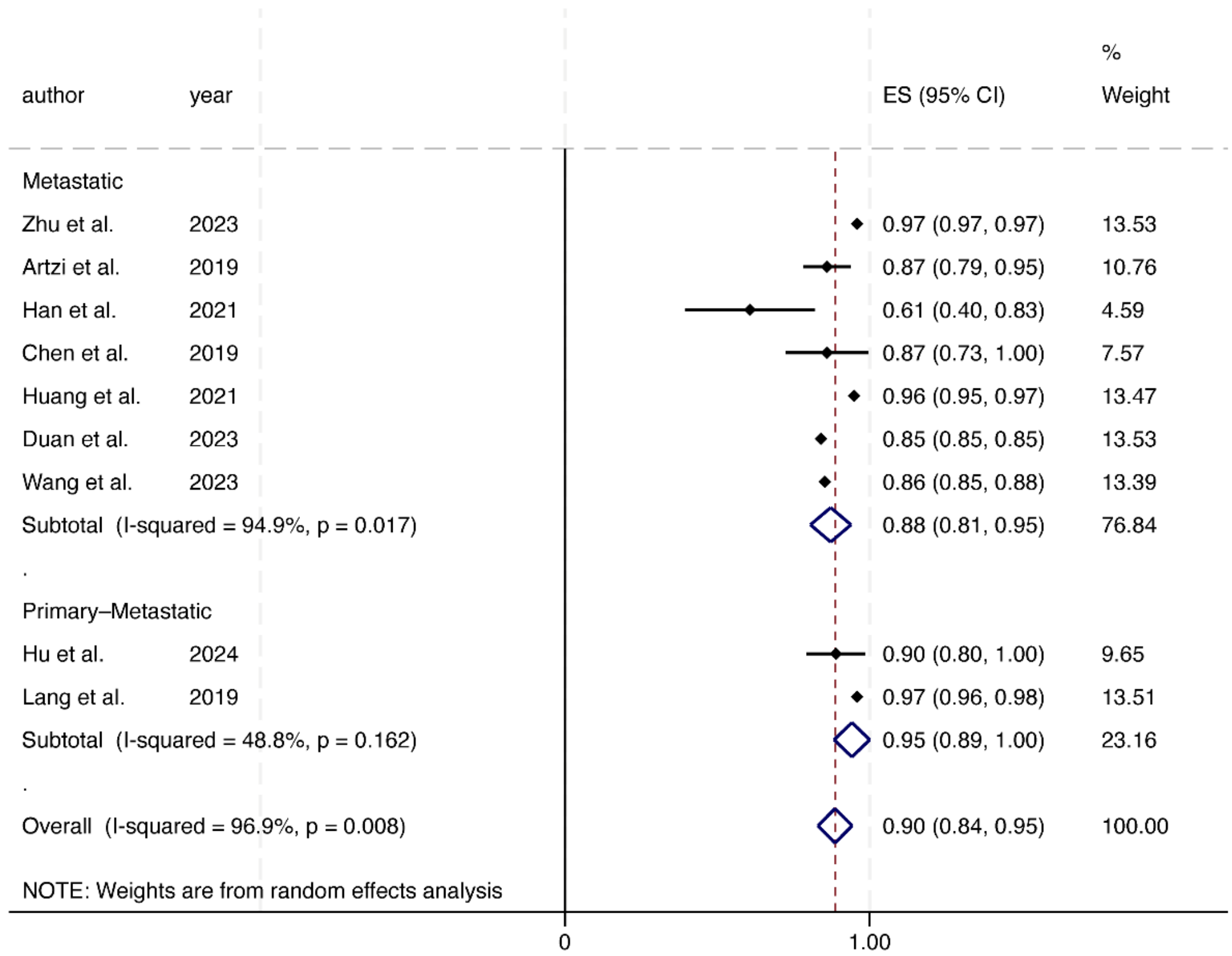

Supplementary Figure 32. Forest plot of pooled Metastasis differentiation performance (specificity) stratified by lesion-level ROI subgroup.

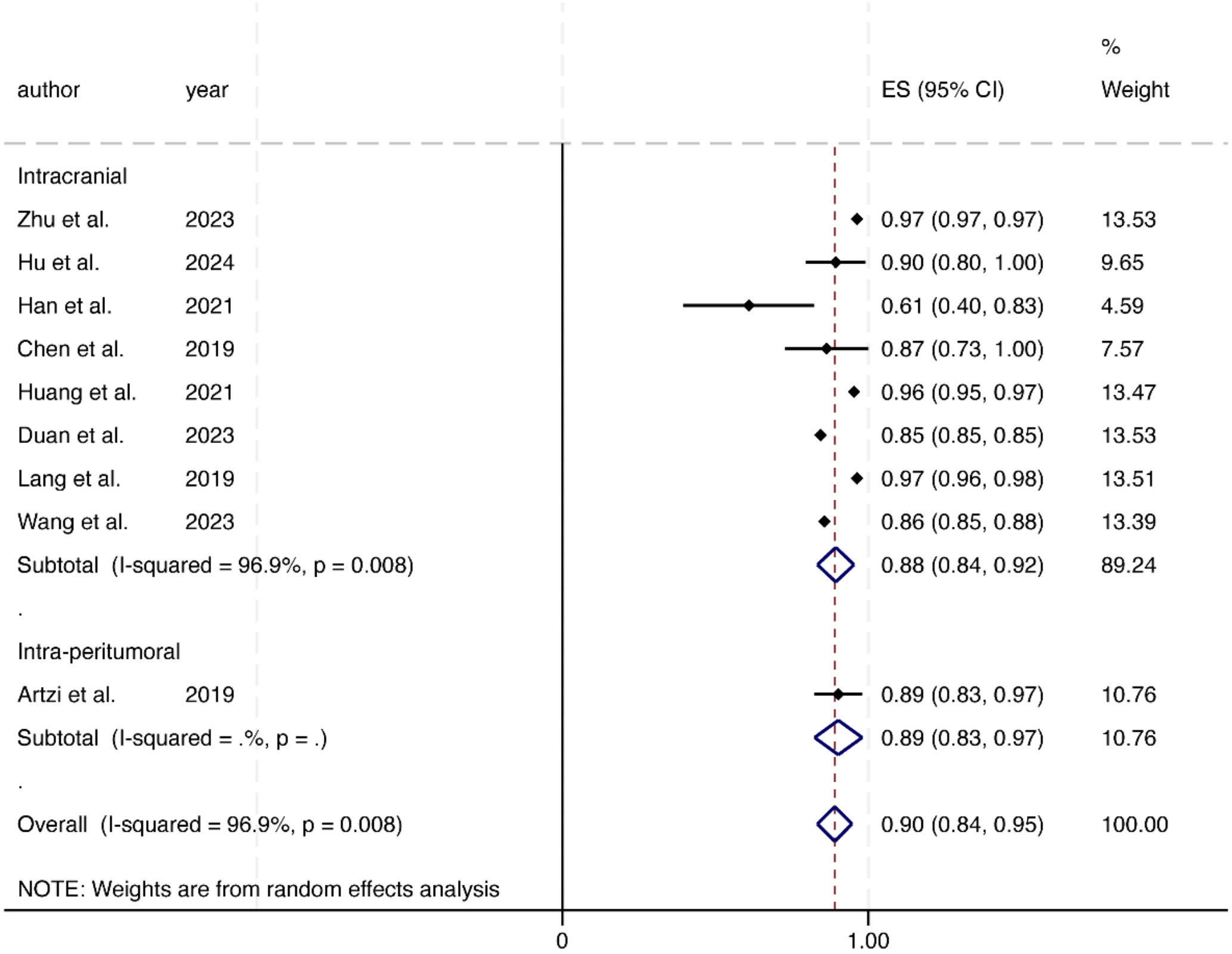

Supplementary Figure 33. Forest plot of pooled Metastasis differentiation performance (specificity) stratified by local metastatic ROI subgroup.

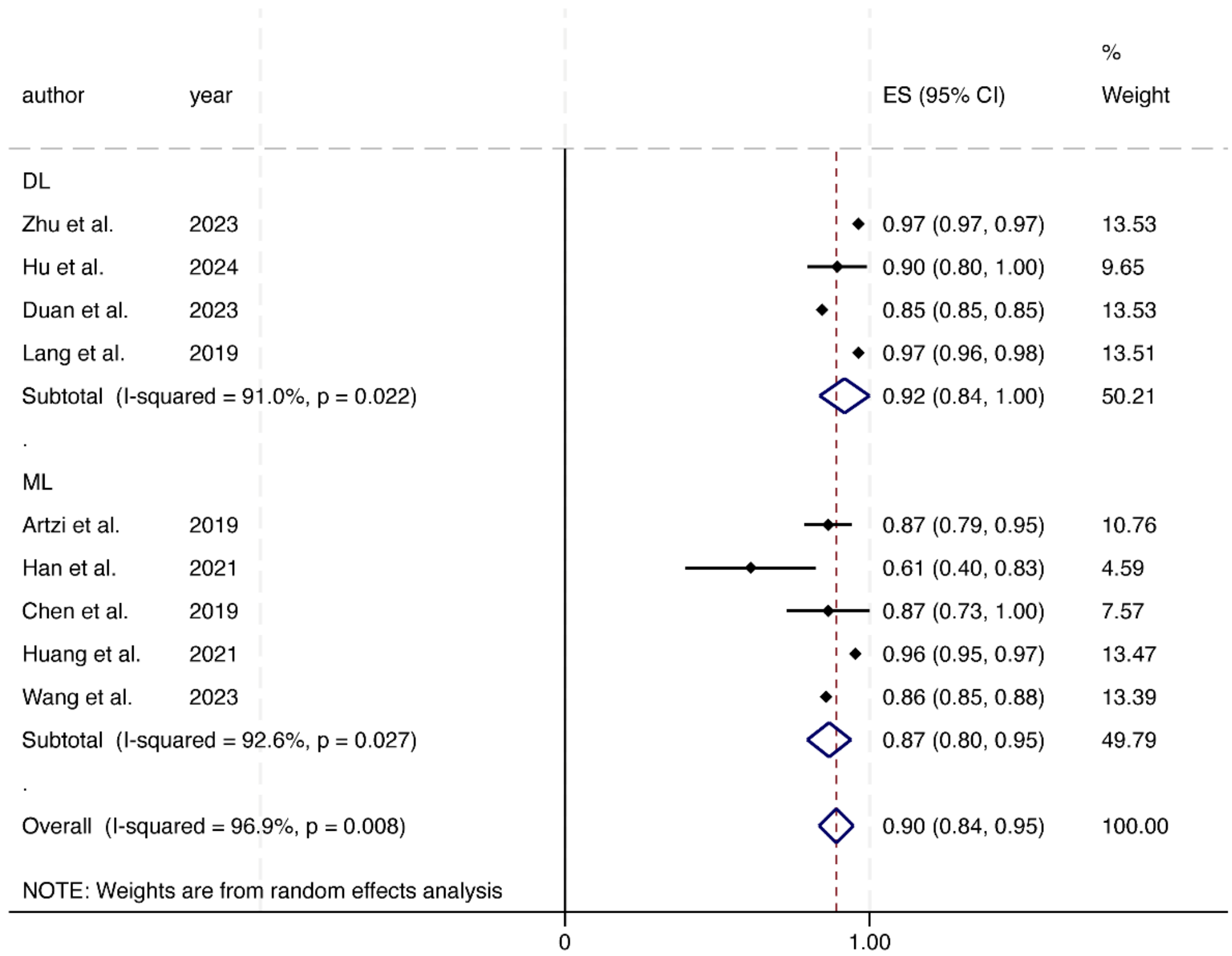

Supplementary Figure 34. Forest plot of pooled Metastasis differentiation performance (specificity) stratified by algorithm type (ML vs. DL).

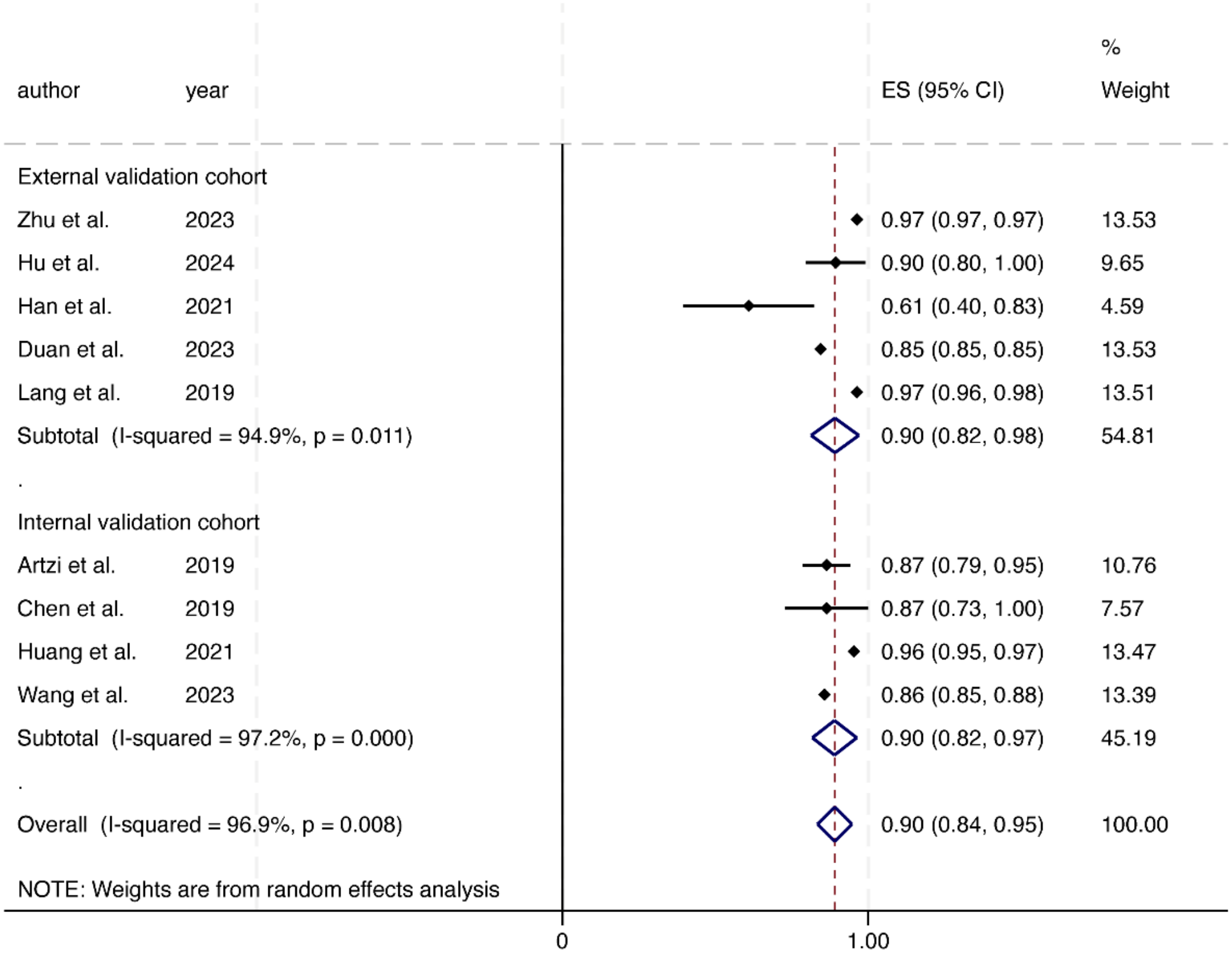

Supplementary Figure 35. Forest plot of pooled Metastasis differentiation performance (specificity) stratified by cohort type (internal vs. external).

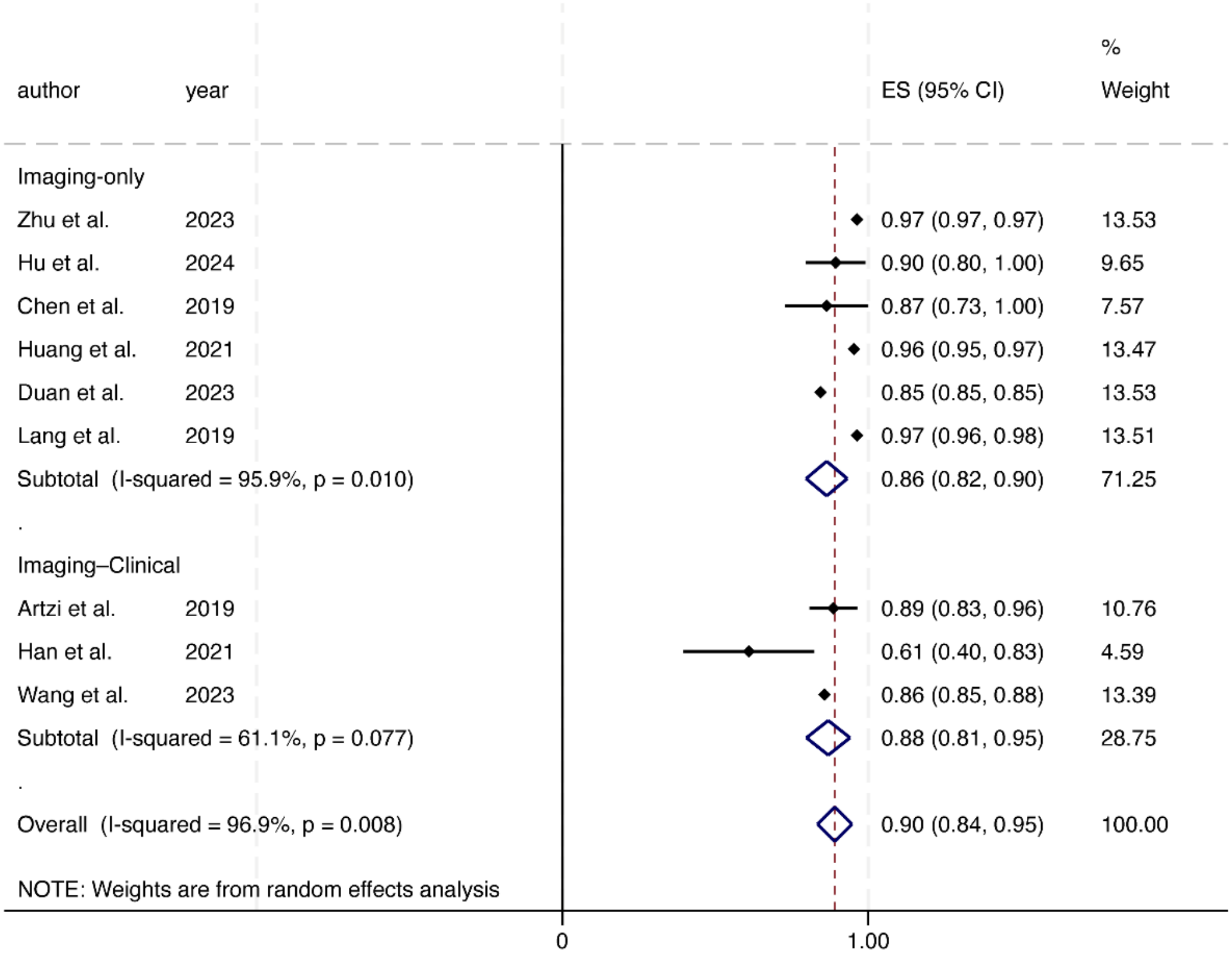

Supplementary Figure 36. Forest plot of pooled Metastasis differentiation performance (specificity) stratified by feature-source subgroup.

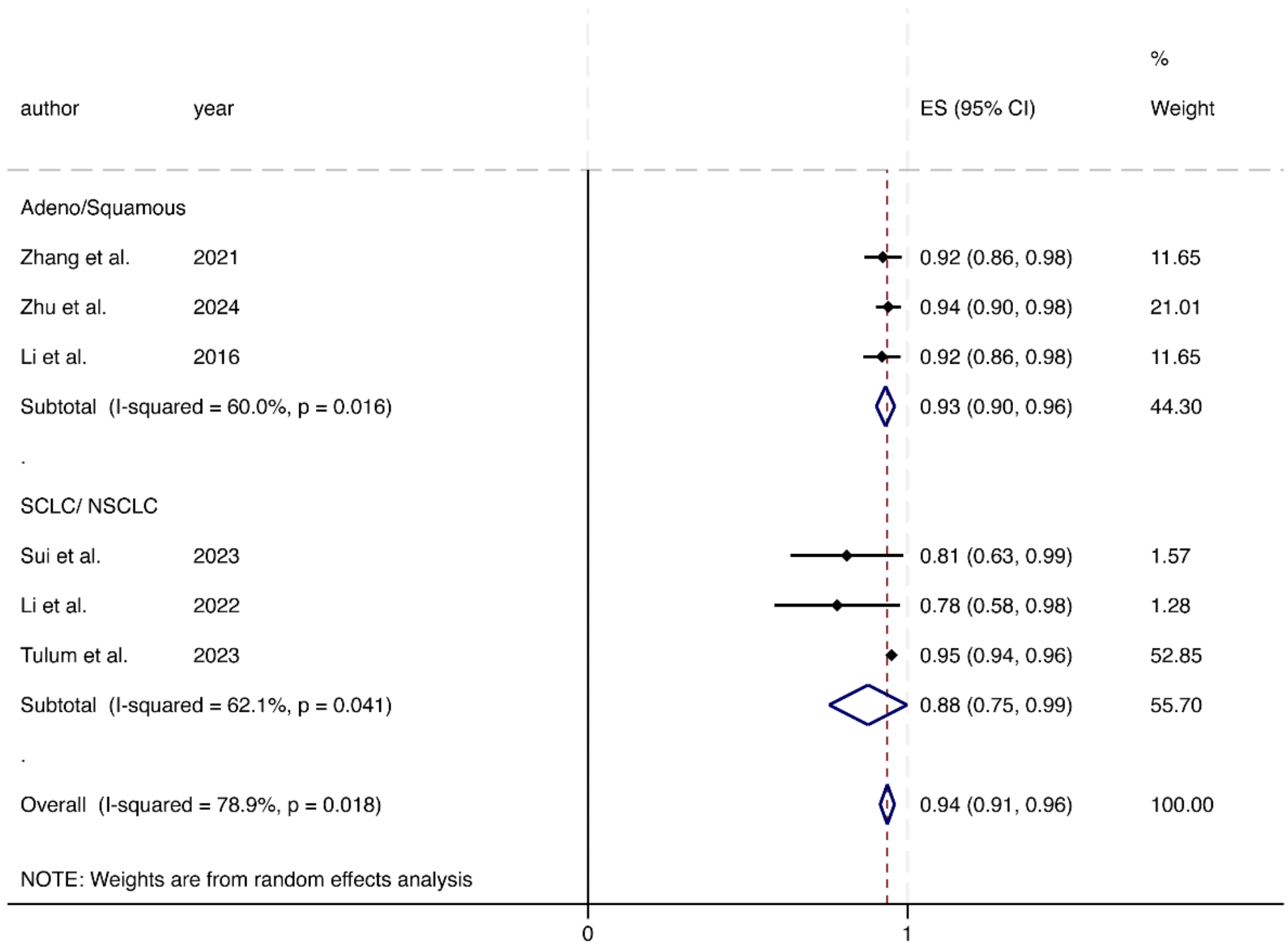

Supplementary Figure 37. Forest plot of pooled Tumor type classification performance (specificity) stratified by histologic subtype classification.

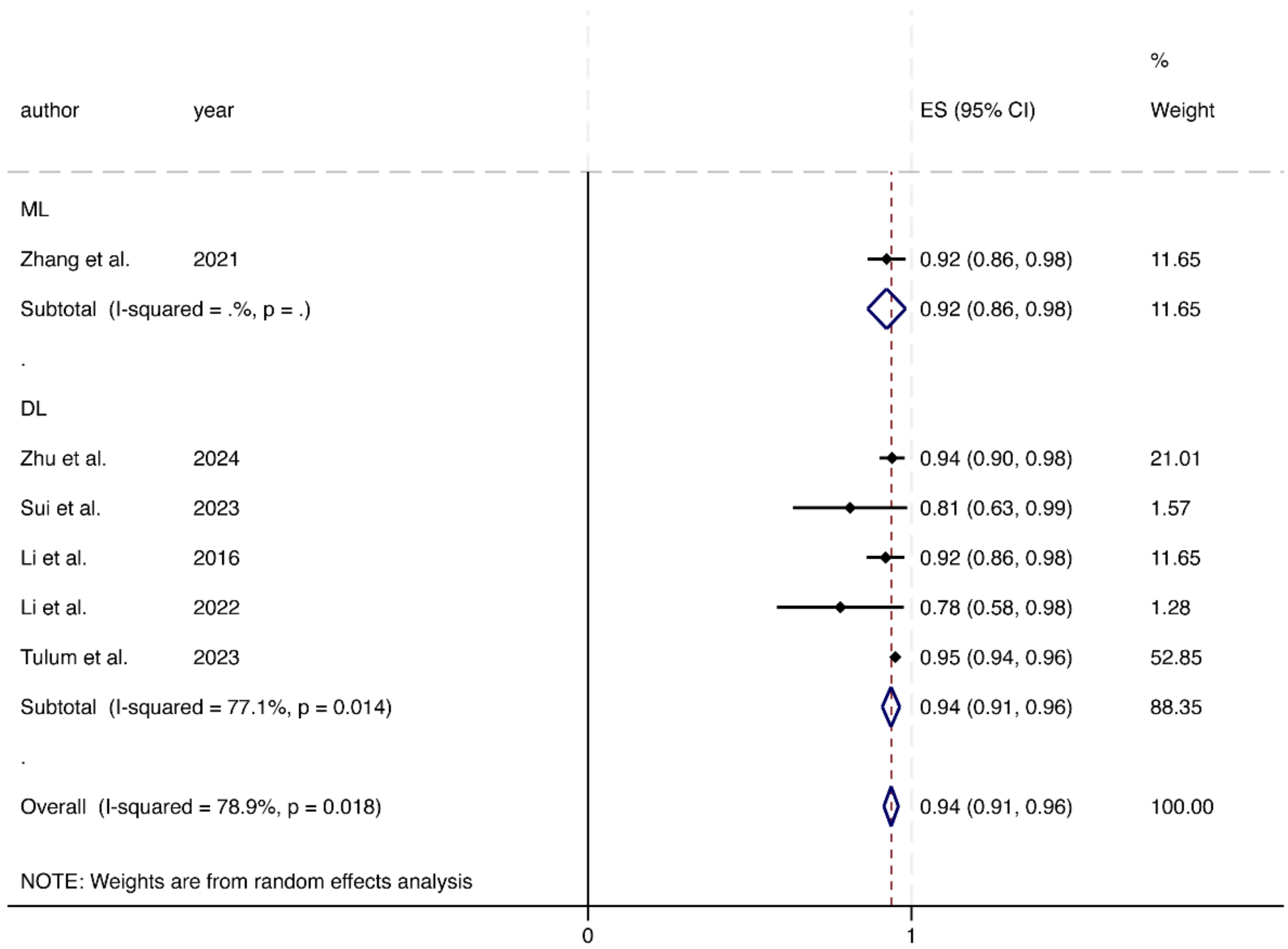

Supplementary Figure 38. Forest plot of pooled Tumor type classification performance (specificity) stratified by algorithm type (ML vs. DL).

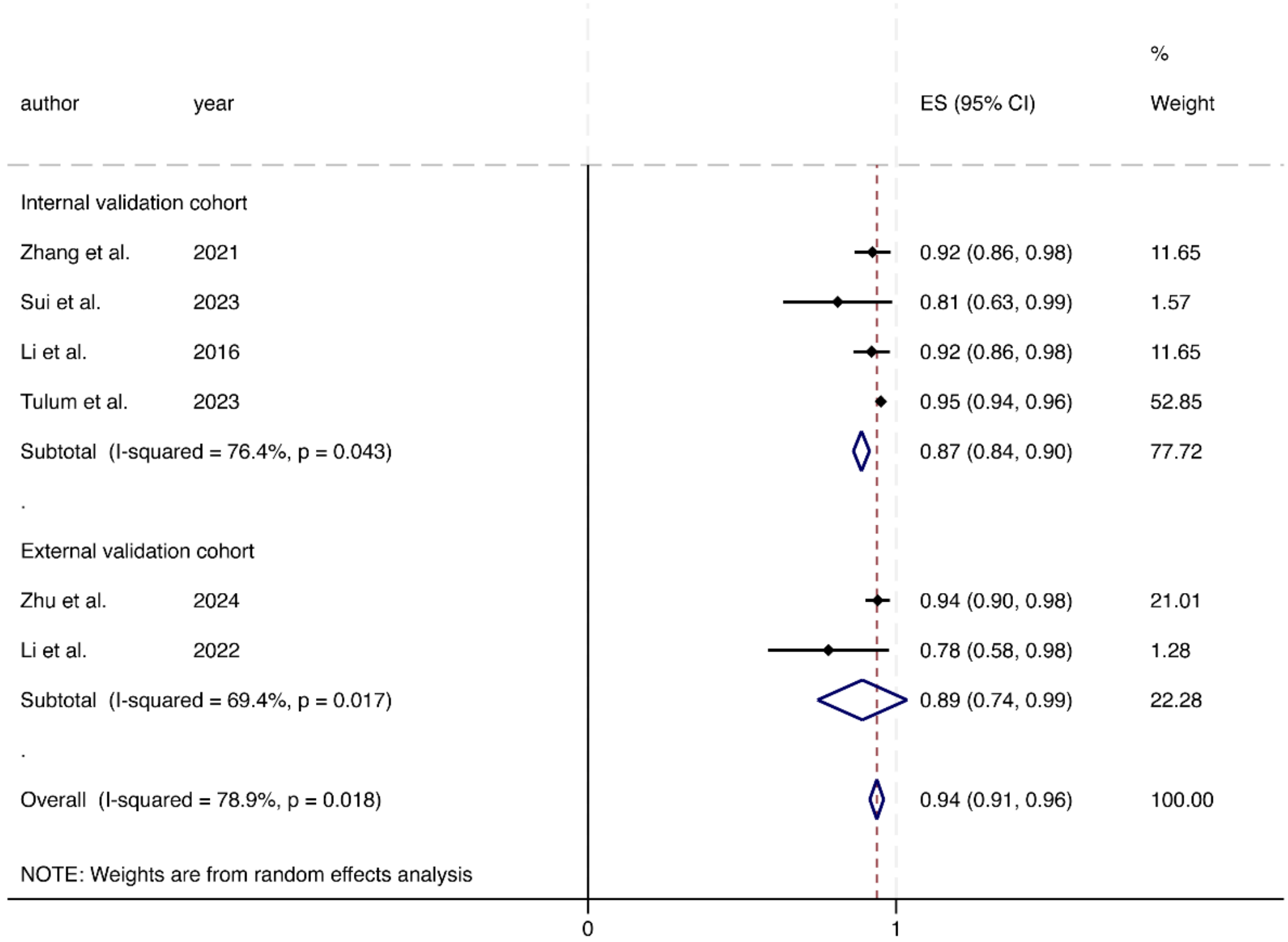

Supplementary Figure 39. Forest plot of pooled Tumor type classification performance (specificity) stratified by cohort type (internal vs. external).

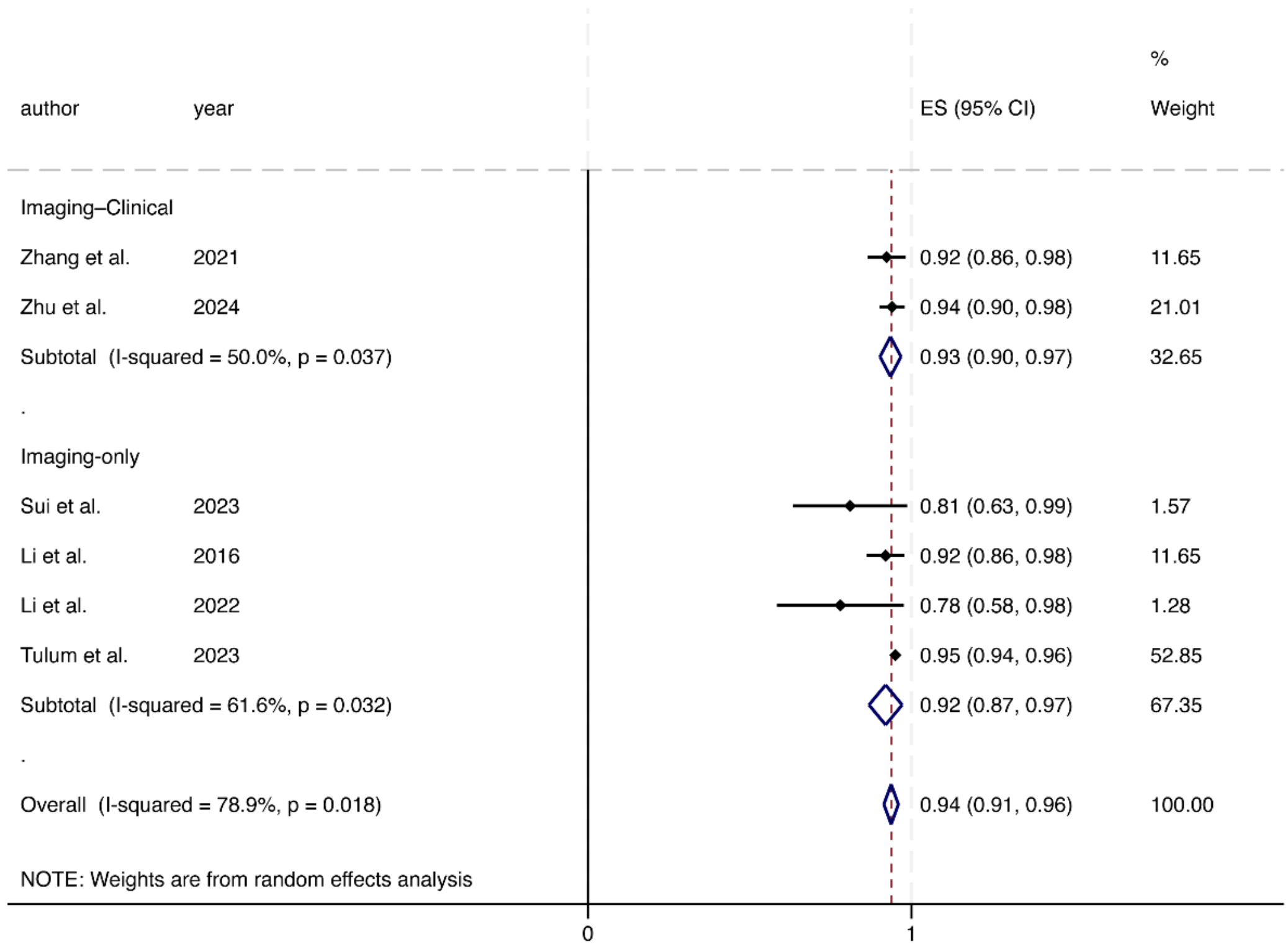

Supplementary Figure 40. Forest plot of pooled Tumor type classification performance (specificity) stratified by feature-source subgroup.

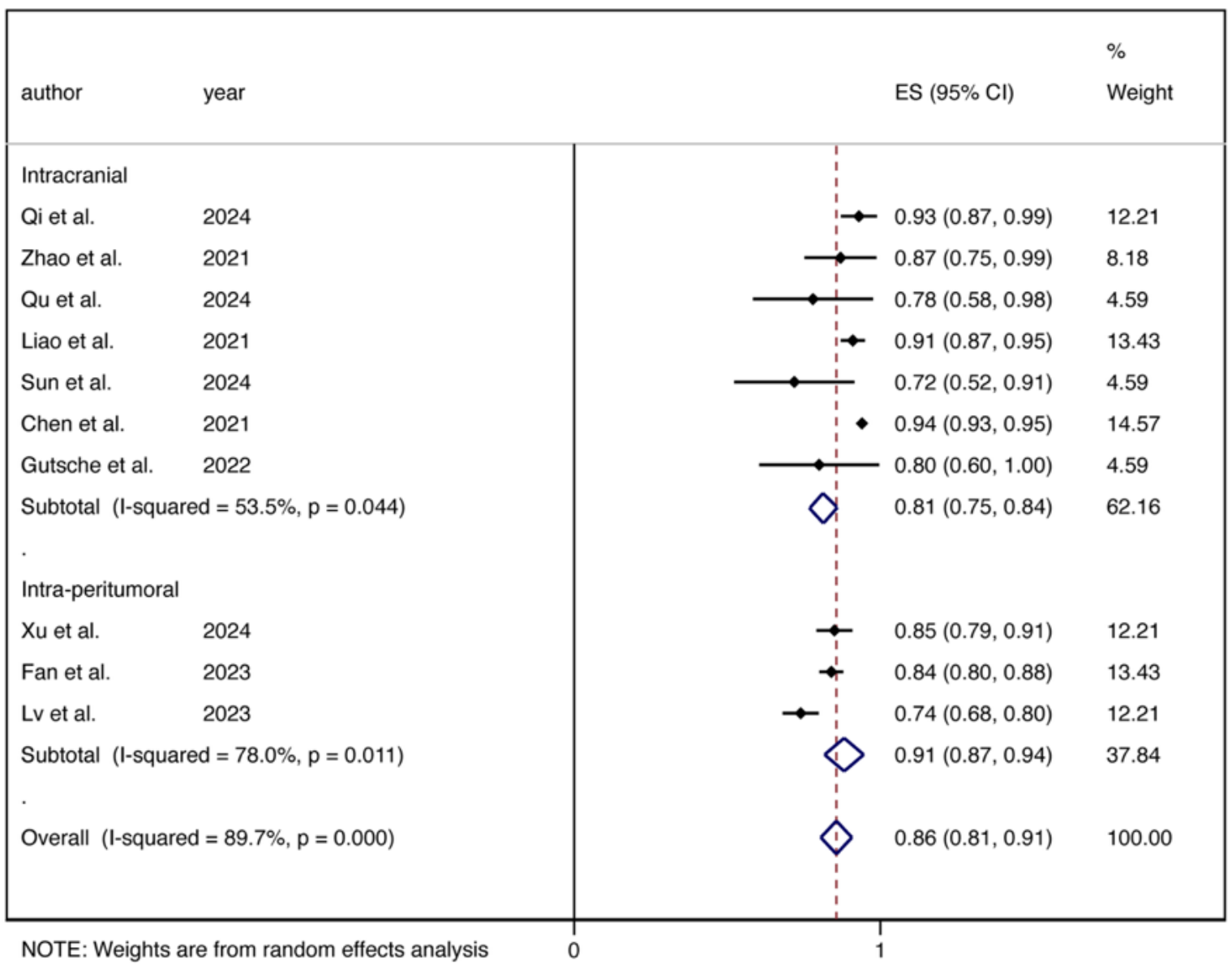

Supplementary Figure 41. Forest plot of pooled Outcome prediction (specificity) stratified by local metastatic ROI subgroup.

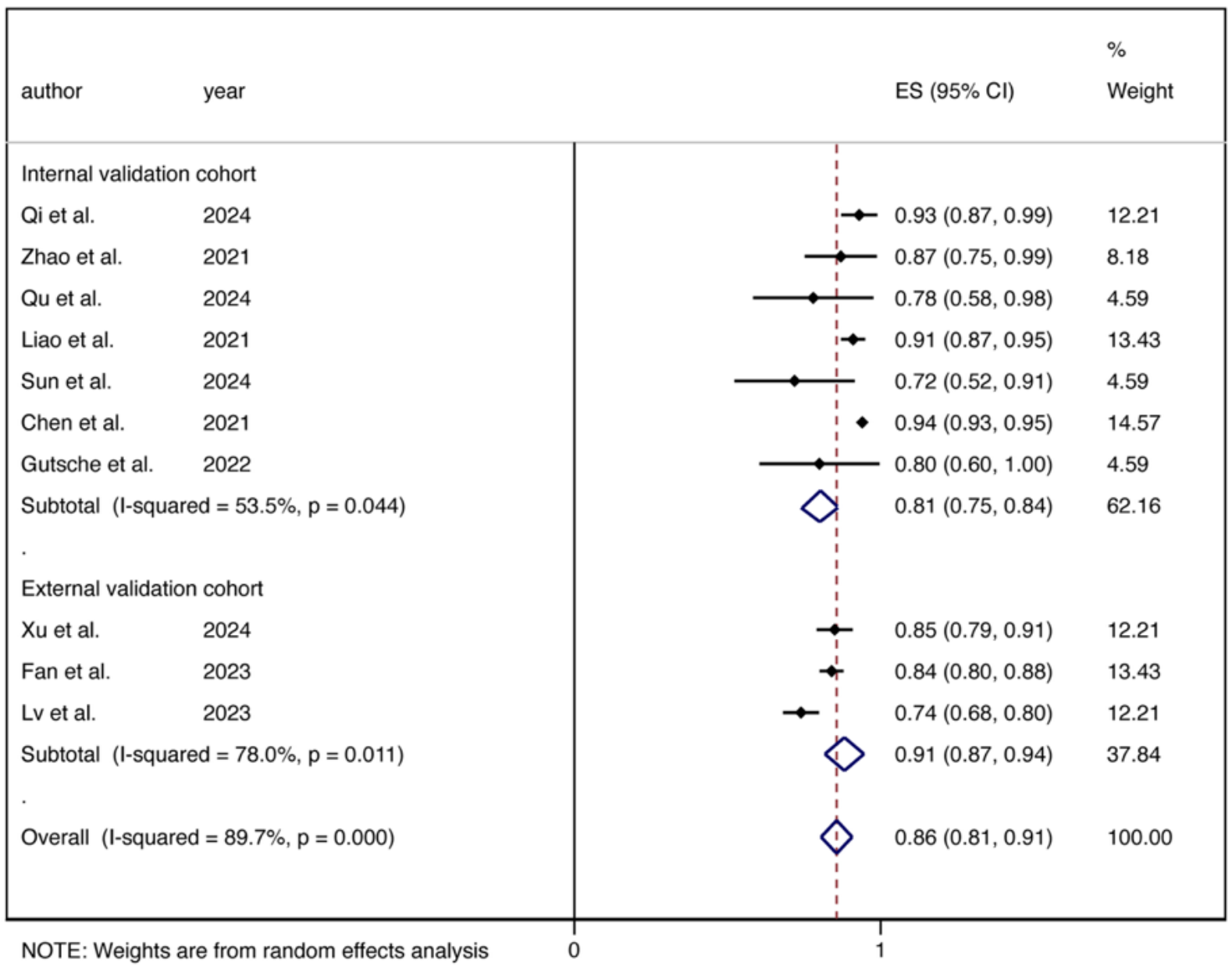

Supplementary Figure 42. Forest plot of pooled Outcome prediction (specificity) stratified by cohort type (internal vs. external).

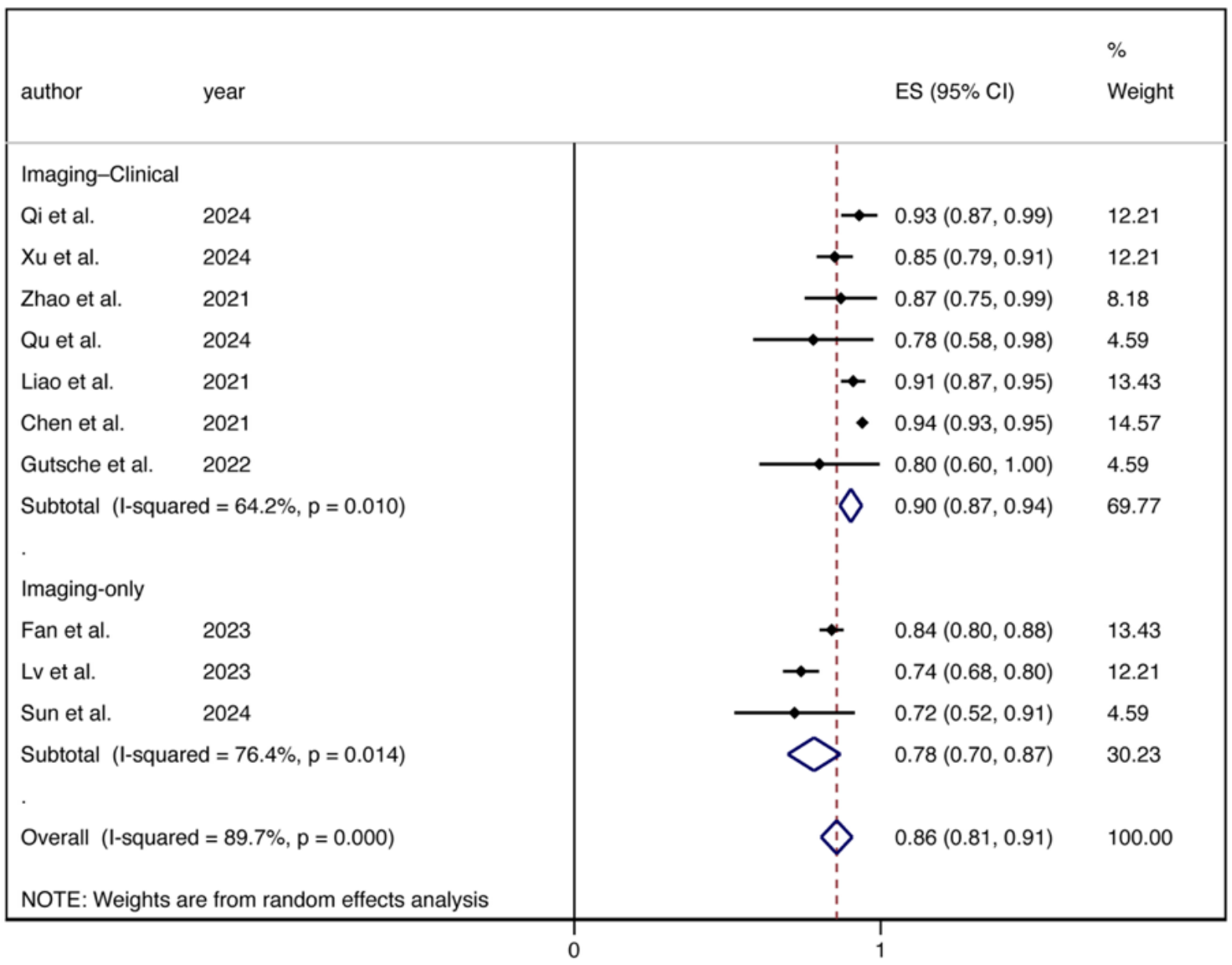

Supplementary Figure 43. Forest plot of pooled Outcome prediction (specificity) stratified by feature-source subgroup.

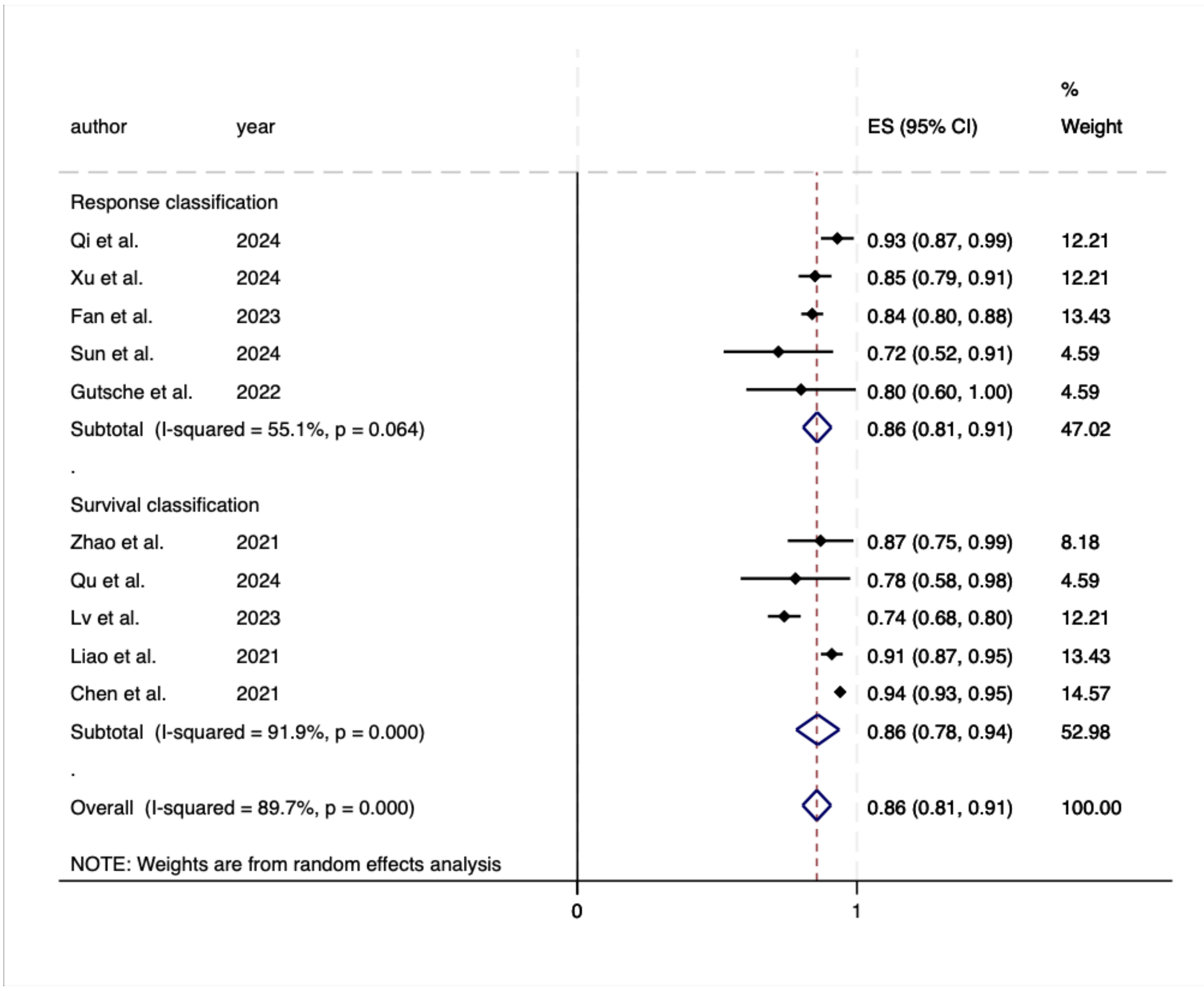

Supplementary Figure 44. Forest plot of pooled Outcome prediction (specificity) stratified by prognostic type classification.

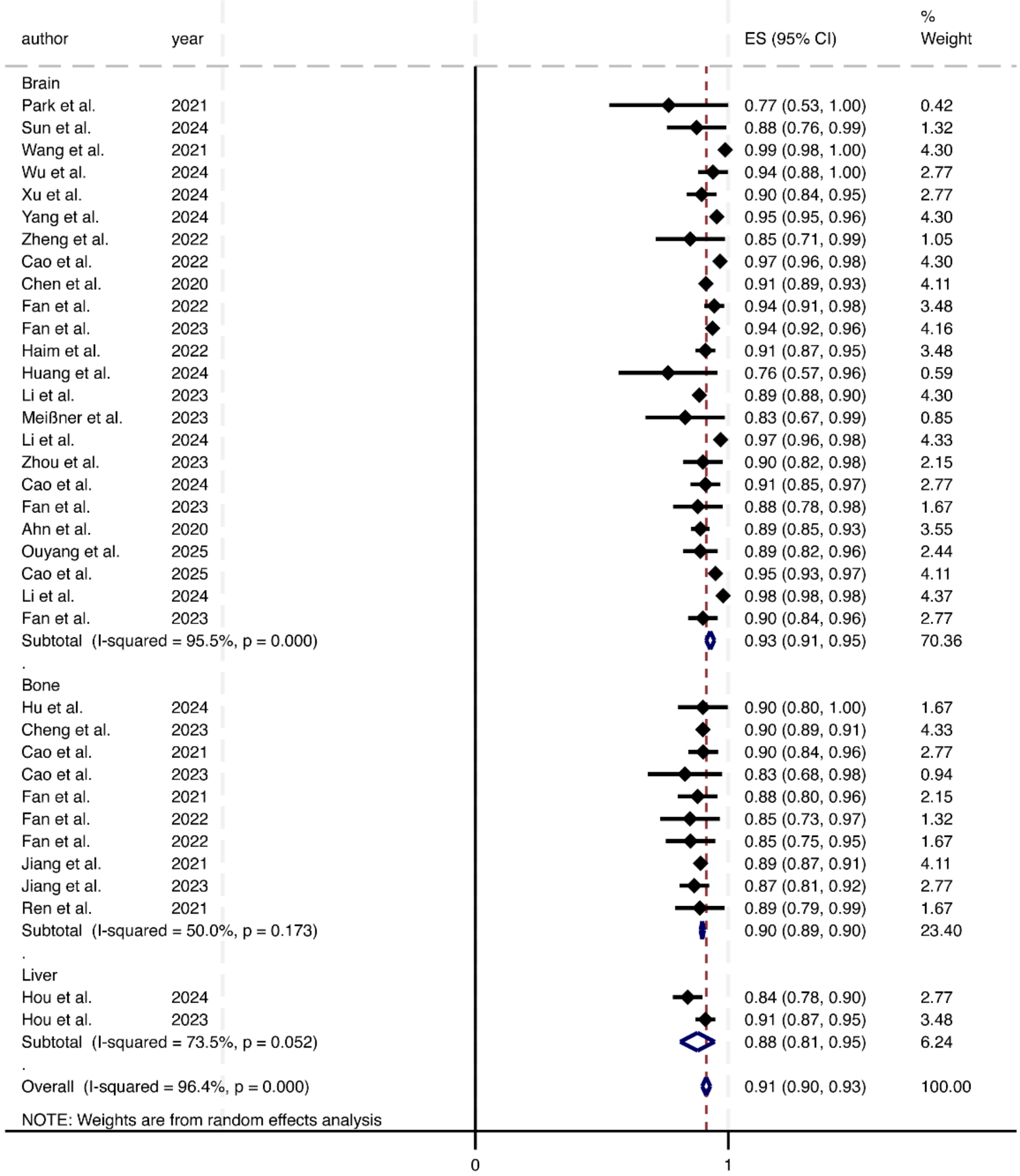

Supplementary Figure 45. Forest plot of pooled Molecular-level prediction performance (AUC) stratified by metastatic lesion subgroup.

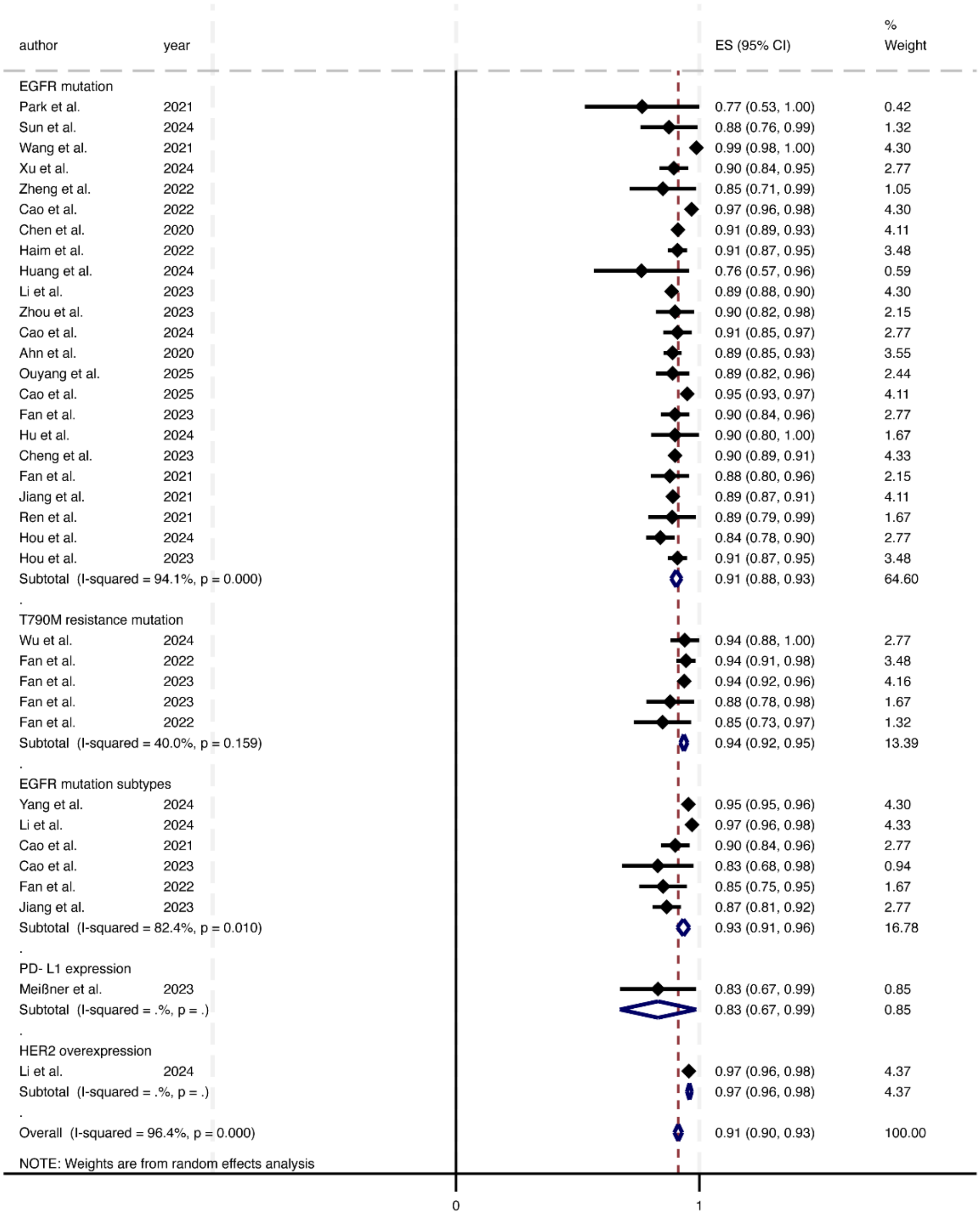

Supplementary Figure 46. Forest plot of pooled Molecular-level prediction performance (AUC) stratified by molecular subtype classification subgroup.

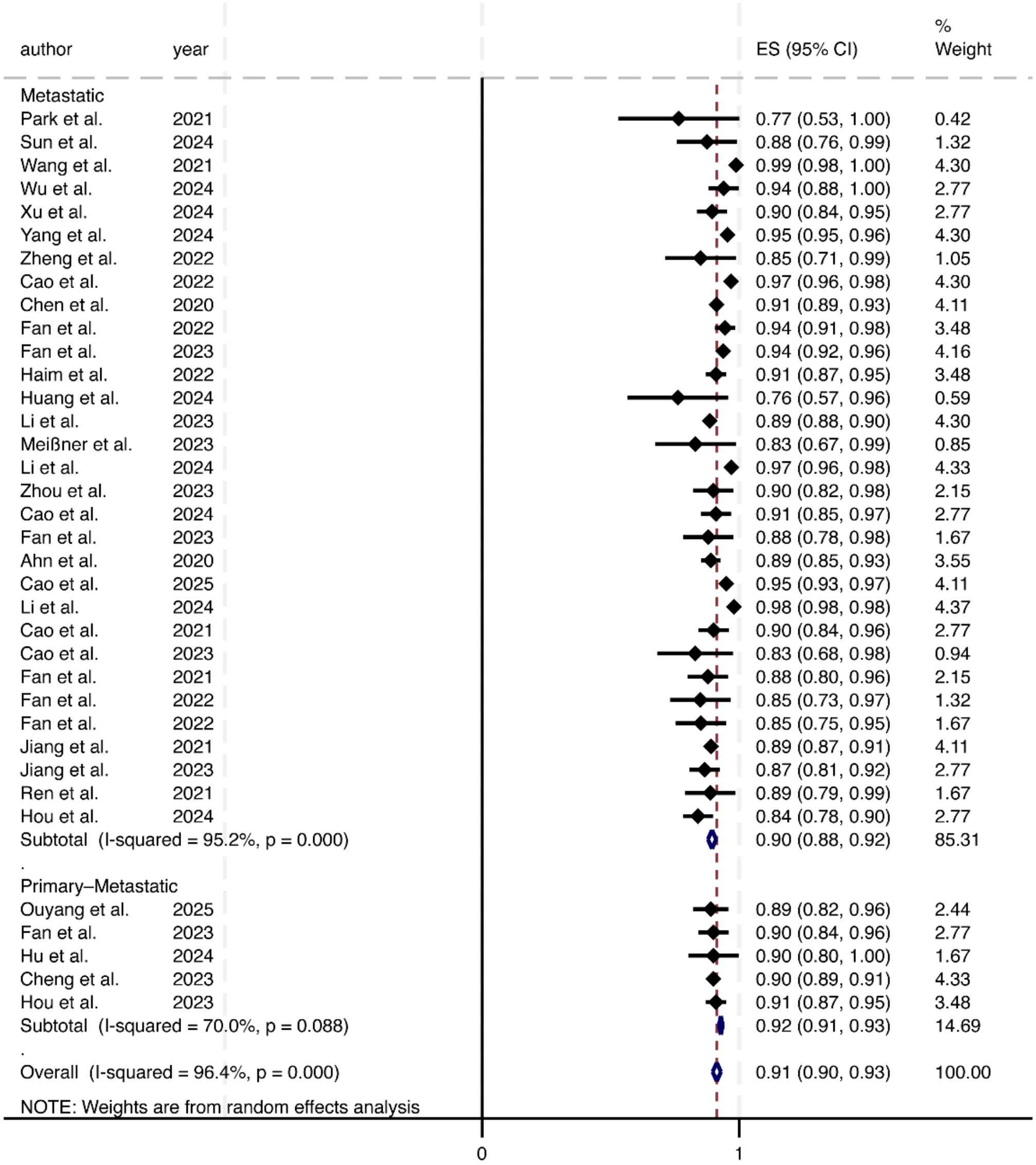

Supplementary Figure 47. Forest plot of pooled Molecular-level prediction performance (AUC) stratified by lesion-level ROI subgroup.

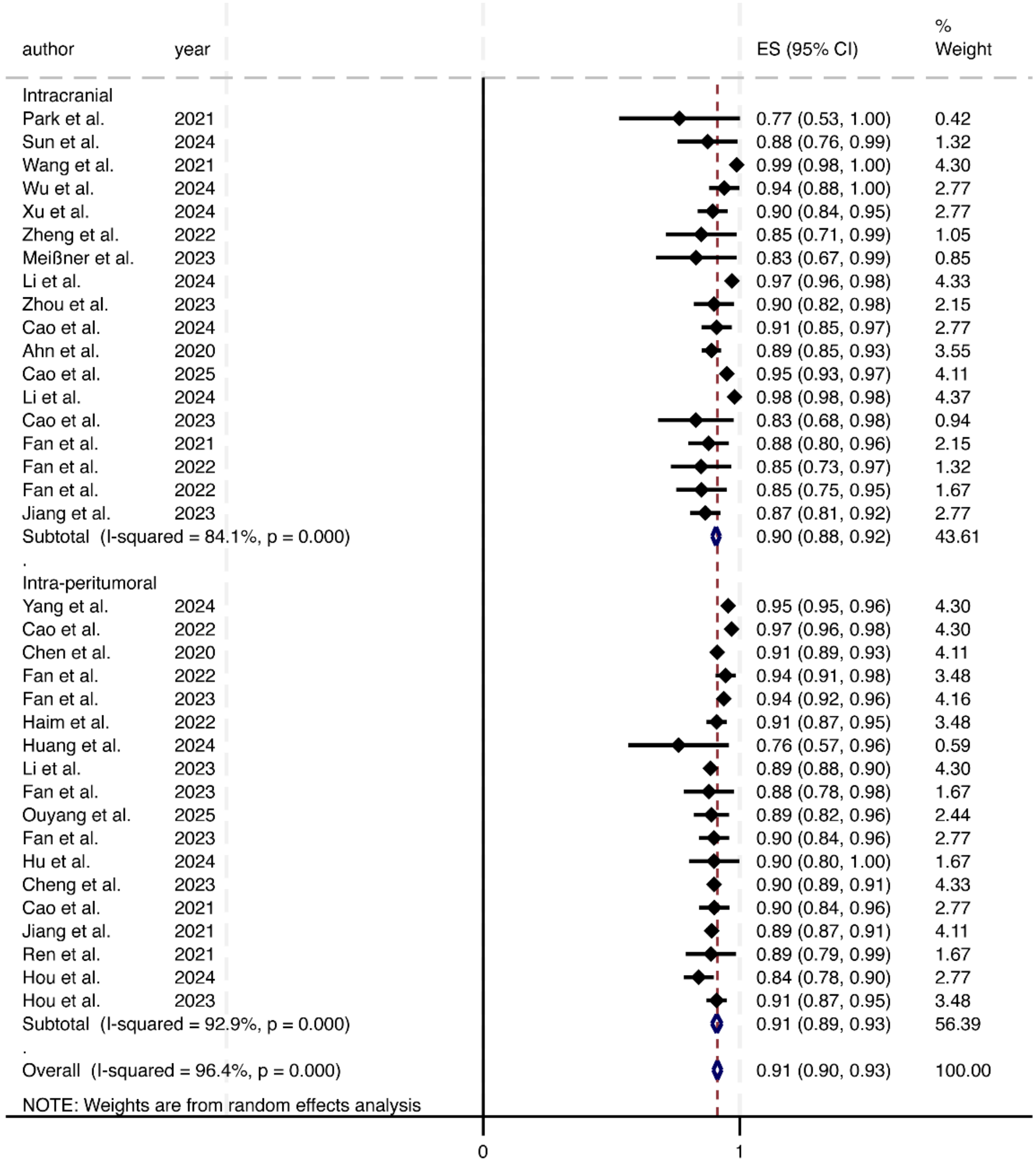

Supplementary Figure 48. Forest plot of pooled Molecular-level prediction performance (AUC) stratified by local metastatic ROI subgroup.

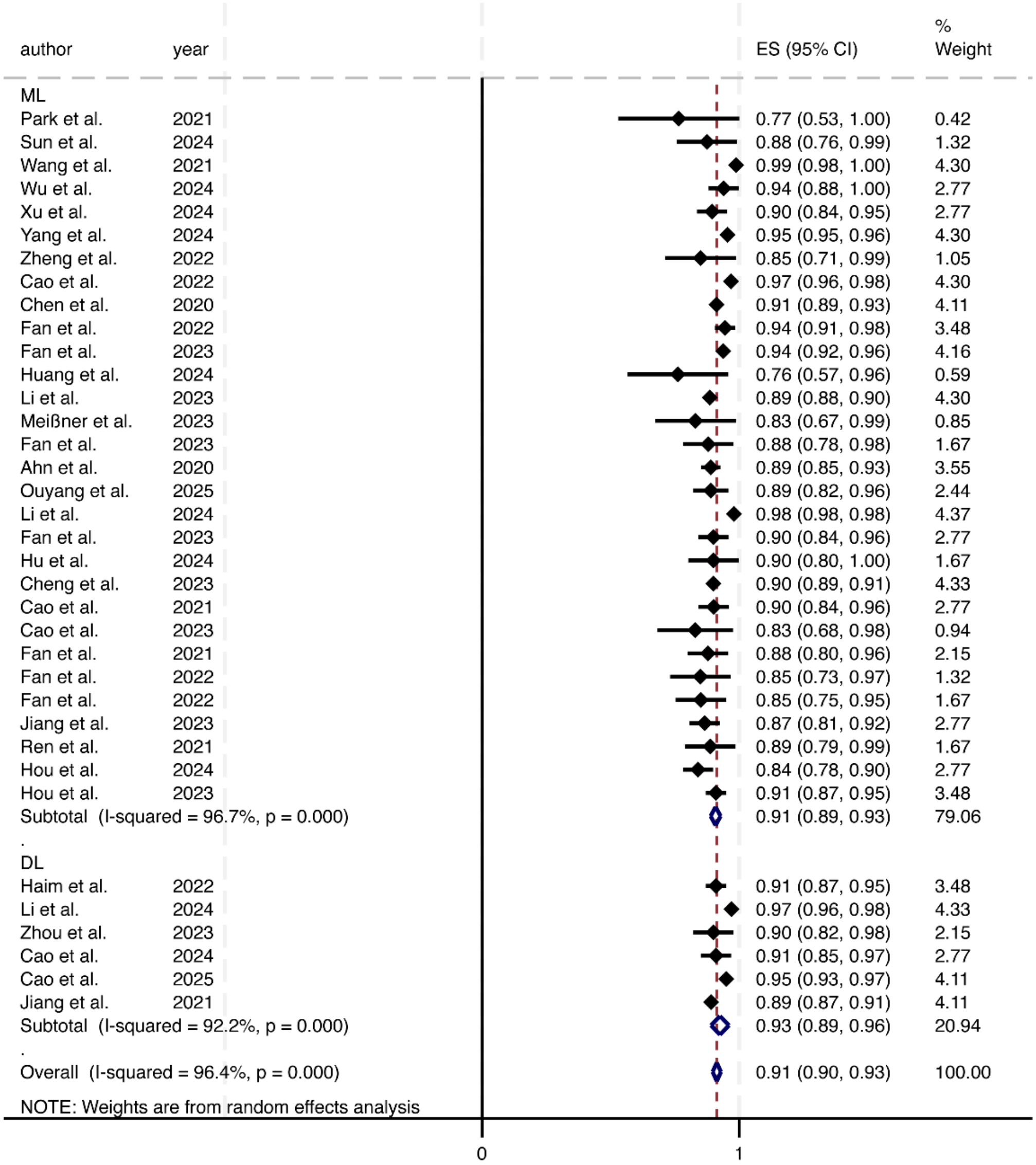

Supplementary Figure 49. Forest plot of pooled Molecular-level prediction performance (AUC) stratified by algorithm type (ML vs. DL).

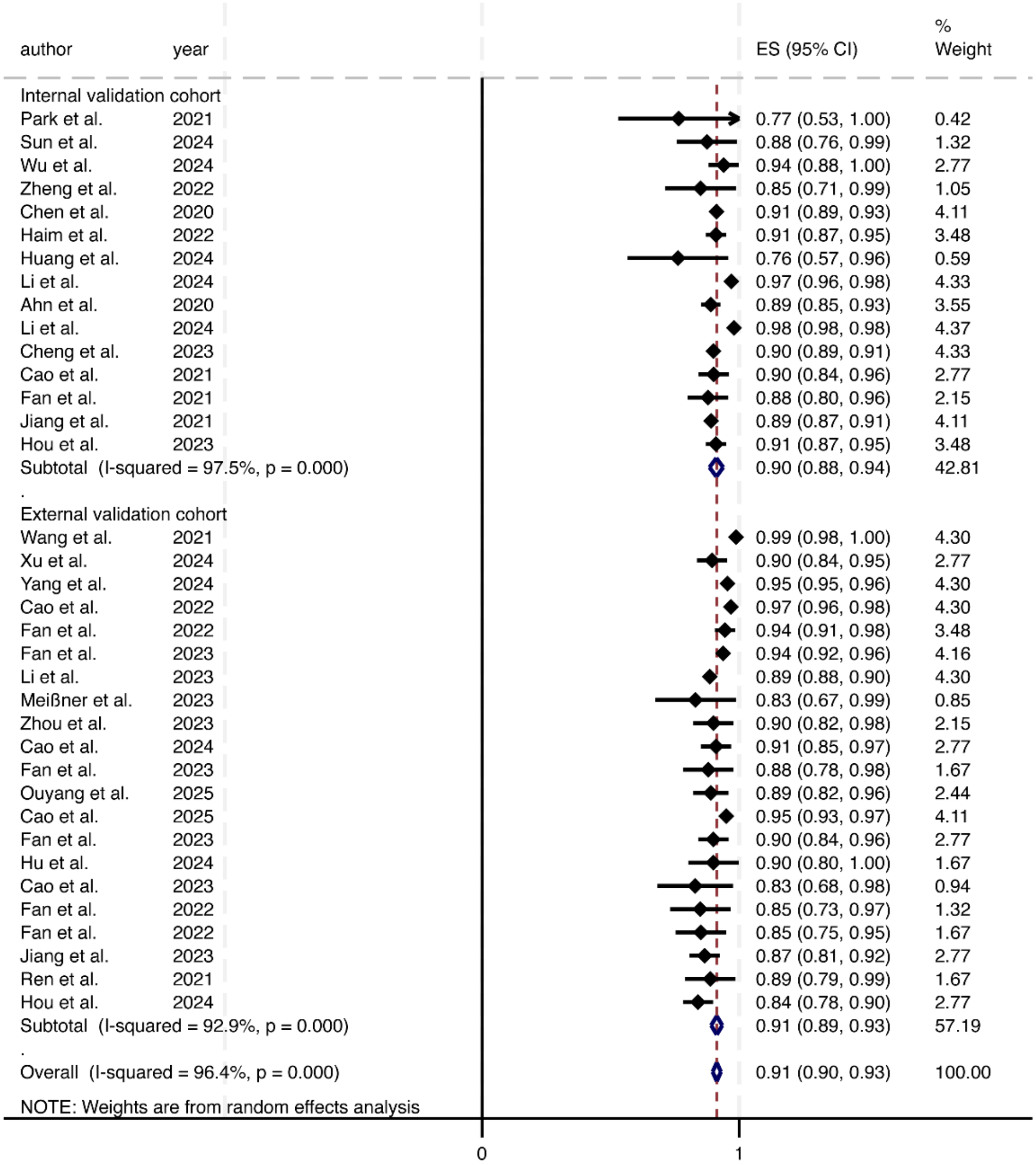

Supplementary Figure 50. Forest plot of pooled Molecular-level prediction performance (AUC) stratified by cohort type (internal vs. external).

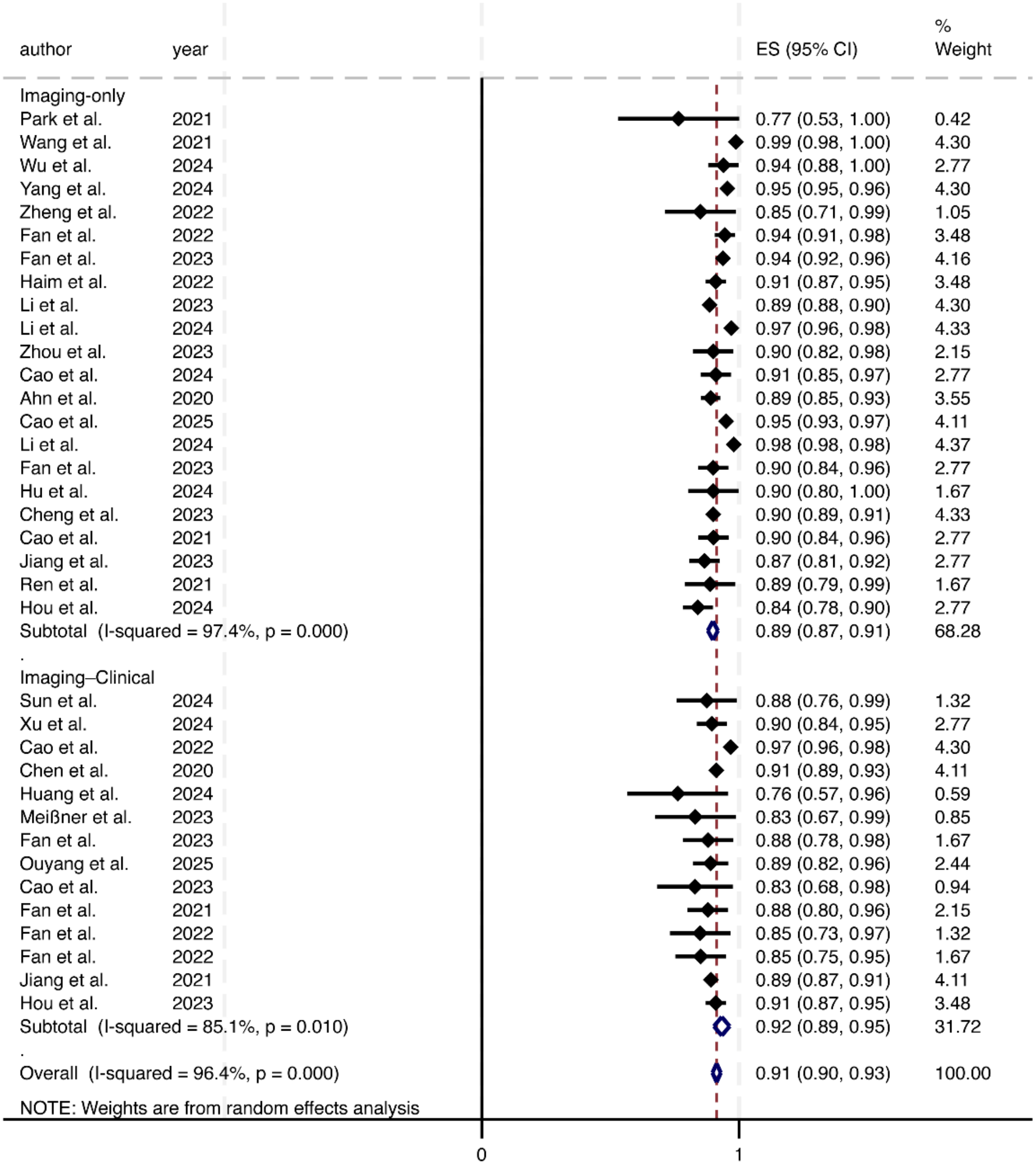

Supplementary Figure 51. Forest plot of pooled Molecular-level prediction performance (AUC) stratified by feature-source subgroup.

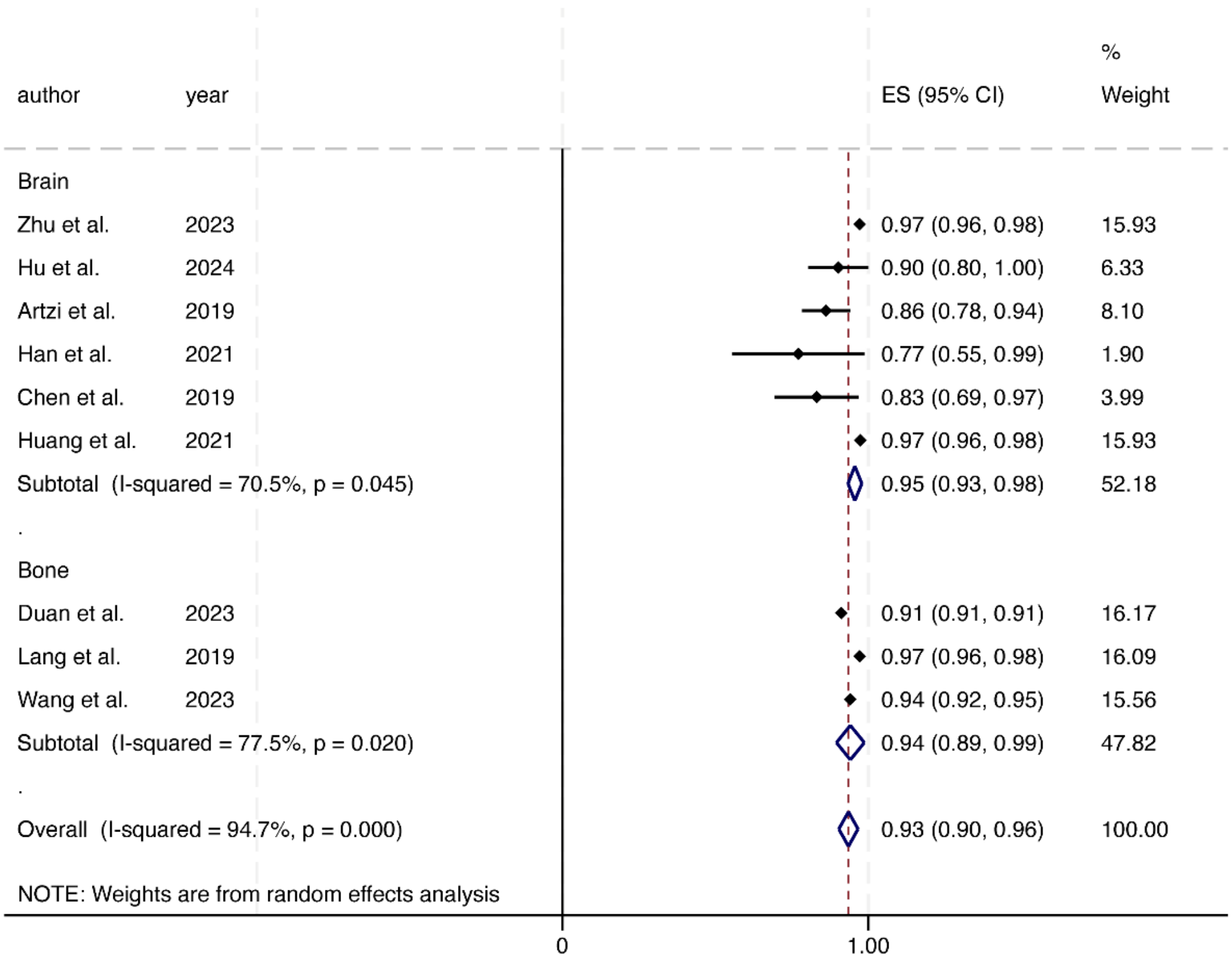

Supplementary Figure 52. Forest plot of pooled Metastasis differentiation performance (AUC) stratified by metastatic lesion subgroup.

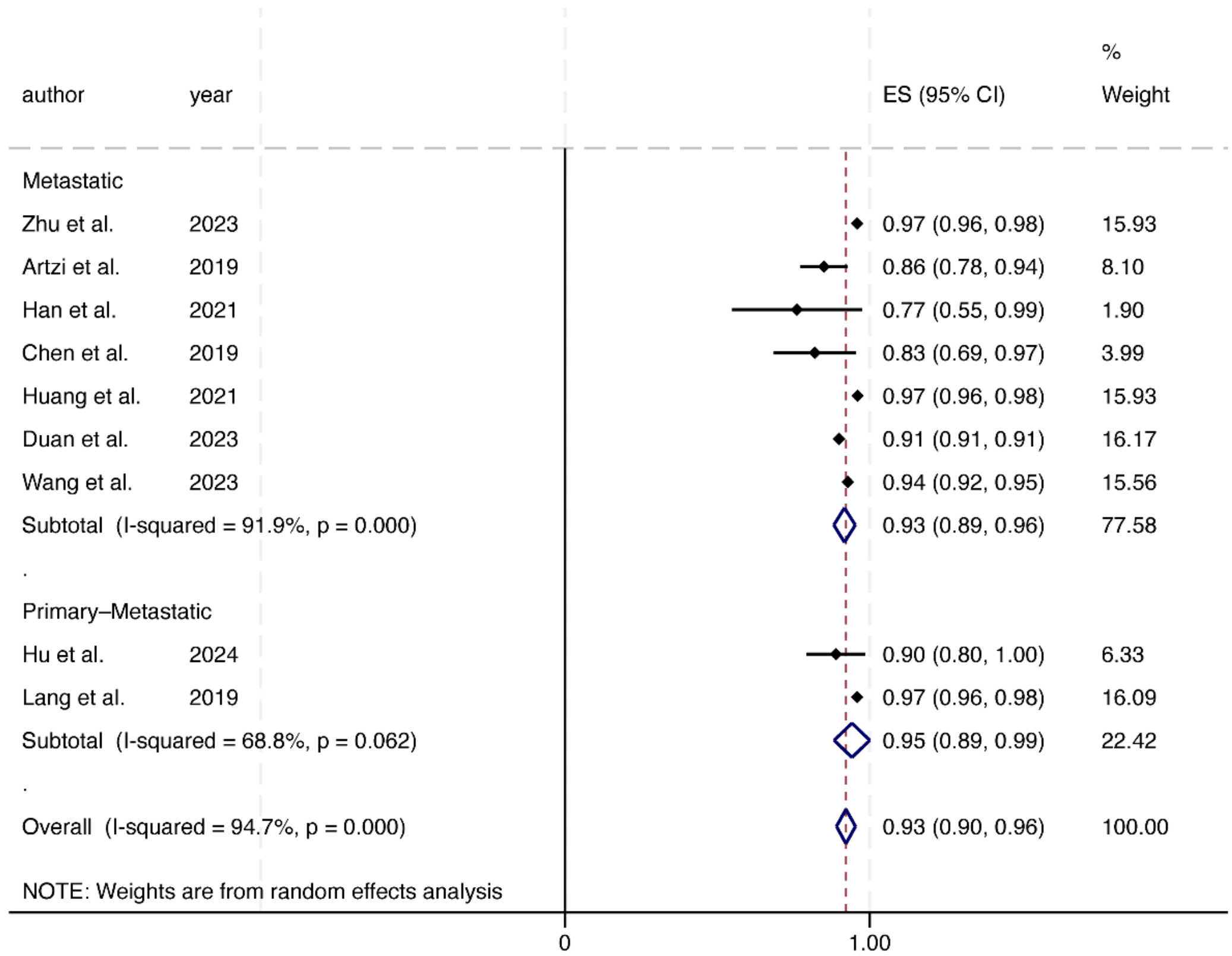

Supplementary Figure 53. Forest plot of pooled Metastasis differentiation performance (AUC) stratified by lesion-level ROI subgroup.

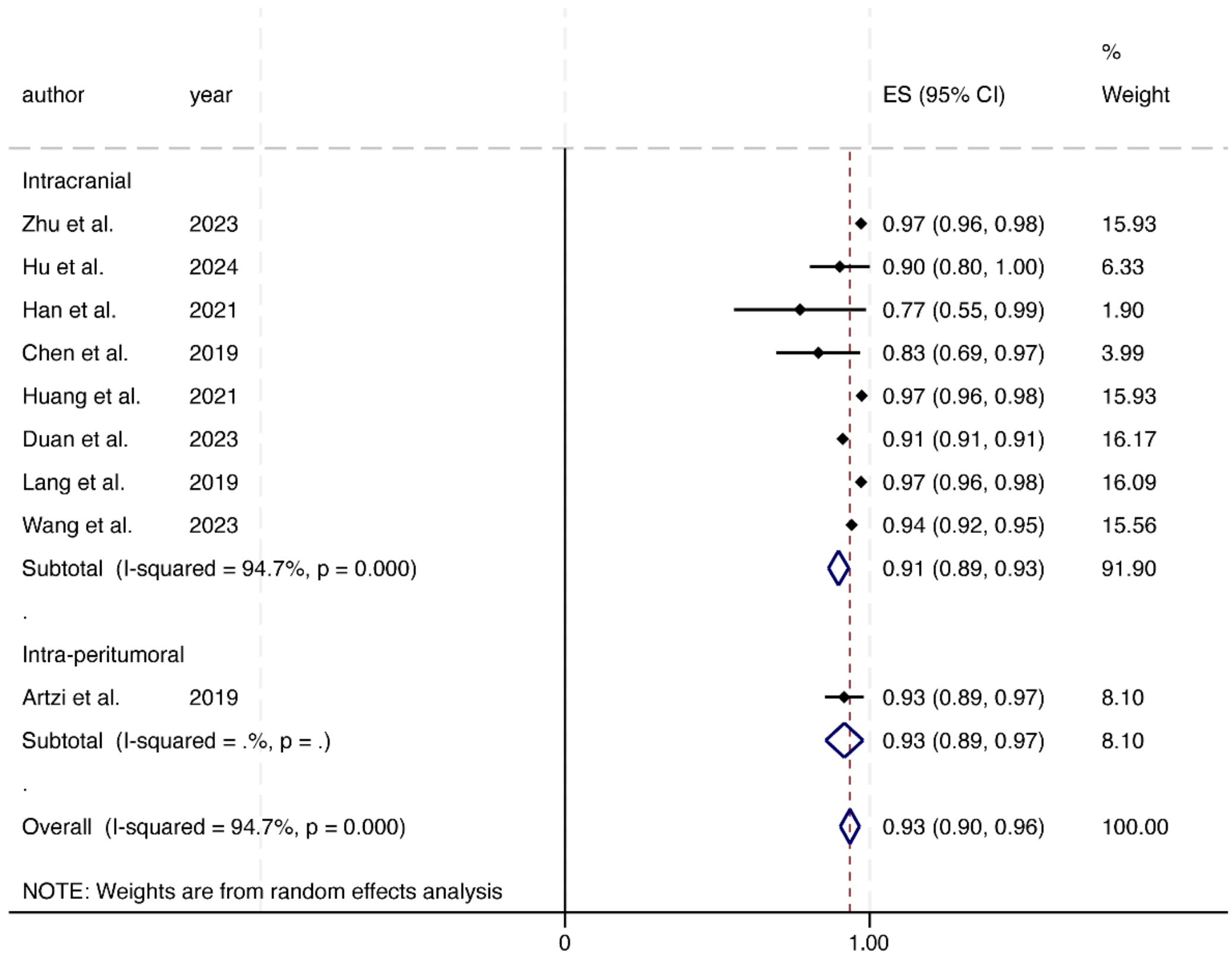

Supplementary Figure 54. Forest plot of pooled Metastasis differentiation performance (AUC) stratified by local metastatic ROI subgroup.

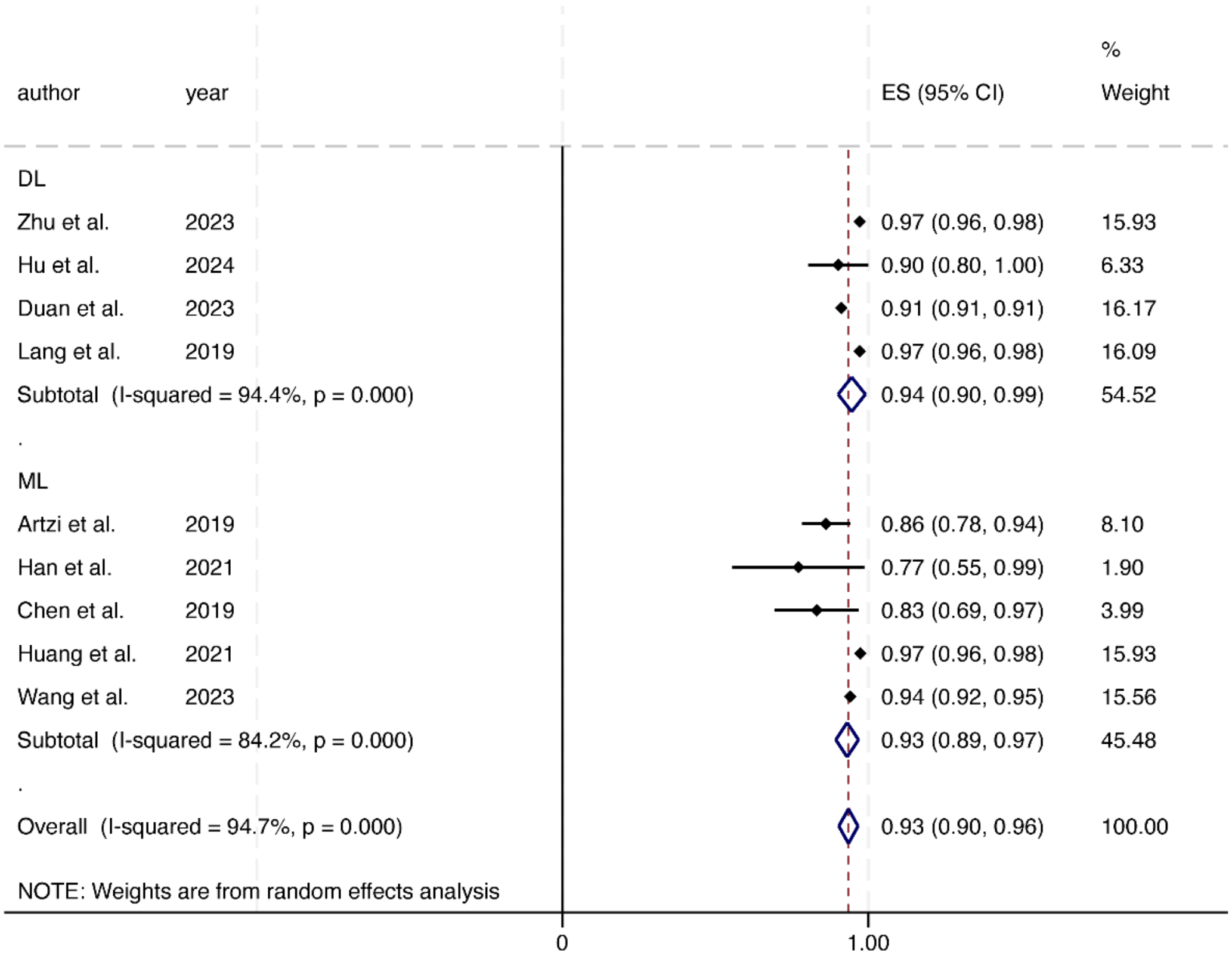

Supplementary Figure 55. Forest plot of pooled Metastasis differentiation performance (AUC) stratified by algorithm type (ML vs. DL).

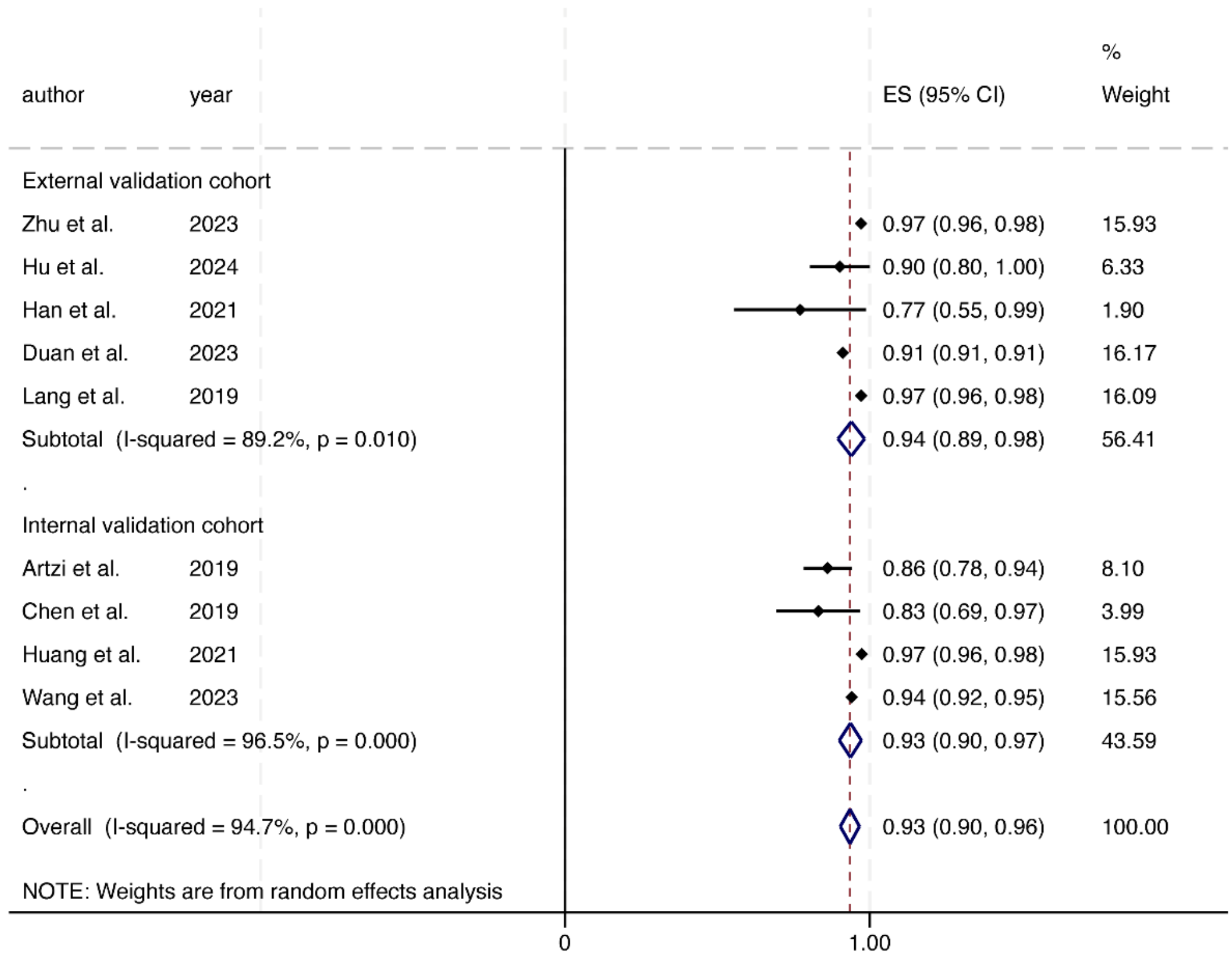

Supplementary Figure 56. Forest plot of pooled Metastasis differentiation performance (AUC) stratified by cohort type (internal vs. external).

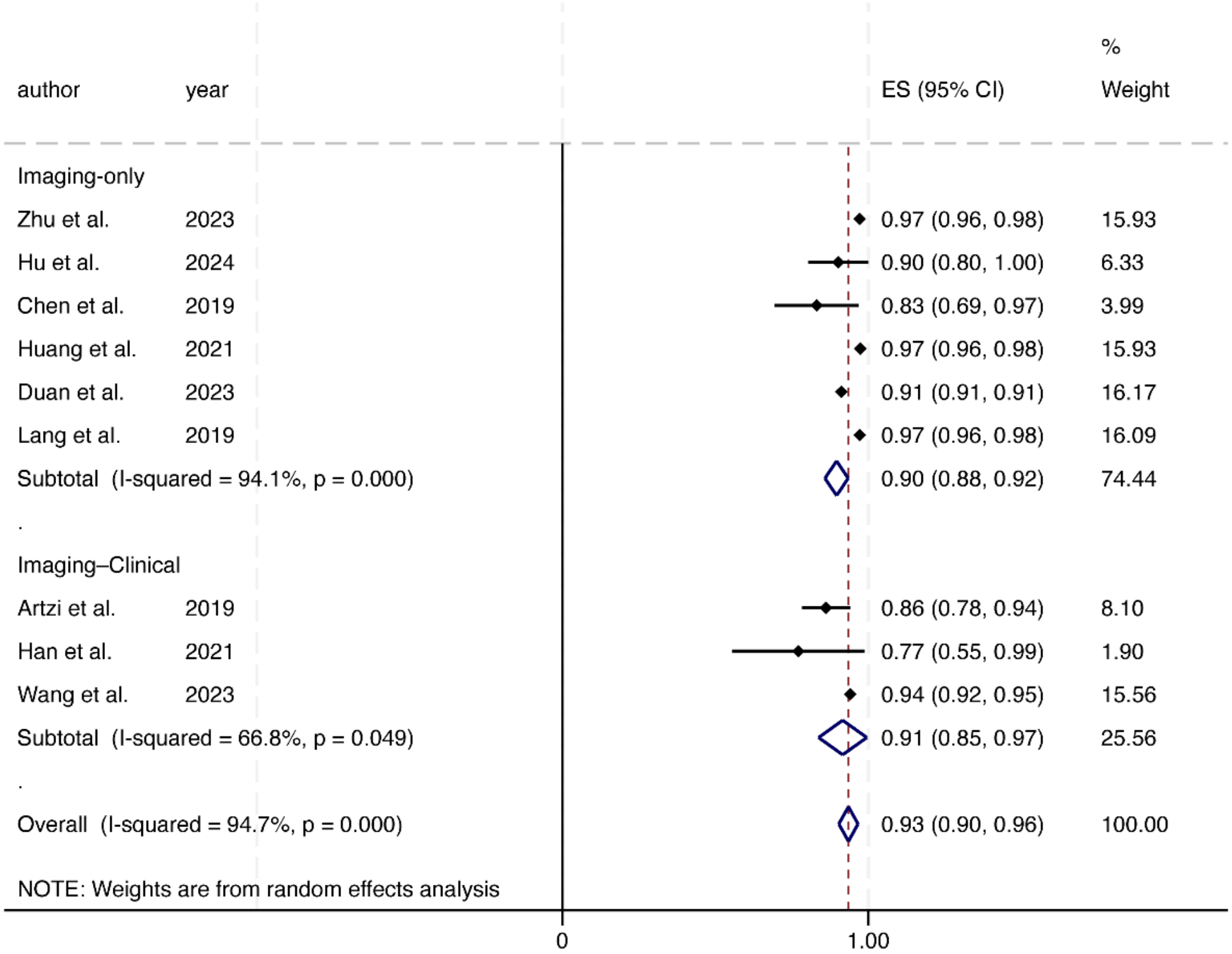

Supplementary Figure 57. Forest plot of pooled Metastasis differentiation performance (AUC) stratified by feature-source subgroup.

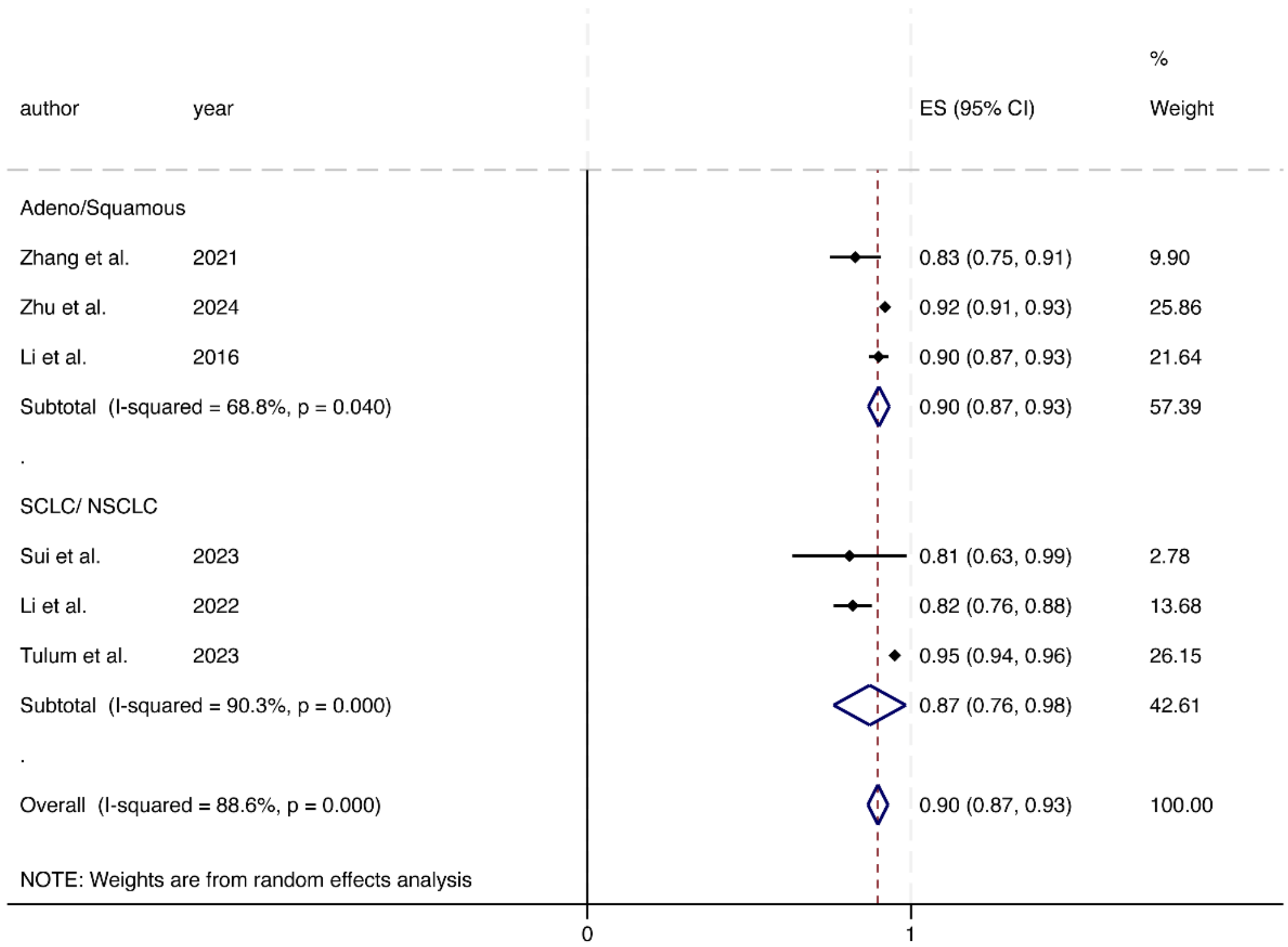

Supplementary Figure 58. Forest plot of pooled Tumor type classification performance (AUC) stratified by histologic subtype classification.

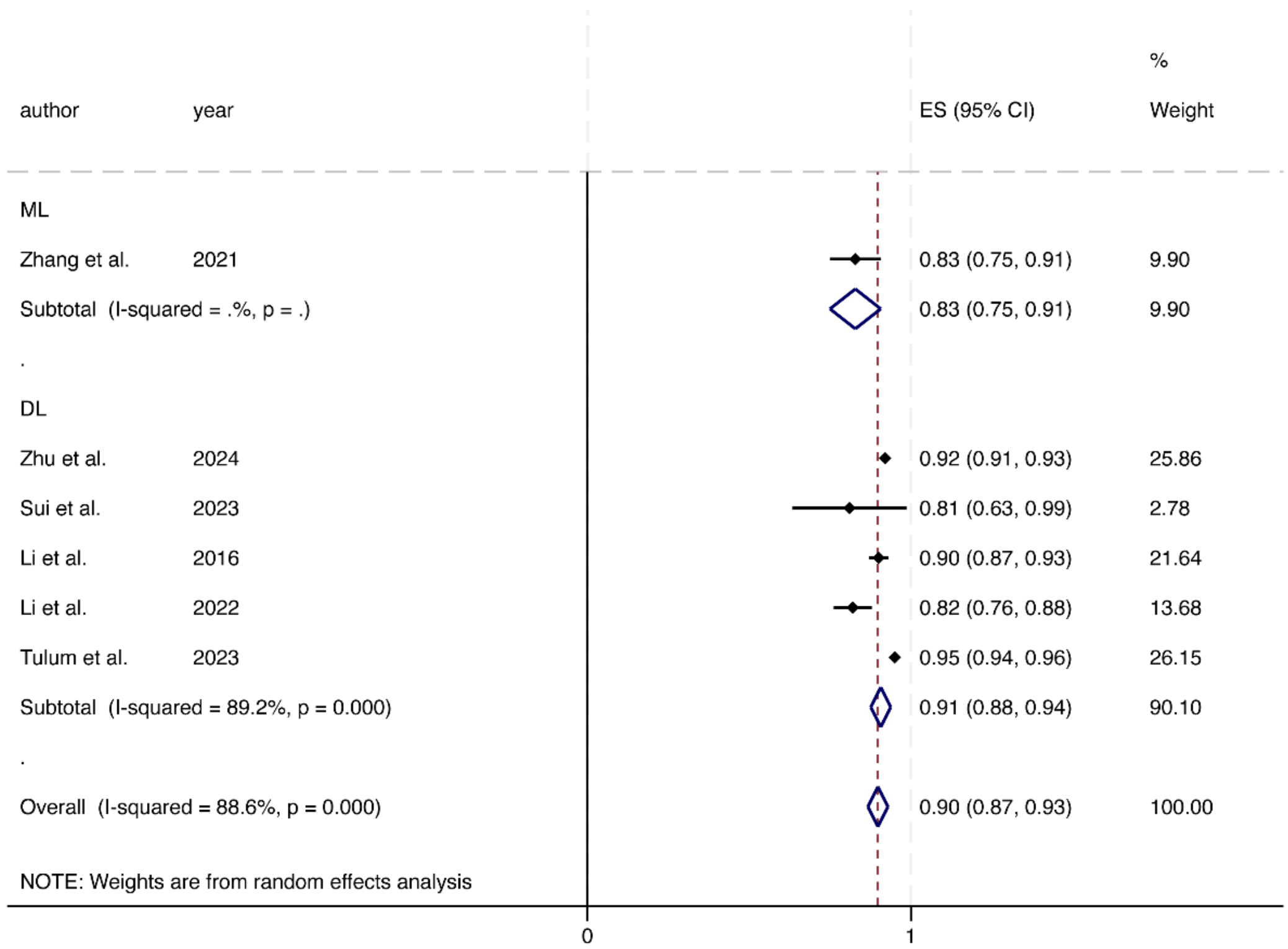

Supplementary Figure 59. Forest plot of pooled Tumor type classification performance (AUC) stratified by algorithm type (ML vs. DL).

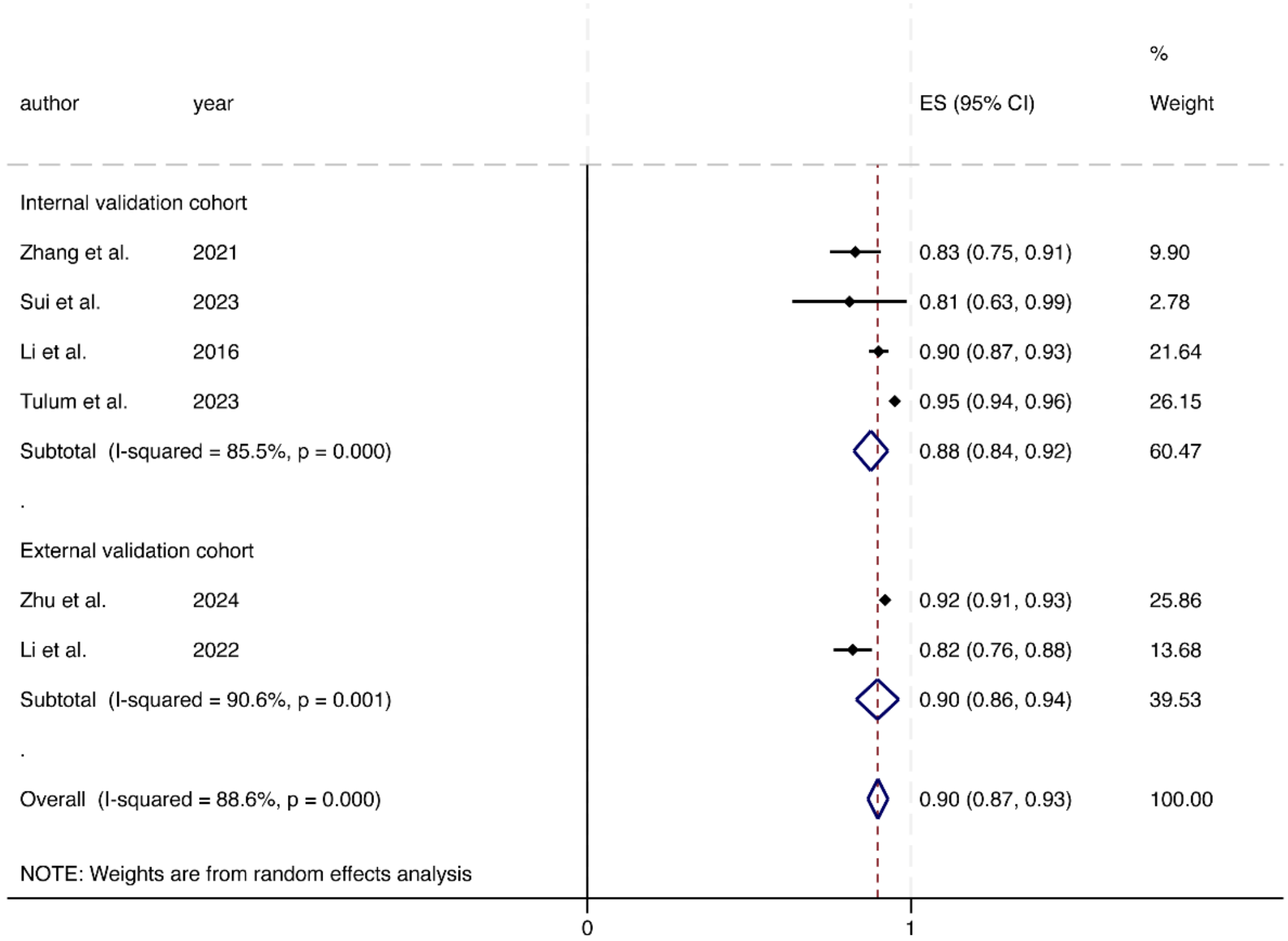

Supplementary Figure 60. Forest plot of pooled Tumor type classification performance (AUC) stratified by cohort type (internal vs. external).

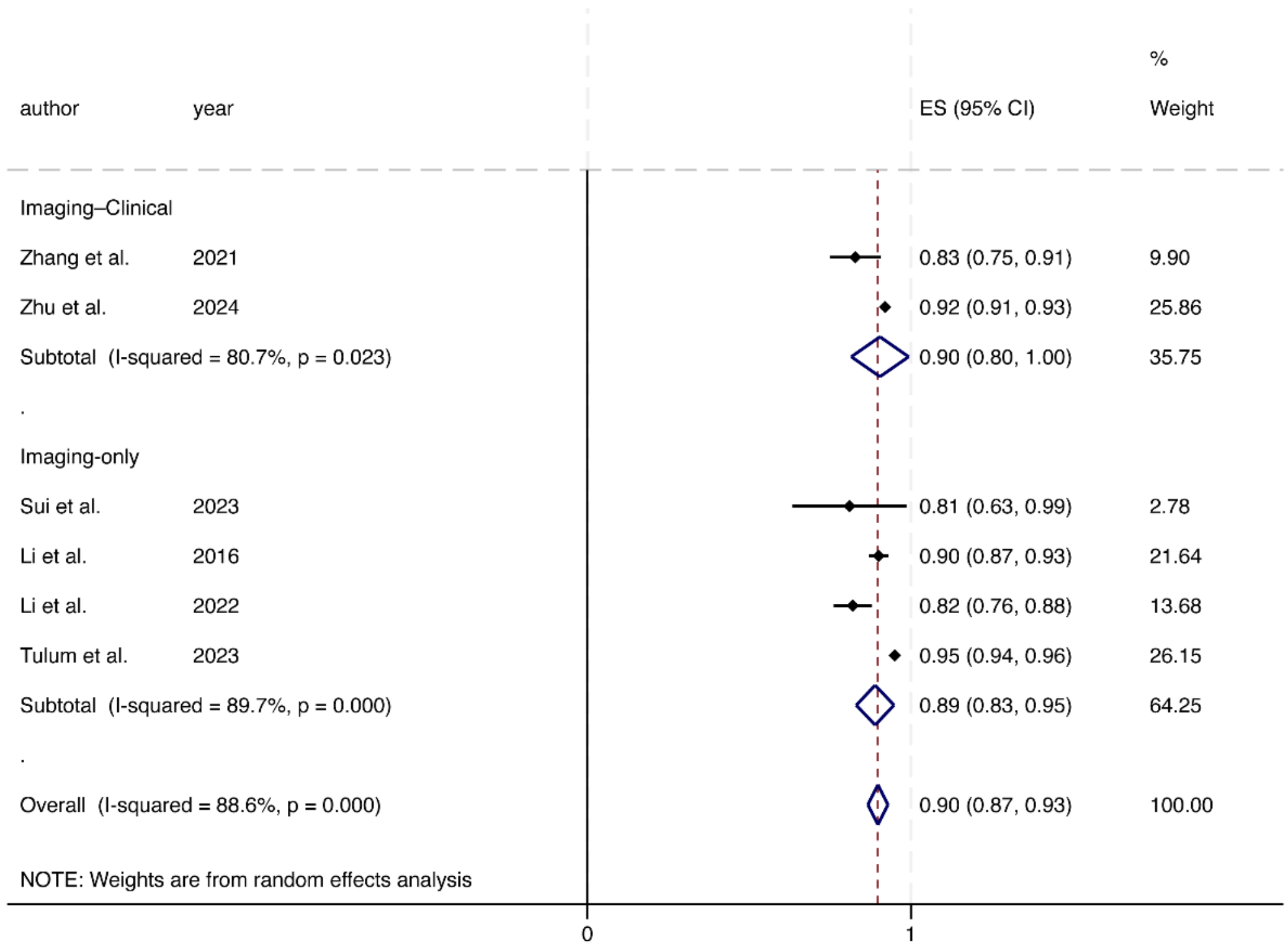

Supplementary Figure 61. Forest plot of pooled Tumor type classification performance (AUC) stratified by feature-source subgroup.

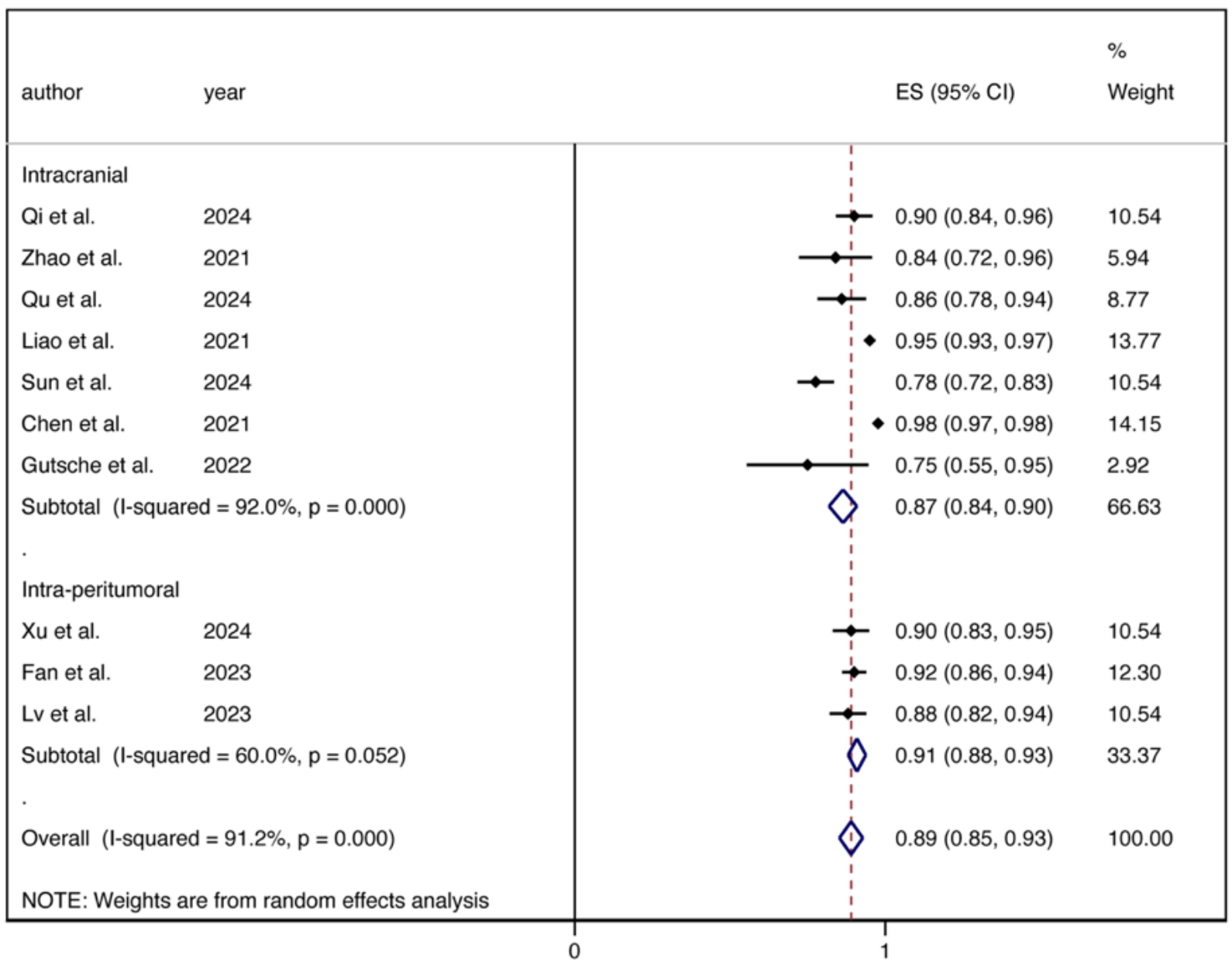

Supplementary Figure 62. Forest plot of pooled Outcome prediction (AUC) stratified by local metastatic ROI subgroup.

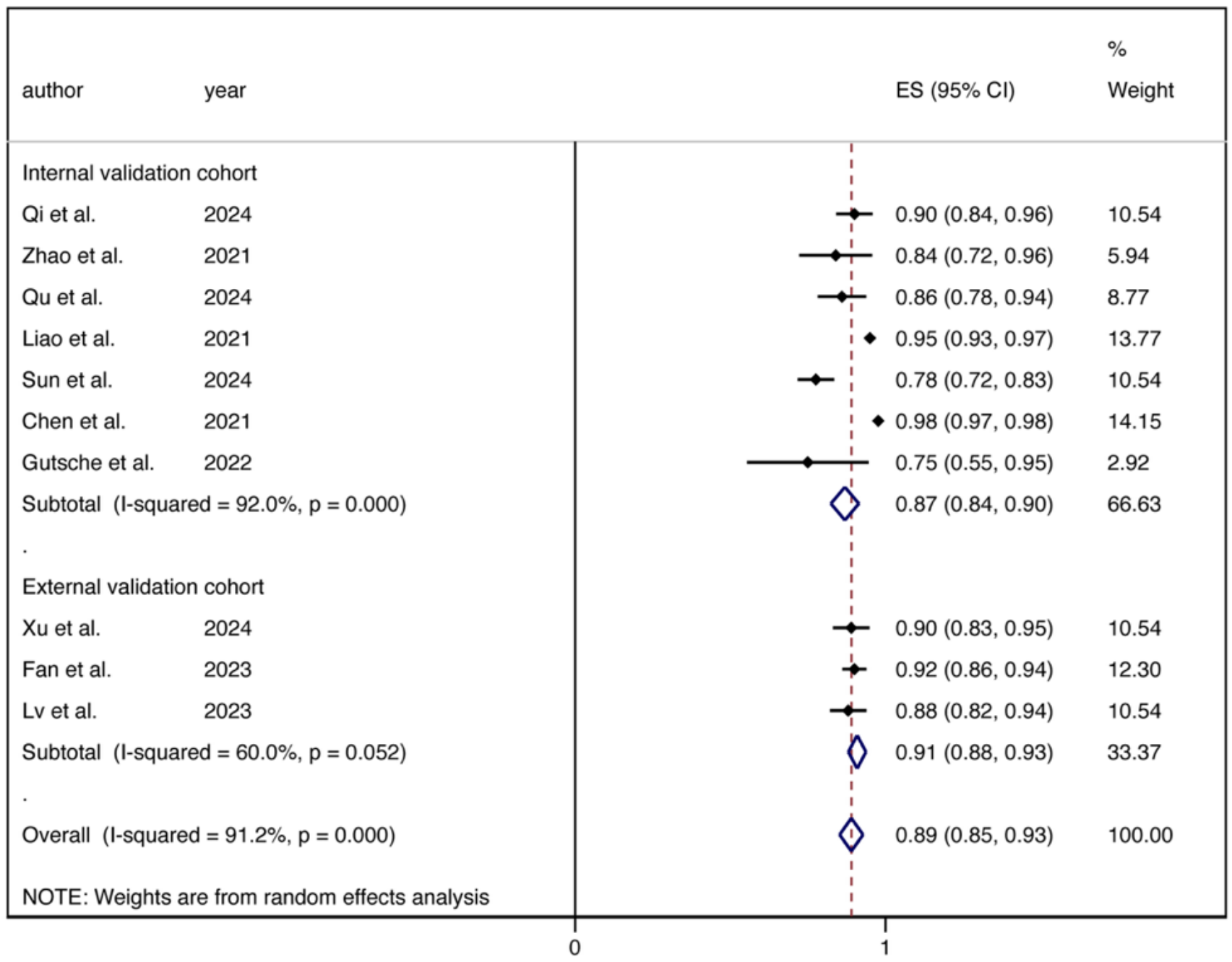

Supplementary Figure 63. Forest plot of pooled Outcome prediction (AUC) stratified by cohort type (internal vs. external).

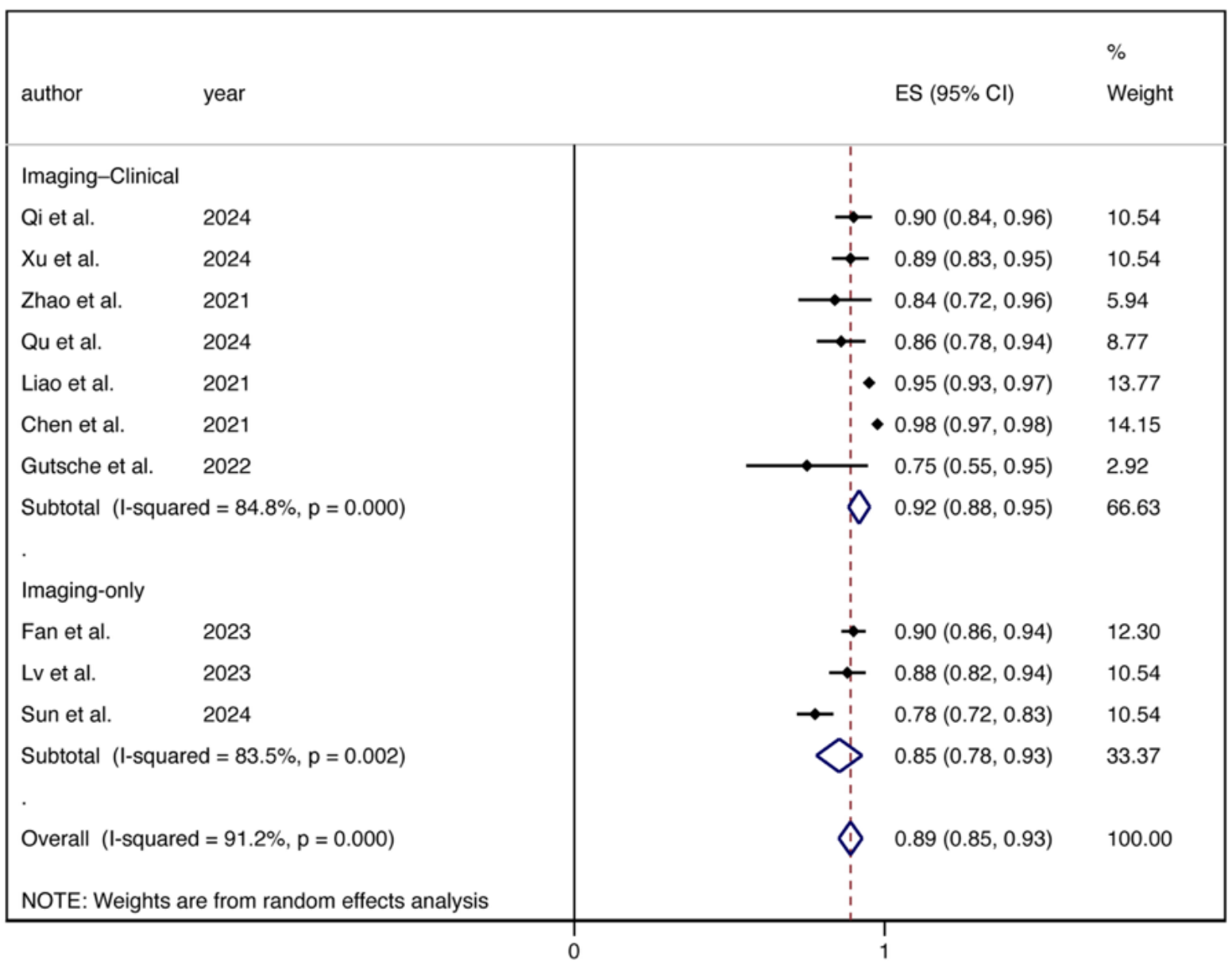

Supplementary Figure 64. Forest plot of pooled Outcome prediction (AUC) stratified by feature-source subgroup.

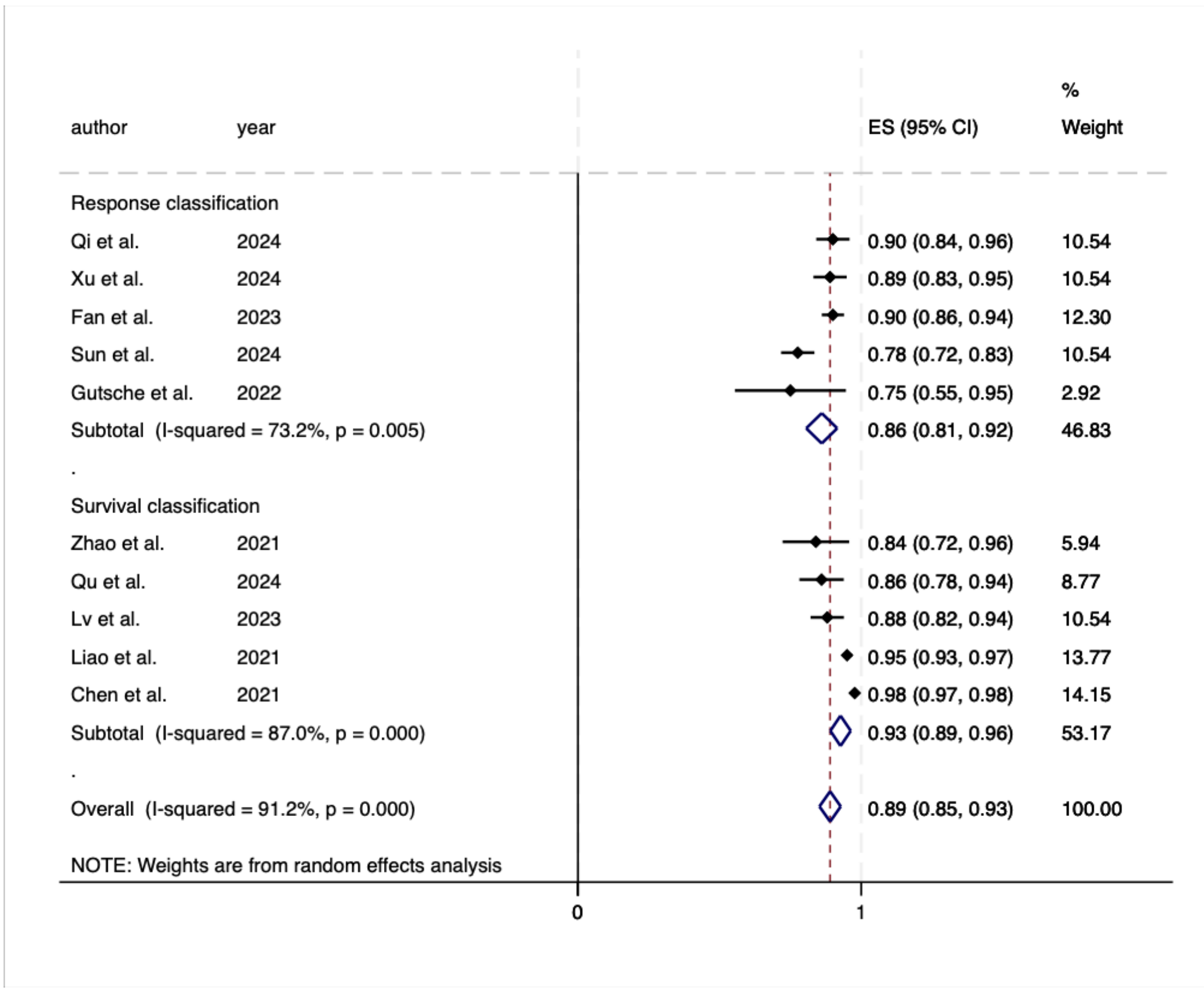

Supplementary Figure 65. Forest plot of pooled Outcome prediction (AUC) stratified by prognostic type classification.

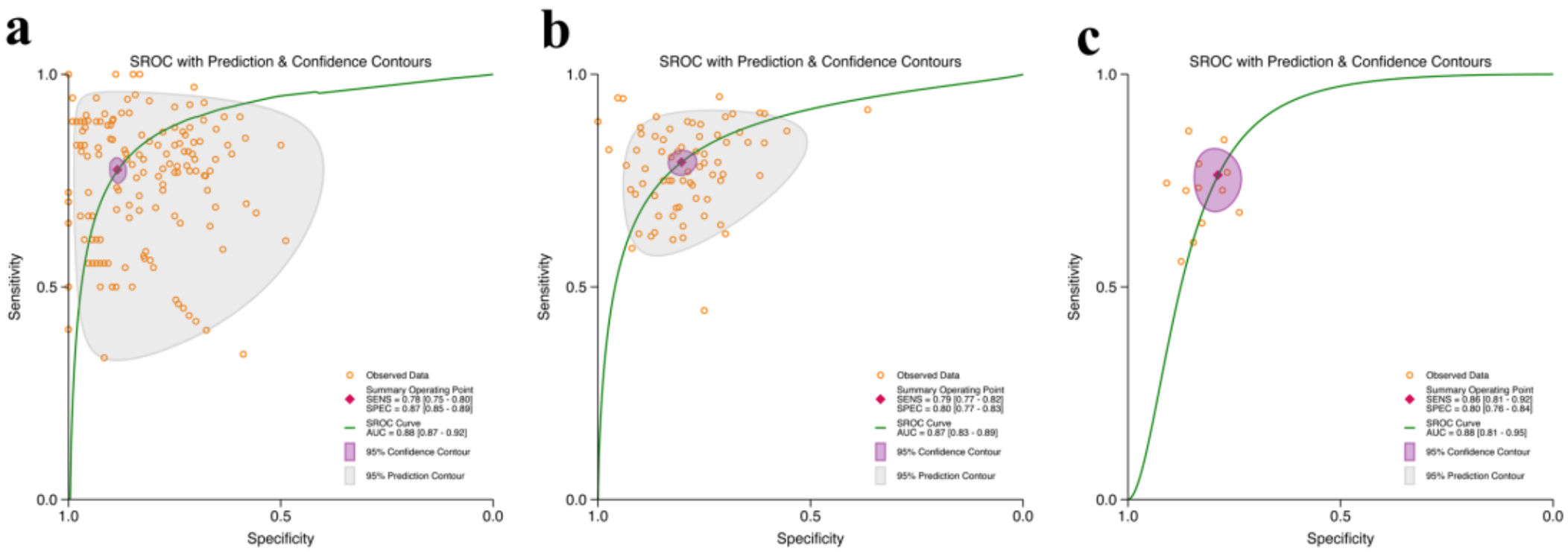

Supplementary Figure 66. SROC curves stratified by metastatic lesion subgroup in Molecular-level prediction studies. Panels show SROC curves for studies of (a) brain metastases (24 studies with 179 tables), (b) bone metastases (10 studies with 59 tables), and (c) liver metastases (2 studies with 12 tables). Abbreviations: SROC=summary receiver operating characteristic; SENS=summary sensitivity; SPEC=summary specificity.

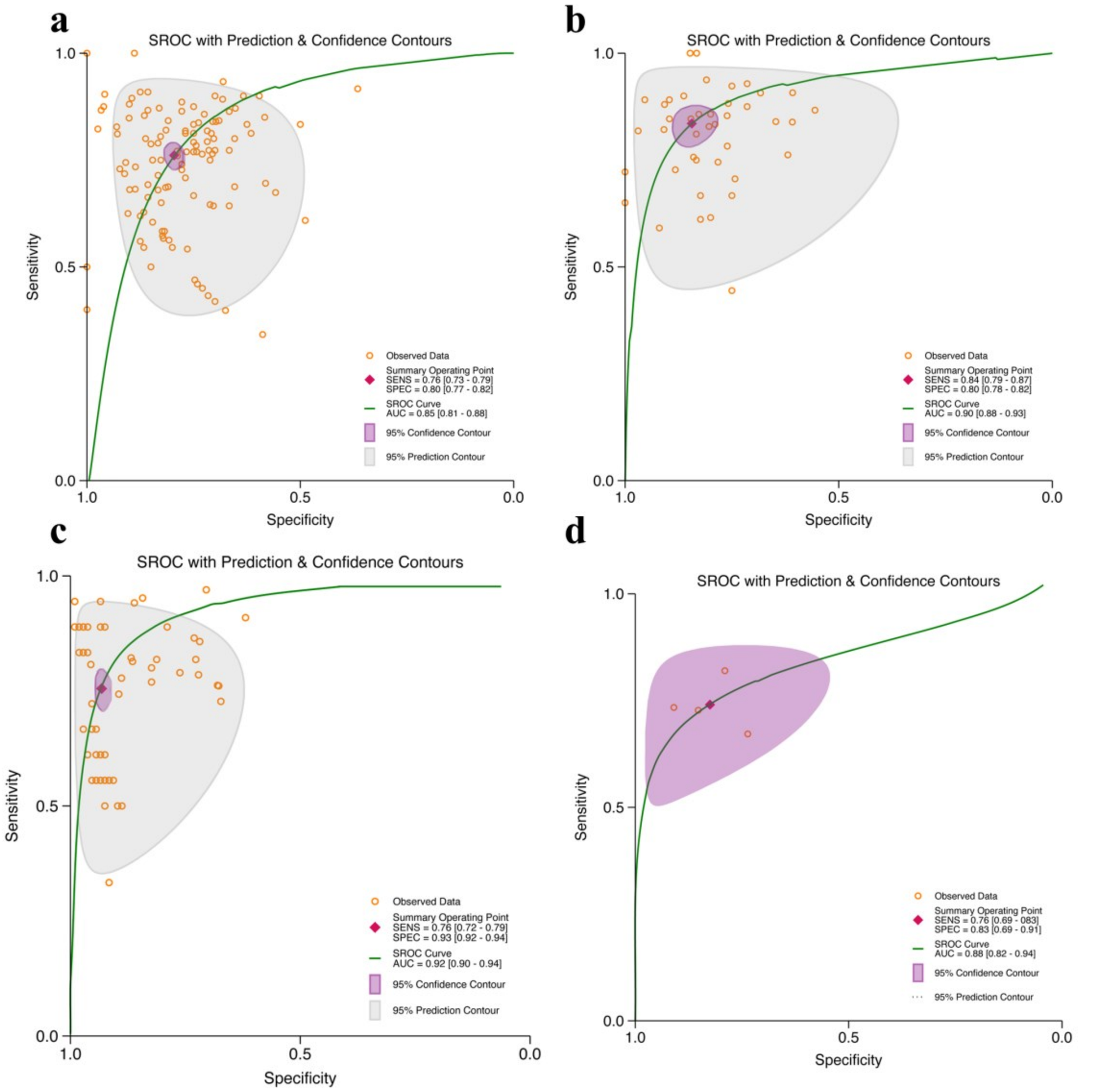

Supplementary Figure 67 SROC curves stratified by molecular subtype classification subgroup in Molecular-level prediction studies. Panels show SROC curves for studies of (a) EGFR mutation (23 studies with 122 tables), (b) EGFR mutation subtypes (6 studies with 41 tables), (c) T790M resistance mutation (5 studies with 81 tables), and (d) HER2 overexpression (1 study with 4 tables). *PD-L1 expression (1 study with 2 tables) did not allow reliable bivariate SROC/HSROC fitting; pooled estimates (univariate random-effects) were SENS 0.92 (0.90–0.94), SPEC 0.68 (0.66–0.70), AUC 0.80 (0.78–0.82). Abbreviations: SROC=summary receiver operating characteristic; SENS=summary sensitivity; SPEC=summary specificity.

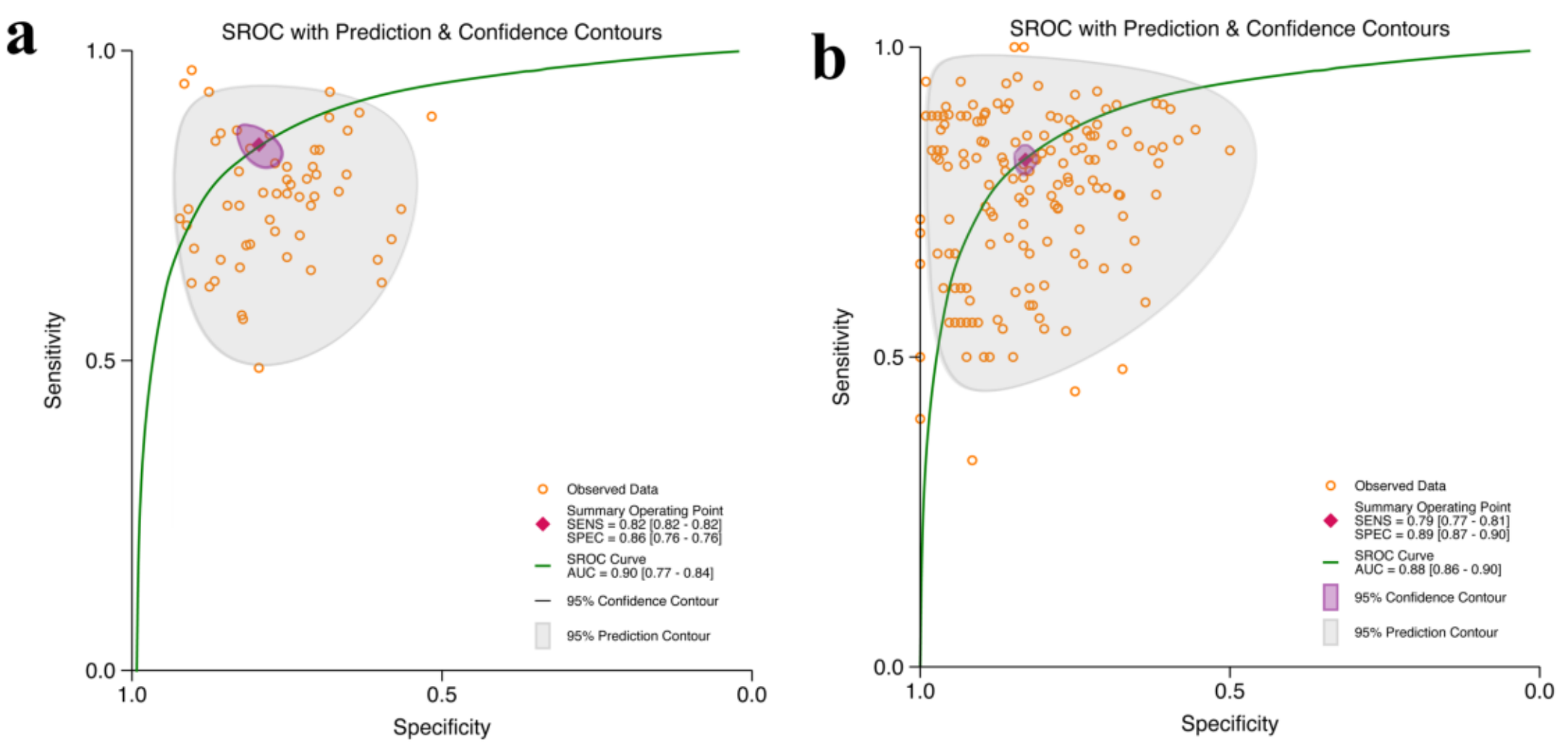

Supplementary Figure 68. SROC curves stratified by lesion-level ROI subgroup in Molecular-level prediction studies. Panels show SROC curves for studies of (a) primary–metastatic (5 studies with 58 tables) and (b) metastatic (31 studies with 192 tables). Abbreviations: SROC=summary receiver operating characteristic; SENS=summary sensitivity; SPEC=summary specificity.

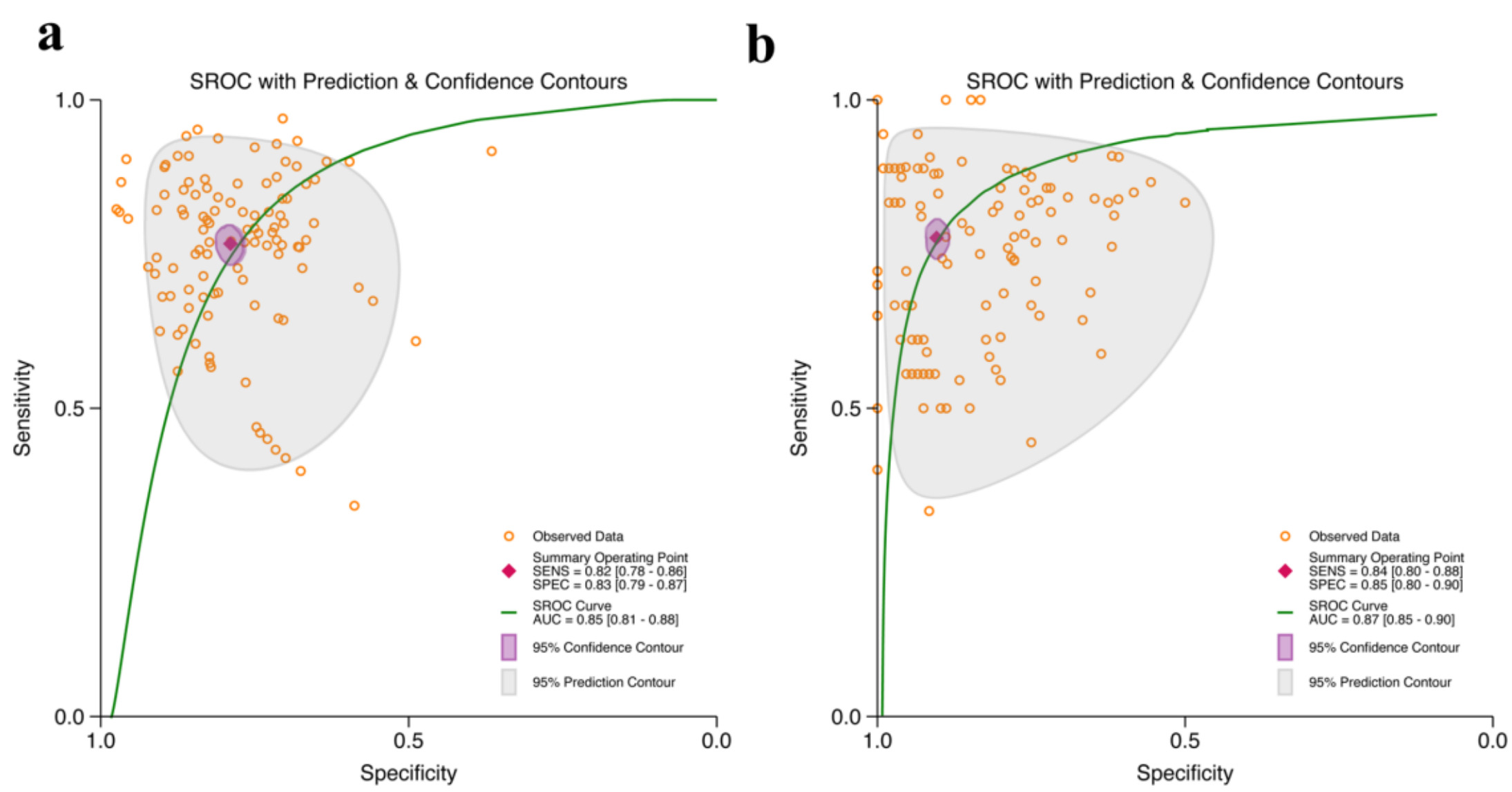

Supplementary Figure 69. SROC curves stratified by local metastatic ROI subgroup in Molecular-level prediction studies. Panels show SROC curves for studies of (a) intratumoral (18 studies with 142 tables) and (b) intra-peritumoral (18 studies with 108 tables). Abbreviations: SROC=summary receiver operating characteristic; SENS=summary sensitivity; SPEC=summary specificity.

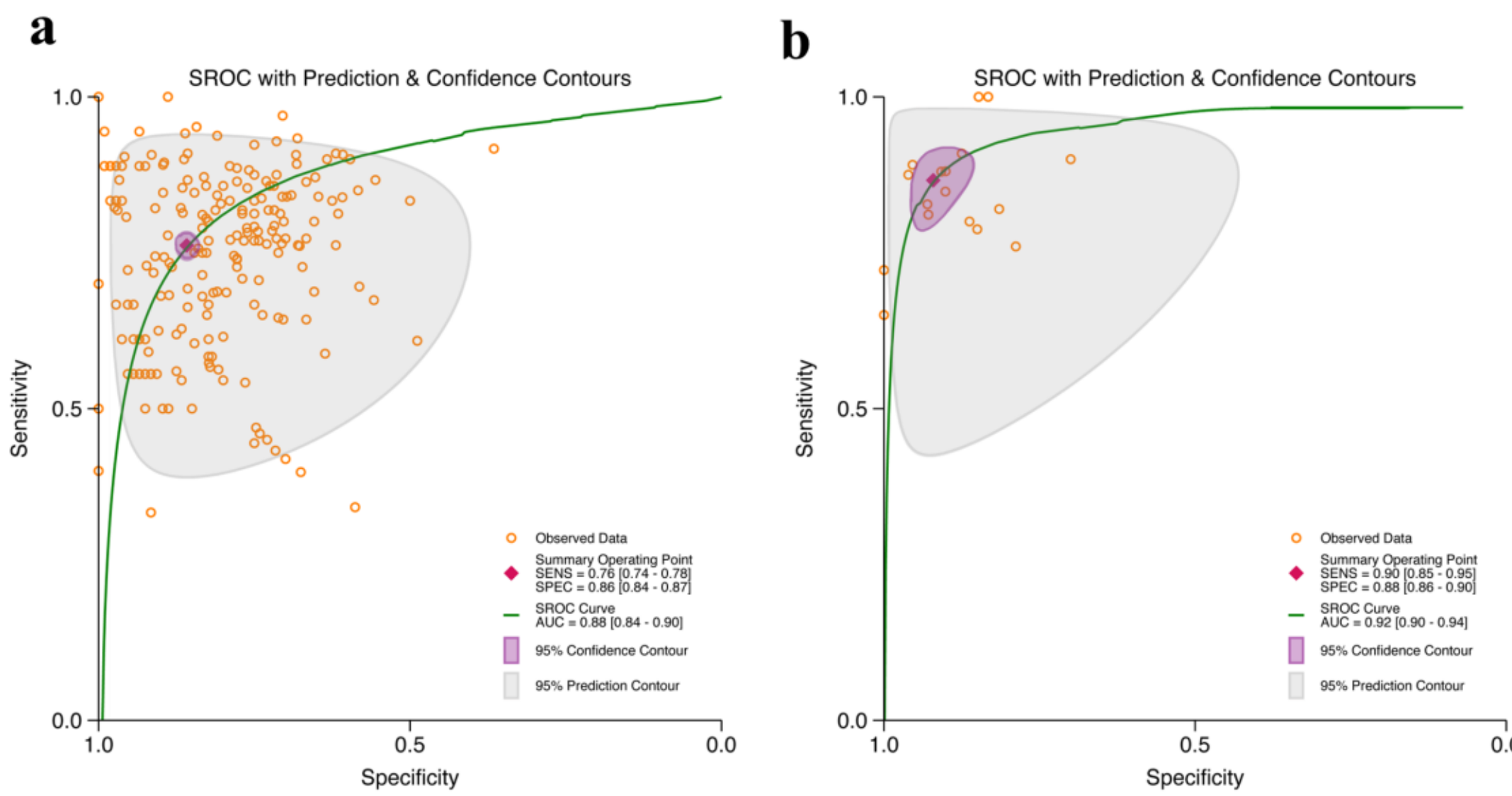

Supplementary Figure 70. SROC curves stratified by algorithm type (ML vs. DL) in Molecular-level prediction studies. Panels show SROC curves for studies using (a) ML algorithms (30 studies with 232 tables) and (b) DL algorithms (6 studies with 18 tables). Abbreviations: SROC=summary receiver operating characteristic; SENS=summary sensitivity; SPEC=summary specificity.

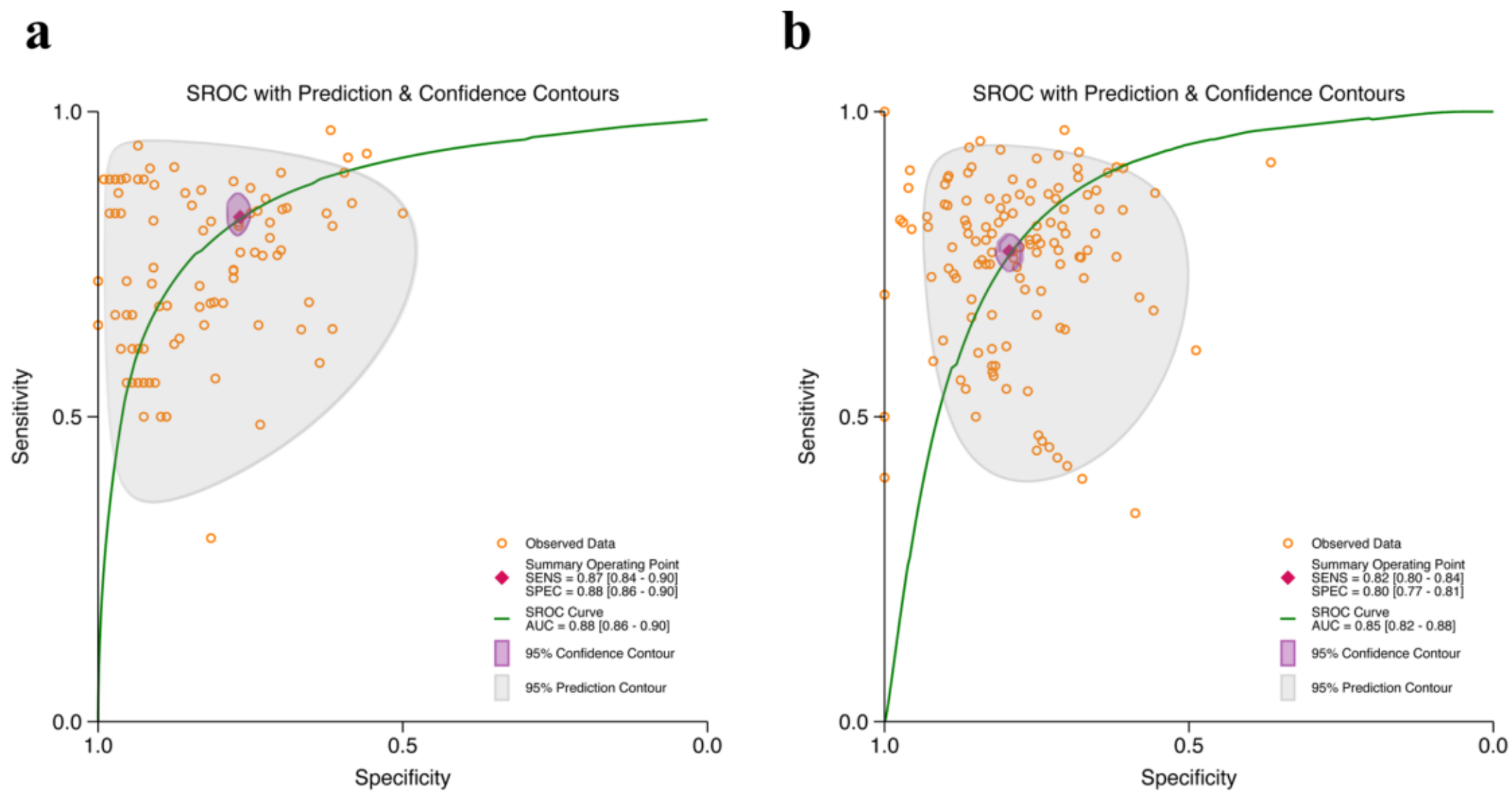

Supplementary Figure 71. SROC curves stratified by cohort type (internal vs. external) in Molecular-level prediction studies. Panels show SROC curves for (a) internal validation (15 studies with 122 tables) and (b) external validation (21 studies with 128 tables). Abbreviations: SROC= summary receiver operating characteristic; SENS= summary sensitivity; SPEC= summary specificity.

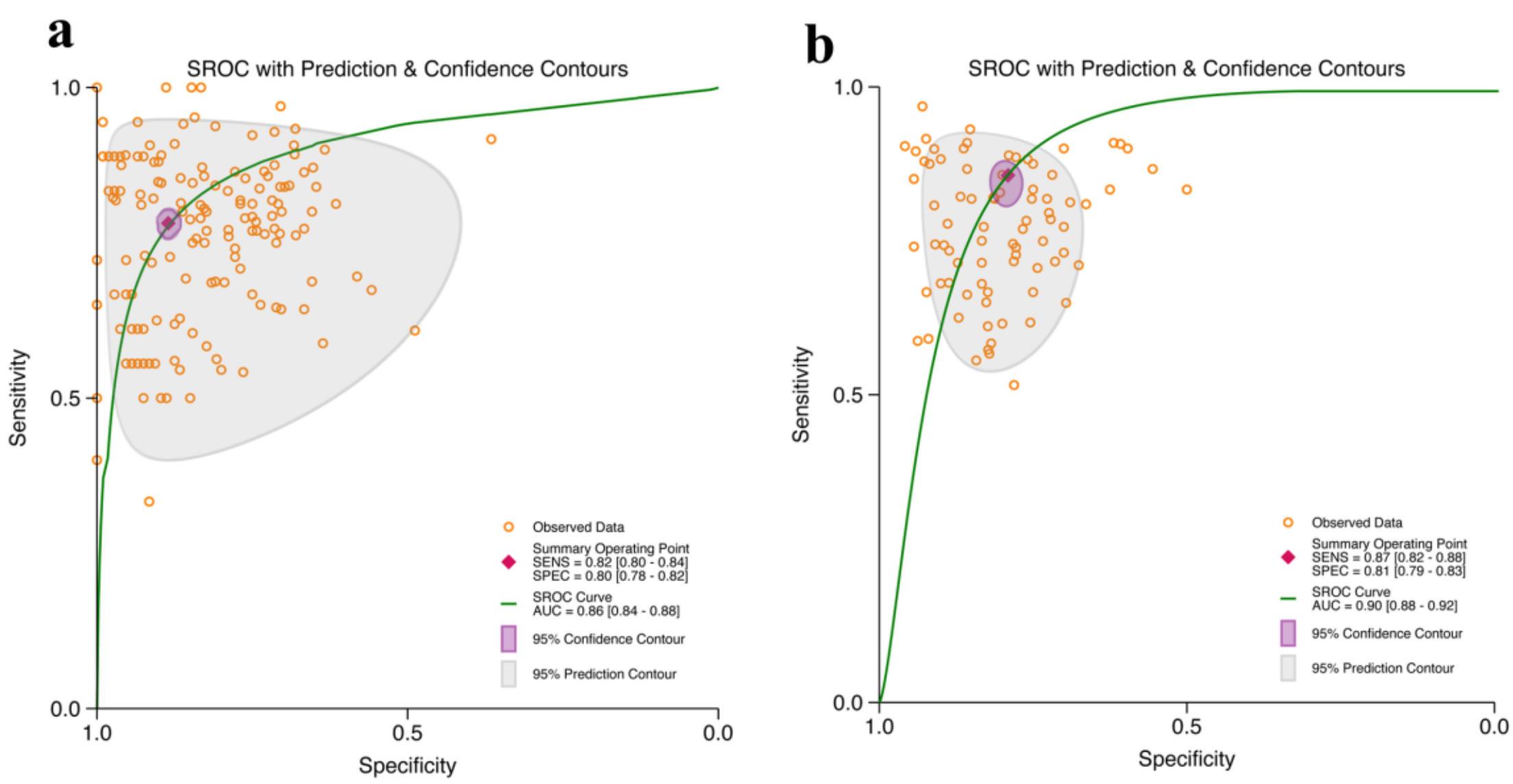

Supplementary Figure 72. SROC curves stratified by feature-source subgroup in Molecular-level prediction studies. Panels show SROC curves for (a) imaging-only studies (22 studies with 180 tables) and (b) imaging–clinical integrated studies (14 studies with 70 tables). Abbreviations: SROC= summary receiver operating characteristic; SENS= summary sensitivity; SPEC= summary specificity.

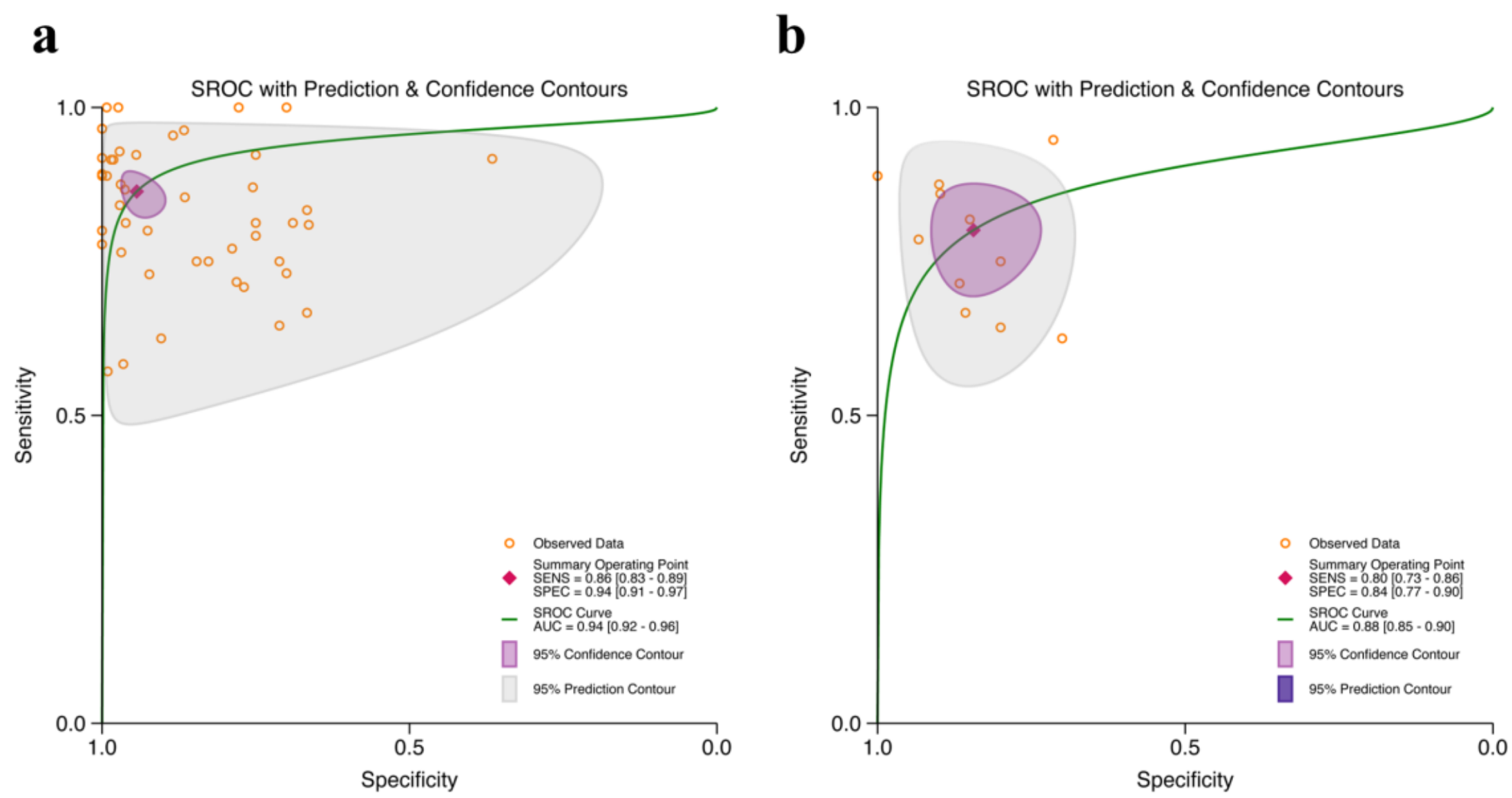

Supplementary Figure 73. SROC curves stratified by metastatic lesion subgroup in Metastasis differentiation studies. Panels show SROC curves for studies of (a) brain metastases (6 studies with 52 tables) and (b) bone metastases (3 studies with 11 tables). Abbreviations: SROC=summary receiver operating characteristic; SENS=summary sensitivity; SPEC=summary specificity.

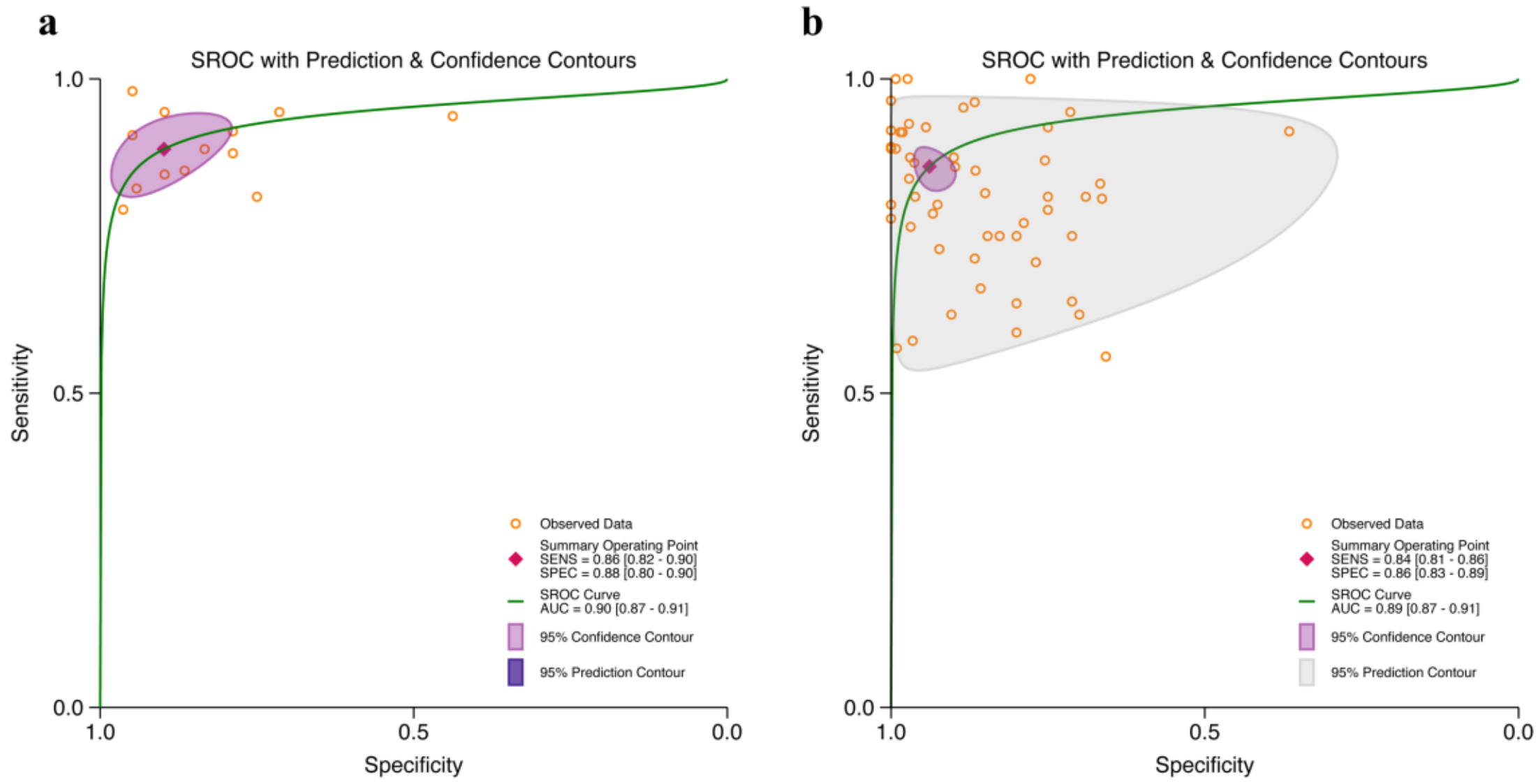

Supplementary Figure 74. SROC curves stratified by lesion-level ROI subgroup in Metastasis differentiation studies. Panels show SROC curves for studies of (a) primary–metastatic (2 studies with 13 tables) and (b) metastatic (7 studies with 50 tables). Abbreviations: SROC=summary receiver operating characteristic; SENS=summary sensitivity; SPEC=summary specificity.

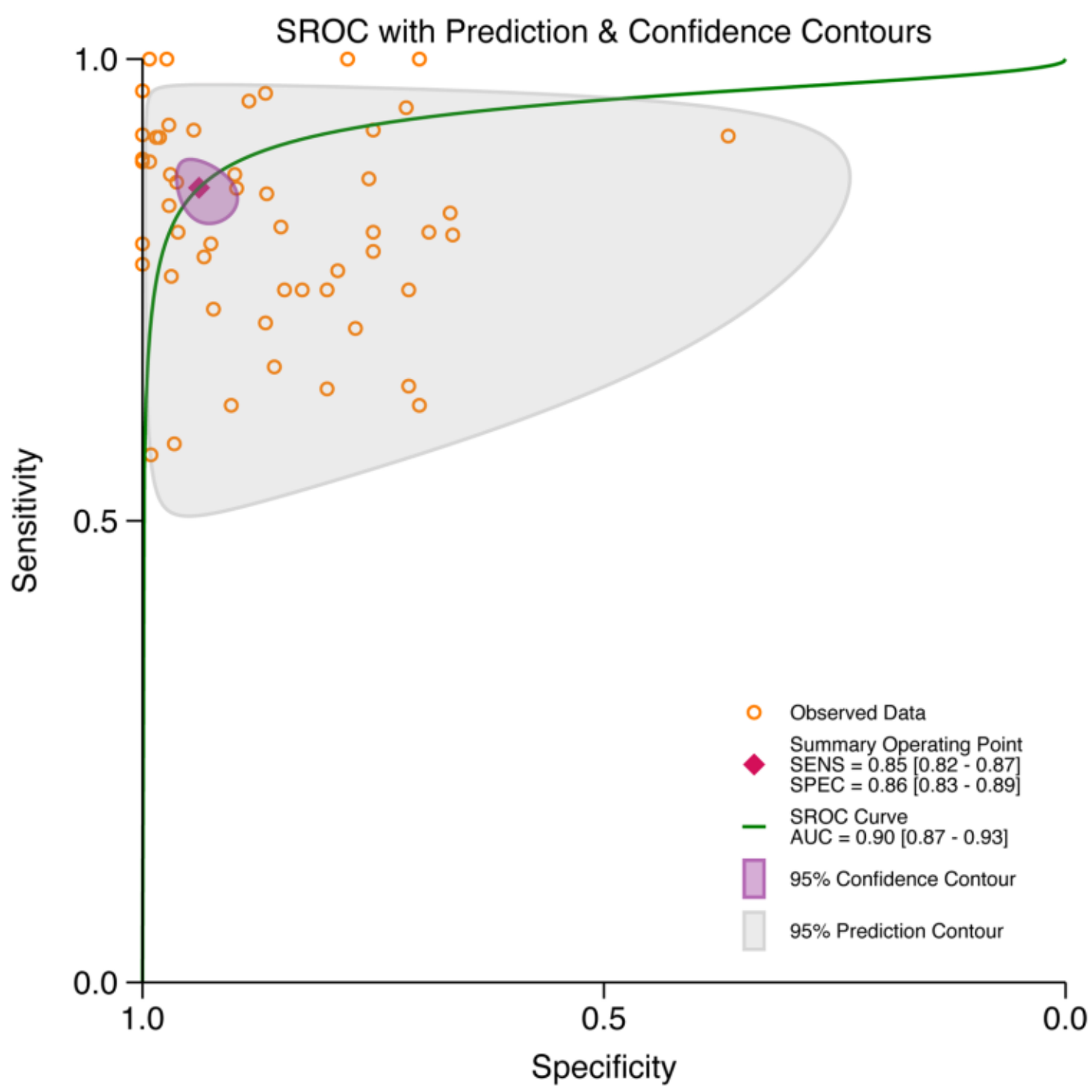

Supplementary Figure 75. SROC curves stratified by local metastatic ROI subgroup in Metastasis differentiation studies. The SROC curve is shown for the intratumoral ROI subgroup (8 studies with 60 tables). The intra-peritumoral subgroup (1 study with 3 tables) was insufficient to support reliable bivariate SROC/HSROC fitting; pooled estimates: SENS 0.90 (0.88–0.92), SPEC 0.86 (0.83–0.89), AUC 0.90 (0.87–0.93). Abbreviations:

SROC=summary receiver operating characteristic; SENS=summary sensitivity; SPEC=summary specificity.

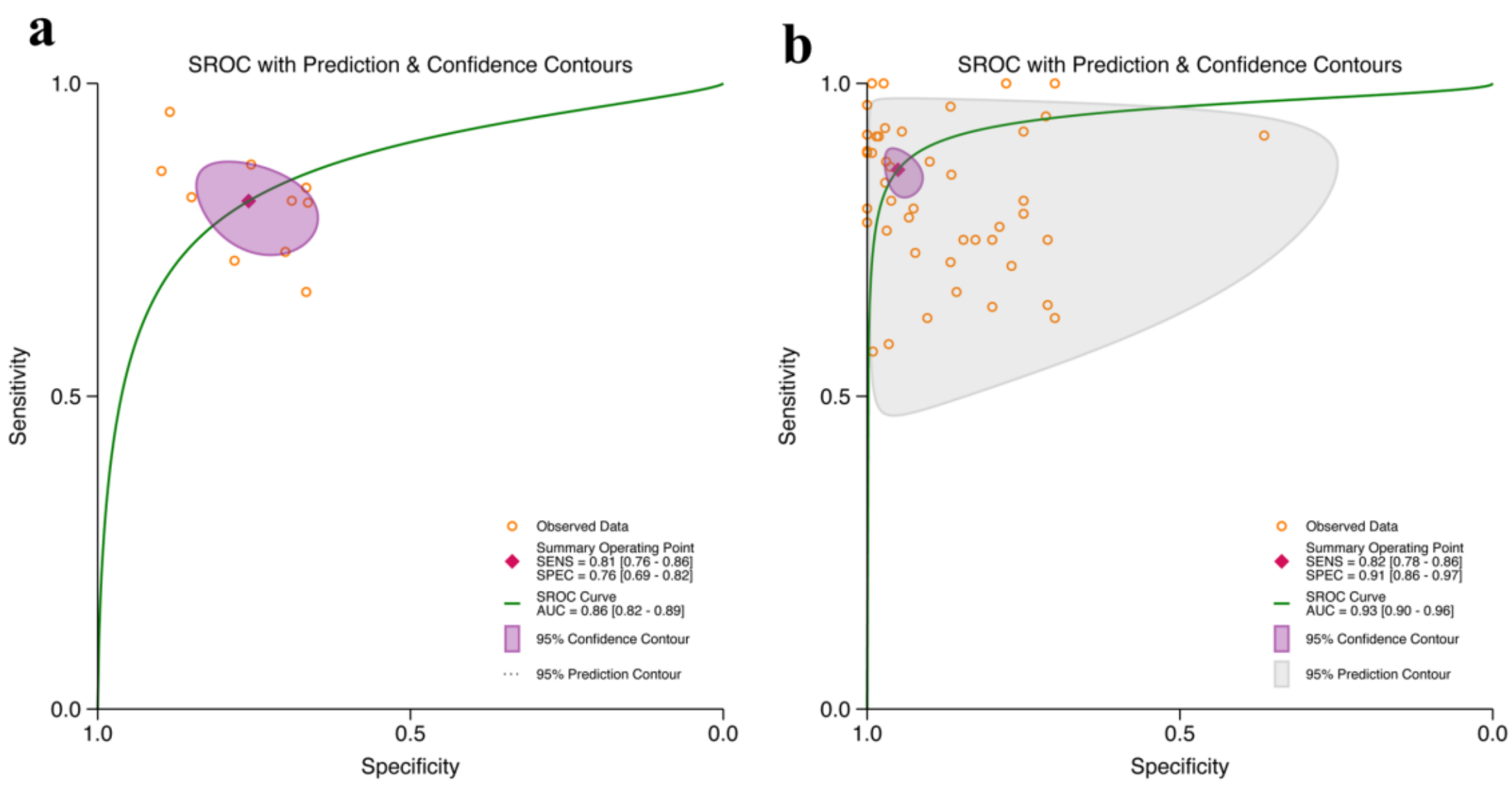

Supplementary Figure 76. SROC curves stratified by algorithm type (ML vs. DL) in Metastasis differentiation studies. Panels show SROC curves for studies using (a) ML algorithms (5 studies with 10 tables) and (b) DL algorithms (4 studies with 53 tables). Abbreviations: ML= machine learning; DL= deep learning; SROC=summary receiver operating characteristic; SENS=summary sensitivity; SPEC=summary specificity.

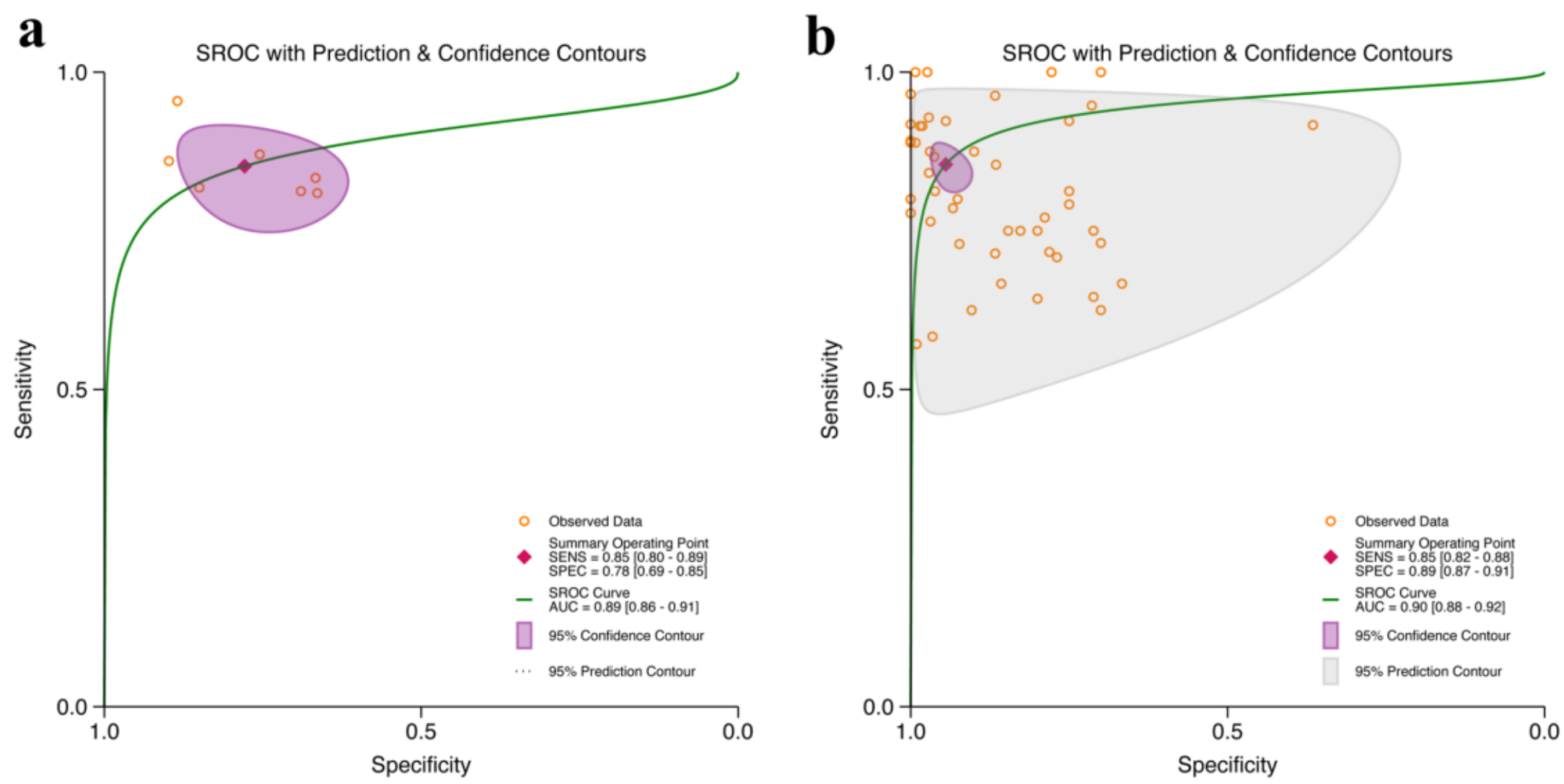

Supplementary Figure 77. SROC curves stratified by cohort type (internal vs. external) in Metastasis differentiation studies. Panels show SROC curves for (a) internal validation (4 studies with 10 tables) and (b) external validation (5 studies with 53 tables). Abbreviations: SROC=summary receiver operating characteristic; SENS=summary sensitivity; SPEC=summary specificity.

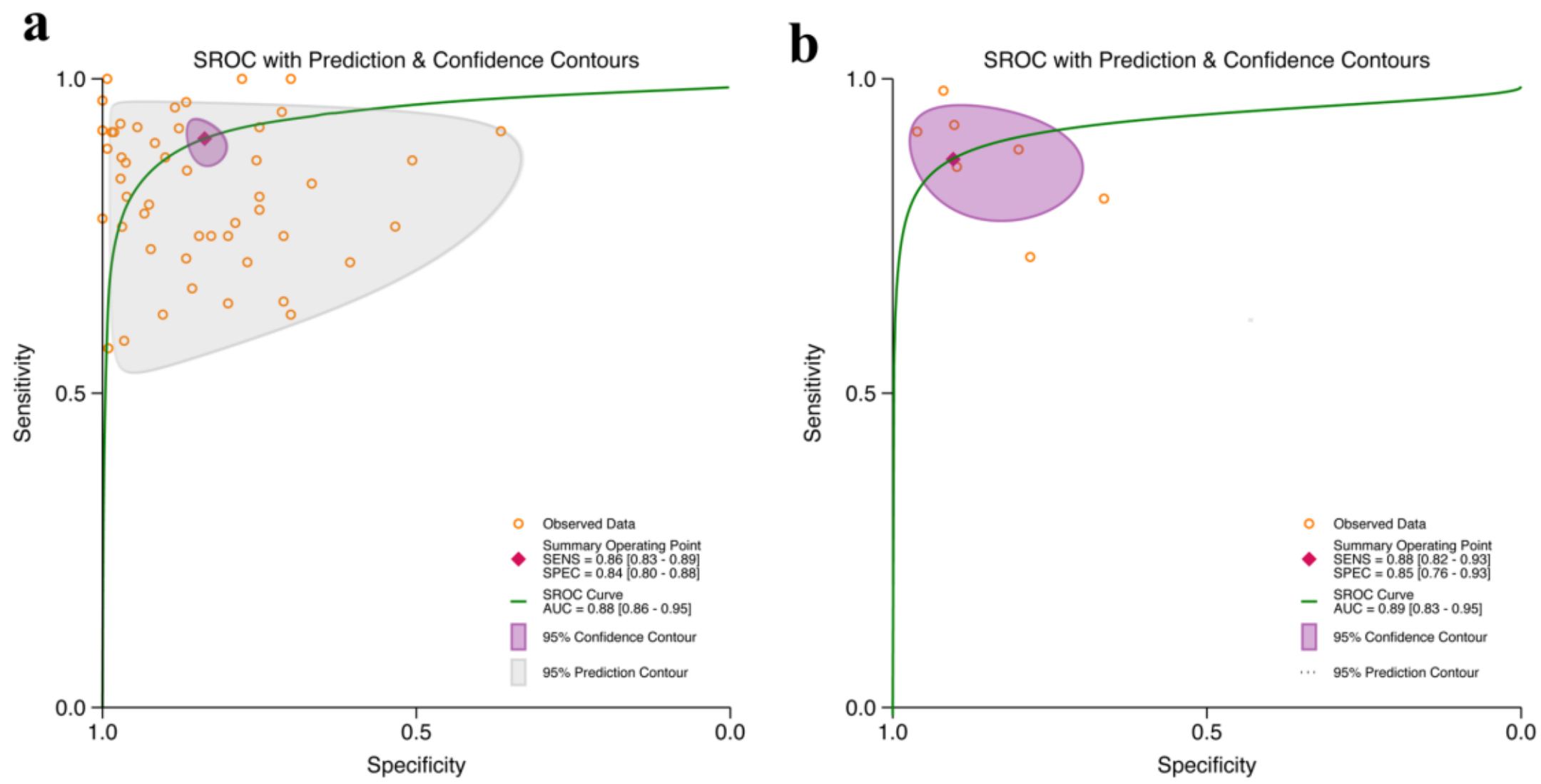

Supplementary Figure 78. SROC curves stratified by feature-source subgroup in Metastasis differentiation studies. Panels show SROC curves for (a) imaging-only studies (6 studies with 56 tables) and (b) imaging–clinical integrated studies (3 studies with 7 tables). Abbreviations: SROC=summary receiver operating characteristic; SENS=summary sensitivity; SPEC=summary specificity.

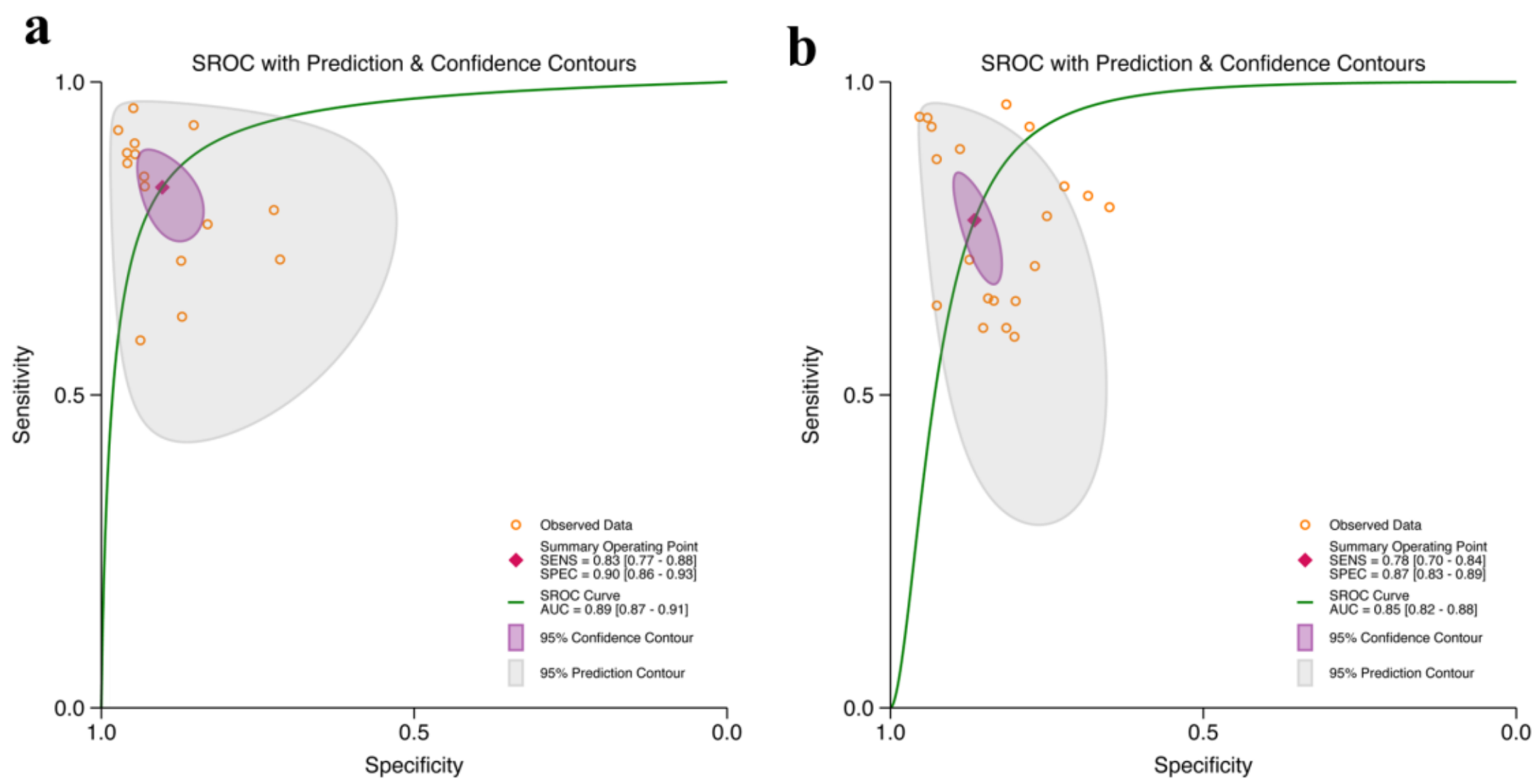

Supplementary Figure 79. SROC curves stratified by histologic subtype classification subgroup in Tumor type classification studies. Panels show SROC curves for (a) adeno/squamous studies (3 studies with 16 tables) and (b) SCLC/NSCLC studies (3 studies with 21 tables). Abbreviations: SROC=summary receiver operating characteristic; SENS=summary sensitivity; SPEC=summary specificity.

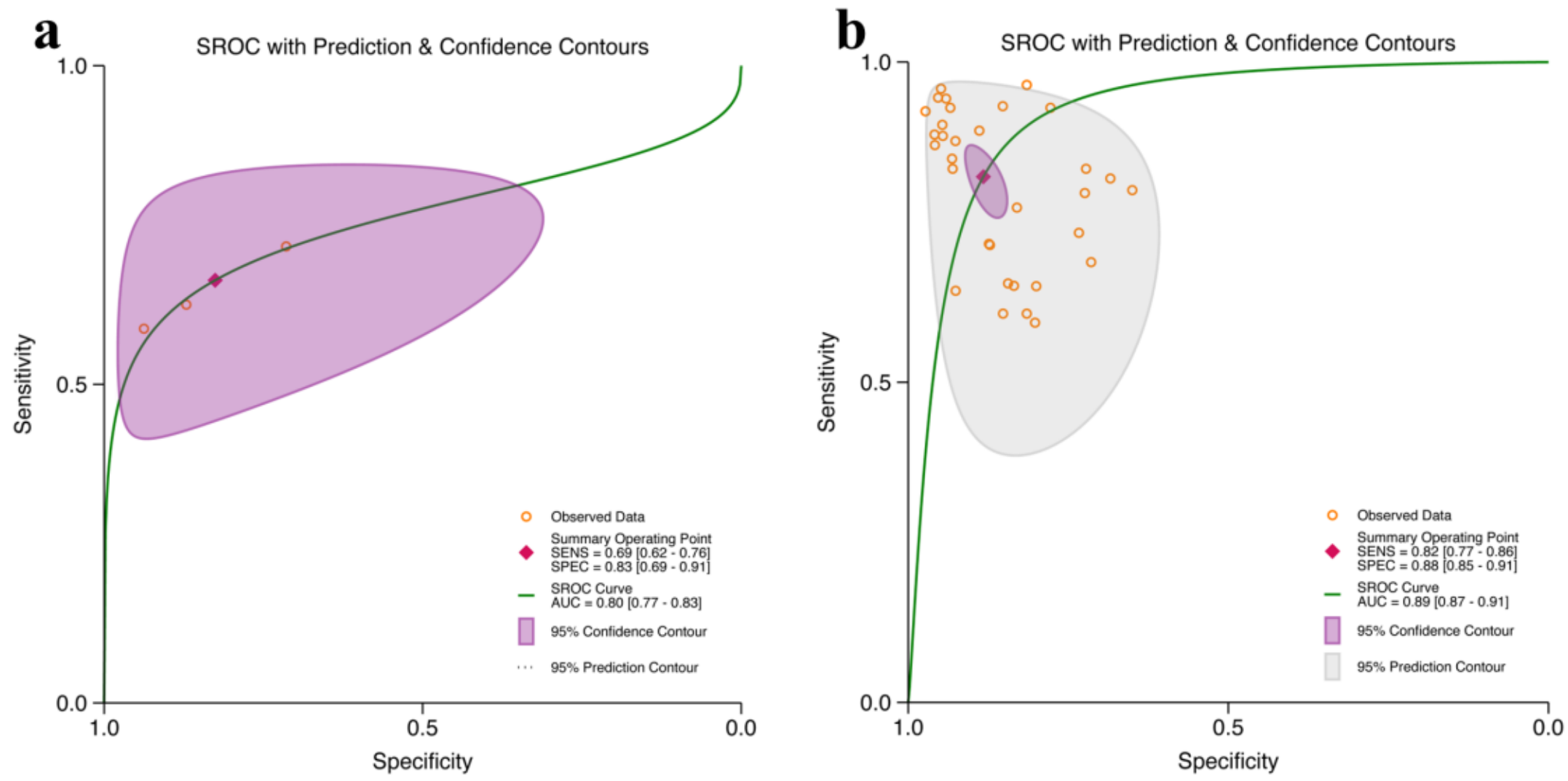

Supplementary Figure 80. SROC curves stratified by algorithm type (ML vs. DL) in Tumor type classification studies. Panels show SROC curves for studies using (a) ML algorithms (1 study with 4 tables) and (b) DL algorithms (5 studies with 33 tables). Abbreviations: ML=machine learning; DL= deep learning; SROC=summary receiver operating characteristic; SENS=summary sensitivity; SPEC=summary specificity.

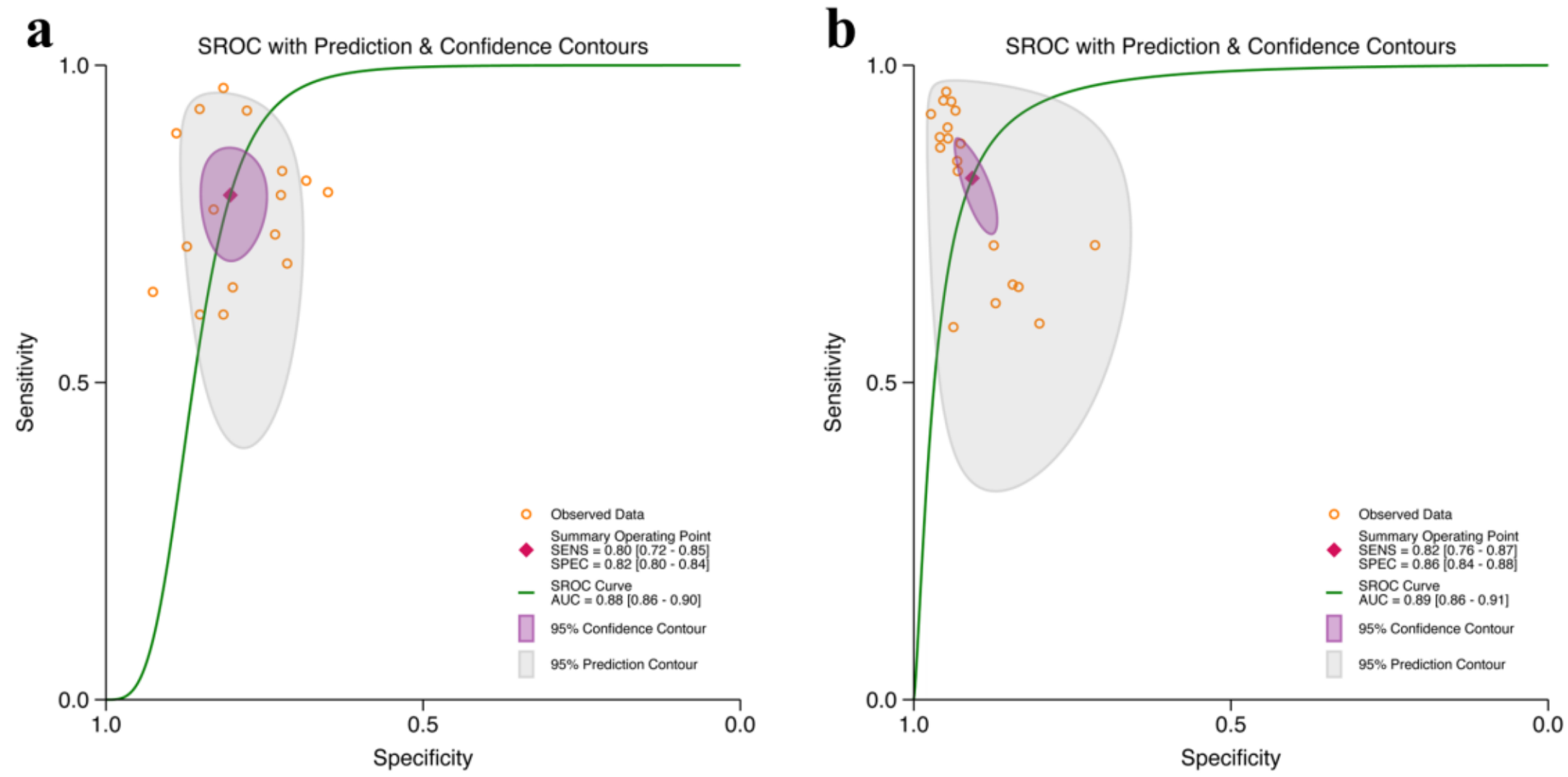

Supplementary Figure 81. SROC curves stratified by cohort type (internal vs. external) in Tumor type classification studies. Panels show SROC curves for (a) internal validation (4 studies with 21 tables) and (b) external validation (2 studies with 16 tables). Abbreviations: SROC= summary receiver operating characteristic; SENS= summary sensitivity; SPEC= summary specificity.

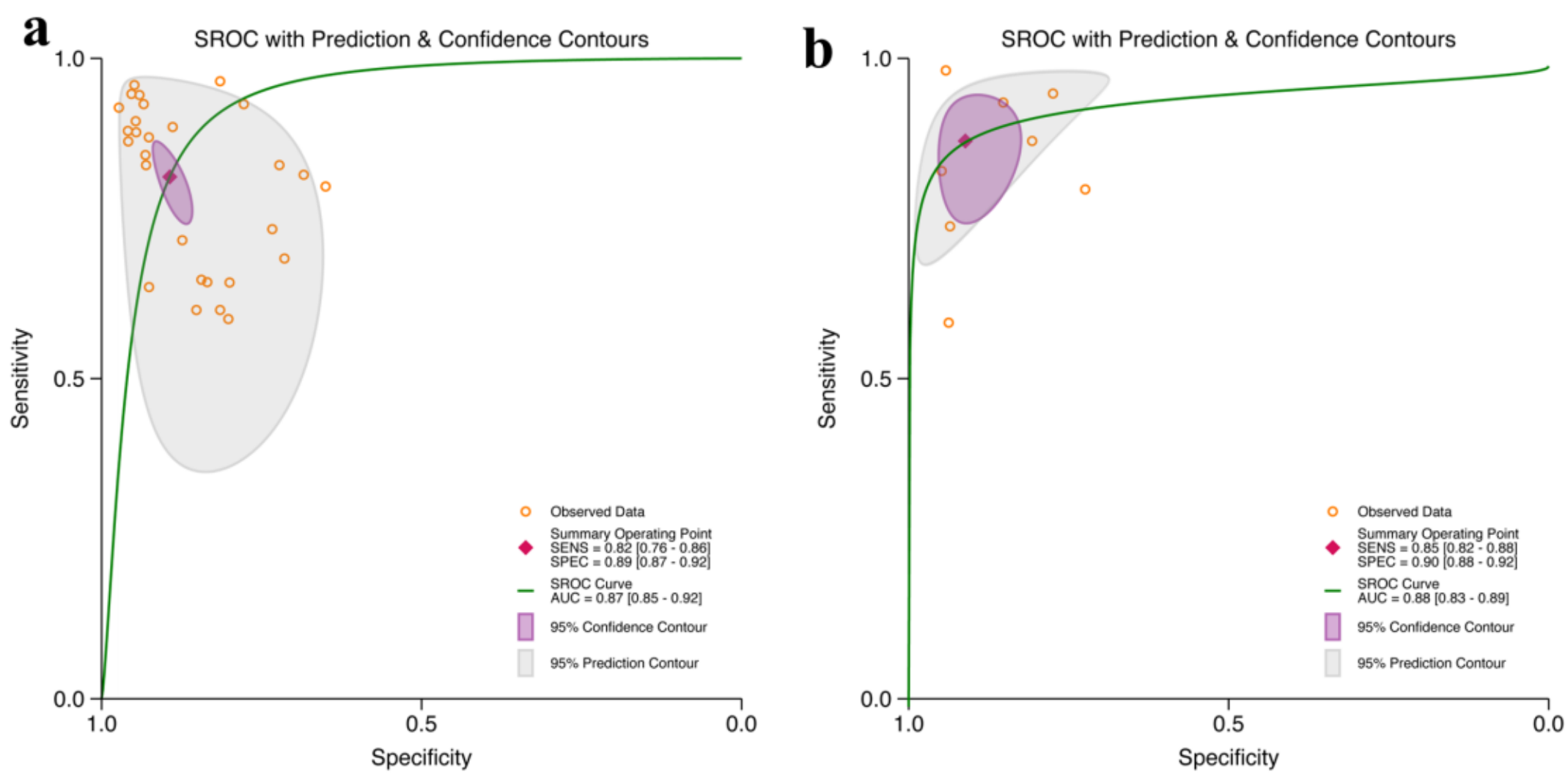

Supplementary Figure 82. SROC curves stratified by feature-source subgroup in Tumor type classification studies. Panels show SROC curves for (a) imaging-only studies (4 studies with 29 tables) and (b) imaging–clinical integrated studies (2 studies with 8 tables). Abbreviations: SROC=summary receiver operating characteristic; SENS=summary sensitivity; SPEC=summary specificity.

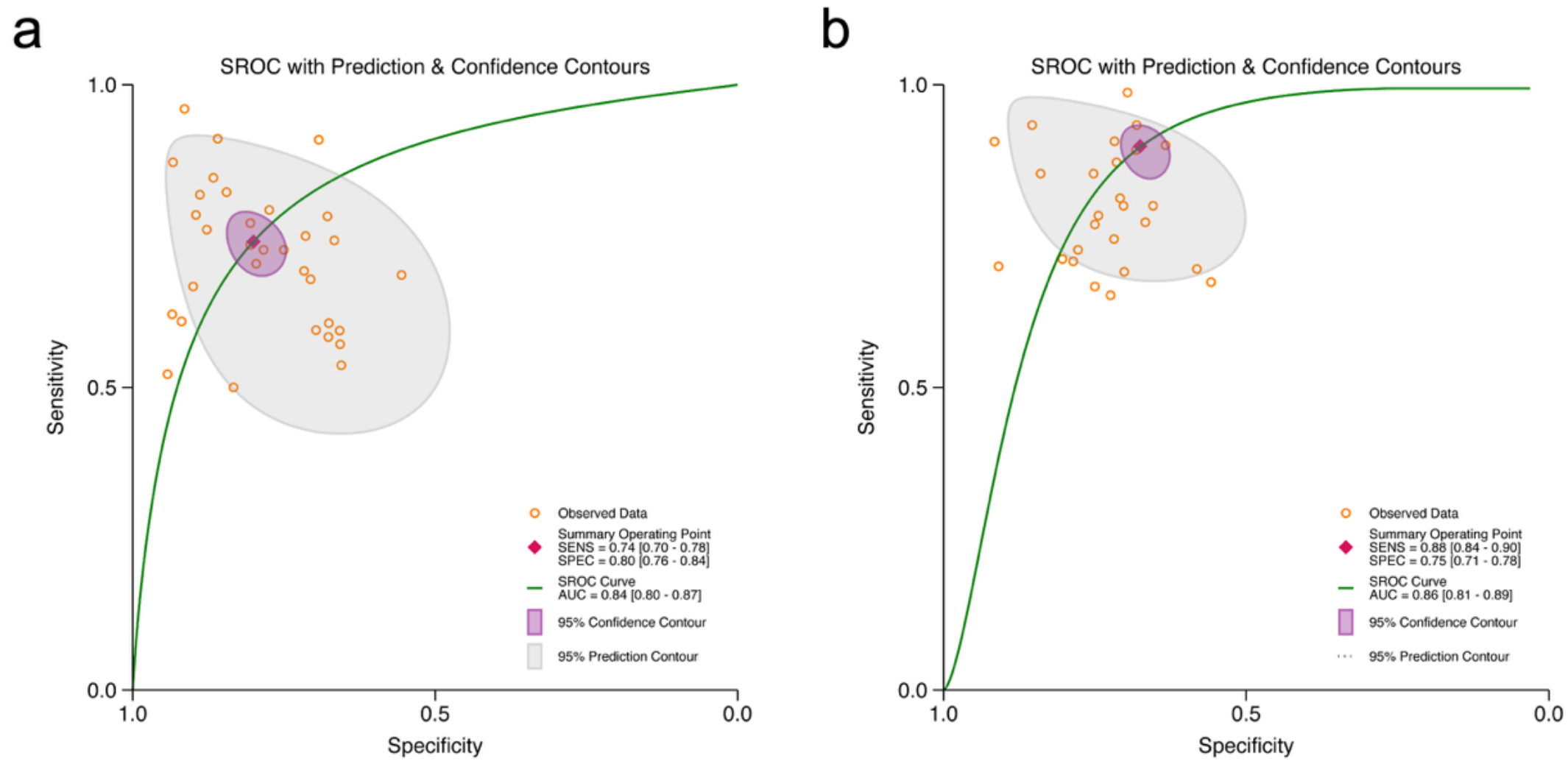

Supplementary Figure 83. SROC curves stratified by local metastatic ROI subgroup in Outcome prediction studies. Panels show SROC curves for studies of (a) intratumoral (7 studies with 33 tables) and (b) intra-peritumoral (3 studies with 26 tables). Abbreviations: SROC=summary receiver operating characteristic; SENS=summary sensitivity; SPEC=summary specificity.

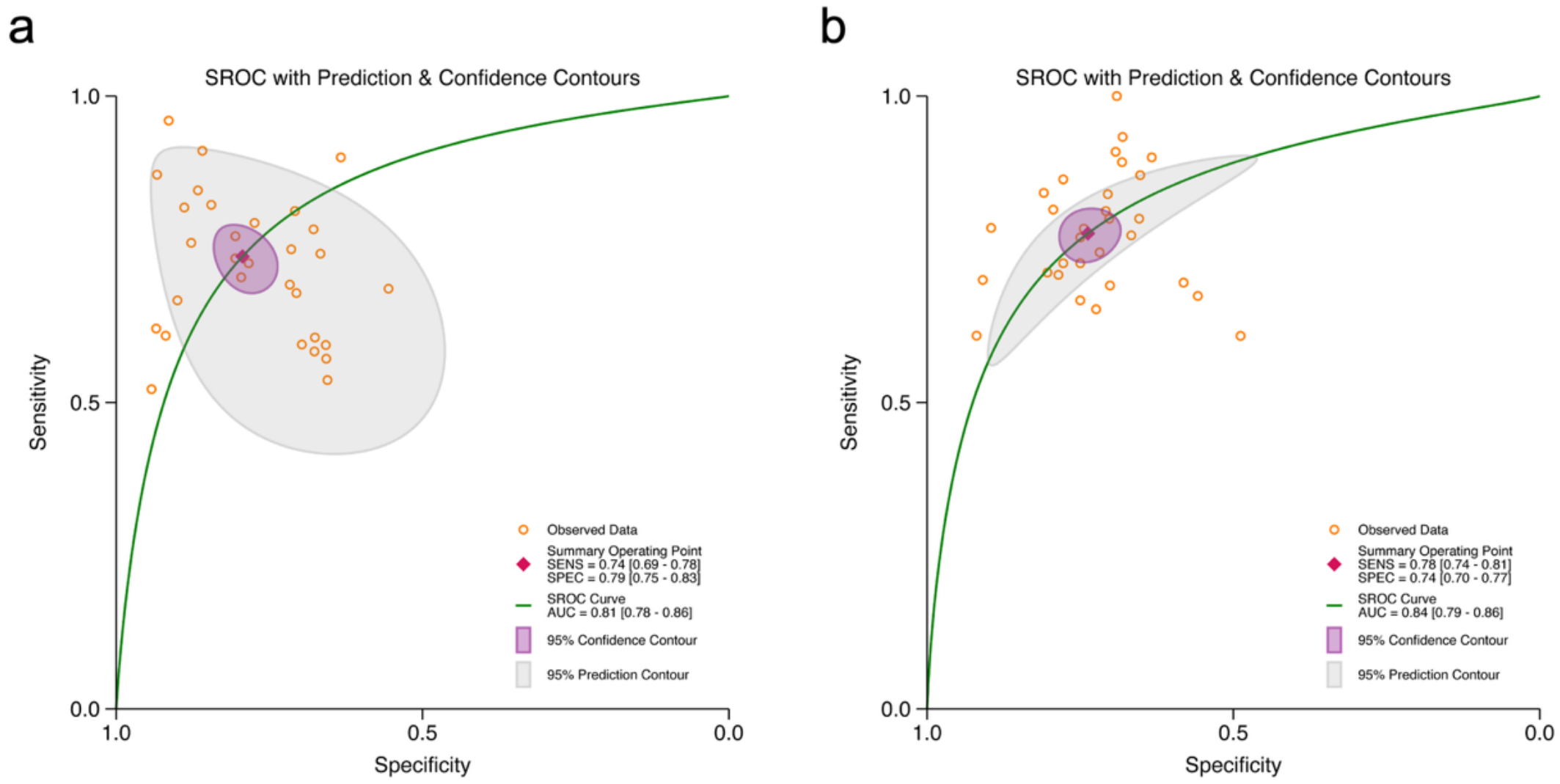

Supplementary Figure 84. SROC curves stratified by cohort type (internal vs external) in Outcome prediction studies. Panels show SROC curves for (a) internal validation (7 studies with 29 tables) and (b) external validation (3 studies with 30 tables). Abbreviations: SROC= summary receiver operating characteristic; SENS= summary sensitivity; SPEC= summary specificity.

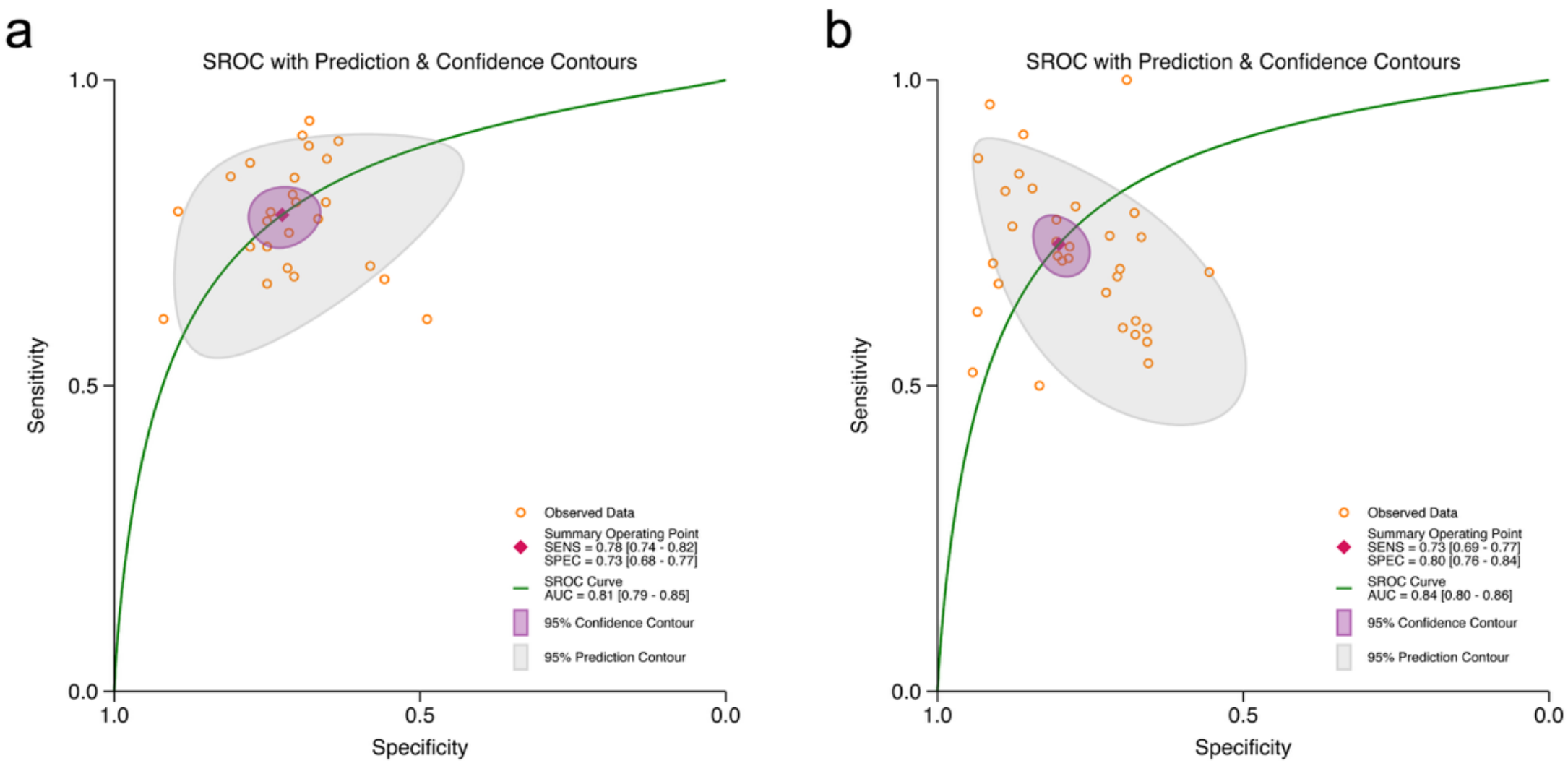

Supplementary Figure 85. SROC curves stratified by feature-source subgroup in Outcome prediction studies. Panels show SROC curves for (a) imaging-only studies (3 studies with 23 tables) and (b) imaging–clinical integrated studies (7 studies with 36 tables). Abbreviations: SROC=summary receiver operating characteristic; SENS=summary sensitivity; SPEC=summary specificity.

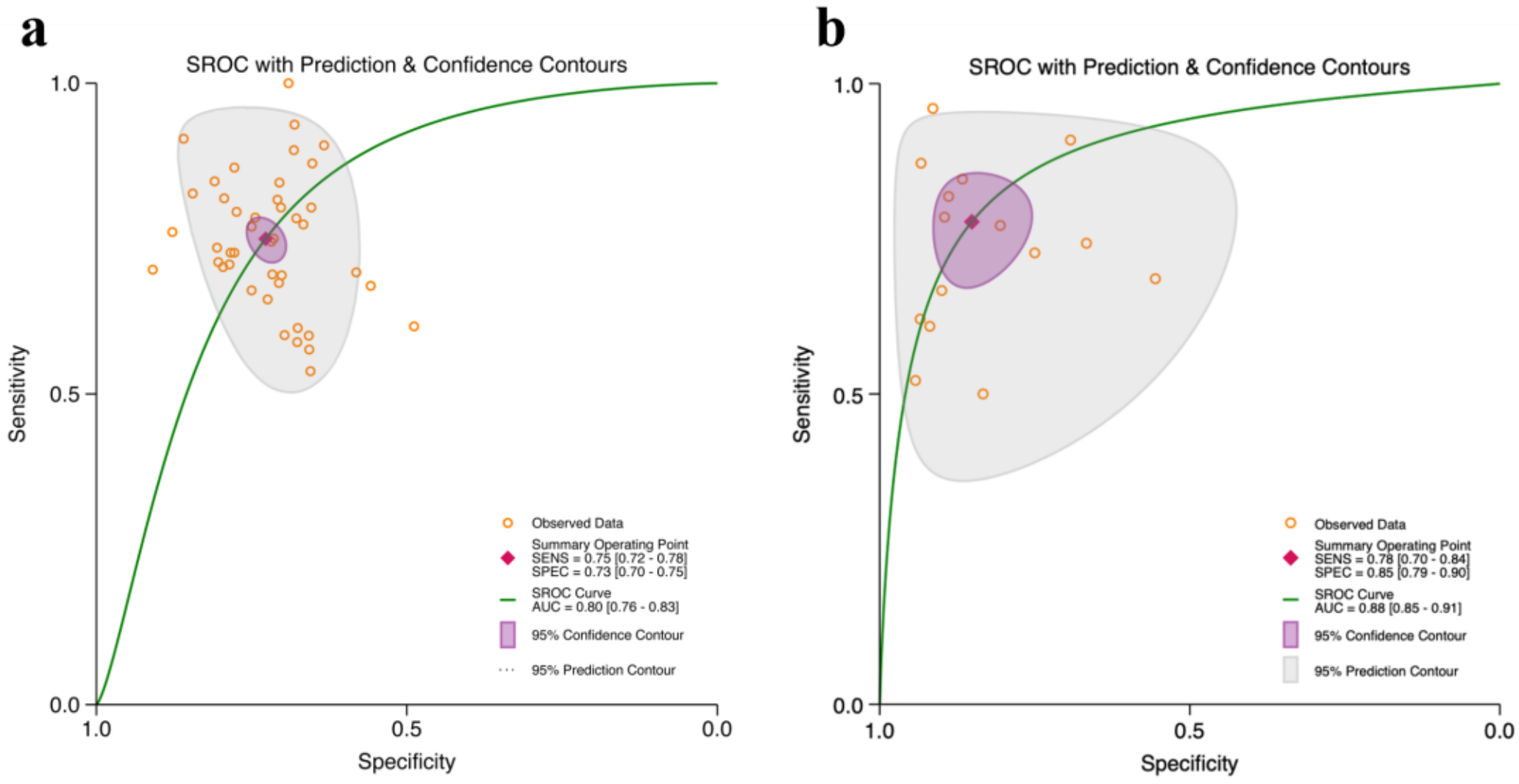

Supplementary Figure 86. SROC plots stratified by prognostic type classification in Outcome prediction studies. Panels show the SROC space for (a) response classification models (5 studies with 44 tables) and (b) survival classification models (5 studies with 15 tables). Abbreviations: SROC=summary receiver operating characteristic; SENS=summary sensitivity; SPEC=summary specificity.

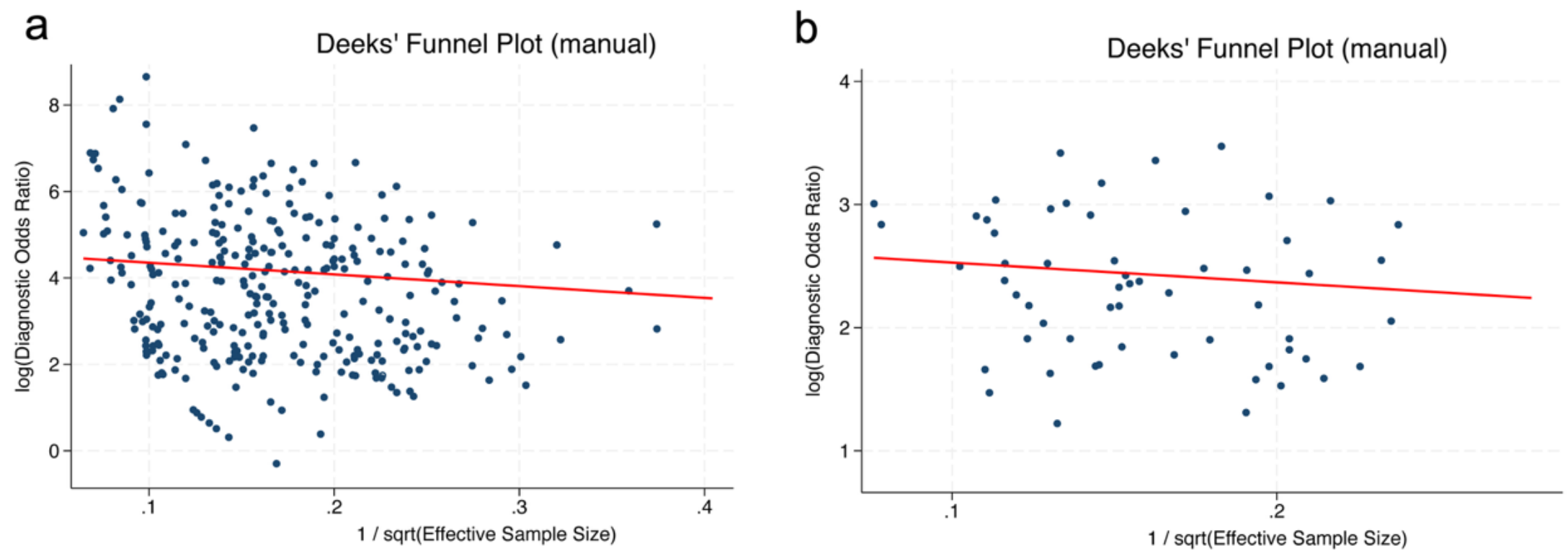

Supplementary Figure 87. Funnel plots for publication bias. Panels show Deeks' funnel plots for (a) the diagnostic-related meta-analysis dataset and (b) the Outcome prediction meta-analysis dataset. Abbreviations: DTA, diagnostic test accuracy.

Reference:

[1] R. Grossman, O. Haim, S. Abramov, B. Shofty, and M. Artzi, Differentiating small-cell lung cancer from non-small-cell lung cancer brain metastases based on MRI using efficientnet and transfer learning approach. Technology in cancer research & treatment 20 (2021) 15330338211004919.

[2] Y.W. Park, C. An, J. Lee, K. Han, D. Choi, S.S. Ahn, H. Kim, S.J. Ahn, J.H. Chang, and S.H. Kim, Diffusion tensor and postcontrast T1-weighted imaging radiomics to differentiate the epidermal growth factor receptor mutation status of brain metastases from non-small cell lung cancer. Neuroradiology 63 (2021) 343-352.

[3] L. Sun, S. Luan, L. Yu, H. Zhu, H. Dong, X. Liu, G. Tao, P. He, Q. Li, and W. Chen, Radiomic signatures of brain metastases on MRI: utility in predicting pathological subtypes of lung cancer. Translational Cancer Research 13 (2024) 6825.

[4] G. Wang, B. Wang, Z. Wang, W. Li, J. Xiu, Z. Liu, and M. Han, Radiomics signature of brain metastasis: prediction of EGFR mutation status. European radiology 31 (2021) 4538-4547.

[5] W.-F. Wu, K.-M. Lai, C.-H. Chen, B.-C. Wang, Y.-J. Chen, C.-W. Shen, K.-Y. Chen, E.C. Lin, and C.-C. Chen, Predicting the T790M mutation in non-small cell lung cancer (NSCLC) using brain metastasis MR radiomics: a study with an imbalanced dataset. Discover Oncology 15 (2024) 447.

[6] J. Xu, Y. Yang, Z. Gao, T. Song, Y. Ma, X. Yu, and C. Shi, Distinguishing EGFR mutation molecular subtypes based on MRI radiomics features of lung adenocarcinoma brain metastases. Clinical Neurology and Neurosurgery 240 (2024) 108258.

[7] C. Yang, Y. Fan, D. Zhao, Z. Wang, X. Wang, H. Wang, Y. Hu, L. He, J. Zhang, and Y. Wang, Habitat-based radiomics for predicting EGFR mutations in Exon 19 and 21 from brain metastasis. Academic Radiology 31 (2024) 3764-3773.

[8] J. Zhang, J. Jin, Y. Ai, K. Zhu, C. Xiao, C. Xie, and X. Jin, Differentiating the pathological subtypes of primary lung cancer for patients with brain metastases based on radiomics features from brain CT images. European Radiology 31 (2021) 1022-1028.

[9] L. Zheng, H. Xie, X. Luo, Y. Yang, Y. Zhang, Y. Li, S. Yin, H. Li, and C. Xie, Radiomic signatures for predicting EGFR mutation status in lung cancer brain metastases. Frontiers in Oncology 12 (2022) 931812.

[10] R. Cao, Z. Pang, X. Wang, Z. Du, H. Chen, J. Liu, Z. Yue, H. Wang, Y. Luo, and X. Jiang, Radiomics evaluates the EGFR mutation status from the brain metastasis: a multi-center study. Physics in Medicine & Biology 67 (2022) 125003.

[11] B.T. Chen, T. Jin, N. Ye, I. Mambetsariev, E. Daniel, T. Wang, C.W. Wong, R.C. Rockne, R. Colen, and A.I. Holodny, Radiomic prediction of mutation status based on MR imaging of lung cancer brain metastases. Magnetic resonance imaging 69 (2020) 49-56.

[12] Y. Fan, Z. Zhao, X. Wang, H. Ai, C. Yang, Y. Luo, and X. Jiang, Radiomics for prediction of response to EGFR-TKI based on metastasis/brain parenchyma (M/BP)-interface. La radiologia medica 127 (2022) 1342-1354.

[13] Y. Fan, L. He, H. Yang, Y. Wang, J. Su, S. Hou, Y. Luo, and X. Jiang, Preoperative MRI-based radiomics of brain metastasis to assess T790M resistance mutation after EGFR-TKI treatment in NSCLC. Journal of Magnetic Resonance Imaging 57 (2023) 1778-1787.

[14] O. Haim, S. Abramov, B. Shofty, C. Fanizzi, F. DiMeco, N. Avisdris, Z. Ram, M. Artzi, and R. Grossman, Predicting EGFR mutation status by a deep learning approach in patients with non-small cell lung cancer brain metastases. Journal of Neuro-Oncology 157 (2022) 63-69.

[15] Z. Huang, X. Tu, T. Yu, Z. Zhan, Q. Lin, and X. Huang, Peritumoural MRI radiomics signature of brain metastases can predict epidermal growth factor receptor mutation status in lung adenocarcinoma. Clinical Radiology 79 (2024) e305-e316.

[16] Y. Li, X. Lv, B. Wang, Z. Xu, Y. Wang, M. Sun, and D. Hou, Predicting EGFR T790M mutation in brain metastases using multisequence MRI-based radiomics signature. Academic Radiology 30 (2023) 1887-1895.

[17] A.-K. Meißner, R. Gutsche, N. Galldiks, M. Kocher, S.T. Jünger, M.-L. Eich, L. Nogova, T. Araceli, N.O. Schmidt, and M.I. Ruge, Radiomics for the non-invasive prediction of PD-L1 expression in patients with brain metastases secondary to non-small cell lung cancer. Journal of neuro-oncology 163 (2023) 597-605.

[18] J. Zhu, L. Zou, X. Xie, R. Xu, Y. Tian, and B. Zhang, 2.5 D deep learning based on multi-parameter MRI to differentiate primary lung cancer pathological subtypes in patients with brain metastases. European Journal of Radiology 180 (2024) 111712.

[19] Y. Li, X. Lv, C. Chen, R. Yu, B. Wang, D. Wang, and D. Hou, A deep learning model integrating multisequence MRI to predict EGFR mutation subtype in brain metastases from non-small cell lung cancer. European Radiology Experimental 8 (2024) 2.

[20] Z. Zhou, M. Wang, R. Zhao, Y. Shao, L. Xing, Q. Qiu, and Y. Yin, A multi-task deep learning model for EGFR genotyping prediction and GTV segmentation of brain metastasis. Journal of Translational Medicine 21 (2023) 788.

[21] R. Cao, L. Fu, B. Huang, Y. Liu, X. Wang, J. Liu, H. Wang, X. Jiang, Z. Yang, and X. Sha, Brain metastasis magnetic resonance imaging-based deep learning for predicting epidermal growth factor receptor (EGFR) mutation and subtypes in metastatic non-small cell lung cancer. Quantitative Imaging in Medicine and Surgery 14 (2024) 4749.

[22] Y. Fan, X. Wang, C. Yang, H. Chen, H. Wang, X. Wang, S. Hou, L. Wang, Y. Luo, and X. Sha, Brain-Tumor Interface-Based MRI Radiomics Models to Determine EGFR Mutation, Response to EGFR-TKI and T790M Resistance Mutation in Non-Small Cell Lung Carcinoma Brain Metastasis. Journal of Magnetic Resonance Imaging 58 (2023) 1838-1847.

[23] S.J. Ahn, H. Kwon, J.-J. Yang, M. Park, Y.J. Cha, S.H. Suh, and J.-M. Lee, Contrast-enhanced T1-weighted image radiomics of brain metastases may predict EGFR mutation status in primary lung cancer. Scientific reports 10 (2020) 8905.

[24] Z. Ouyang, G. Zhang, S. He, Q. Huang, L. Zhang, X. Duan, X. Zhang, Y. Liu, T. Ke, and J. Yang, CT and MRI bimodal radiomics for predicting EGFR status in NSCLC patients with brain metastases: A multicenter study. European Journal of Radiology 183 (2025) 111853.

[25] L. Sui, S. Chang, L. Xue, J. Wang, Y. Zhang, K. Yang, B.-L. Gao, and X. Yin, Deep learning based on enhanced MRI T1 imaging to differentiate small-cell and non-small-cell primary lung cancers in patients with brain metastases. Current Medical Imaging Reviews 19 (2023) 1541-1548.

[26] P. Cao, X. Jia, X. Wang, L. Fan, Z. Chen, Y. Zhao, J. Zhu, and Q. Wen, Deep learning radiomics for the prediction of epidermal growth factor receptor mutation status based on MRI in brain metastasis from lung adenocarcinoma patients. BMC cancer 25 (2025) 443.

[27] Z. Li, Y. Mao, H. Li, G. Yu, H. Wan, and B. Li, Differentiating brain metastases from different pathological types of lung cancers using texture analysis of T1 postcontrast MR. Magnetic resonance in medicine 76 (2016) 1410-1419.
[28] Y. Li, X. Lv, B. Wang, Z. Xu, Y. Wang, S. Gao, and D. Hou, Differentiating EGFR from ALK mutation status using radiomics signature based on MR sequences of brain metastasis. European Journal of Radiology 155 (2022) 110499.
[29] Y. Li, R. Yu, H. Chang, W. Yan, D. Wang, F. Li, Y. Cui, Y. Wang, X. Wang, and Q. Yan, Identifying pathological subtypes of brain metastasis from lung cancer using MRI-based deep learning approach: A multicenter study. Journal of Imaging Informatics in Medicine 37 (2024) 976-987.
[30] Y. Li, Y. Jin, Y. Wang, W. Liu, W. Jia, and J. Wang, MR-based radiomics predictive modelling of EGFR mutation and HER2 overexpression in metastatic brain adenocarcinoma: a two-centre study. Cancer Imaging 24 (2024) 65.
[31] Y. Fan, X. Wang, Y. Dong, E. Cui, H. Wang, X. Sun, J. Su, Y. Luo, T. Yu, and X. Jiang, Multiregional radiomics of brain metastasis can predict response to EGFR-TKI in metastatic NSCLC. European Radiology 33 (2023) 7902-7912.
[32] G. Tulum, Novel radiomic features versus deep learning: differentiating brain metastases from pathological lung cancer types in small datasets. The British Journal of Radiology 96 (2023) 20220841.
[33] F.-Y. Zhu, Y.-F. Sun, X.-P. Yin, Y. Zhang, L.-H. Xing, Z.-P. Ma, L.-Y. Xue, and J.-N. Wang, Using machine learning-based radiomics to differentiate between glioma and solitary brain metastasis from lung cancer and its subtypes. Discover Oncology 14 (2023) 224.
[34] Y. Hu, Y. Geng, H. Wang, H. Chen, Z. Wang, L. Fu, B. Huang, and W. Jiang, Improved prediction of epidermal growth factor receptor status by combined radiomics of primary nonsmall-cell lung cancer and distant metastasis. Journal of Computer Assisted Tomography 48 (2024) 780-788.
[35] M. Artzi, I. Bressler, and D. Ben Bashat, Differentiation between glioblastoma, brain metastasis and subtypes using radiomics analysis. Journal of Magnetic Resonance Imaging 50 (2019) 519-528.
[36] Y. Han, L. Zhang, S. Niu, S. Chen, B. Yang, H. Chen, F. Zheng, Y. Zang, H. Zhang, and Y. Xin, Differentiation between glioblastoma multiforme and metastasis from the lungs and other sites using combined clinical/routine MRI radiomics. Frontiers in cell and developmental biology 9 (2021) 710461.
[37] C. Chen, X. Ou, J. Wang, W. Guo, and X. Ma, Radiomics-based machine learning in differentiation between glioblastoma and metastatic brain tumors. Frontiers in oncology 9 (2019) 806.
[38] Y. Huang, S. Huang, and Z. Liu, Multi-task learning-based feature selection and classification models for glioblastoma and solitary brain metastases. Frontiers in Oncology 12 (2022) 1000471.
[39] S. Duan, W. Dong, Y. Hua, Y. Zheng, Z. Ren, G. Cao, F. Wu, T. Rong, and B. Liu, Accurate differentiation of spinal tuberculosis and spinal metastases using MR-based deep learning algorithms. Infection and Drug Resistance (2023) 4325-4334.

[40] N. Lang, Y. Zhang, E. Zhang, J. Zhang, D. Chow, P. Chang, H.J. Yu, H. Yuan, and M.-Y. Su, Differentiation of spinal metastases originated from lung and other cancers using radiomics and deep learning based on DCE-MRI. Magnetic resonance imaging 64 (2019) 4.
[41] Y. Cheng, H. Wang, W. Yuan, H. Wang, Y. Zhu, H. Chen, and W. Jiang, Combined radiomics of primary tumour and bone metastasis improve the prediction of EGFR mutation status and response to EGFR-TKI therapy for NSCLC. Physica Medica 116 (2023) 103177.
[42] R. Cao, Y. Dong, X. Wang, M. Ren, X. Wang, N. Zhao, T. Yu, L. Zhang, Y. Luo, and E.-N. Cui, MRI-based radiomics nomogram as a potential biomarker to predict the EGFR mutations in exon 19 and 21 based on thoracic spinal metastases in lung adenocarcinoma. Academic radiology 29 (2022) e9-e17.
[43] R. Cao, H. Chen, H. Wang, Y. Wang, E.-N. Cui, and W. Jiang, Comprehensive analysis of prediction of the EGFR mutation and subtypes based on the spinal metastasis from primary lung adenocarcinoma. Frontiers in oncology 13 (2023) 1154327.
[44] Y. Fan, Y. Dong, H. Yang, H. Chen, Y. Yu, X. Wang, X. Wang, T. Yu, Y. Luo, and X. Jiang, Subregional radiomics analysis for the detection of the EGFR mutation on thoracic spinal metastases from lung cancer. Physics in Medicine & Biology 66 (2021) 215008.
[45] Y. Fan, Y. Dong, H. Wang, H. Wang, X. Sun, X. Wang, P. Zhao, Y. Luo, and X. Jiang, Development and externally validate MRI-based nomogram to assess EGFR and T790M mutations in patients with metastatic lung adenocarcinoma. European radiology 32 (2022) 6739-6751.
[46] Y. Fan, Y. Dong, X. Sun, H. Wang, P. Zhao, H. Wang, and X. Jiang, Development and validation of MRI-based radiomics signatures as new markers for preoperative assessment of EGFR mutation and subtypes from bone metastases. BMC cancer 22 (2022) 889.
[47] X. Jiang, M. Ren, X. Shuang, H. Yang, D. Shi, Q. Lai, and Y. Dong, Multiparametric MRI-based radiomics approaches for preoperative prediction of EGFR mutation status in spinal bone metastases in patients with lung adenocarcinoma. Journal of Magnetic Resonance Imaging 54 (2021) 497-507.
[48] T. Jiang, X. Sun, Y. Dong, W. Guo, H. Wang, Z. Yue, Y. Luo, and X. Jiang, Deep learning for preoperative prediction of the EGFR mutation and subtypes based on the MRI image of spinal metastasis from primary NSCLC. Biomedical Signal Processing and Control 79 (2023) 104084.
[49] M. Ren, H. Yang, Q. Lai, D. Shi, G. Liu, X. Shuang, J. Su, L. Xie, Y. Dong, and X. Jiang, MRI-based radiomics analysis for predicting the EGFR mutation based on thoracic spinal metastases in lung adenocarcinoma patients. Medical physics 48 (2021) 5142-5151.
[50] H. Wang, Y. Chen, J. Qiu, J. Xie, W. Lu, J. Ma, and M. Jia, Machine learning based on SPECT/CT to differentiate bone metastasis and benign bone lesions in lung malignancy patients. Medical Physics 51 (2024) 2578-2588.
[51] S. Hou, H. Wang, X. Wang, H. Chen, B. Zhou, R. Meng, X. Sha, S. Chang, H. Wang, and W. Jiang, Tumor-liver interface in MRI of liver metastasis enables prediction of

EGFR mutation in patients with lung cancer: A proof-of-concept study. Medical Physics 51 (2024) 1083-1091.

[52] S. Hou, Y. Fan, X. Wang, J. Su, M. Ren, Y. Wu, J. Zhou, M. Qu, Y. Luo, and W. Jiang, Radiomics for detection of the EGFR mutation in liver metastatic NSCLC. Academic radiology 30 (2023) 1039-1046.

[53] H. Qi, Y. Hou, Z. Zheng, M. Zheng, Q. Qiao, Z. Wang, X. Sun, and L. Xing, Clinical characteristics and MRI based radiomics nomograms can predict iPFS and short-term efficacy of third-generation EGFR-TKI in EGFR-mutated lung adenocarcinoma with brain metastases. BMC cancer 24 (2024) 362.

[54] J. Xu, P. Wang, Y. Li, X. Shi, T. Yin, J. Yu, and F. Teng, Development and validation of an MRI-Based nomogram to predict the effectiveness of immunotherapy for brain metastasis in patients with non-small cell lung cancer. Frontiers in Immunology 15 (2024) 1373330.

[55] S. Zhao, D. Hou, X. Zheng, W. Song, X. Liu, S. Wang, L. Zhou, X. Tao, L. Lv, and Q. Sun, MRI radiomic signature predicts intracranial progression-free survival in patients with brain metastases of ALK-positive non-small cell lung cancer. Translational Lung Cancer Research 10 (2021) 368.

[56] J. Qu, T. Zhang, X. Zhang, W. Zhang, Y. Li, Q. Gong, L. Yao, and S. Lui, MRI radiomics for predicting intracranial progression in non-small-cell lung cancer patients with brain metastases treated with epidermal growth factor receptor tyrosine kinase inhibitors. Clinical Radiology 79 (2024) e582-e591.

[57] H. Qi, Y. Hou, Z. Zheng, M. Zheng, X. Sun, and L. Xing, MRI radiomics predicts the efficacy of EGFR-TKI in EGFR-mutant non-small-cell lung cancer with brain metastasis. Clinical Radiology 79 (2024) 515-525.

[58] J.H. Park, B.S. Choi, J.H. Han, C.-Y. Kim, J. Cho, Y.J. Bae, L. Sunwoo, and J.H. Kim, MRI texture analysis for the prediction of stereotactic radiosurgery outcomes in brain metastases from lung cancer. Journal of Clinical Medicine 10 (2021) 237.

[59] X. Lv, Y. Li, B. Wang, Y. Wang, Y. Pan, C. Li, and D. Hou, Multisequence MRI-based radiomics analysis for early prediction of the risk of T790M resistance in new brain metastases. Quantitative Imaging in Medicine and Surgery 13 (2023) 8599.

[60] C.-Y. Liao, C.-C. Lee, H.-C. Yang, C.-J. Chen, W.-Y. Chung, H.-M. Wu, W.-Y. Guo, R.-S. Liu, and C.-F. Lu, Enhancement of radiosurgical treatment outcome prediction using MRI radiomics in patients with non-small cell lung cancer brain metastases. Cancers 13 (2021) 4030.

[61] Y. Sun, F. Liang, J. Yang, Y. Liu, Z. Shen, C. Zhou, and Y. Xia, Pilot study: radiomic analysis for predicting treatment response to whole-brain radiotherapy combined temozolomide in lung cancer brain metastases. Frontiers in Oncology 14 (2024) 1395313.

[62] T.-W. Wang, H.-S. Chao, H.-Y. Chiu, C.-F. Lu, C.-Y. Liao, Y. Lee, J.-R. Chen, T.-H. Shiao, Y.-M. Chen, and Y.-T. Wu, Radiomics of metastatic brain tumor as a predictive image biomarker of progression-free survival in patients with non-small-cell lung cancer with brain metastasis receiving tyrosine kinase inhibitors. Translational Oncology 39 (2024) 101826.

[63] B.T. Chen, T. Jin, N. Ye, I. Mambetsariev, T. Wang, C.W. Wong, Z. Chen, R.C. Rockne, R.R. Colen, and A.I. Holodny, Predicting survival duration with MRI radiomics of brain metastases from non-small cell lung cancer. Frontiers in Oncology 11 (2021) 621088.

[64] R. Gutsche, P. Lohmann, M. Hoevels, D. Ruess, N. Galldiks, V. Visser-Vandewalle, H. Treuer, M. Ruge, and M. Kocher, Radiomics outperforms semantic features for prediction of response to stereotactic radiosurgery in brain metastases. Radiotherapy and Oncology 166 (2022) 37-43.

Section and Topic	Item #	Checklist item	Location where item is reported
**TITLE**			
Title	1	Identify the report as a systematic review.	Title Page
**ABSTRACT**			
Abstract	2	See the PRISMA 2020 for Abstracts checklist.	Abstract, Page 2
**INTRODUCTION**			
Rationale	3	Describe the rationale for the review in the context of existing knowledge.	Introduction, Page 3-4
Objectives	4	Provide an explicit statement of the objective(s) or question(s) the review addresses.	Introduction, Page 4
**METHODS**			
Eligibility criteria	5	Specify the inclusion and exclusion criteria for the review and how studies were grouped for the syntheses.	Methods, Page 13
Information sources	6	Specify all databases, registers, websites, organisations, reference lists and other sources searched or consulted to identify studies. Specify the date when each source was last searched or consulted.	Supplementary Note 1
Search strategy	7	Present the full search strategies for all databases, registers and websites, including any filters and limits used.	Supplementary Note 1
Selection process	8	Specify the methods used to decide whether a study met the inclusion criteria of the review, including how many reviewers screened each record and each report retrieved, whether they worked independently, and if applicable, details of automation tools used in the process.	Methods, Page 13
Data collection process	9	Specify the methods used to collect data from reports, including how many reviewers collected data from each report, whether they worked independently, any processes for obtaining or confirming data from study investigators, and if applicable, details of automation tools used in the process.	Methods, Page 14
Data items	10a	List and define all outcomes for which data were sought. Specify whether all results that were compatible with each outcome domain in each study were sought (e.g. for all measures, time points, analyses), and if not, the methods used to decide which results to collect.	Methods, Page 14

Section and Topic	Item #	Checklist item	Location where item is reported
	10b	List and define all other variables for which data were sought (e.g. participant and intervention characteristics, funding sources). Describe any assumptions made about any missing or unclear information.	-
Study risk of bias assessment	11	Specify the methods used to assess risk of bias in the included studies, including details of the tool(s) used, how many reviewers assessed each study and whether they worked independently, and if applicable, details of automation tools used in the process.	Methods, Page 14
Effect measures	12	Specify for each outcome the effect measure(s) (e.g. risk ratio, mean difference) used in the synthesis or presentation of results.	
Synthesis methods	13a	Describe the processes used to decide which studies were eligible for each synthesis (e.g. tabulating the study intervention characteristics and comparing against the planned groups for each synthesis (item #5)).	Methods, Page 14
	13b	Describe any methods required to prepare the data for presentation or synthesis, such as handling of missing summary statistics, or data conversions.	Methods, Page 14-15
	13c	Describe any methods used to tabulate or visually display results of individual studies and syntheses.	Methods, Page 14-15
	13d	Describe any methods used to synthesize results and provide a rationale for the choice(s). If meta-analysis was performed, describe the model(s), method(s) to identify the presence and extent of statistical heterogeneity, and software package(s) used.	Methods, Page 15
	13e	Describe any methods used to explore possible causes of heterogeneity among study results (e.g. subgroup analysis, meta-regression).	Methods, Page 14
	13f	Describe any sensitivity analyses conducted to assess robustness of the synthesized results.	Methods, Page 14
Reporting bias assessment	14	Describe any methods used to assess risk of bias due to missing results in a synthesis (arising from reporting biases).	Methods, Page 15
Certainty assessment	15	Describe any methods used to assess certainty (or confidence) in the body of evidence for an outcome.	Methods, Page 15

Section and Topic	Item #	Checklist item	Location where item is reported
**RESULTS**			
Study selection	16a	Describe the results of the search and selection process, from the number of records identified in the search to the number of studies included in the review, ideally using a flow diagram.	Results, Page 4
	16b	Cite studies that might appear to meet the inclusion criteria, but which were excluded, and explain why they were excluded.	Results, Page 4
Study characteristics	17	Cite each included study and present its characteristics.	Results, Page 5
Risk of bias in studies	18	Present assessments of risk of bias for each included study.	Results, Page 6
Results of individual studies	19	For all outcomes, present, for each study: (a) summary statistics for each group (where appropriate) and (b) an effect estimate and its precision (e.g. confidence/credible interval), ideally using structured tables or plots.	Results, Page 4-8
Results of syntheses	20a	For each synthesis, briefly summarise the characteristics and risk of bias among contributing studies.	Results, Page 8; Supplementary Note 3; Supplementary Figure 2
	20b	Present results of all statistical syntheses conducted. If meta-analysis was done, present for each the summary estimate and its precision (e.g. confidence/credible interval) and measures of statistical heterogeneity. If comparing groups, describe the direction of the effect.	Results, Page 4-8
	20c	Present results of all investigations of possible causes of heterogeneity among study results.	Results, Page 4-8
	20d	Present results of all sensitivity analyses conducted to assess the robustness of the synthesized results.	Results, Page 4-8
Reporting biases	21	Present assessments of risk of bias due to missing results (arising from reporting biases) for each synthesis assessed.	Results, Page 4-8; Supplementary Note 3; Supplementary Figure 87
Certainty of evidence	22	Present assessments of certainty (or confidence) in the body of evidence for each outcome assessed.	Results, Page 6-8;

Section and Topic	Item #	Checklist item	Location where item is reported
			Supplementary Note 3; Supplementary Figure 87
**DISCUSSION**			
Discussion	23a	Provide a general interpretation of the results in the context of other evidence.	Discussion, Page 8-10
	23b	Discuss any limitations of the evidence included in the review.	Discussion, Page 9-11
	23c	Discuss any limitations of the review processes used.	Discussion, Page 11-13
	23d	Discuss implications of the results for practice, policy, and future research.	Discussion, Page 11-13
**OTHER INFORMATION**			
Registration and protocol	24a	Provide registration information for the review, including register name and registration number, or state that the review was not registered.	PROSPERO (Registration Number: 1056539)
Support	25	Describe sources of financial or non-financial support for the review, and the role of the funders or sponsors in the review.	This work was supported by the National Key R&D Program of China: BTIT (Grant NO.2022YFF1202803), Science and Technology Joint Program Fund Project of Liaoning (2023JH2/101700175) and General Program from Department of Education

Section and Topic	Item #	Checklist item	Location where item is reported
			of Liaoning Province (JYTMS20230132).
Competing interests	26	Declare any competing interests of review authors.	No competing interests
Availability of data, code and other materials	27	Report which of the following are publicly available and where they can be found: template data collection forms; data extracted from included studies; data used for all analyses; analytic code; any other materials used in the review.	The template data collection forms, extracted data from included studies, data used for all analyses, analytic code, and any other materials used in the review are available upon reasonable request from the corresponding author
